# Identification and engineering of potent cyclic peptides with selective or promiscuous binding through biochemical profiling and bioinformatic data analysis†

**DOI:** 10.1039/d3cb00168g

**Published:** 2023-11-14

**Authors:** Thomas P. Smith, Bhaskar Bhushan, Daniele Granata, Christian S. Kaas, Birgitte Andersen, Klaas W. Decoene, Qiansheng Ren, Haimo Liu, Xinping Qu, Yang Yang, Jia Pan, Quijia Chen, Martin Münzel, Akane Kawamura

**Affiliations:** a Chemistry – School of Natural and Environmental Sciences, Newcastle University, Newcastle upon Tyne NE1 7RU UK akane.kawamura@ncl.ac.uk; b Department of Chemistry, University of Oxford OX1 3TA UK; c Digital Science and Innovation, Novo Nordisk A/S Novo Nordisk Park 2760 Måløv Denmark; d Global Research Technologies, Novo Nordisk A/S Novo Nordisk Park 2760 Måløv Denmark myzm@novonordisk.com; e Global Drug Discovery, Novo Nordisk A/S Novo Nordisk Park 2760 Måløv Denmark; f Novo Nordisk Research Center China, Novo Nordisk A/S Shengmingyuan West Ring Rd, Changping District Beijing China

## Abstract

As our understanding of biological systems grows, so does the need to selectively target individual or multiple members of specific protein families in order to probe their function. Many targets of current biological and pharmaceutical interest are part of a large family of closely related proteins and achieving ligand selectivity often remains either an elusive or time-consuming endeavour. Cyclic peptides (CPs) occupy a key niche in ligand space, able to achieve high affinity and selectivity while retaining synthetic accessibility. *De novo* cyclic peptide ligands can be rapidly generated against a given target using mRNA display. In this study we harness mRNA display technology and the wealth of next generation sequencing (NGS) data generated to explore both experimental approaches and bioinformatic, statistical data analysis of peptide enrichment in cross-screen selections to rapidly generate high affinity CPs with differing intra-family protein selectivity profiles against fibroblast growth factor receptor (FGF-R) family proteins. Using these methods, CPs with distinct selectivity profiles can be generated which can serve as valuable tool compounds to decipher biological questions.

## Abstract 2

In this work different methods stemming from mRNA display were investigated toward developing potent binding cyclic peptides with distinct selectivity profiles against targets of a related protein family.

## Graphical abstract

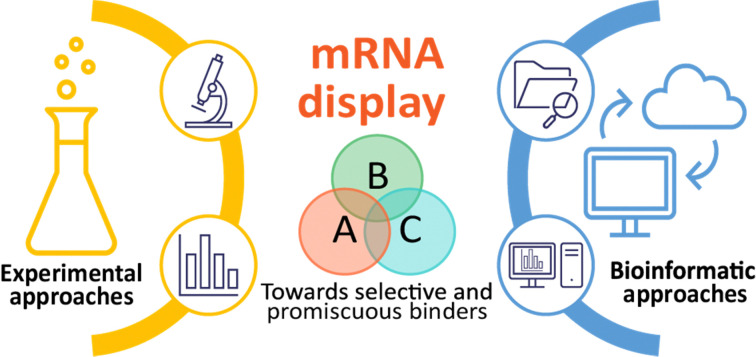

## Introduction

In the study of biological systems, it is crucial to be able to selectively modulate individual targets or a family of targets within complex networks. In practice this is achieved either genetically by affecting target expression levels (*e.g.* RNAi, or CRISPR) or chemically by influencing the function or interactions of protein targets (small molecules, antibodies, PROTACs), with each approach being associated with specific advantages and disadvantages. Cyclic peptides (CPs) are an emerging compound class that combines the specificity and affinity of antibodies while being smaller in size and with the synthetic tractability of classical small molecules. Modern display technologies such as phage, ribosome or mRNA display enable the rapid identification of potent and selective CP ligands, for subsequent use in biological experiments or drug discovery efforts.^1–3^ In a previous study, we demonstrated the use of an efficient, high throughput strategy from next generation sequencing (NGS) hits to identifying high affinity CP binders.^4^ In this study, we aimed to investigate different avenues to develop CP binders against a protein family, with differing target binding profiles which either interact with a target selectively or provide a specific interaction profile (binding to only a subset of targets). We used mRNA-display to generate ligands for fibroblast growth factor receptors (FGF-Rs), a family of tyrosine kinases which are important targets in biomedical research, such as in cancer and metabolic disease.^5,6^

**Scheme 1:**
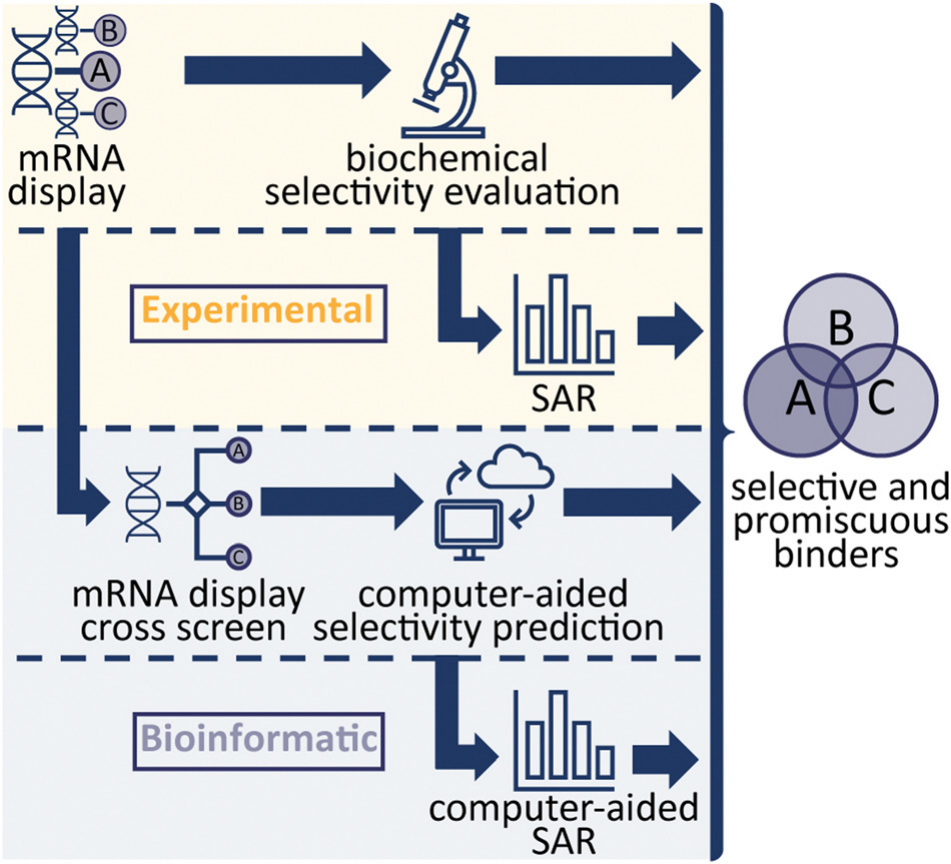
Workflows investigated towards generating peptide binders with different selectivity profiles for FGF-R family proteins. The first two routes (top, highlighted in yellow) followed traditional experimental approaches utilising firstly mRNA display and affinity measurements, and secondly performing SAR analysis of hit peptides to refine selectivity. The latter two methods (bottom, in grey) aim to predict selectivity *via* statistical data analysis of mRNA display utilising peptide libraries in mRNA display cross screens against multiple targets.

Four FGF-R families are encoded in the human genome sharing 56–71% sequence homology, with 48 splice variants existing on the proteome level.^7,8^ Each FGF-R consists of an extracellular ligand binding domain, a single transmembrane helix domain and an intracellular tyrosine kinase domain. These receptors are bound by a total of 22 individual fibroblast growth factor (FGF) ligands which exhibit overlapping FGF-R selectivity.^9^ A subset of the FGFs also interact with their targets through co-receptors (alpha and beta klotho).^10^ In nature, no subfamily selective ligands are found, hence selective antagonists of individual FGF-Rs would be valuable research tools to decipher their exact biological role. Numerous small molecule inhibitors of FGF receptors have been reported, however these are primarily pan-FGF-R binders or selectively target FGF-R4 only and engage the target *via* the intracellular kinase domain.^11^ Previous work on FGF-Rs has yielded a number of binding peptides, but to the best of our knowledge comprehensive analysis of their selectivity has rarely been performed.^12,13^ Therefore, the FGF-Rs were selected as an ideal target family to investigate optimal routes towards generating CP ligands with different binding profiles. In total, we explored four methodologies (Scheme 1), all focussing on the mRNA display technology to evaluate the different approaches.^4^ First, we explored traditional experimental routes towards peptide binders by performing mRNA display against each target (namely FGF-R1C, R3C and R4), synthesising the CPs based on NGS clustering and hits prioritisation, then testing the selectivity in biochemical assays.^4^ In a second, purely experimental approach, we selected individual peptide binders from our original biochemical assays and generated detailed structure–activity relationship (SAR) data in an attempt to engineer the interaction profile. The final two methods aimed to utilise bioinformatic analysis of peptide enrichment to predict sequence selectivity; NGS data from mRNA display cross screens, in which the peptide-RNA libraries are exposed to multiple targets during selection, were used to calculate selectivity profiles of individual peptides. Finally, NGS data from original selections were used to generate an anticipated SAR picture. Potent peptides with a variety of interaction profiles were found, with statistical, bioinformatical approaches being able to successfully predict binding.

## Results and discussion

### mRNA display and hit follow-up

**Fig. 1:**
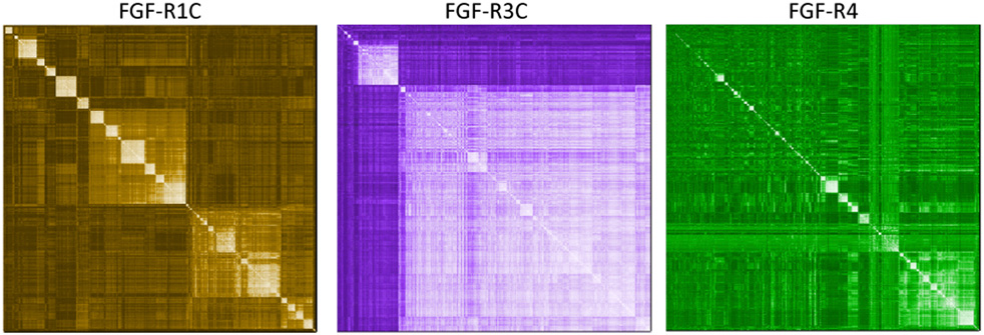
mRNA display campaigns against three separate FGF-R targets. NGS hierarchical clustering diagrams of sequences from the 5th selection round. Peptide similarity for each enriched hits are compared individually for each target with low (dark) to high (light) similarity scores shaded as a heat-map: FGF-R1C (left), FGF-R3C (middle), and FGF-R4 (right).

In a first approach to selective ligands, we embarked on the classical route and performed three individual selections against the extracellular domains (ECDs) of the closely related FGF-R1C, FGF-R3C and FGF-R4, utilising a DNA library comprising NNK codons (4–12 mer variable region) with Cys–Cys disulfide cyclisation using published procedures.^4^ However, in the selection against FGF-R4 we initially noticed very large recovery, which we ascribed to binding of the oligonucleotide region of the peptide–oligonucleotide conjugate to a putative heparin binding site in the receptor. This interaction was blocked by the addition of total yeast RNA extract to the selection buffer. After 5 rounds of selection (Fig. S11, ESI†) against the individual targets, the total recovered cDNA was analysed by next generation sequencing (NGS) and the resulting sequence lists were clustered as described previously (for complete list of enriched peptides and NGS read counts see Supporting Dataset – next generation sequencing).^4^ Despite the close relationship of the receptors and the use of the same starting library, the three selections showed very distinct result sets (Fig. 1). While the FGF-R1C binders clustered into many peptide families with clearly visible subfamilies, the selection against FGF-R3C was dominated by one peptide family. FGF-R4 yielded smaller clusters, together with a significant amount of un-clustered peptides (possibly reflecting residual non-specific enrichment of the RNA instead of the peptide region in the selection). Peptide sequence lengths showed greater consistency between each selection with an overwhelming majority comprising a 10–12 mer variable region (98%, 99%, and 96% 10–12 mer variable region sequences for R1C, R3C and R4 respectively, top 1000 sequences with the highest read count in round five). Based on the clustering analysis and enrichment scores for the subclusters, we chose 98 unique sequences in total for an initial high throughput characterisation (Table S3, ESI†).^4^ Peptides were synthesized in a 96-well format with a C-terminal flag tag to ensure solubility (C-terminal flag tag has been previously confirmed to not impact binding).^4^ After cleavage from the resin, the peptides were cyclised *via* disulfide bond formation (50 mM HEPES, 20% DMSO, pH 7.4, overnight) and their purity, identity and concentration determined by UPLC-CAD-MS. Finally, we investigated the binding properties of the crude CPs against all three receptors using single concentration biolayer interferometry (BLI) experiments (for complete results see ESI†). The majority (70%) of sequences were found to have a *K*_D_ <1000 nM against at least one FGF receptor. Of these, 24 representative peptides that appeared to show selective or promiscuous binding properties (Table 1) were purified and subjected to rigorous multi concentration BLI experiments to determine their binding across all three FGF-Rs (see Fig. S18, ESI† for exemplary cross-screening BLI kinetic curves of selected peptides. See Fig. S19–S21, ESI† for all BLI binding traces). Furthermore, the same panel was tested for FGF21 competitive binding to the ectodomain of FGFR1c/KLB and FGFR3c/BKL and FGFR4/KLB complexes (Fig. S25, ESI†).^14^

**Table 1:** Biochemical data of purified peptides from the NNK library mRNA display selections. Sequences were chosen from the top binding hits of each selection from single concentration BLI data while maintaining sequence diversity. *K*_D_ was determined by multi-concentration BLI and IC_50_ determined by Alphascreen with respective receptor and natural FGF-ligand. *K*_D_ < 1000 nM were considered as binders. Affinity (*K*_D_ or IC_50_) graded on a colour scheme from low affinity (red) to high affinity (blue). Peptide names are indicated numerically by FGF-R target that the sequence was originally enriched against during mRNA display. CPs were considered selective where *K*_D_ for one FGFR is >100 fold over others. All peptides synthesised with a C-terminal flag tag (GSGSDYKDDDDK-NH_2_) and disulfide cyclised (see Table S1, ESI for complete list of identities). *1C.4 and 1C.5 represent disulfide bond isomers of the same peptide. Other peptides with the potential to form multiple disulfide bond isomers were isolated as a single peak

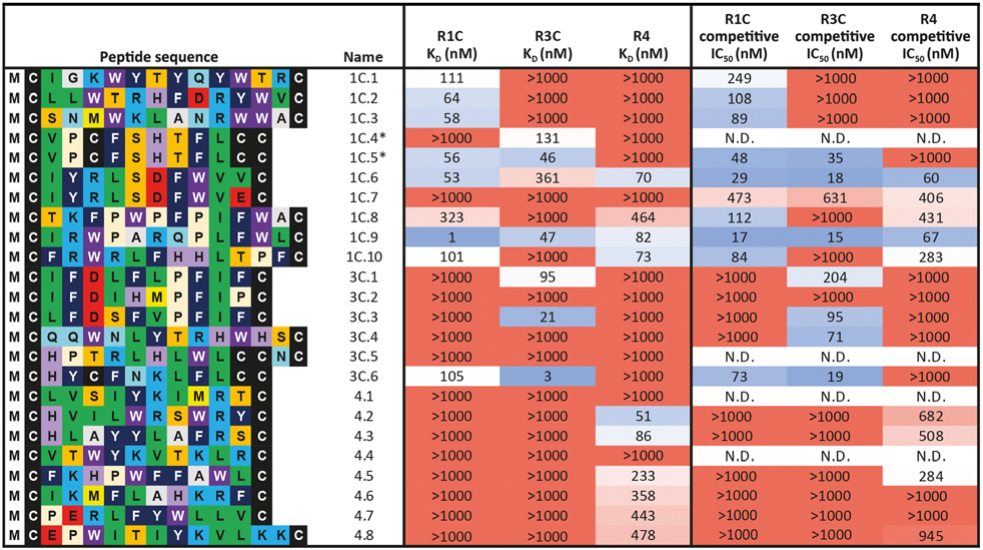

Overall, the purified CP BLI affinity data confirmed the HT analysis in 86% of cases (binders, *K*_D_ < 1000 nM and non-binders, *K*_D_ > 1000 nM). Several potent and selective binders for individual targets were identified – three for FGF-R1C (1C.1, 1C.2, 1C.3) and FGF-R3C (3C.1, 3C.3, 3C.4), and six for FGF-R4 (4.2, 4.3, 4.5, 4.6, 4.7, 4.8) (Table 1). Furthermore, peptide binders for two receptors (1C.5, 1C.8, 1C.10, 3C.6), and for all three receptors (1C.6 and 1C.9) were identified, potentially suggesting conserved binding sites on the receptors. The success rate of our multiple concentration assays (peptide sequences showing <1000 nM affinity for at least one receptor) was 75%. Each of these peptides carries the potential to unravel specific questions around FGF-Rs, or FGF-Rs in complex with their co-receptor, which require ligands with defined selectivity. Four peptides did not show any binding against FGF-Rs. Interestingly, the most enriched binder from the FGF-R3C selection (3C.2, see Supporting Dataset – next generation sequencing) was among these, whereas the other family members (3C.1 and 3C.3) do bind the receptor as expected. This highlights that the level of enrichment does not necessarily correlate with target binding affinity (as demonstrated previously), and that the clustering information is valuable in prioritisation of peptides.^4^

In general, good correlation was observed between the BLI and AlphaScreen data, indicating that the CPs selected against the ECD competitively displace their natural FGF ligands. An interesting case is 3C.4, the most prominent member of a clearly distinct family in the FGF-R3C selection. We could not detect any binding in the BLI experiments, yet the peptide efficiently competes with FGF21 on the ectodomain FGF-Rs. Similar properties were also observed by 1C.7. This may be caused in part by the different positioning of target protein when immobilised on different surfaces that occludes the peptide binding site. This case emphasises the importance of running orthogonal assays for hit prioritisation.

### Use of trimer-18 libraries to improve hit diversity

When comparing the selection results for the different FGF-R targets, the FGF-R4 CP hits were lower affinity binders (*K*_D_ > 100 nM) and only FGF-R4 selective. We reasoned that a clustering pattern with peptides showing a greater degree of sequence similarity could give a larger chance of both finding more potent binders, and identifying sequences which exhibited binding to multiple targets. To this end we constructed two model libraries using trinucleotide phosphoramidites, which enable a greater peptide diversity by eliminating codon redundancy. Unlike the NNK library, the Trimer-18 library (T18) contains an equal mixture of codons from 18 different amino acids.^15^ Stop codons, as well as those for methionine and cysteine were omitted in the library in order to prevent sequence truncations, methionine oxidation, and disulfide bond isomers, respectively. Random codons of 10, 11, and 12 mer length were used in the libraries as these sequences were the most enriched in the previous NNK selections. These sequence lengths were mixed either in a (T18_1), similar to our previous NNK library, or 1 : 18 : 324 (T18_2) 10 : 11 : 12 mer for sampling theoretical diversity. Selection against FGF-R4 ECD was carried out, with comparable enrichment achieved in just 4 rounds for both libraries (Fig. S11, ESI†). NGS and subsequent analysis for both selections revealed an exemplary clustering and sub-clustering pattern similar to that of the previous FGF-R1C selection (Fig. S12, ESI†). The 1 : 1 : 1 library yielded a larger array of subclusters than the 1 : 18 : 324 library (46 and 13 respectively) indicating that more variation in sequence length may contribute to greater cluster and hit diversity.

Representative peptides from each subcluster from both selections (80 and 85 total from the 1 : 1 : 1 library and 1 : 18 : 324 library respectively) were synthesised and affinity measured against all three FGF-Rs following the same workflow as before (Table 2). In some cases, an additional short spacer followed by acidic residues (GSGSEE) was inserted in peptides with a calculated net charge of 0 at pH7 (4.9 and 4.10) in order to improve solubility in cyclisation conditions (pH7.4). The majority of the equal ratio library (70%) and the unequal ratio library (65%) sequences showed binding (*K*_D_ < 1000 nM) to FGF-R4 (Tables S4 and S5, ESI†). Peptides displaying high affinity and diverse selectivity were resynthesised, purified, and *K*_D_ determined *via* multi-concentration BLI (Fig. S22–S24, ESI†). Again, the majority of the results (75%) from purified peptides were in agreement with the single concentration data and compounds displaying unexpected binding characteristics, such as 4.16 which showed no observable binding to FGF-R4, were infrequent. Overall, 71% of sequences tested showed binding (*K*_D_ < 1000 nM) to FGF-R4. Not only were high affinity binders to FGF-R4 found, but also a number of sequences exhibiting affinity for FGF-R4 and FGF-R3C, as well as all three receptors. In particular, peptide 4.22 showed exemplary sub-nanomolar binding affinity to FGF-R4 from our BLI assay despite occupying the smallest subcluster from T18_2. The results of both the clustering analysis and biochemical data show the value of using trimer-18 libraries over NNK libraries in this case to not only improve sequence diversity amongst selection hits, but also in finding high affinity binders to a variety of binding sites with reduced selection rounds. Further studies involving further comparisons of trimer *vs.* NNK libraries for multiple targets would be needed to validate the generality of this trend.

**Table 2:** Biochemical data of purified peptides from the trimer-18 library mRNA display selections. Sequences were chosen from the top binding hits from single concentration BLI data. *K*_D_ was determined by multi-concentration BLI. *K*_D_ < 1000 nM were considered as binders. Affinity (*K*_D_) graded on a colour scheme from low affinity (red) to high affinity (blue). Peptide names are indicated numerically according to the FGF-R4 target selected against. CPs were considered selective where *K*_D_ for one FGF-R is >100 fold over others. All peptides synthesised with an N-terminal acyl, C-terminal flag tag (GSGSDYKDDDDK-NH_2_) and end-to-end disulfide cyclised (see Table S2, ESI for complete list of identities). Peptides 4.9 to 4.17 originated from T18_1 selection and 4.18 to 4.25 from T18_2 selection. Peptides 4.9 and 4.10 synthesised with an additional GSGSEE sequence to lower the isoelectic point

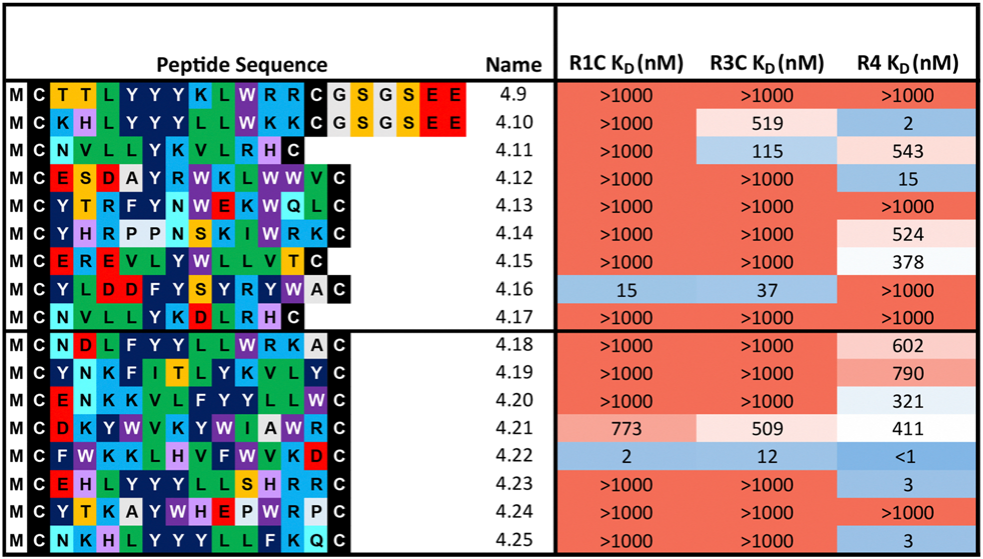

### Selectivity engineering through SAR

**Fig. 2:**
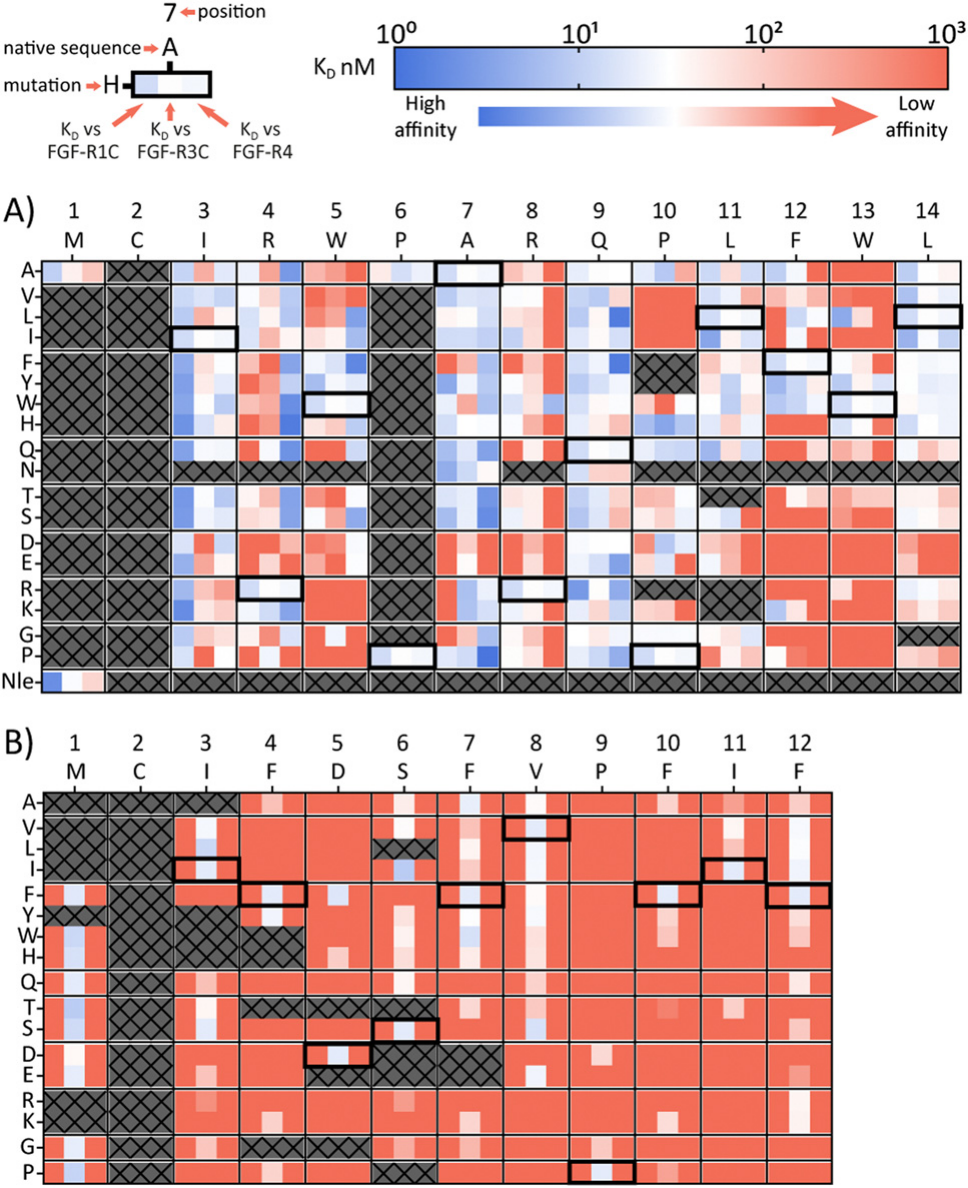
Full amino acid scan heat maps showing *K*_D_ (nM) for (A) pan-FGF-R binding peptide 1C.9 and (B) FGF-R3C selective peptide 3C.3. Native sequence and position shown along top of heat map, amino acid changes to the parent sequence mutations shown along the side. For each amino acid, every first column shows *K*_D_ against FGR-R1C, middle column FGF-R3C *K*_D_ and last column FGF-R4 *K*_D_. Affinity displayed on a log scale from high affinity (1 nM, blue) to low affinity (1000 nM, red). Crosses denote amino acid mutations that were not tested or where synthesis failed. Native sequence results highlighted in black box. All sequences tested contained a disulfide bond, N-terminal acetyl and C-terminal cysteine-spacer-flag-amide (CGSGSDYKDDDDK-NH_2_). *K*_D_ affinity obtained through single concentration BLI assays with biotinylated FGF-R ECD with crude peptides.

A second approach to identifying peptides with a desired binding profile is the evolution of hit peptides through SAR analysis. In the first case, we explored the possibility of engineering a promiscuously binding peptide to a selective one. We focussed on peptide 1C.9 as a starting point, which shows potent binding to all three receptors (*K*_D_ = 1 nM, 47 nM, 82 nM for FGF-R1, FGF-R3C and FGF-R4 respectively). Using parallel peptide synthesis, we made a full amino acid scan. In order to keep the number of peptides to 192, Pro6 was only substituted to Ala, and Met and Asn (where deamidation can occur) were omitted. After quality control (mean purity 70%), the peptides were again subjected to high throughput single concentration BLI experiments to generate detailed SAR knowledge. The results (Fig. 2(A)) show distinct binding behaviour of the mutants for different FGF-Rs. Whereas Arg4 residue substitution is detrimental to the binding to FGF-R1C and FGF-R3C, binding to FGF-R4 is retained for most combinations. Conversely, substitution of Arg8 is not possible for FGF-R4, whilst the other two receptors are more tolerant to substitutions at this position. Additionally, a large number of Ile3 mutations preferentially improve affinity for FGF-R1C, without increasing affinity for the other two FGF-Rs. Analysis of the SAR data (Fig. 2(A)) allow the identification of substitutions that are specific for each receptor, specific for two given receptors, and which are tolerated for all three receptors. As such, the dataset is an invaluable resource to tailor made ligands with a specific selectivity to address a given biological problem.

In a final experimental approach, we explored whether it is possible to engineer a selective peptide into a promiscuous one. In order to investigate this, we performed a full amino acid scan of peptide 3C.3, which stems from the largest cluster from the FGF-R3C selection. While some positions clearly change the binding behaviour to the original target FGF-R3C, none of the mutant peptides bound to either FGF-R1C or FGF-R4 (Fig. 2(B)), suggesting that 3C.3 binds to a unique binding site on FGF-R3C. Therefore, even if a binder is known, using SAR to tailor ligand selectivity must be carried out with caution. While SAR-directed peptide engineering can be valuable, as in the case for 1C.9, in our view the additional time and resource required means choosing a variety of peptides from the mRNA display clustering from the initial screening with the desired binding profile is likely more efficient.

### mRNA display cross screens and statistical evaluation to predict biochemical outcomes

**Fig. 3:**
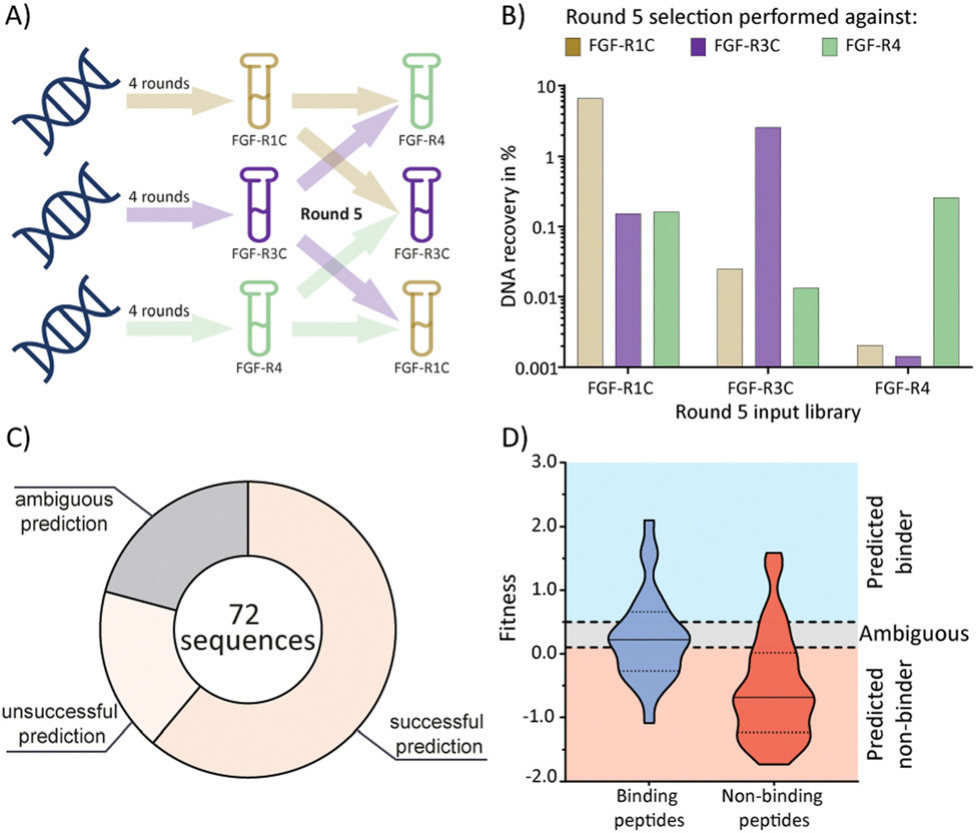
mRNA display cross screen selections and selectivity prediction results. (A) schematic representation of cross screen selections: 4 rounds of mRNA display performed against a single FGF-R target, followed by an additional 5th round against the other two targets in parallel. (B) percentage DNA recovery comparison from the round 5 positive selection for each library against each cross screen target. DNA recovery calculated by qPCR and given as a percentage of the calculated input library. Input library named by the FGF-R target selected against in the previous 4 rounds and the recovery given for round 5 positive selection against FGF-R1C (gold, left column), FGF-R3C (purple, middle column), and FGF-R4 (green, right column). (C) pie chart showing agreement between the fitness binding prediction and the observed biochemical *K*_D_ data determined by BLI. Successful predictions are where fitness values equated to biochemical data, unsuccessful predictions are where fitness values did not equate to observed biochemical data, and ambiguous fitness values are shown in grey. Only BLI data from purified peptides from our original NNK selections (Table 1) were included. *K*_D_ < 1 μM was categorised as a binding sequence to the target. Fitness value <0.1 – predicted non binder, fitness value >0.5, predicted binder, 0.1< fitness <0.5 – ambiguous fit. (D) violin plot showing distribution of individual fitness values for binding (*K*_D_ < 1 μM, dark blue) and non-binding (*K*_D_ > 1 μM, red) peptide sequences. Region of fitness predicting binding to the receptor (fitness >0.5) highlighted in light blue, region of fitness not predicting receptor binding highlighted in orange. Binding peptides that lie within the light blue region, and non-binding peptides lying within the orange region are successfully predicted by the fitness values.

Having established the possibility of identifying selective ligands by traditional methods, we strove to investigate whether there is a more efficient route to the same goal. We turned our attention to the selection process that, together with the analysis by NGS, could yield a wealth of unused information. To investigate this, we used cDNA pools from the round 4 of all original selections, and subsequently performed cross screens (an additional round of mRNA display), using the ECD of the respective other receptors as a target (Fig. 3(A)). Cross screen percentage DNA recovery was lower than for the original round 5 data and lowest for the FGF-R4 library (Fig. 3(B)). This was in line with expectations as the peptide biochemical data from the original FGF-R4 selection show little promiscuity in binding (Table 1). After NGS analysis we calculated fitness values for each of the 24 original peptides investigated above based on the relative enrichment of each peptide sequence during mRNA display (see ESI† for details on the fitness calculation).^4^ We rationalized that a positive value for fitness (>0.5) would indicate binding to the other receptors in the cross screen, whereas a negative value (<0.1) would indicate selectivity for the original receptor (fitness 0.1 to 0.5 was defined as an ambiguous grey zone).

As shown in Fig. 3(C) and (D), the cross screening results were generally in line with the biochemical data, with fitness correctly predicting binding characteristics in 61% of cases (see Fig. S26, ESI† for full list of individual results). There are, however, a number of exceptions, notably the promiscuous peptide 1C.9, which shows potent binding to all receptors in the biochemical assays, but clearly negative fitness values in the NGS data. This observation might stem from the competition of several peptide families for the same binding site. If a better binding peptide family outcompetes the promiscuous peptide, the fitness value would be negative, although the peptide family are binders for the given receptor. In conclusion, the analysis of the NGS data from cross screens can guide the selection of selective peptides. However, the additional workload for the cross screens (total for all 3 targets = 6 additional rounds of mRNA display after 4 rounds of traditional selection, including NGS analysis) does not in our opinion warrant the benefit, as the hits still will need to be followed up by synthesis and biochemical assays, as per the workflow without cross screens. Furthermore, the cluster analysis already guides the choice of peptides for follow up. As this comes without the need for extra experiments this should be the preferred choice.

### SAR prediction using NGS data

**Fig. 4:**
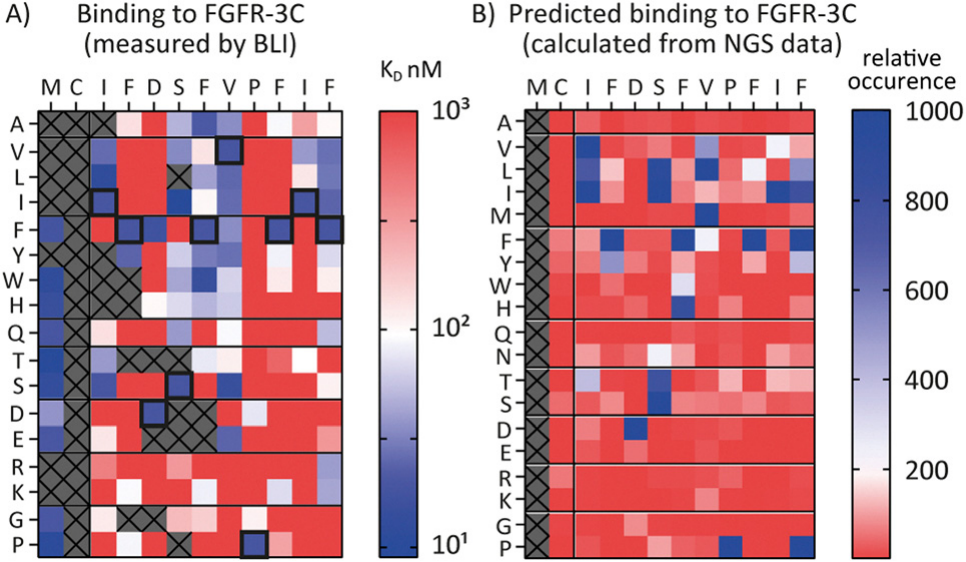
Comparison of biochemical and NGS based SAR analysis of FGF-R3C selective peptide 3C.3. The native sequence is shown along the top, amino acid mutations shown along the side. (A) Full amino acid scan heat maps showing peptide affinity (*K*_D_, nM) against FGF-R3C only. Data obtained from single concentration BLI assays using crude peptides synthesised with an N-terminal acyl and C-terminal cysteine-spacer-flag-amide (CGSGSDYKDDDDK-NH_2_). Affinity displayed on a log scale from high affinity (10 nM, blue) to low affinity (1000 nM, red). Crosses denote amino acid mutations that were not tested or where synthesis failed. Native sequence results highlighted in black box. (B) Heat map showing predicted relative binding affinity of each amino acid mutation as determined from the NGS data of all peptides present within the same cluster as 3C.3 (3879 sequences total). A low relative occurrence of an individual amino acid (red) was taken to predict poor affinity, a high relative occurrence (blue) was taken to predict high affinity.

Encouraged by the possibility to use fitness values from NGS data to determine selectivity (Fig. 3) and the wealth of information provided by the full amino acid scans (Fig. 2), we asked a final question, namely whether it is possible to use NGS data to predict the SAR of a compound. To investigate this, we attempted to use the available round five NGS data to predict the 3C.3 SAR biochemical data previously obtained. Heatmaps were computed from all round five FGF-R3C selection sequences occupying the 3C.3 cluster (Fig. 4, in total 3284 sequences – see ESI† for details). At each residue position, the occurrence of each amino acid was calculated relative to the fitness of the sequence it is found in. A high relative occurrence (>200) was used to predict higher affinity binding to FGF-R3C, since the amino acid at this position was enriched more within the cluster, whereas a low relative occurrence (<200) was used to predict a lower affinity due to lower enrichment.

When comparing the NGS statistical analysis with the biochemical data (Fig. 4), it is apparent that general trends can be reproduced. For example, both representations clearly indicate that aliphatic amino acids (Val, Leu, Ile) are tolerated in position 3, whereas the Pro in position 9 is invariable. A closer look at the data, however, show that the BLI data are far more nuanced, whereas the NGS derived heatmap gives a more coarse grain picture. Importantly, enrichment in mRNA display is relative, meaning that low binding substitutions will be outcompeted by higher affinity binders, whereas the biochemical data show absolute binding values in a situation where the peptides are not in competition with each other. Furthermore, the NGS dataset can also be misleading, as it indicates that Pro is tolerated at position 12, whereas the binding data suggest that this is not the case. Interestingly, a C-terminal Pro was identified as false positive signal in the case of 3C.2 and 4.22 (Tables 1 and 2 respectively) and this analysis may offer an explanation for these observed characteristics. NGS derived SAR data can thus be valuable as a head start for SAR analyses or for the quick design of second-generation libraries, but cannot replace biochemical data on synthesized peptides if a detailed, exact SAR understanding is desired.

## Conclusions

In conclusion, we have shown that potent and selective ligands for three related FGF-receptors, as well as promiscuous binders can be identified through mRNA display, which could serve as valuable tools to decipher the complex FGF biology. We also demonstrated that selectivity can be achieved through a variety of routes including both experimental and bioinformatic data analysis methods. Experimentally, this was accomplished by independent selection campaigns against individual receptors, or by SAR evaluation of a promiscuous peptide binder. Furthermore, use of a ‘higher-quality’ DNA library constructed using trinucleotide phosphoramidites, rather than traditional NNK synthesis, was shown to improve hit diversity, making this an ideal toolkit amongst ‘difficult’ selection targets. However, in our hands, it was not possible to render a selective ligand promiscuous through analysis of SAR, likely indicating binding to a unique site on the receptor for the selected hit peptide. Finally, we were successful in predicting selectivity by computing fitness values for cross screening selections against multiple receptors and generating SAR information solely based on NGS data from the original selections for particular sequence clusters. This bioinformatic approach unravelled a valuable, albeit rougher resolution of selectivity and cluster SAR, and therefore is useful but would struggle to identify lead compounds. Overall, when considering statistical data analysis of selectivity, the full workload needed to generate the necessary datasets must be considered. Therefore, simple selection procedures coupled to powerful sequence clustering methods may be favoured for the choice of sequences intended for detailed biochemical characterisation.

## Author contributions

T. P. S. investigation, data curation, formal analysis, writing – original draft, writing – review and editing, B. B. investigation, data curation, formal analysis, writing – original draft, D. G. methodology, software, C. S. K. software, data curation, B. A. investigation, K. W. D. investigation, Q. R. investigation, L. H. investigation, Q. X. investigation, Y. Y. investigation, P. J. investigation, C. Q. investigation, M. M. conceptualisation, funding acquisition, data curation, visualisation, methodology, supervision, writing – original draft, writing – review and editing, A. K. conceptualisation, methodology, supervision, funding acquisition, writing – review and editing. All authors have reviewed the final draft.

## Conflicts of interest

All authors except T. P. S., B. B., and A. K. are or were employees and shareholders of Novo Nordisk A/S. A. K. was a consultant to Novo Nordisk A/S.

## Supplementary Material

CB-005-D3CB00168G-s001

CB-005-D3CB00168G-s002

CB-005-D3CB00168G-s003

CB-005-D3CB00168G-s004

CB-005-D3CB00168G-s005
