# Supplementary material for: Identification and engineering of potent cyclic peptides with selective or promiscuous binding through biochemical profiling and bioinformatic data analysis
Source: RSC Chem Biol. 2023 Nov 14;5(1):12–8. doi: 10.1039/d3cb00168g (PMC10763615; doi:10.1039/d3cb00168g)
Supplement: CB-005-D3CB00168G-s001 [file CB-005-D3CB00168G-s001.pdf]

Electronic Supplementary Material (ESI) for RSC Chemical Biology.
This journal is © The Royal Society of Chemistry 2023

Supplementary Information

Identification and engineering of potent cyclic peptides with selective or promiscuous binding through biochemical profiling and bioinformatic data analysis

Thomas P. Smith[a,†], Bhaskar Bhushan[b,†], Daniele Granata[c], Christian S. Kaas[d], Brigitte Andersen[e], Klaas W. Decoene[d], Qiansheng Ren[f], Haimo Liu[f], Xinping Qu[f], Yang Yang[f], Jia Pan[f], Quijia Chen[f], Martin Münzel[d,‡], Akane Kawamura[a,b,‡]

[a] Chemistry - School of Natural and Environmental Sciences, Newcastle University, Newcastle upon Tyne, NE1 7RU, UK

[b] Chemistry Research Laboratory, Department of Chemistry, University of Oxford, OX1 3TA, UK

[c] Digital Science and Innovation, Novo Nordisk A/S, Novo Nordisk Park, 2760 Måløv, Denmark

[d] Global Research Technologies, Novo Nordisk A/S, Novo Nordisk Park, 2760 Måløv, Denmark

[e] Global Drug Discovery, Novo Nordisk A/S, Novo Nordisk Park, 2760 Måløv, Denmark

[f] Novo Nordisk Research Center China, Novo Nordisk A/S, Shengmingyuan West Ring Rd, Changping District, Beijing, China

†These authors contributed equally

‡Corresponding authors:

Akane Kawamura, E-mail: akane.kawamura@ncl.ac.uk

Martin Münzel, E-mail: myzm@novonordisk.com

Table of Contents

Protein Expression and Purification of hFGFR ECDs 1
In vitro selection of cyclic peptide libraries against FGF-R ECD targets 7
Next generation sequencing and clustering analysis 10
Chemical synthesis of selected macrocyclic peptides 10
BLI binding measurements 19
NNK single concentration BLI initial screen 21
NNK purified peptide BLI binding data 22
Trimer-18 single concentration BLI initial screen 25
Trimer-18 Purified peptide BLI binding data 27
Alphascreen binding assay 31
Statistical analysis of peptide fitness for affinity prediction 32
Supporting datasets 33
References 34

Protein Expression and Purification of hFGFR ECDs

Materials: EndoFree Plasmid Maxi Kit was purchased from QIAGEN. Optimum Growth™ Flasks were purchased from THOMSON. FreeStyle™ 293 Expression Medium, Geneticin™ Selective Antibiotic, Pluronic™ F-68 Non-ionic Surfactant (100 X), 293fectin™ Transfection Reagent, Opti-MEM™ Reduced Serum Medium were purchased from Invitrogen. Stericup Quick Release-HV Sterile Vacuum Filtration System was purchased from Merck. NuPAGE™ 4 to 12%, Bis-Tris, 1.0–1.5 mm, Mini Protein Gels was purchased from Invitrogen. Superdex® 75 was purchased from GE Healthcare. SEC- HPLC was performed on TSKgel G2000 SWxl column coupled with Agilent HPLC. LC-MS was performed on Agilent PLRP-S column coupled with Waters UPLC Xevo G2 Q-TOF. Kinetic Turbidimetric LAL Test kit was purchased from Charles River Laboratories.

DNA sequences of FGFRs cloned for expression:

FGF-R1C-ECD: AGGCCCTCCCCCACACTGCCCGAACAAGCTCAACCTTGGGGAGCTCCCGTGGAGGTGGAAAGCTTT
CTGGTGCACCCCGGCGATTTACTGCAGCTGAGATGTCGTCTGAGAGACGACGTCCAGAGCATCAACTGGCTCAGAG
ACGGCGTGCAGCTGGCCGAATCCAATCGTACCAGAATCACCGGCGAGGAGGTCGAGGTCCAAGATAGCGTGCCCG
CTGATTCCGGTTTATATGCTTGCGTGACCAGCTCCCCTAGCGGCAGCGATACCACCTACTTCAGCGTGAATGTCTCC
GACGCTTTACCCAGCAGCGAGGATGACGACGACGATGACGACTCCTCCTCCGAGGAGAAGGAGACAGATAACACC
AAGCCCAATCGTATGCCCGTGGCCCCCTACTGGACATCCCCCGAGAAGATGGAGAAGAAACTCCATGCCGTGCCCG
CTGCTAAGACCGTGAAATTCAAGTGTCCCAGCTCCGGCACCCCCAATCCTACTTTAAGGTGGCTCAAGAATGGCAAG
GAGTTCAAGCCCGACCATCGTATCGGCGGCTATAAGGTGAGGTACGCCACTTGGAGCATCATCATGGACTCCGTCG
TGCCCAGCGACAAGGGCAATTACACTTGTATCGTGGAGAACGAGTACGGAAGCATCAACCACACCTACCAGCTGGA
TGTGGTGGAGAGGAGCCCCCATCGTCCTATTCTGCAAGCTGGACTGCCCGCTAACAAGACCGTCGCTTTAGGCAGC
AACGTGGAGTTCATGTGCAAGGTCTACTCCGATCCCCAGCCCCACATTCAGTGGCTGAAGCACATTGAGGTGAATG
GCAGCAAGATTGGCCCCGACAATTTACCCTACGTGCAGATTTTAAAGACCGCCGGCGTCAACACCACCGACAAGGA
GATGGAGGTGCTGCACCTCAGAAACGTGAGCTTCGAGGACGCCGGCGAATATACTTGTCTGGCCGGCAACAGCAT
CGGACTGAGCCACCACAGCGCTTGGCTGACAGTTTTAGAGGCTCTGGAAGAGAGGCCCGCCGTGATGACCTCCCCT
CTGTATTTAGAG

FGF-R3C-ECD: GAGTCTTTAGGCACCGAACAAAGGGTCGTGGGCAGAGCTGCCGAAGTGCCCGGTCCCGAACCCG
GACAGCAAGAACAGCTGGTGTTCGGAAGCGGCGACGCTGTCGAGCTCAGCTGTCCTCCTCCCGGTGGCGGACCTAT
GGGCCCCACAGTGTGGGTGAAGGACGGCACCGGTTTAGTCCCTAGCGAGAGAGTGCTGGTCGGCCCTCAGAGGCT
GCAAGTTCTGAACGCCTCCCACGAGGATTCCGGCGCCTACTCTTGTAGACAGAGACTCACCCAGAGGGTGCTGTGC
CACTTCAGCGTGAGGGTGACAGATGCCCCCAGCTCCGGCGATGACGAGGATGGAGAGGATGAGGCCGAGGACAC
TGGTGTGGATACCGGCGCTCCCTACTGGACAAGGCCCGAGAGGATGGATAAGAAATTATTAGCCGTGCCCGCCGCT
AACACCGTGAGATTTCGTTGTCCCGCTGCCGGAAACCCCACCCCTAGCATTAGCTGGCTGAAGAACGGTCGTGAGT
TTAGAGGCGAGCACAGAATCGGCGGCATCAAGCTGAGGCACCAGCAGTGGTCTTTAGTGATGGAGAGCGTGGTGC

CCAGCGACAGAGGAAACTACACTTGTGTCGTGGAGAACAAGTTCGGCAGCATTCGTCAGACCTACACTTTAGACGT
TTTAGAGAGATCCCCCCACAGACCCATTTTACAAGCTGGACTGCCCGCTAATCAGACAGCCGTGCTGGGCTCCGATG
TCGAGTTCCACTGCAAGGTGTACTCCGACGCCCAGCCCCACATCCAGTGGCTGAAGCACGTGGAGGTGAATGGCAG
CAAAGTGGGACCCGATGGCACCCCTTACGTGACCGTGCTGAAGACCGCCGGCGCTAACACCACCGATAAGGAGCT
GGAGGTGCTGTCTTTACACAACGTGACCTTCGAGGATGCCGGCGAGTACACTTGTCTCGCCGGCAATTCCATCGGCT
TTTCCCACCACAGCGCTTGGCTGGTGGTGCTGCCCGCTGAGGAAGAACTGGTGGAAGCTGACGAGGCCGGAAGCG
TGTATGCCGGT

FGF-R4-ECD: CTGGAGGCCAGCGAGGAGGTGGAACTGGAGCCTTGTTTAGCTCCTTCTTTAGAGCAGCAAGAACAA
GAACTGACAGTGGCCCTCGGACAGCCCGTGAGACTGTGCTGTGGTCGTGCTGAGAGGGGAGGCCACTGGTACAAG
GAGGGCTCCAGACTGGCCCCCGCTGGAAGAGTGAGAGGCTGGAGAGGAAGGCTGGAGATCGCCAGCTTCCTCCCC
GAAGACGCCGGTCGTTATTTATGTCTGGCTCGTGGCTCCATGATCGTGCTGCAGAATTTAACACTGATCACCGGCGA
CTCTTTAACCAGCAGCAACGACGATGAAGATCCTAAGTCCCATAGAGACCCCAGCAATCGTCACAGCTATCCTCAGC
AAGCTCCTTACTGGACCCACCCCCAGAGGATGGAGAAGAAGCTGCATGCCGTGCCCGCCGGCAACACAGTGAAGTT
TCGTTGTCCCGCTGCCGGAAACCCCACCCCCACCATTCGTTGGCTGAAGGACGGCCAAGCTTTCCACGGCGAGAATC
GTATCGGCGGCATTCGTCTGAGGCACCAGCACTGGTCTTTAGTGATGGAGAGCGTCGTGCCCTCCGATCGTGGCAC
CTATACATGTTTAGTGGAGAACGCCGTGGGAAGCATTCGTTACAATTATTTACTCGACGTGCTGGAAAGGTCCCCCC
ACAGACCTATTTTACAAGCTGGACTGCCCGCTAACACAACCGCCGTGGTGGGCTCCGACGTGGAGCTGCTGTGTAA
GGTGTACAGCGATGCCCAGCCTCACATCCAGTGGCTGAAGCACATCGTGATCAACGGCTCCAGCTTTGGCGCCGAC
GGCTTCCCCTATGTCCAAGTTCTGAAAACCGCCGACATTAACAGCAGCGAGGTGGAGGTGCTGTATCTGAGAAACG
TGTCCGCCGAGGATGCTGGCGAGTACACTTGTCTCGCCGGAAACAGCATCGGCCTCTCCTACCAGTCCGCTTGGCTG
ACCGTTTTACCCGAAGAAGATCCTACATGGACAGCCGCCGCTCCCGAGGCTAGATACACCGAT

Preparation of plasmids: Three different DNA constructs for the extracellular domain (ECD) of human FGF-R1C (uniprotKB P11362), FGF-R3C (uniprotKB P22607), and FGF-R4 (uniprotKB 22455) were generated and cloned into the expression vector pJSV002 using seamless assembly cloning technology (Taihe Biotechnology Co.,LTD)(Figure S 1). Sequences also contained C-terminal Avi and HPC4 sequences. The expression vectors were then transformed separately into DH5α *Escherichia coli* (*E. coli*), plated onto a LB plate containing ampicillin (100 μg/mL), and subsequently incubated overnight at 37 °C. The resulting culture medium of transformed cells was then used to obtain plasmid DNA, which was sequenced to confirm the proper reading frame of constructs. For mammalian cell transfection, plasmid preparation kits (EndoFree Plasmid Maxi Kit) were used to purify endotoxin-free plasmids from large-volume *E. coli* cultures.

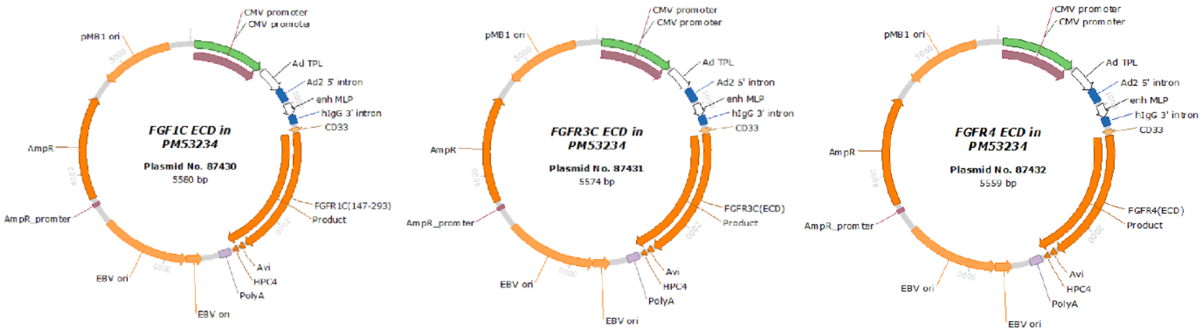

Figure S 1 Plasmids prepared for recombinant FGFR ECD protein expression

Expression of recombinant FGF-Rs: Plasmids of FGF-Rs were transiently transfected in suspension-growing mammalian cells using a FreeStyle 293 Expression Medium (Gibco). Briefly, HEK 293-6E cell suspension culture was maintained in FreeStyle 293 expression medium with 1% F-68 and 25 µg/mL Geneticin (Gibco). A shake flask with a vented cap was used to produce the culture at 37 °C in a humidified atmosphere containing 5% CO_2 with 50 mm orbital shaking (135 rpm/min). For transient transfection of recombinant protein, the cells were split on at density of 0.7~1 × 10^6 cells/mL the day before transfection and allowed to grow overnight. Cells were then diluted to a final volume of 560 mL with the density of 1.07×10^6 viable cells/mL. Transfection was carried out by: 1) 600 µg expression plasmid(s) and Opti-MEM were gently mixed in a final volume of 20 mL; 2) diluted DNA was then added to the diluted 293fectin (600 µL 293fectin in 20 mL Opti-MEM, incubated at room temperature for 5 min) and mixed followed by 20 minutes incubation at room temperature for 20 minutes; 3) DNA/293fectin mixture was added drop wise to 560 mL cells in a 1.6-L Optimum Growth™ flask and incubated at 37 °C on an orbital shaker with a humidified atmosphere of 5% CO_2. After 5 days, cell culture medium was harvested by centrifugation for 30 minutes at 6000 rpm and then filtered with a 0.45 µm membrane. Protein expression level was then determined by SDS-PAGE.

Purification of recombinant FGFRs: All the purification steps were performed at 4 °C. The cell culture was loaded onto an anti-HPC4 column (prepared in house) and washed with washing buffer (20 mM Tris-HCl, 100 mM NaCl, 1 mM $CaCl_2$, pH 7.5), and then eluted with elution buffer (20 mM Tris-HCl, 100 mM NaCl, 1 mM EDTA, pH 7.5). Eluted fractions were biotinylated using BirA biotin ligase (in house produced). Finally, the biotinylated protein was purified using gel filtration with Superdex® 75 (PBS, pH 7.4). The eluted filtrate fraction was then concentrated using Amicon Ultra concentrator (Millipore) with a 10 kDa cut-off filter. Protein purity and integrity was analysed by SDS-PAGE and SEC-HPLC (TSKgel G2000 SWxl column on Agilent HPLC, 1x PBS pH 7.4) respectively (Supplementary Figure S2-10). LC-MS (Agilent PLRP-S column on Waters UPLC Xevo G2 Q-TOF) was used to confirm the protein mass, and the endotoxin was determined using Kinetic Turbidimetric LAL Test kit.

FGF-R1C analytical data:

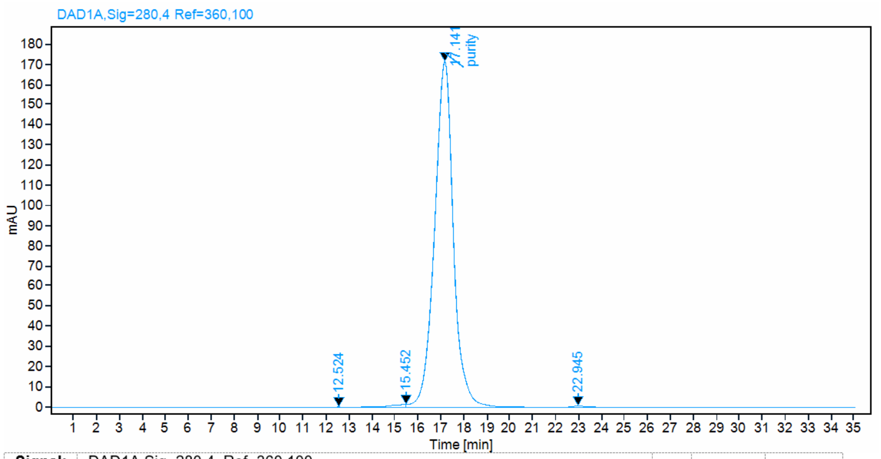

Signal:	DAD1A,Sig=280,4 Ref=360,100						
	RT [min]	Type	Width [min]	Area	Height	Area%	Name
	12.524	BB	0.92	3.63	0.12	0.04	
	15.452	BV F	2.37	70.01	1.32	0.71	
	17.141	VB	5.73	9791.96	171.63	99.02	purity
	22.945	BB	1.65	22.82	0.63	0.23	

Figure S 2 SEC-HPLC chromatogram of purified FGF-R1C ECD. Column: TSKgel G2000 SWxl

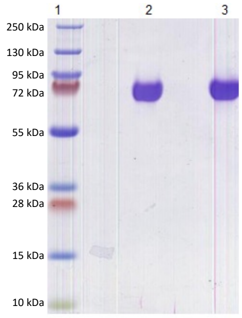

Figure S 3 SDS-PAGE of purified biotinylated FGF-R1C ECD. Lane 1: Molecular weight marker. Lane 2: FGF-R1C ECD (reducing conditions), Lane 3: FGF-R1C ECD (oxidising conditions)

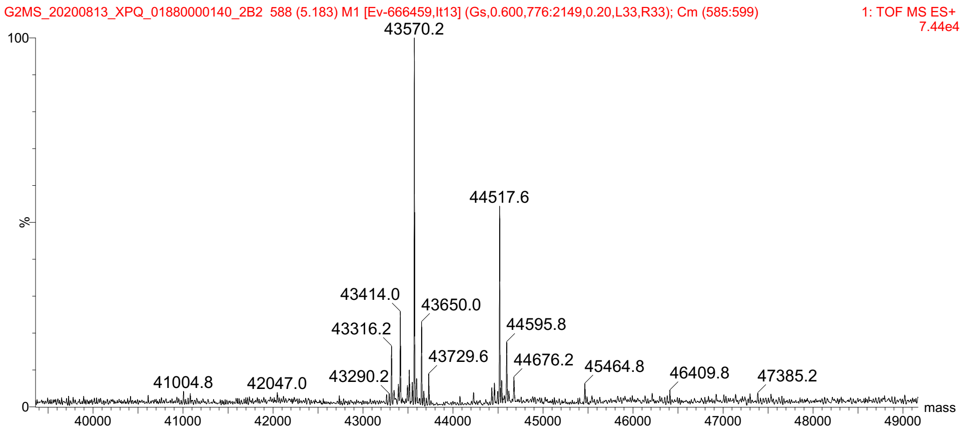

Figure S 4 LC-MS spectrum of purified biotinylated FGF-R1C ECD. Theoretical m/z biotinylated FGF-R1C ECD: 43570.3 Da, observed m/z: 43570.2. Theoretical m/z biotinylated FGF-R1C ECD + O-Glycan modification: 44517.3, observed m/z: 44517.6.

FGF-R3C analytical data:

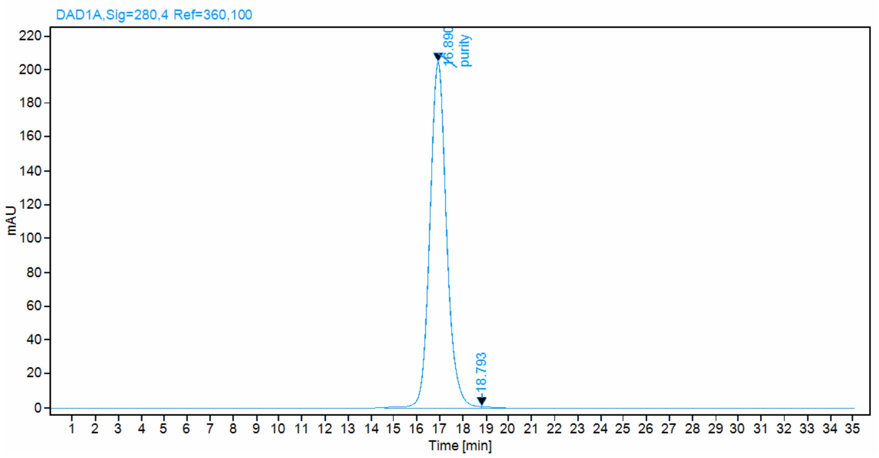

Signal:	DAD1A,Sig=280,4 Ref=360,100						
	RT [min]	Type	Width [min]	Area	Height	Area%	Name
	16.89	VV B	4.24	10381.24	204.68	99.64	purity
	18.793	VB B	2.59	37.39	0.96	0.36	

Figure S 5 SEC-HPLC chromatogram of purified FGF-R3C ECD. Column: TSKgel G2000 SWxl.

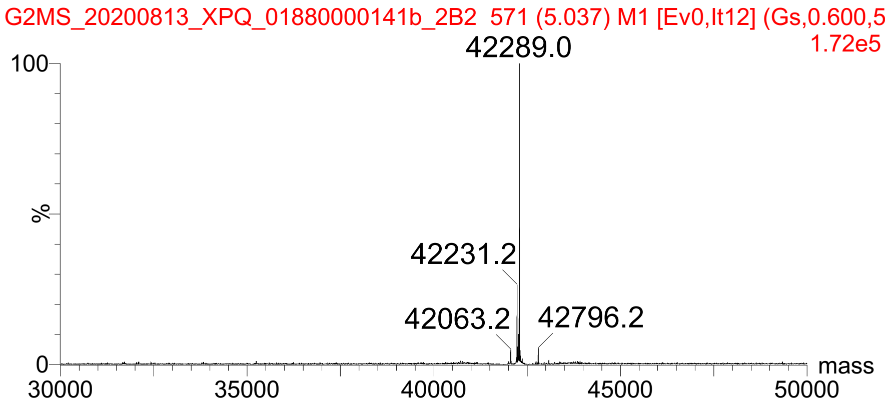

Figure S 6 LC-MS spectrum of purified biotinylated FGF-R3C ECD. Theoretical m/z biotinylated FGF-R3C ECD: 42290.1 Da, observed m/z: 42289.0.

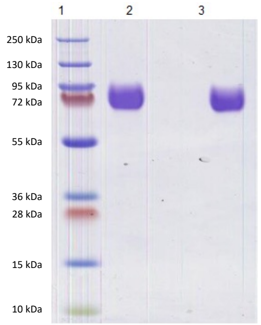

Figure S 7 SDS-PAGE of purified biotinylated FGF-R3C ECD. Lane 1: Molecular weight marker. Lane 2: FGF-R3C ECD (reducing conditions), Lane 3: FGF-R3C ECD (oxidising conditions)

FGF-R4 analytical data:

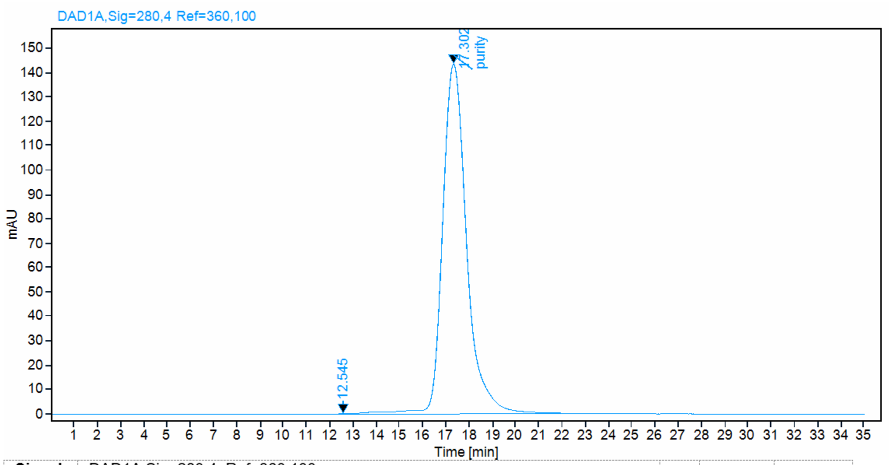

Signal:	DAD1A,Sig=280,4 Ref=360,100						
	RT [min]	Type	Width [min]	Area	Height	Area%	Name
	12.545	BV F	0.8	5.2	0.2	0.05	
	17.302	VB	10.61	10389.38	143.77	99.95	purity

Figure S 8 SEC-HPLC chromatogram of purified FGF-R4 ECD. Column: TSKgel G2000 SWxl.

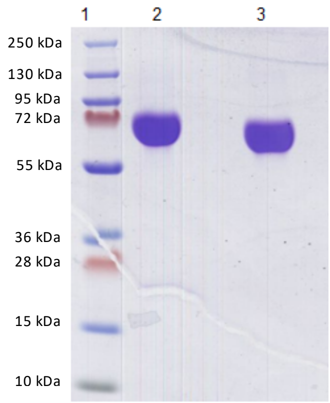

Figure S 9 SDS-PAGE of purified biotinylated FGF-R4 ECD. Lane 1: Molecular weight marker. Lane 2: FGF-R4 ECD (reducing conditions), Lane 3: FGF-R4 ECD (oxidising conditions)

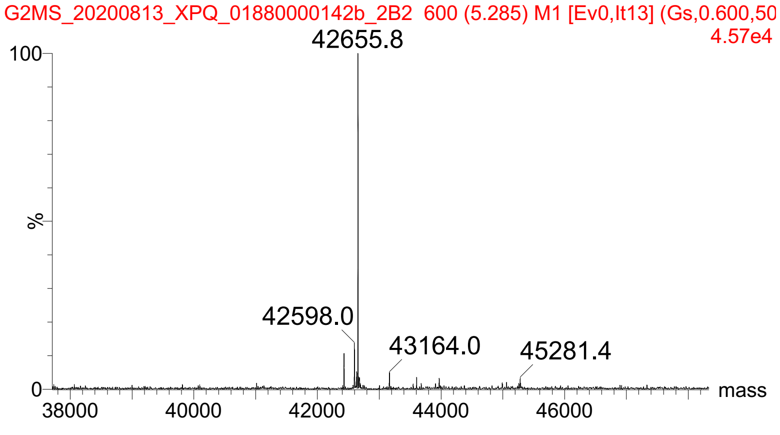

Figure S 10 LC-MS spectrum of purified biotinylated FGF-R4 ECD. Theoretical m/z biotinylated FGF-R4 ECD: 42657.5 Da, observed m/z: 42655.8.

In vitro selection of cyclic peptide libraries against FGF-R ECD targets

Preparation of puromycin-fused mRNA library

RNAs consisting of 4−12 repeated NNK random sequences (DNA: 5′-TAA TAC GAC TCA CTA TAG GGT TAA CTT TAA GAA GGA GAT ATA CAT **ATG TGC** (**NNK**)$_m$ **TGC GGC AGC GGC AGC GGC AGC** TAG GAC GGG GGG CGG AAA-3′, *m* = 4-12) were prepared by *in vitro* transcription according to the reported method.[1] The resulting RNAs were mixed in the following ratio: $(NNK)_4$:$(NNK)_5$:$(NNK)_6$:$(NNK)_7$:$(NNK)_8$:$(NNK)_9$:$(NNK)_{10}$:$(NNK)_{11}$:$(NNK)_{12}$=1:2:4:8:16:32:64:64:64. The mRNA library was ligated with a puromycin linker (5′-CTCCC GCCCC CCGTC C-$(SPC18)_5$-CC-puromycin-3′) by T4 RNA ligase (Thermo Fisher). The ligated product was purified by phenol–chloroform extraction and ethanol precipitation.

Trimer-18 (T18) RNAs were constructed as above using 10-12 repeated random T18 codons purchased from Ella Biotech (DNA: 5′-TAA TAC GAC TCA CTA TAG GGT TAA CTT TAA GAA GGA GAT ATA CAT ATG TGC $(XXX)_n$ TGC GGC AGC GGT TCT GGT AGT TAG GAC GGG GGG CGG AAA-3′, n = 10-12). The RNAs were then mixed in the following ratio: $(XXX)_{10}$:$(XXX)_{11}$:$(XXX)_{12}$=1:1:1 or 1:18:324.

In vitro selection of cyclic peptides binding to FGF-R

Translation of the first selection rounds was performed using 25 pmol mRNA-puromycin in 11.5 µL of translation mixture (PURExpress in vitro protein synthesis kit, NEB) at 37 °C for 30 min in the presence of PURExpress disulfide-bond enhancers (NEB), followed by incubation at 60 °C for 10 min to dissociate ribosomes from the mRNA constructs.

This solution was then diluted up to 18.7 µL with M-MLV reverse transcriptase (Promega, 5.3 U/µL), reverse primer P2 (5′-TGC GGC AGC GGC AGC GGC AGC TAG GAC GGG GGG CGG AAA-3′, 1.8 uM), M-MLV buffer (Promega), dNTPs (0.3 mM), and MilliQ water. The contents were reverse transcribed at 42 °C for 1 h. Following this, the contents were diluted to 40 µL with selection buffer (PBS pH7.4 supplemented with 0.05% Tween-20 and 0.01% BSA). For FGF-R4, yeast total RNA (Roche diagnostics) was added to the diluted reverse-transcribed pool to a final concentration of 1 mg/mL.

Negative selections were performed by incubation of the diluted reverse-transcription solution for 30 min at 4 °C with a suspension of streptavidin-functionalised Dynabeads magnetic beads (M-280, Invitrogen) which were pre-loaded with biotinylated Avi-tag peptide for NNK selections or biocytin (Sigma Aldrich) for T18 selections. Following this, the beads were precipitated using a magnet, the supernatant collected, and transferred to a fresh tube for another round of negative selection. After three rounds of negative selection, the resulting supernatant was then incubated for 30 min at 4 °C with biotinylated FGF-R loaded

magnetic beads. For T18 selections, FGF-R4 was pre-incubated with the supernatant for 30 min at 4 °C before incubation for a further 10 min with magnetic beads.

All bead pellets were washed three times with milliQ water, and the bound cDNA sequences eluted by incubation at 95 °C for 5 min in 100 µL PCR buffer (1x NH4 buffer, 2.5 mM $MgCl_2$, 0.25 mM dNTPs, and 0.5 µM each of reverse primer P2 (5'-TGC GGC AGC GGC AGC GGC AGC TAG GAC GGG GGG CGG AAA-3') and forward primer P1 (5'-TAA TAC GAC TCA CTA TAG GGT TAA CTT TAA GAA GGA GAT ATA CAT ATG TGC-3'). For T18 selections PCR buffer used reverse primer CPrevCys.R39 (5'-TTT CCG CCC CCC GTC CTA ACT ACC AGA ACC GCT GCC GCA-3') and forward primer CPfwdCys.F51 (5'-TAA TAC GAC TCA CTA TAG GGT TAA CTT TAA GAA GGA GAT ATA CAT ATG TGC-3'). Absolute quantities of recovered DNA after each selection step were determined by qPCR (Figure S 11).

After addition of Taq DNA polymerase to the positive selection eluate, the mixture was used for PCR amplification. The amplified DNA was purified by phenol/chloroform extraction followed by ethanol precipitation. The resulting DNA library was transcribed *in vitro*, the resultant RNA purified by phenol/chloroform extraction, and quantified using an Invitrogen™ Qubit™ 4 Fluorometer (Thermo Fisher). The RNA was then ligated with the puromycin linker and used for the next round of selection.

Subsequent selection rounds were performed as described but using 12.5 pmol puromycylated mRNA in 5.5 µL IVTT reactions instead. Dynabead (mass per pmol of puromycylated mRNA library) used for both negative and positive selections for each target was 8-12 µg/pmol. Protein (8-10 pmol) was loaded onto the Dynabeads to a final concentration of 420-500 nM. After the final rounds of the selection, the resulting cDNA libraries were sequenced by next-generation sequencing.

For mRNA display cross screens mRNA display procedure was performed as above. Round 5 (R5) input puromycin-ligated RNA from all three NNK selections were subjected to two R5 selections in parallel against the two receptors not selected for in the previous 4 rounds, i.e. R5 input ligated RNA from FGF-R1C selection was selected against both FGF-R3C and FGF-R4 in parallel.

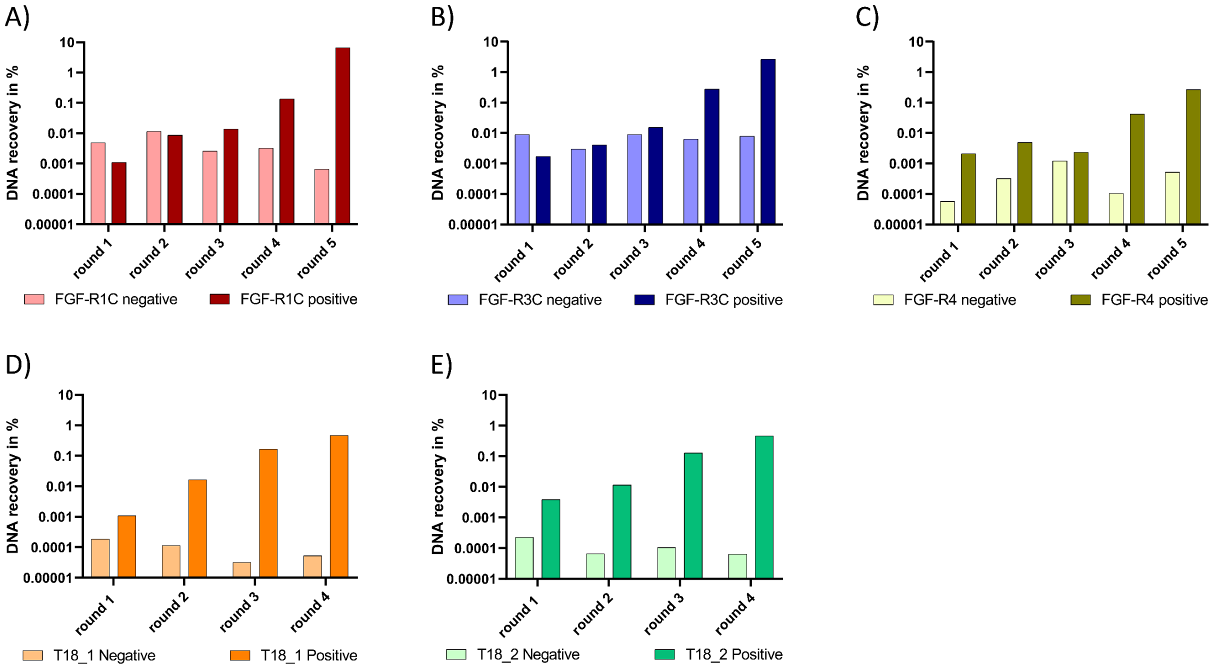

Figure S 11 Enrichment data from individual selections against each FGF-R target using NNK input libraries (1:2:4:8:16:32:64:64:64 4-12mer variable codons) (A: FGF-R1C, B: FGF-R3C, C: FGF-R4) and against FGF-R4 using two different Trimer-18 input libraries. T18_1: 1:1:1 10-12mer variable codons, T18_2: 1:18:324 10-12mer variable codons libraries (D-E). % DNA recovery as determined by qPCR shown for each round of selection against biotinylated FGF-R ECD. Negative data obtained after 3 selections against beads immobilised with biotinylated avitag peptide (A-C) or biocytin (D-E), positive data obtained after single selection against beads immobilised with target receptor.

Next generation sequencing and clustering analysis

For Illumina MiSeq sequencing, the DNA output of each selection round was amplified in two PCR steps to append the required barcodes and constant regions. The PCR amplification was performed using Roche KAPA HiFi HotStart Library Amp Kit following the manufacturer's protocol. The PCR products were then gel purified, quantified using Qubit Broad range dsDNA assay kit (Thermo Fisher) and pooled into libraries of 20 pM. The libraries also contained 10% PhiX DNA following manufacturers recommendations. Finally, the NGS library was sequenced using MiSeq® Reagent Kit v3 (150 cycles). See already published procedures for further details and clustering procedure (Bhushan, B. et al, Chem Sci 2022).[2]

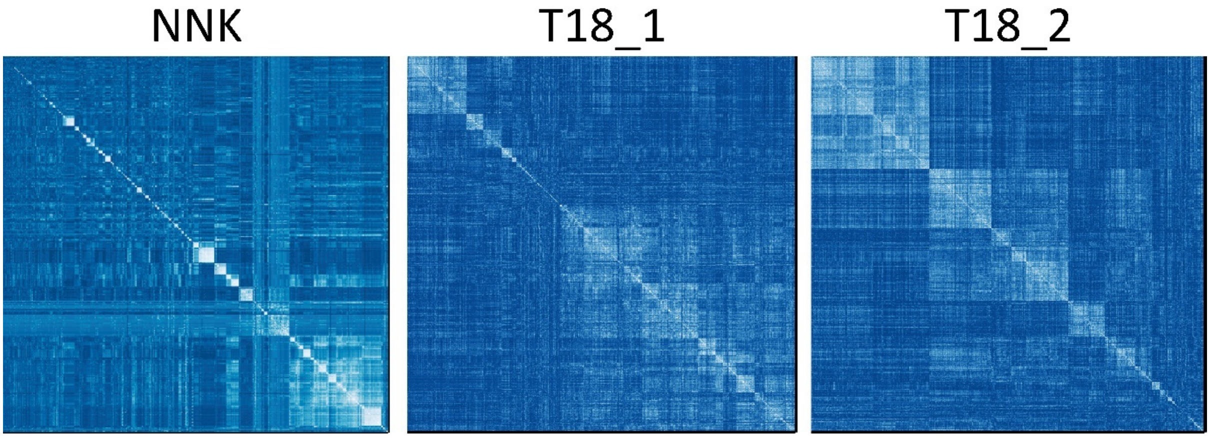

Figure S 12 Comparison of clustering of three different mRNA display libraries against FGF-R4 target. NNK library comprised 4-12mer variable NNK codons (1:2:4:8:16:32:64:64:64), T18_1 comprised 10-12mer variable Trimer-18 codons (1:1:1), T18_2 comprised 10-12mer variable trimer-18 codons (1:18:324). NGS hierarchical clustering diagrams of the top enriched sequences in 5th NNK selection round or 4th T18 selection round. Peptide similarity for each enriched hits are compared individually for each target with low (dark) to high (light) similarity scores shaded as a heat-map: NNK FGF-R4 selection (left), T18_1 FGF-R4 selection (middle), and T18_2 FGFR4 selection (right).

Chemical synthesis of selected macrocyclic peptides

General: All chemicals were of analytical grade or higher. Acetonitrile (MeCN) (LiChrosolve), trifluoroacetic acid S3 (TFA), and diethyl ether were purchased from Merck KGaA (Darmstadt, Germany). Triisopropylsilane (TIPS), N,N´- diisopropylcarbodiimide (DIC), dithiothreitol (DTT), collidine, dimethyl sulfoxide (DMSO) were from Sigma–Aldrich, Chemie GmBH (Steinheim, Germany). Water was obtained from a MilliQ equipment (Advantage A10) from Millipore (Molsheim, France). N-Methyl pyrrolidone (NMP) dimethylformamide (DMF) and piperidine were from Biosolve (Dieuze, France). Standard Fmoc amino acids, resins and coupling reagents, Oxyma Pure were from Novabiochem (Darmstadt, Germany) or Protein Technologies (Tucson, USA). PS resin, and Fmoc-Rink amide PS resin, were all purchased from Merck Millipore (Novabiochem).

Array synthesis of peptides selected for primary screen/ full amino acid scans: Peptides selected for initial high throughput screening, as well as those for mutational scans, were synthesised in a 96-well plate format using Intavis Multipep RSI instruments. The synthesis was performed on a 5 µmol scale using 0.3 mM of

Fmoc-PAL AM resin (Novabiochem) pre-loaded with the sequence: GSGSDYKDDDDK-NH_2. This corresponds to the spacer sequence present in our mRNA display libraries as well as a Flag-tag added for solubility and potential detection in bioassays. Synthesis was performed using standard Fmoc chemistry. Double deprotection (5 + 8 min) was achieved at room temperature using 20% (v/v) piperidine with 0.1 M Oxyma in DMF (200 µL). Double couplings (60 + 120 min) were performed at room temperature with 3 M DIC (9 µL), 3 Mcollidine (9 µL), and 0.1 M amino acid solution containing 0.1 M Oxyma (83 µL), all in DMF. A capping step was added after each amino acid coupling for some peptides using 0.3 M AcOH in DMF/ 0.1 M Oxyma with coupling reagents. Peptides shown in supplementary table S3/4/5.

After completion of the synthesis, peptides were cleaved from the resin using a mixture of trifluoroacetic acid (TFA)/ water/ triisopropylsilane (TIPS)/ dithiothreitol (DTT) (94:2:2:2). Approximately 1.2 mL of the cleavage mixture was added stepwise each well over two hours. After incubation, addition of diethlyether was used to precipitate the peptides and the subsequent mixture of cleavage solution/diethylether was separated from the precipitated peptide using gravity filtration. The peptides were then washed an additional 3 times with 500 µL diethylether and dried using vacuum filtration. Each of the dried peptides were re-dissolved in DMSO (1 mL) and transferred to a master plate (96 deep-well). Concentration in the master plate was mostly in the range 1 – 2 mM. Finally, peptides were diluted to 50 µM in folding buffer (100 mM HEPES pH 7.4, 20% DMSO) to promote disulfide bond formation and peptide macrocyclization. 50 mM ammonium bicarbonate was also used for some 96 well plates instead of 100 mM HEPES.

Otherwise, peptides were purchased from Apigenex, Prague, Czech Republic, or GL-Biochem, Shanghai, China.

Purification of selected peptides: Purification was performed on Gilson LC systems, equipped with a 322 pump module, 155 UV/vis detector and GX-271 automated sample collector. A typical gradient of 20% - 60% solvent B over 30 min (flow rate of 25 mL/min, Axia Gemini 5µM NX-C18 110 Å columns, 250x30 mm) was employed (Solvent A: 0.1% TFA in water, Solvent B: 0.1% TFA in Acetonitrile). Finally, each peptide was isolated by lyophilisation.

Peptide characterisation: 96 well plate peptides were characterised by LC-MS (Waters Acquity system with QDA detector, KDA3351, and TUV detector, F08UPT321M) using Waters Acquity UPLC BEH C18 column (130 Å, 1.7 µm, 2.1 mm x 50 mm). A 3 min linear gradient of 10% to 90% solvent B was run; solvent A: 0.05% TFA in water, solvent B: 0.05% TFA in MeCN. NNK Purified peptide identity was determined either by method 1: Agilent 1220 system with a Phenomenex BioZen Peptide PS-C18 column (3 µm, 150 x 4.6 mm) using a 20 min linear gradient of 2% to 98% solvent B in solvent A (solvent A 0.1% TFA in water, solvent B 0.085% TFA in MeCN); method 2: Waters Acquity system with a PDA detector (MIOUPD33A), and SQ detector (BA699) using Waters Acquity UPLC BEH C18 column (130 Å, 1.7 µm, 2.1 mm x 50 mm) on a 6 min linear gradient of 0% to 80% solvent D in solvent C (solvent C: 10 mM Tris 15 mM$(NH_4)_2SO_4$, solvent D:

80% MeCN in water); method 3: Waters Acquity UPLC BEH C18 column (130 Å, 1.7 µm, 2.1 mm x 50 mm) on a 6 min linear gradient of 0% to 60% solvent D in solvent A (solvent A: 0.1% TFA in water, solvent D: 80% MeCN in water). Purified peptide identity shown in Table S 1and Table S 2. Analytical HPLC traces shown in Figure S 13-17.

Peptides were quantified using either NanoDrop (Thermo Scientific One Microvolume UV-Vis Spectrophotometer, for peptides 4.9-4.25 only), or UHPLC-CAD (Thermo Fisher Vanquish system) with a Waters Acquity UPLC BEH C18 column (130 Å, 1.7 µm, 2.1 mm x 50 mm) coupled to a charged aerosol detector H. Gradient: 6 min, 5-95% solvent F in solvent E, flow rate 0.45 mL/min.; solvent E: 0.05% TFA in water, solvent F: 0.05% TFA in MeCN.

Table S 1 Sequences and UPLC-MS identity of purified peptides from NNK selections and initial screening. 1C.9 and 1C.10 LC-MS data show HPLC fraction data prior to fraction pooling.

Name	Sequence	N-terminus	C-terminus	HPLC method	HPLC retention time [min]	HPLC Purity [%]	Exact mass found	Exact mass calculated [M+xH]/x	charge state MS x =
1C.1	MCIGKWYTYQYWTRC	H	GSGSDYKDDDDK-NH_2	2	7.4	81.6	1095.1	1094.5	3
1C.2	MCLLWTRHFDRYWVC	H	GSGSDYKDDDDK-NH_2	2	7.3	61.4	1104.1	1103.5	3
1C.3	MCSNMWKLANRWWAC	H	GSGSDYKDDDDK-NH_2	2	7.6	65.3	1060.9	1060.4	3
1C.4	MCVPCFSHTFLCC	Acetyl	GSGSDYKDDDDK-NH_2	1	4.1	84.3	937.9	938.0	3
1C.5	MCVPCFSHTFLCC	Acetyl	GSGSDYKDDDDK-NH_2	1	4.8	81.2	1406.2	1406.5	2
1C.6	MCIYRLSDFWVVC	Acetyl	GSGSDYKDDDDK-NH_2	3	5.9	55.0	1479.3	1478.6	2
1C.7	MCIYRLSDFWVEC	Acetyl	GSGSDYKDDDDK-NH_2	1	3.8	93.1	1494.0	1493.6	2
1C.8	MCTKFPWPFPIFWAC	H	GSGSDYKDDDDK-NH_2	2	2.0	49.5	1052.4	1051.8	3
1C.9	MCIRWPARQPLFWLC	H	GSGSDYKDDDDK-NH_2	2	8.4	75.7	1067.6	1067.2	3
1C.10	MCFRWRLFHHLTPFC	H	GSGSDYKDDDDK-NH_2	2	7.8	53.0	1092.4	1091.8	3
3C.1	MCIFDLFLPFIFC	Acetyl	GSGSDYKDDDDK-NH_2	1	6.5	91.3	1466.4	1465.6	2
3C.2	MCIFDIHMPFIPC	H	GSGSDYKDDDDK-NH_2	3	6.0	95.7	950.0	949.4	3
3C.3	MCLFDSFVPFIFC	H	GSGSDYKDDDDK-NH_2	3	6.7	83.0	1425.5	1424.6	2
3C.4	MCQQWNLYTRHWHSC	Acetyl	GSGSDYKDDDDK-NH_2	1	3.3	97.0	1105.9	1105.4	3
3C.5	MCHPTRLHLWLCCNC	Acetyl	GSGSDYKDDDDK-NH_2	1	4.2	62.1	1051.7	1051.1	3
3C.6	MCHYCFNKLFLCC	Acetyl	GSGSDYKDDDDK-NH_2	1	4.3	91.9	1473.2	1473.6	2
4.1	MCLVSIYKIMRTC	Acetyl	GSGSDYKDDDDK-NH_2	1	4.2	100.0	962.1	961.4	3
4.2	MCHVILWRSWRYC	Acetyl	GSGSDYKDDDDK-NH_2	1	4.4	69.6	1538.8	1537.7	2
4.3	MCHLAYYLAFRSC	Acetyl	GSGSDYKDDDDK-NH_2	1	4.5	88.8	1450.6	1450.1	2
4.4	MCVTWYKVTKLRC	Acetyl	GSGSDYKDDDDK-NH_2	1	3.7	89.4	985.5	984.8	3
4.5	MCFKHPWFFAWLC	Acetyl	GSGSDYKDDDDK-NH_2	1	5.7	79.2	1521.1	1519.1	2
4.6	MCIKMFLAHKRFC	Acetyl	GSGSDYKDDDDK-NH_2	3	5.0	92.3	984.4	983.8	3
4.7	MCPERLFYWLLVC	Acetyl	GSGSDYKDDDDK-NH_2	1	5.4	51.9	1000.0	998.8	3
4.8	MCEPWITIYKVLKKC	Acetyl	GSGSDYKDDDDK-NH_2	3	5.3	79.9	1060.1	1059.5	3

Table S 2 Sequences and UPLC-MS identity of purified peptides from trimer-18 selections and initial screening. Peptides purchased from GL-biochem (shanghai). GSGSEE sequence included for peptides with calculated isoelectric point (pI) of approximately 7

Name	Sequence	N-terminus	C-terminus	HPLC retention time [min]	HPLC Purity [%]	Exact mass found	calculated [M+xH]/x	state MS x =
4.9	MCTTLYYYKLWRRC	Acetyl	GSGSEEGSGSDYKDDDDK-NH_2	12.40	85.19	1257.2	1256.5	3
4.10	MCKHLYYYLLWKKC	Acetyl	GSGSEEGSGSDYKDDDDK-NH_2	13.36	88.75	1254.6	1253.9	3
4.11	MCNVLLYKVLRHC	Acetyl	GSGSDYKDDDDK-NH_2	10.41	86.88	1458.1	1457.2	2
4.12	MCESDAYRWKLWWVC	Acetyl	GSGSDYKDDDDK-NH_2	10.64	86.50	1100.1	1099.8	3
4.13	MCYTRFYNWEKWQLC	Acetyl	GSGSDYKDDDDK-NH_2	11.62	87.56	1132.2	1131.5	3
4.14	MCYHRPPNSKIWRKC	Acetyl	GSGSDYKDDDDK-NH_2	12.61	88.36	1621.6	1620.7	2
4.15	MCEREVLYWLLVTC	Acetyl	GSGSDYKDDDDK-NH_2	10.94	85.07	1541.1	1540.2	2
4.16	MCYLDDFYSYRYWAC	Acetyl	GSGSDYKDDDDK-NH_2	12.20	88.29	1661.5	1660.6	2
4.17	MCNVLLYKDLRHC	Acetyl	GSGSDYKDDDDK-NH_2	9.26	86.47	1465.7	1465.1	2
4.18	MCNDLFYYLLWRKAC	Acetyl	GSGSDYKDDDDK-NH_2	8.38	86.11	1088.1	1087.5	3
4.19	MCYNKFITLYKVLYC	Acetyl	GSGSDYKDDDDK-NH_2	10.66	85.53	1075.7	1075.1	3
4.20	MCENKKVLFYYLLWC	Acetyl	GSGSDYKDDDDK-NH_2	9.14	85.09	1092.7	1092.2	3
4.21	MCDKYWVKYWIAWRC	Acetyl	GSGSDYKDDDDK-NH_2	12.12	88.62	1125.4	1124.8	3
4.22	MCFWKKLHVFWVKDC	Acetyl	GSGSDYKDDDDK-NH_2	9.56	95.71	1098.4	1097.8	3
4.23	MCEHLYYYLLSHRRC	Acetyl	GSGSDYKDDDDK-NH_2	9.92	85.17	1104.4	1103.5	3
4.24	MCYTKAYWHEPWRPC	Acetyl	GSGSDYKDDDDK-NH_2	10.08	92.50	1098.6	1098.1	3
4.25	MCNKHLYYYLLFKQC	Acetyl	GSGSDYKDDDDK-NH_2	12.46	92.86	823.4	822.9	4

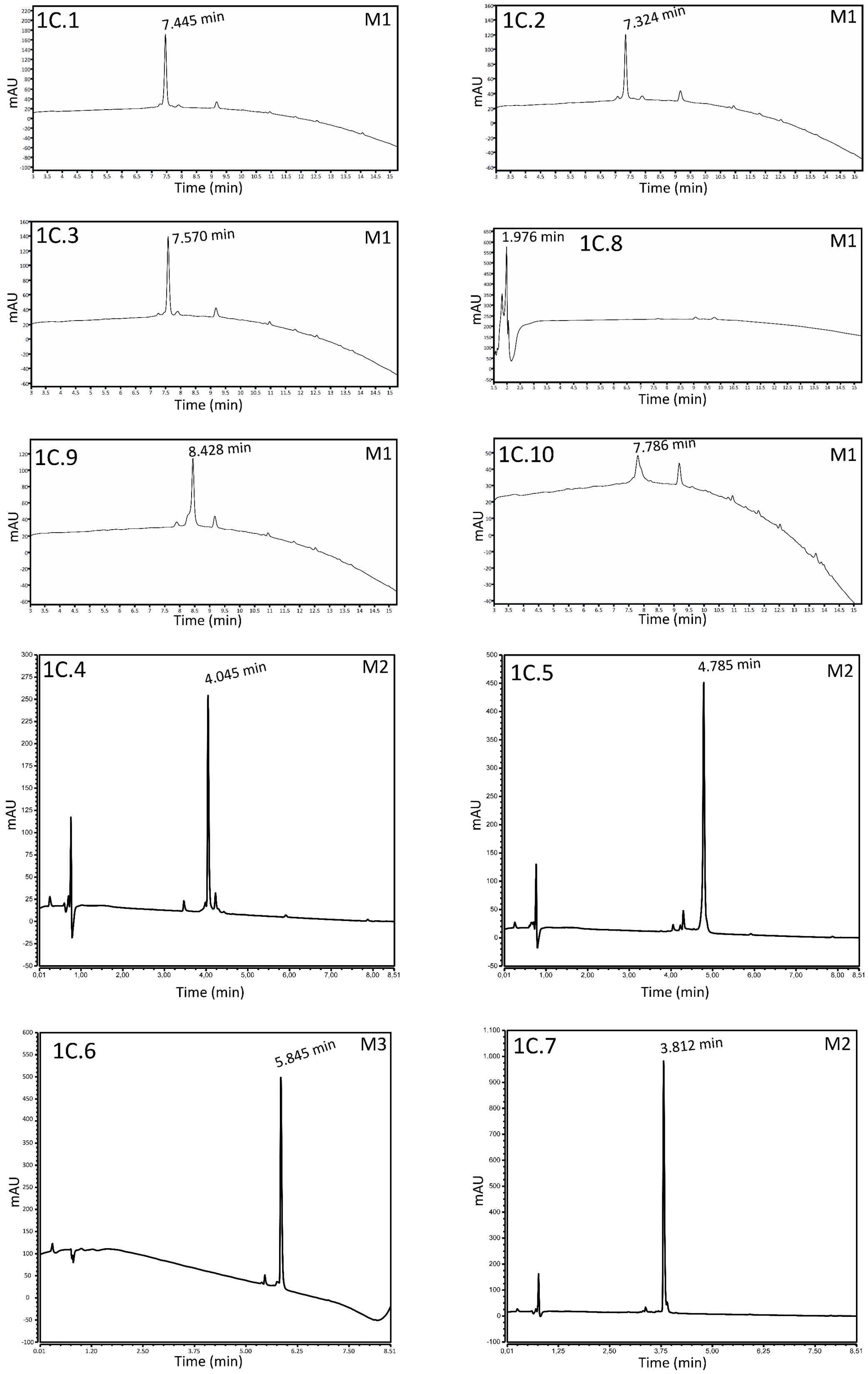

Figure S 13 HPLC traces of purified peptides from FGF-R1C NNK selection and initial screening. Method 1 (M1): Agilent 1220 system with a Phenomenex BioZen Peptide PS-C18 column (3 µm, 150 x 4.6 mm) using a 20 min linear gradient of 2% to 98% solvent B in solvent A (solvent A 0.1% TFA in water, solvent B 0.085% TFA in MeCN); method 2 (M2): Waters Acquity system with a PDA detector (MIOUPD33A), and SQ detector (BA699) using Waters Acquity UPLC BEH C18 column (130 Å, 1.7 µm, 2.1 mm x 50 mm) on a 6 min linear gradient of 0% to 80% solvent D in solvent C (solvent C: 10 mM Tris 15 mM(NH4)2SO4, solvent D: 80% MeCN in water); method 3 (M3): Waters Acquity UPLC BEH C18 column (130 Å, 1.7 µm, 2.1 mm x 50 mm) on a 6 min linear gradient of 0% to 60% solvent D in solvent A (solvent A: 0.1% TFA in water, solvent D: 80% MeCN in water).

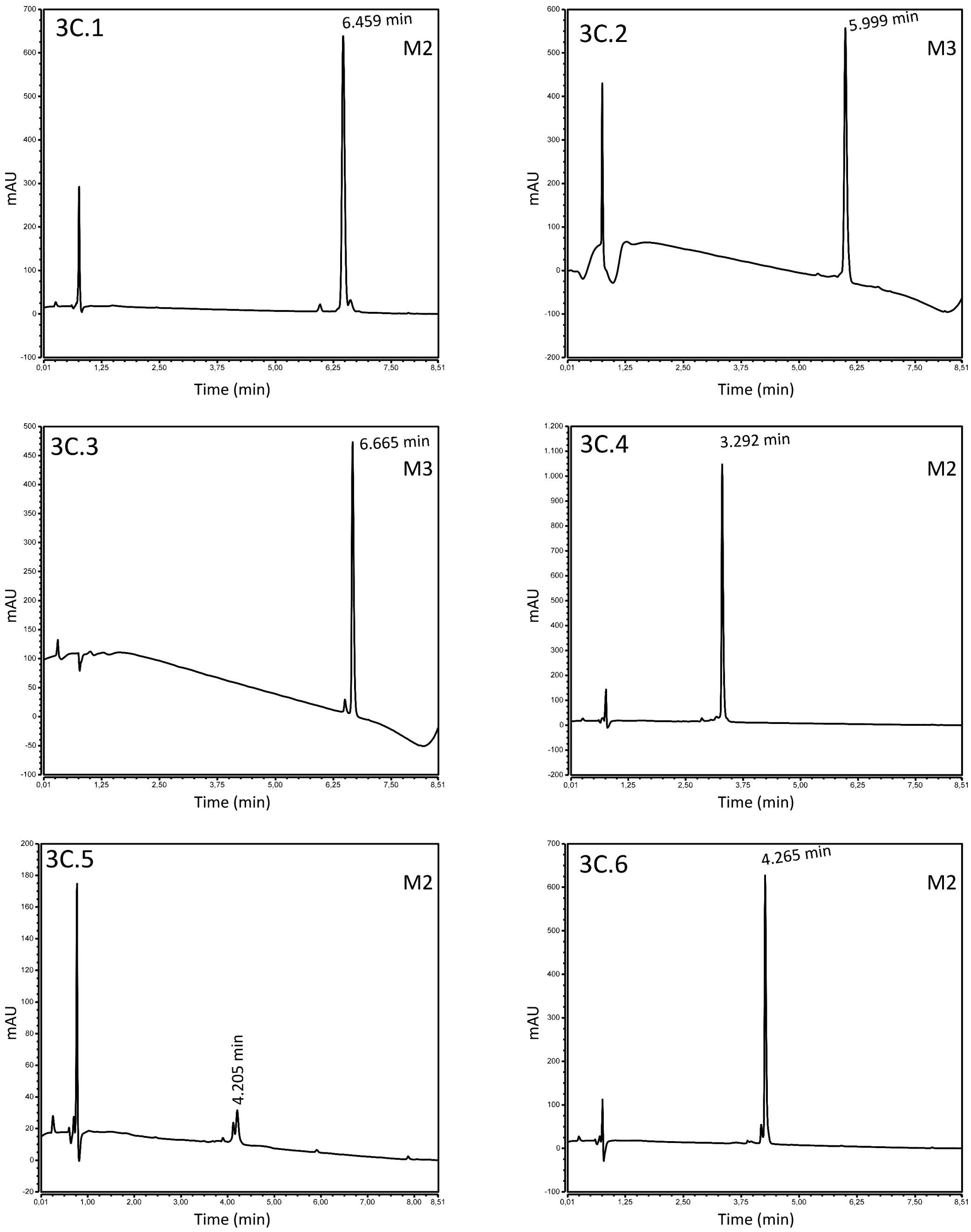

Figure S 14 HPLC traces of purified peptides from FGF-R3C NNK selection and initial screening. Method 2 (M2): Waters Acquity system with a PDA detector (MIOUPD33A), and SQ detector (BA699) using Waters Acquity UPLC BEH C18 column (130 Å, 1.7 µm, 2.1 mm x 50 mm) on a 6 min linear gradient of 0% to 80% solvent D in solvent C (solvent C: 10 mM Tris 15 mM$(NH_4)_2SO_4$, solvent D: 80% MeCN in water); method 3 (M3): Waters Acquity UPLC BEH C18 column (130 Å, 1.7 µm, 2.1 mm x 50 mm) on a 6 min linear gradient of 0% to 60% solvent D in solvent A (solvent A: 0.1% TFA in water, solvent D: 80% MeCN in water).

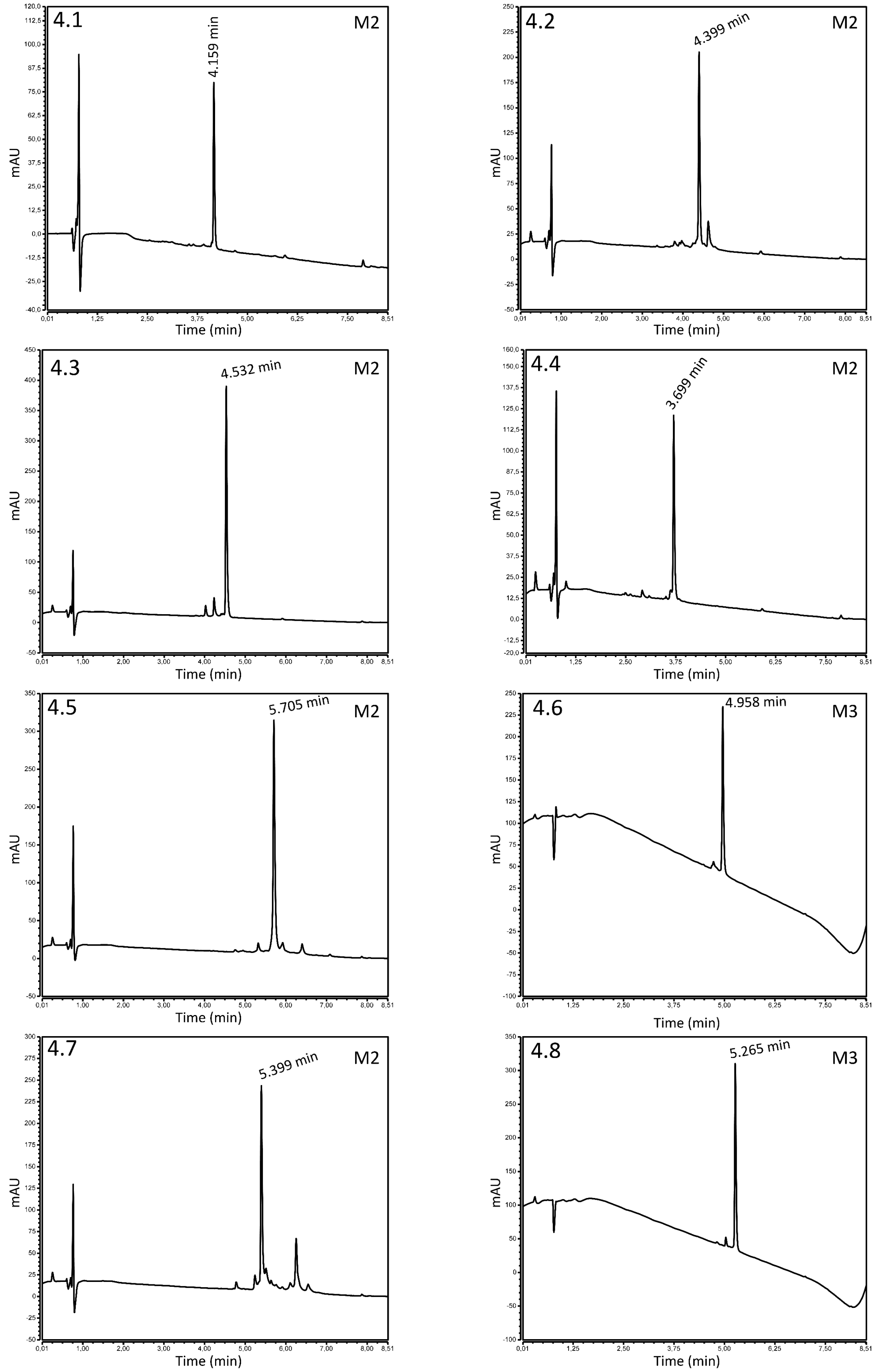

Figure S 15 HPLC traces of purified peptides from FGF-R4 NNK selection and initial screening. Method 2 (M2): Waters Acquity system with a PDA detector (MIOUPD33A), and SQ detector (BA699) using Waters Acquity UPLC BEH C18 column (130 Å, 1.7 µm, 2.1 mm x 50 mm) on a 6 min linear gradient of 0% to 80% solvent D in solvent C (solvent C: 10 mM Tris 15 mM$(NH_4)_2SO_4$, solvent D: 80% MeCN in water); method 3 (M3): Waters Acquity UPLC BEH C18 column (130 Å, 1.7 µm, 2.1 mm x 50 mm) on a 6 min linear gradient of 0% to 60% solvent D in solvent A (solvent A: 0.1% TFA in water, solvent D: 80% MeCN in water).

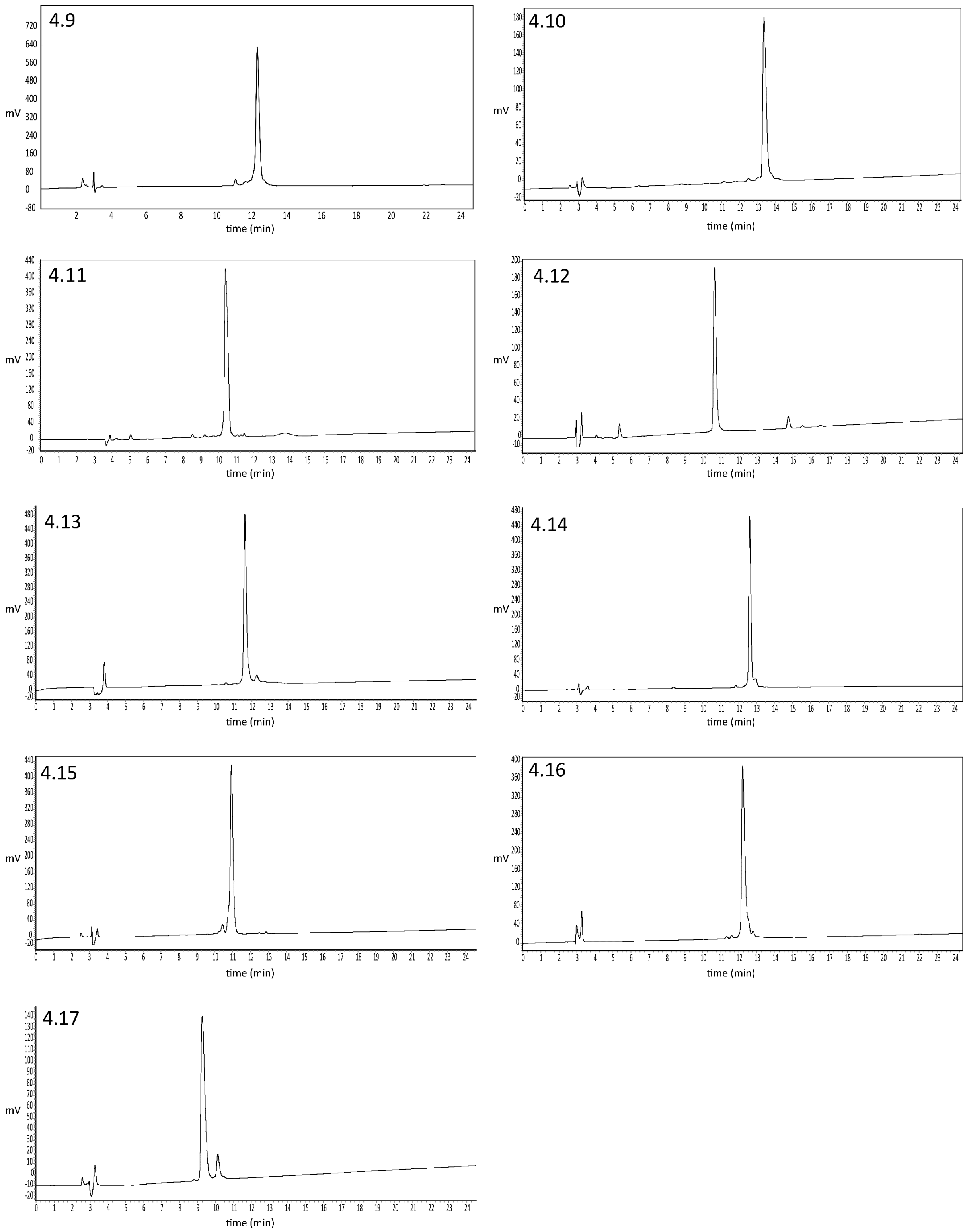

Figure S 16 HPLC traces of purified peptides from FGF-R4 T18_1 selection and initial screening. Peptides purchased from GL-biochem (shanghai). 250 x 4.6 mm, Kromasil-C18-5 µm column used on a 20 minute linear gradient of 25% to 50% solvent G in solvent A (solvent A: 0.1% TFA in water, solvent G: 0.1% TFA in MeCN).

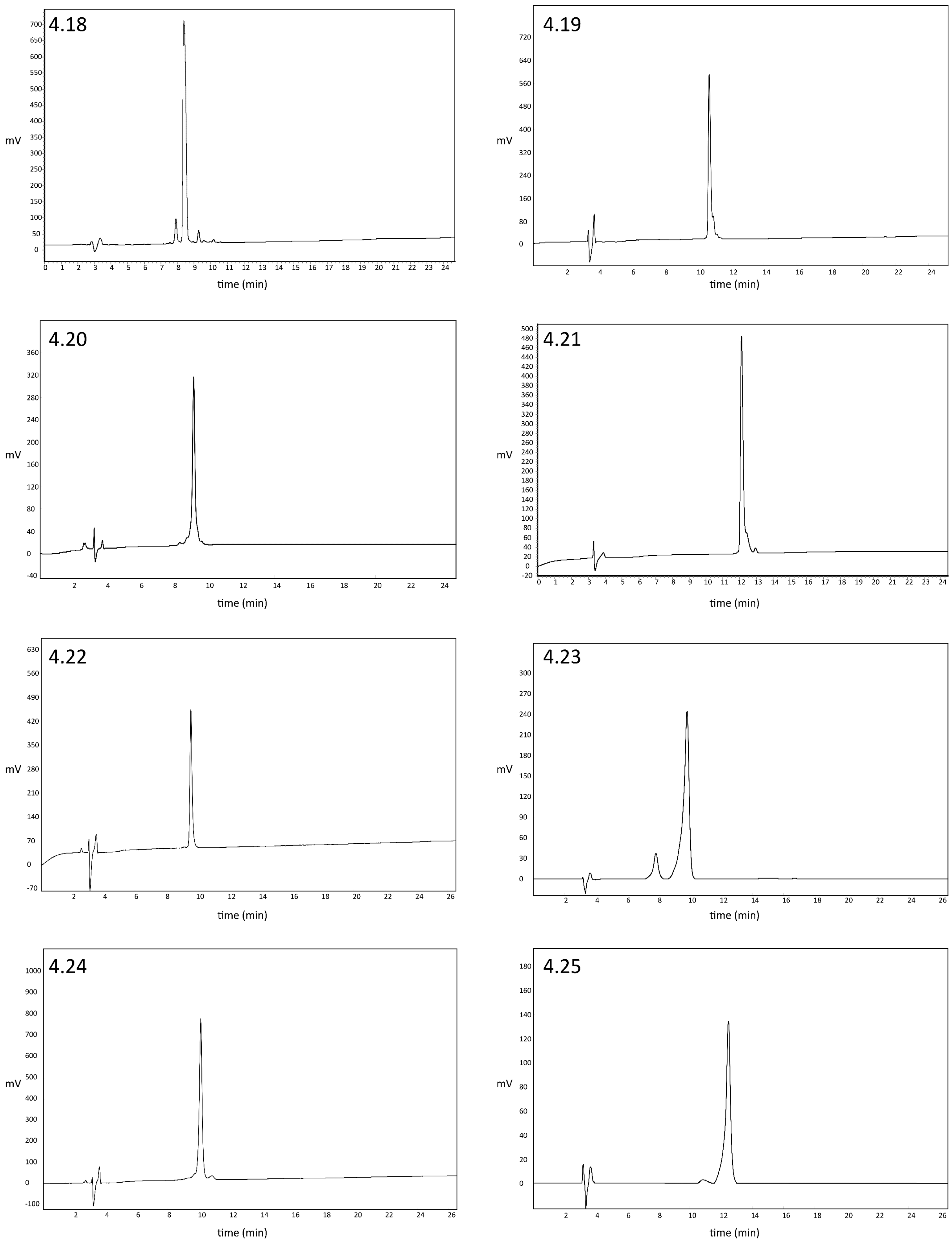

Figure S 17 HPLC traces of purified peptides from FGF-R4 T18_2 selection and initial screening. Peptides purchased from GL-biochem (shanghai). 250 x 4.6 mm, Kromasil-C18-5 µm column used on a 20 minute linear gradient of 25% to 50% solvent G in solvent A (solvent A: 0.1% TFA in water, solvent G: 0.1% TFA in MeCN).

BLI binding measurements

K_D affinity binding measurements were obtained by biolayer interferometry (BLI) instruments (Fortebio Octet HTX, or Sartorius Octet RH16 using either 8 or 16 channel mode.

For single concentration binding experiments, 50 µM peptide stocks in folding buffer were diluted 1:50 in mRNA display selection buffer (PBS pH7.4 supplemented with 0.05% Tween-20 and 0.01% BSA) in either a 96 well black plate, or a 384 well tilted bottom microplate (ForteBio). Biotinylated FGF-R protein was loaded onto Streptavidin-functionalised biosensors (ForteBio) and immersed into ~1 µM peptide to obtain association kinetics curves. Dissociation kinetics curves were obtained by subsequent immersion of ligand-associated loaded sensors into a 1:50 dilution of folding buffer in mRNA display selection buffer. Fitted K_D data was adjusted for concentration as determined by UPLC-CAD.

For accurate multi-concentration K_D determination, dilution series of each peptide were made, and association and dissociation kinetics curves were obtained as above for each concentration to fit a multi-point KD binding model. Data collection and analysis was performed using the Octet Instrument Control and Data Analysis software packages (ForteBio, Ver 9.0.0.10) or Prism (GraphPad Software, Ver. 7.05). Reference sensor was subtracted for all assays and where non-specific binding was observed, double referencing was used. Exemplary BLI kinetics curves of peptides showing different levels of selectivity to FGF-Rs is shown in Figure S 18. Single concentration BLI data shown in supplementary table S3-5 and multi-concentration assay kinetics curves shown in supplementary Figure S 19-24.

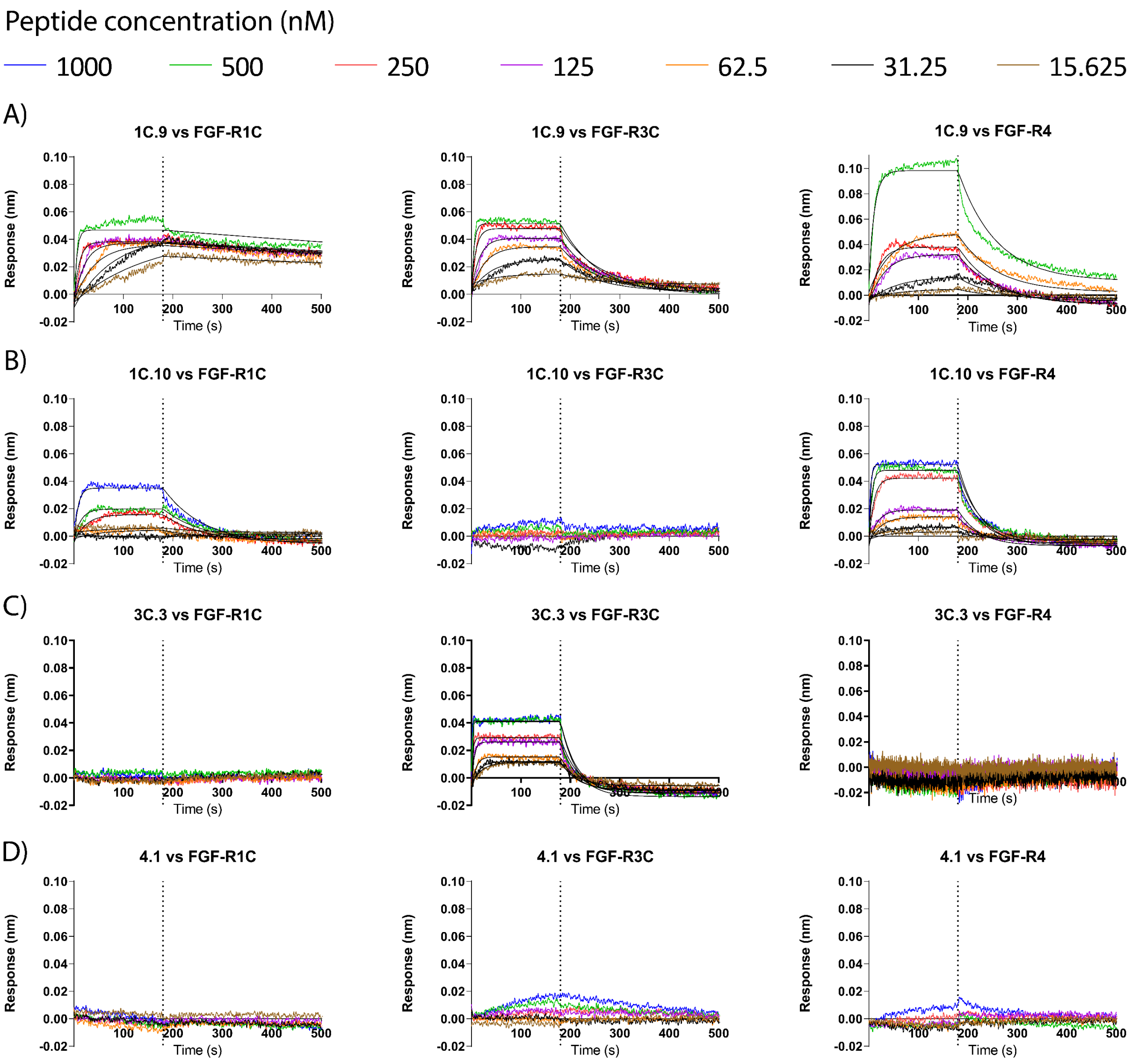

Figure S 18 Exemplary BLI kinetics curves from peptides displaying differing FGF-R selectivity. (A) pan binding 1C.9; (B) FGF-R1C and FGF-R3C binding 1C.10; (C) FGF-R3C selective 3C.3; (D) non-binding 4.1. Data from multi-concentration association and dissociation kinetics curves binding of FGF-R ECD to purified NNK CP. Data generated by Fortebio Octet HTX.

NNK single concentration BLI initial screen

Table S 3 Single concentration K_D values of crude synthesised peptides against different FGF-R ECDs and corresponding FGF-R selected against during mRNA display (NNK library). Affinity determined using single concentration BLI assays (Fortebio Octet HTX). Peptides shown were identified from NNK selections. All peptides synthesised with a C-terminal $(GS)_2$ spacer-flag-amide ($GSGSDYKDDDDK-NH_2$) and cyclised via disulfide bond. Purity determined by UPLC. Peptides with 0 % purity were followed up in multi-concentration BLI assays on pure peptide to confirm results

Name	Purity (in %)	Peptide sequence	K_D (1C) (nM)	K_D (3C) (nM)	K_D (4) (nM)	selected against
flag		GSGSDYKDDDDK	>10000	>10000	>10000	control
1C.7	51.51	MCIYRLSDFWVEC	47	281	45	FGFR-1C
	87.99	MCFRWRLFHHLTPYC	136	>10000	400	FGF-R1C
1C.2	42.79	MCLLWTRHFDRYWVC	95	>10000	>10000	FGF-R1C
1C.1	100	MCIGKWYTYQYWTRC	488	>10000	>10000	FGF-R1C
	30.18	MCILHGLIYWAPKQC	>10000	>10000	>10000	FGF-R1C
	61.38	MCHFRPNKFTAWLPC	>10000	387	>10000	FGF-R1C
1C.9	81.1	MCIRWPARQPLFWLC	19	112	82	FGF-R1C
	61.38	MCIRWPARQSLFWLC	31	463	88	FGF-R1C
	69.82	MCIRWPARQSLFWLC	85	157	>10000	FGF-R1C
	55.16	MCYKWPIRSHLYWLC	144	146	>10000	FGF-R1C
	100	MCFVYPRRQPLYWIC	147	44	>10000	FGF-R1C
	100	MCIRWPARQSLFWIC	49	65	>10000	FGF-R1C
1C.10	80.99	MCFRWRLFHHLTPFC	50	>10000	79	FGF-R1C
	68.53	MCSNMWKLANRWWAC	391	>10000	>10000	FGF-R1C
1.C3	69.11	MCSNMWKLANRWWAC	112	30	>10000	FGF-R1C
1C.8	23.51	MCTKFPWPFPIFWAC	155	47	70	FGF-R1C
	24.21	MCTKFPWPLPIFWAC	545	>10000	346	FGF-R1C
	74.28	MCYRHPHSNPLFWAC	85	>10000	>10000	FGF-R1C
	83.22	MCSYGSWYGYSYLAC	>10000	>10000	>10000	FGF-R1C
	50.17	MCYRFPSSHLFWAC	>10000	>10000	93	FGF-R1C
	100	MCIFELNLPFIPC	>10000	>10000	>10000	FGF-R1C
1.C6	62.55	MCIYRLSDFWVVC	19	12	47	FGF-R1C
1C.4	56.69	MCVPCFSHTFLCC	>10000	475	>10000	FGF-R1C
3C.4	75.4	MCQQWNLYTRHWHSC	>10000	>10000	>10000	FGF-R3C
3C.5	0	MCHPTRLHLWLCCNC	>10000	271	>10000	FGF-R3C
	64.14	MCQQWNLYTRHWHIC	>10000	>10000	>10000	FGF-R3C
3C.6	0	MCHYCFNKLFLCC	522	37	>10000	FGF-R3C
	79.77	MCIFDNWLPFIYC	>10000	91	>10000	FGF-R3C
	92.48	MCIFDTHMPFIPC	>10000	>10000	>10000	FGF-R3C
	71.94	MCIFDTFLPFIFC	>10000	100	>10000	FGF-R3C
3C.2	85.5	MCIFDIHMPFIPC	>10000	>10000	>10000	FGF-R3C
	41.9	MCIFDNWLPFIFC	>10000	76	>10000	FGF-R3C
	55.83	MCIWDSFVPFILC	>10000	85	>10000	FGF-R3C
3C.1	0	MCIFDLFLPFIFC	>10000	64	>10000	FGF-R3C
	93.22	MCLFDLHLPFIFC	>10000	172	>10000	FGF-R3C
3C.3	78.93	MCLFDSFVPFIFC	>10000	29	>10000	FGF-R3C
	78.76	MCLYDSFLPFIFC	>10000	84	>10000	FGF-R3C
	41.31	MCTFDLFLPFIFC	>10000	177	>10000	FGF-R3C
	76.88	MCTFDTHFPFITC	>10000	287	>10000	FGF-R3C
	82.09	MCVFDSFLPFIIC	>10000	38	>10000	FGF-R3C
	86.52	MCVFDSWLPFVYC	>10000	618	>10000	FGF-R3C
	91.39	MCVFDSFLPFVLC	>10000	60	>10000	FGF-R3C
	78.64	MCVFDSWLPFVFC	>10000	1064	>10000	FGF-R3C
	52.63	MCVYDIFVPFIIC	>10000	29	>10000	FGF-R3C
	67.66	MCVYDLFLPFIYC	>10000	126	>10000	FGF-R3C
	87.07	MCYFDPHFIFIPC	>10000	>10000	>10000	FGF-R3C
4.8	0	MCEPWITIYKVLKKC	>10000	1254	287	FGF-R4
	75.8	MCPKYFLLWRLMTC	>10000	>10000	1836	FGF-R4
	34.38	MCNIILYKIMKNRC	>10000	>10000	>10000	FGF-R4
	60.15	MCSHCWLFKVLLM	>10000	>10000	180	FGF-R4
	60.67	MCAPKWLIKVLSC	>10000	>10000	>10000	FGF-R4
	40.37	MCDCHPLLCFLLC	>10000	>10000	>10000	FGF-R4
	77.3	MCDVLLYKLFRYC	111	1927	32	FGF-R4
	50.76	MCDCLHVVCFLVC	>10000	>10000	>10000	FGF-R4
4.5	0	MCFKHPWFFAWLC	>10000	>10000	35	FGF-R4
	61.47	MCFALLYKILSRC	>10000	>10000	>10000	FGF-R4
	76.61	MCFWPIWYKFMAC	>10000	>10000	>10000	FGF-R4
	35.76	MCFWRFFPALLAC	>10000	>10000	>10000	FGF-R4
	63.78	MCGHFSITWFKWC	>10000	>10000	>10000	FGF-R4
	54.21	MCGVLLYKVMRVC	>10000	>10000	124	FGF-R4
4.3	54.88	MCHLAYYLAFRSC	>10000	>10000	58	FGF-R4
	44.09	MCHCFPTICWLVC	>10000	>10000	>10000	FGF-R4
	73.32	MCHCFPTICWLEC	>10000	>10000	>10000	FGF-R4
	32.77	MCHCFPIVCWFEC	>10000	>10000	>10000	FGF-R4
4.2	64.78	MCHVILWRSWRYC	>10000	>10000	93	FGF-R4
	42.81	MCHITIYKILRYC	>10000	>10000	124	FGF-R4
	72.16	MCILLYKVLRGRC	>10000	>10000	128	FGF-R4
4.6	0	MCIKMFLAHKRFC	>10000	>10000	97	FGF-R4
	54.65	MCIHRLSDFWVVC	19	49	41	FGF-R4
	34.87	MCIYRLSDFWAVC	21	49	133	FGF-R4
	55.56	MCIKTFLLWRSSC	>10000	>10000	>10000	FGF-R4
	63.23	MCKKHLLYYLIAC	>10000	>10000	115	FGF-R4
4.1	71.68	MCLVSIYKIMRTC	>10000	6121	193	FGF-R4
	51.75	MCLISIYKVLKHC	>10000	>10000	>10000	FGF-R4
	67.27	MCLAYYLAFHSLC	>10000	2555	126	FGF-R4
	52.57	MCNILLYKILSVC	>10000	>10000	105	FGF-R4
	57.48	MCPVIIYKILSHC	>10000	>10000	198	FGF-R4
4.7	0	MCPERLFYWLLVC	>10000	>10000	68	FGF-R4
	69.38	MCPRWIYYYMLHC	>10000	>10000	139	FGF-R4
	63.09	MCPKWLIKVLLNC	>10000	>10000	4194	FGF-R4
	63.82	MCPKWFILWRQLC	>10000	>10000	>10000	FGF-R4
	42.96	MCRDFWHLWFRVC	261	>10000	>10000	FGF-R4
	57.23	MCRELLYYLLQRC	>10000	>10000	59	FGF-R4
	67.7	MMSHCWLFKVLLC	>10000	>10000	239	FGF-R4
	37.51	MCSCFPPLCFLIC	>10000	>10000	>10000	FGF-R4
	82.97	MCSILLYKVLSQC	>10000	>10000	84	FGF-R4
	47.44	MCSILLYKVLSRC	>10000	>10000	>10000	FGF-R4
	65.74	MCSITIYKLLRFC	>10000	591	192	FGF-R4
	79.27	MCVVCYKAFVMCC	>10000	>10000	3332	FGF-R4
	16.68	MMVKCWLAYIRTC	>10000	>10000	>10000	FGF-R4
	68.07	MCVWPKWLTKLIC	>10000	>10000	174	FGF-R4
4.4	0	MCVTWYKVTKLRC	>10000	>10000	57	FGF-R4
	66.54	MCVFWTGMSIWIC	>10000	>10000	>10000	FGF-R4
	72.79	MCVSYYRAYFAHC	>10000	2996	102	FGF-R4
	75.7	MCVKYYLIMKSLC	>10000	>10000	>10000	FGF-R4
	33.24	MCWWFMPELVLKC	>10000	>10000	>10000	FGF-R4
	78.56	MCWVKYWTVYKQC	>10000	>10000	207	FGF-R4
	52.31	MCYKLFLLWKQWC	>10000	>10000	>10000	FGF-R4
	60.09	MCHYISDWLC	>10000	>10000	>10000	FGF-R4

NNK purified peptide BLI binding data

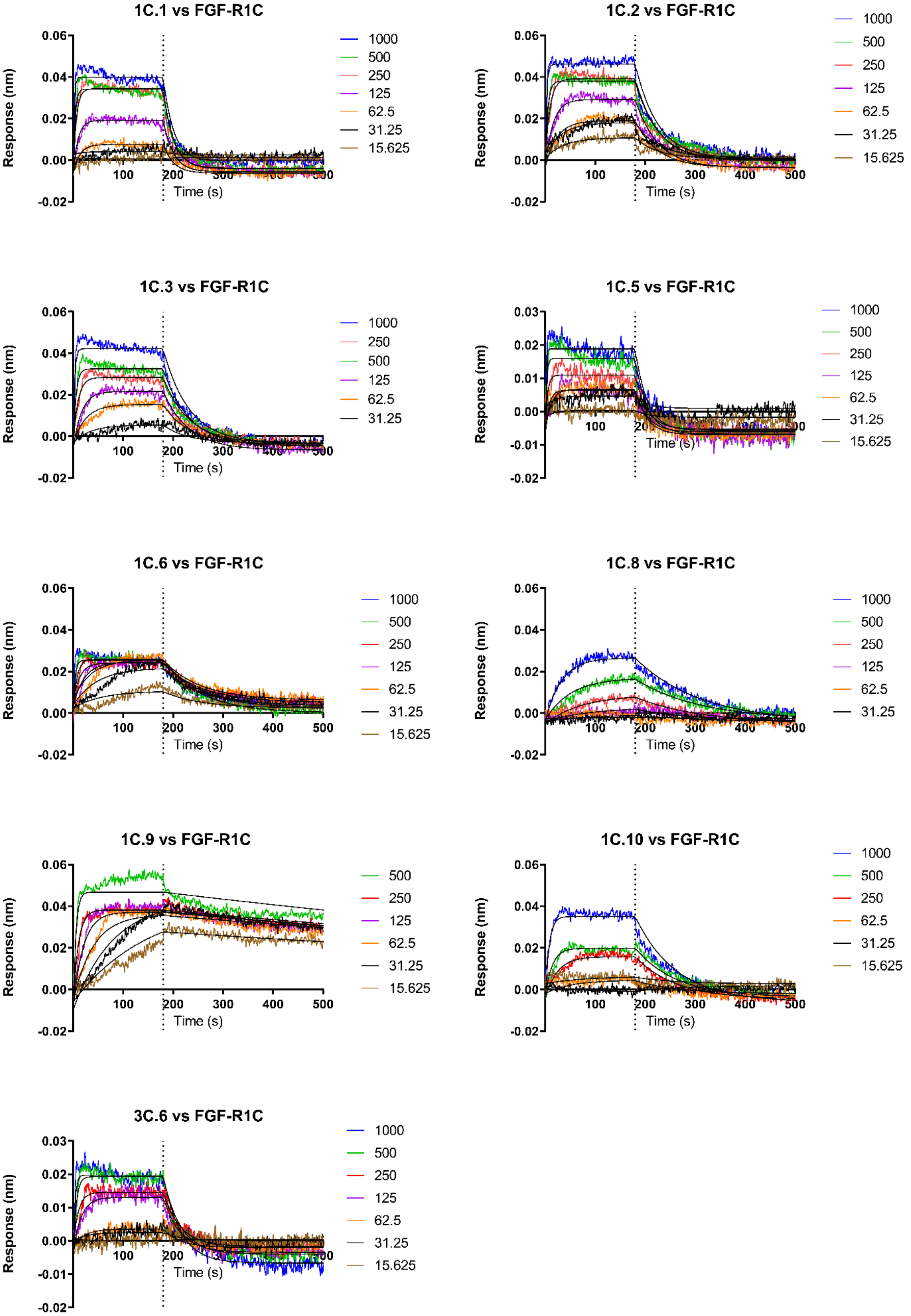

Figure S 19 Purified NNK CP multi-concentration association and dissociation kinetics curves binding to FGF-R1C ECD. Data generated by Fortebio Octet HTX. Concentrations shown in nM

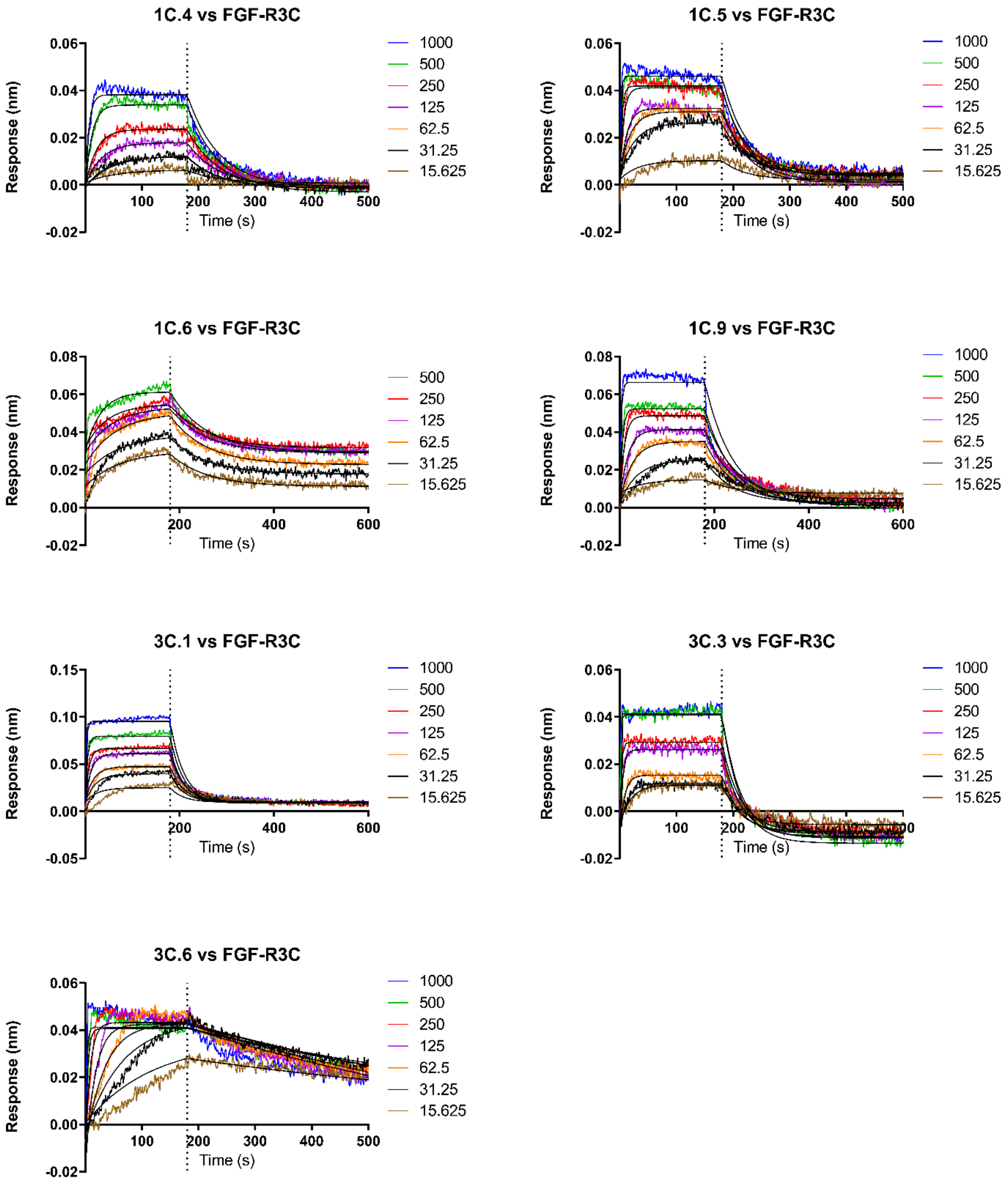

Figure S 20 Purified NNK CP multi-concentration association and dissociation kinetics curves binding to FGF-R3C ECD. Data generated by Fortebio Octet HTX. Concentrations shown in nM.

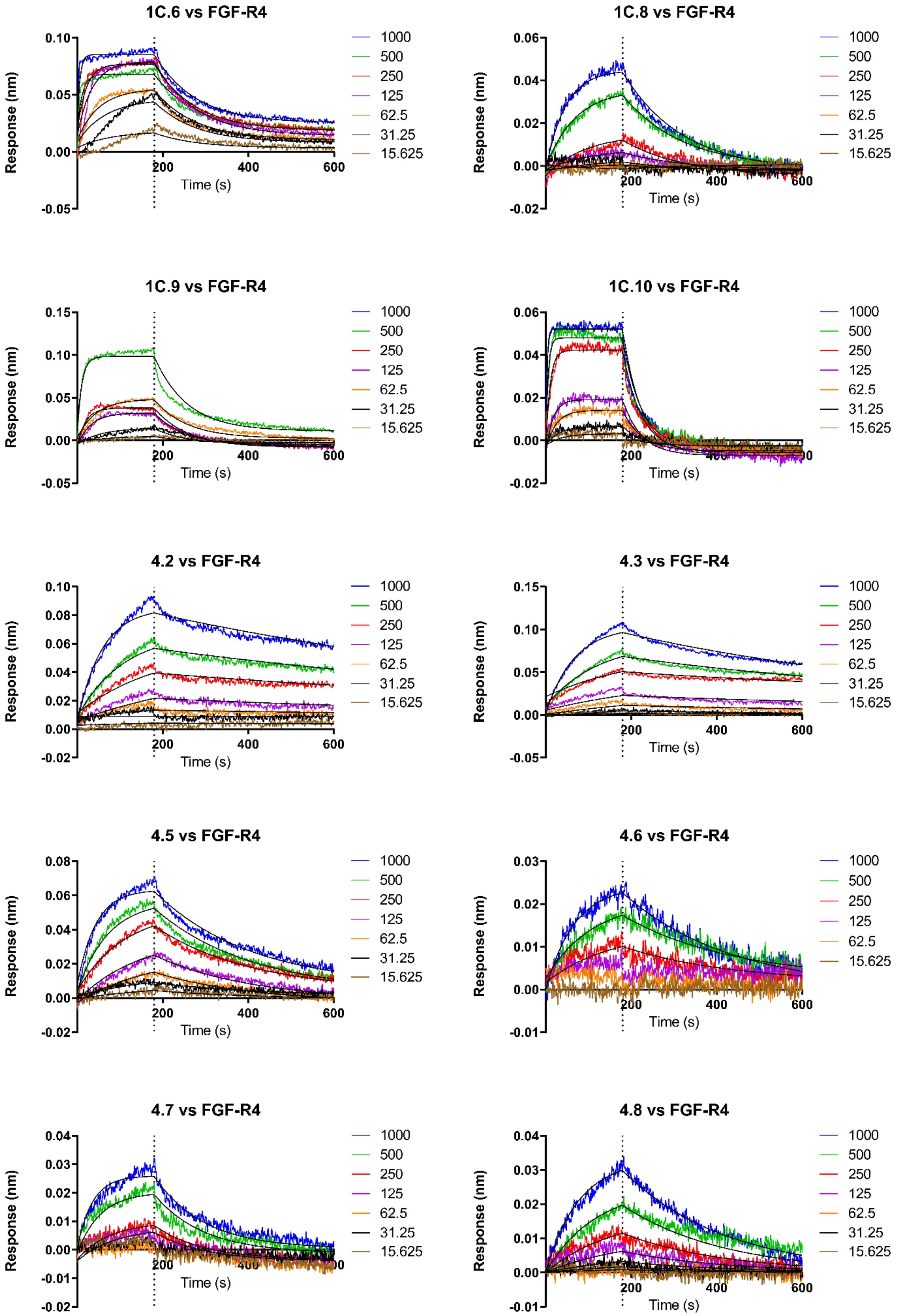

Figure S 21 Purified NNK CP multi-concentration association and dissociation kinetics curves binding to FGF-R4 ECD. Data generated by Fortebio Octet HTX. Concentrations shown in nM

Trimer-18 single concentration BLI initial screen

Table S 4 Single concentration K_D values of crude synthesised peptides against different FGF-R ECDs and corresponding FGF-R selected against during mRNA display from T18 selection1. Peptides shown were identified from trimer-18 selection 1 using a 1:1:1 library of 10-12mer variable codons. Affinity determined using single concentration BLI assays. All peptides synthesised with a C-terminal $(GS)_2$ spacer-flag-amide ($GSGSDYKDDDDK-NH_2$), N-terminal Acetyl, and cyclised via disulfide bond. GSGSEE sequence included for peptides with calculated isoelectric point (pI) of approximately 7. Purity determined by UPLC.

Name	Purity (in %)	Peptide sequence	K_D (1C) (nM)	K_D (3C) (nM)	K_D (4) (nM)	selected against
4.11	78.00	MCNVLLYKVLRHC	>10000	>10000	11	FGF-R4
4.17	65.14	MCNVLLYKDLRHC	>10000	>10000	>10000	FGF-R4
	54.80	MCHLLYYKLFHSC	63	>10000	114	FGF-R4
4.9	77.24	MCTTLYYYKLWRRCGSGSEE	699	2247	1	FGF-R4
	30.34	MCTYVYYYKLFHRC	>10000	>10000	3	FGF-R4
	19.95	MCREIRYWQLYRAFC	>10000	>10000	A.D.	FGF-R4
	42.58	MCRHLFYWKLWHQRCGSGSEE	>10000	>10000	2	FGF-R4
	47.25	MCRWVKYYQLVHRKC	>10000	>10000	1	FGF-R4
	40.28	MCREIRYWQLWQC	>10000	>10000	27	FGF-R4
	44.06	MCYNIYWYKYVRREC	387	>10000	1	FGF-R4
	59.54	MCHIRYWQLVRKHCGSGSEE	>10000	>10000	10	FGF-R4
	33.86	MCEVKYFLIWKRWC	650	>10000	15	FGF-R4
	50.47	MCYTVLWYKYQKKCGSGSEE	>10000	>10000	1	FGF-R4
	49.15	MCPYAIYWYKWRKTCGSGSEE	>10000	>10000	11	FGF-R4
	33.38	MCEHLFYYLLVKRQC	>10000	>10000	1	FGF-R4
	47.25	MCEDLFYYIINRYGC	>10000	>10000	18	FGF-R4
	41.72	MCSILWYKITKPLC	>10000	>10000	13	FGF-R4
	38.42	MCEVIWYKIQWRKC	>10000	>10000	12	FGF-R4
4.10	47.42	MCKHLYYYLLWKKCGSGSEE	1029	928	1	FGF-R4
	19.38	MCEDLFYYLLFHHC	4537	>10000	81	FGF-R4
	89.29	MCYKHILWYKIQKLCGSGSEE	33	>10000	1	FGF-R4
	42.02	MCYEIIWYKLQRVC	>10000	>10000	A.D.	FGF-R4
	27.66	MCLWYYLQFHRREC	21	>10000	5	FGF-R4
	22.18	MCAVYYKLWLKHSRCGSGSEE	>10000	A.D.	7	FGF-R4
	48.94	MCKEYEILWYKLQYC	>10000	>10000	17	FGF-R4
	26.58	MCDLYTVLWYKIQKEC	21	>10000	19	FGF-R4
	36.14	MCAKYFLIWRAYQQC	>10000	>10000	10	FGF-R4
	12.76	MCAKAFLLWKGKFCGSGSEE	>10000	>10000	>10000	FGF-R4
	47.51	MCPIHFFKIVYRHCGSGSEE	10	>10000	10	FGF-R4
	57.28	MCLWHYYKIFRRQKC	96	>10000	8	FGF-R4
	38.86	MCEWFYYHKSWYVYC	191	>10000	>10000	FGF-R4
	55.61	MCHRYIYLDYWHC	>10000	>10000	44	FGF-R4
	63.49	MCLRHLYEPYWHC	>10000	>10000	>10000	FGF-R4
	70.25	MCYKNISTGIFLYC	>10000	>10000	A.D.	FGF-R4
	65.58	MCYKNIDNGVLVFC	>10000	>10000	14	FGF-R4
	36.61	MCTYDFVLGHHYLIC	>10000	>10000	>10000	FGF-R4
	58.48	MCTYEFLFEELYLHC	>10000	>10000	>10000	FGF-R4
	62.65	MCYYNFLNKVLVYC	>10000	>10000	8	FGF-R4
	49.84	MCFLDHKTKVFVYC	>10000	>10000	130	FGF-R4
	55.20	MCDSIRAQYARWWIC	>10000	>10000	183	FGF-R4
4.12	59.42	MCESDAYRWKLWWVC	1567	>10000	10	FGF-R4
	46.75	MCYDIVFDYITVYHC	>10000	>10000	21	FGF-R4
	39.17	MCVWQYSHHYNWHC	>10000	>10000	>10000	FGF-R4
	29.62	MCVWKYRLDRNWYC	78	>10000	2043	FGF-R4
	34.21	MCWWFKQHEFWFKVC	>10000	>10000	2	FGF-R4
	58.39	MCWYDQHSFYWERLC	>10000	>10000	>10000	FGF-R4
	34.92	MCKYATLQWYKWFYC	114	7341	63	FGF-R4
	53.42	MCPYDYYDWHDWRDC	>10000	>10000	1585	FGF-R4
	11.27	MCWSKLIIKYYLSQC	>10000	>10000	16	FGF-R4
	100.00	MCNLSIIHGYVAYIC	>10000	>10000	1	FGF-R4
4.16	23.91	MCYLDDFYSYRYWAC	54	>10000	>10000	FGF-R4
	40.00	MCHIWYFNNQFHYVC	>10000	A.D.	6	FGF-R4
	43.18	MCKIWYVDHKFWWVC	>10000	>10000	2	FGF-R4
	31.04	MCVWWSHPSIWIC	65	>10000	>10000	FGF-R4
	38.56	MCHRHLYYWRLFHSCGSGSEE	>10000	>10000	5	FGF-R4
4.15	54.53	MCEREVLYWLLVTC	48	>10000	128	FGF-R4
	49.40	MCKRHIRYWQLYVCGSGSEE	265	838	A.D.	FGF-R4
	21.16	MCKREIKYWLVFC	>10000	>10000	8	FGF-R4
	45.38	MCAKEHLFYYLLWC	>10000	>10000	41	FGF-R4
	68.57	MCKRHLIYYKIARC	>10000	1540	1	FGF-R4
	40.68	MCIRYLFYYLISHC	>10000	>10000	2	FGF-R4
	36.54	MCPYYVQYWRVFC	>10000	>10000	9	FGF-R4
	52.99	MCTGRHLYYWKLFHCGSGSEE	>10000	>10000	10	FGF-R4
	47.51	MCDAYYVRYWQVLHC	>10000	>10000	36	FGF-R4
	34.45	MCKYSKDLWLYRLWC	>10000	>10000	2	FGF-R4
	38.33	MCYKYSKDLWLYRLWC	502	>10000	22	FGF-R4
	59.90	MCIKDLHLFYYLLC	30	>10000	14	FGF-R4
	46.62	MCDKFVKWYIVFRHCGSGSEE	>10000	>10000	1	FGF-R4
	54.15	MCVKYIKYWSVFHC	>10000	>10000	17	FGF-R4
	34.72	MCDWYVTIYKVLKKC	>10000	>10000	8	FGF-R4
	28.19	MCYEDEIWLYKVLYC	>10000	>10000	9	FGF-R4
	50.13	MCYHRHIFQNHWYKCGSGSEE	A.D.	A.D.	A.D.	FGF-R4
4.14	71.65	MCYHRPPNSKIWRKC	>10000	>10000	214	FGF-R4
	69.02	MCEQRSRWSNYWYPC	>10000	>10000	>10000	FGF-R4
	29.58	MCYTRFWTEEWQPC	>10000	>10000	>10000	FGF-R4
	64.78	MCYTRHPYEALWHQC	>10000	>10000	>10000	FGF-R4
	73.30	MCTHRHPYETLWHQC	>10000	>10000	>10000	FGF-R4
4.13	62.39	MCYTRFYNWEKWQLC	17	>10000	>10000	FGF-R4
	61.82	MCITRKNKWEEWRPC	>10000	>10000	>10000	FGF-R4
	58.43	MCEHRHPYGHWEQC	>10000	>10000	>10000	FGF-R4

Table S 5 Single concentration K_D values of crude synthesised peptides against different FGF-R ECDs and corresponding FGF-R selected against during mRNA display from T18 selection 2. Peptides shown were identified from trimer-18 selection 2 using a 1:18:324 library of 10-12mer variable codons. Affinity determined using single concentration BLI assays. All peptides synthesised with a C-terminal $(GS)_2$ spacer-flag-amide ($GSGSDYKDDDDK-NH_2$), N-terminal Acetyl, and cyclised via disulfide bond. GSGSEE sequence included for peptides with calculated isoelectric point (pI) of approximately 7. Purity determined by UPLC.

Name	Purity (in %)	Peptide sequence	K_D (1C) (nM)	K_D (3C) (nM)	K_D (4) (nM)	selected against
	28.05	MCFHIKANYGFVRVC	>10000	>10000	>10000	FGF-R4
	31.99	MCLHIKANYGFVRVC	>10000	>10000	>10000	FGF-R4
	24.56	MCTYTYVFNQTYVHC	>10000	A.D.	>10000	FGF-R4
	29.92	MCVYEYVFDRLTVTC	>10000	>10000	>10000	FGF-R4
	28.18	MCEYTYLFGYHYIKC	>10000	>10000	>10000	FGF-R4
	15.76	MCAYEYVFDRLTVTC	A.D.	>10000	28	FGF-R4
	30.02	MCHNKYLYYYLLYSC	>10000	>10000	36	FGF-R4
	35.72	MCYKRDLYYYLVFKC	>10000	A.D.	8	FGF-R4
	26.24	MCDKRHLYYYLLVC	>10000	>10000	32	FGF-R4
4.21	22.3	MCDKYWVKYWIAWRC	A.D.	>10000	1	FGF-R4
	28.38	MCEREFLYYYLYYKC	>10000	A.D.	>10000	FGF-R4
	17.18	MCWEEFTKFGWWTIC	>10000	>10000	1	FGF-R4
	17.17	MCFKQHLFYYLLHC	>10000	>10000	22	FGF-R4
4.19	27.72	MCYNKFITLYKVLYC	505	79	13	FGF-R4
	25.37	MCHDKFITLYKVLYC	>10000	A.D.	48	FGF-R4
	27.34	MCYYEVLWYQQQRHC	>10000	>10000	12	FGF-R4
	30.15	MCRDFDIIWYKLQFC	5364	340	20	FGF-R4
	42.08	MCKYSVYWYKWQRKC	>10000	A.D.	A.D.	FGF-R4
	16.57	MCEFEIRWYQYQRKC	>10000	>10000	16	FGF-R4
	18.94	MCWEEIIWYKLQHKC	A.D.	>10000	7	FGF-R4
	18.42	MCDNRRWVRYYQIFC	>10000	>10000	11	FGF-R4
4.20	27.69	MCENKKVLFYYLLWC	>10000	>10000	<1	FGF-R4
	19.7	MCDLKRELFYYLLVC	>10000	A.D.	27	FGF-R4
	32.31	MCDHKRHLYYYILWC	>10000	>10000	5	FGF-R4
	30.31	MCKFDLDLLFYKIWC	>10000	>10000	9	FGF-R4
	30.35	MCHFKRDYYWYLWQC	>10000	>10000	73	FGF-R4
	27.43	MCKYHRDLLYYFLHC	>10000	>10000	23	FGF-R4
	7.25	MCVHKKWIRYWQIFCGSGSEE	A.D.	>10000	6	FGF-R4
	21.06	MCFWHKRSDFWLQFC	A.D.	>10000	367	FGF-R4
	36.05	MCWWFRNHKFWQQIC	>10000	>10000	9	FGF-R4
4.22	24.34	MCFWKKLHVFWVKDC	23	>10000	A.D.	FGF-R4
	26.53	MCWYVQHSFYWERLC	>10000	A.D.	33	FGF-R4
	41.92	MCWWIQHPLFWSKIC	A.D.	>10000	22	FGF-R4
	33.09	MCFRHKRSDFWLQFC	>10000	>10000	>10000	FGF-R4
	36.29	MCEHRHPYSNFWILC	>10000	>10000	>10000	FGF-R4
	48.58	MCEHRHPFTQYWLRC	>10000	>10000	>10000	FGF-R4
	53.91	MCEHRHPYEAFWHEC	>10000	>10000	>10000	FGF-R4
	63.29	MCVHRHRFEPEWHVC	>10000	>10000	>10000	FGF-R4
	45.07	MCAYRHIYEPYWHSC	>10000	>10000	>10000	FGF-R4
	42.83	MCEHKHPFERVWRSC	>10000	>10000	>10000	FGF-R4
	44.26	MCEYRHKFEERWHVC	>10000	>10000	>10000	FGF-R4
	54.81	MCEHRHPYSNFWIPC	>10000	>10000	>10000	FGF-R4
	33.02	MCYTRHPFQKRWHYCGSGSEE	>10000	>10000	>10000	FGF-R4
4.24	49.75	MCYTKAYWHEPWRPC	>10000	3148	>10000	FGF-R4
	29.41	MCYTRKPFEFFWRLC	>10000	A.D.	168	FGF-R4
	37.7	MCYTRHKFDHRWRACGSGSEE	>10000	>10000	>10000	FGF-R4
	34.3	MCYTRDRYELHWRLC	>10000	>10000	>10000	FGF-R4
	52.97	MCYTRHKFDQYWQPC	>10000	>10000	>10000	FGF-R4
	21.14	MCITRHRFEQFWHLC	>10000	>10000	>10000	FGF-R4
	27.67	MCYTKATVFSQWVLC	>10000	>10000	>10000	FGF-R4
	54.73	MCYTRRFKWQDWHAC	>10000	A.D.	>10000	FGF-R4
	33.84	MCYTKYFYFEPWRIC	>10000	>10000	>10000	FGF-R4
	40.25	MCTTRKNKWEEWRPC	>10000	>10000	>10000	FGF-R4
	38.6	MCYTKSFYWPIWRSC	>10000	A.D.	>10000	FGF-R4
	41.33	MCYTRLNKHAKWQKC	>10000	A.D.	149	FGF-R4
	27.09	MCTDLYYYLIWQRYC	>10000	>10000	10	FGF-R4
4.18	19.04	MCNDLFYYLLWRKAC	A.D.	>10000	1	FGF-R4
	52.09	MCKHLFYYKLIRKAC	>10000	>10000	7	FGF-R4
	22.55	MCVDLFYYKLFRKLC	>10000	>10000	9	FGF-R4
	42.02	MCPYTKYWIVWRHSC	>10000	>10000	3	FGF-R4
4.23	42.61	MCEHLYYYLLSHRRC	172	>10000	7	FGF-R4
	24.68	MCRELFYYRFWQKTC	>10000	>10000	14	FGF-R4
	45.99	MCRHLAYWKLWHRKC	>10000	A.D.	8	FGF-R4
	12.32	MCDIVIYKILRSLC	>10000	3	4	FGF-R4
	21.21	MCDLFYYLQHHKKVC	>10000	>10000	8	FGF-R4
	18.41	MCYVQYYRVWRSIC	>10000	>10000	4	FGF-R4
	25.46	MCEVIWYKIQYRLC	>10000	A.D.	21	FGF-R4
	17.33	MCDLFYYLFIKRRKCGSGSEE	>10000	A.D.	10	FGF-R4
	19.76	MCHLWYYLVHHRRDC	>10000	A.D.	6	FGF-R4
	11.65	MCVIVIYKILRSLC	>10000	A.D.	>10000	FGF-R4
	60.49	MCYNVHWYRWIRRRC	>10000	>10000	>10000	FGF-R4
	19.23	MCYEVLWYKLQRKHC	A.D.	>10000	3	FGF-R4
	13.76	MCYTELWYKYIRRHC	1120	>10000	6	FGF-R4
	15.39	MCYDVLWYRYRYKQC	>10000	>10000	22	FGF-R4
	24.43	MCKIIWLYKAARRFC	>10000	A.D.	7	FGF-R4
	15.96	MCYEVLWYQYWRKVC	>10000	>10000	22	FGF-R4
	24.32	MCFNVHWYRWIRRRC	>10000	A.D.	3	FGF-R4
	26.09	MCNKHLYYYLLFKHC	>10000	A.D.	9	FGF-R4
	25.95	MCPRYVLYYKLFHHC	>10000	>10000	8	FGF-R4
	13.54	MCKRHITYWKLWHFCGSGSEE	A.D.	A.D.	10	FGF-R4
	16.46	MCHDYIVFYKVLRHC	>10000	>10000	17	FGF-R4
	39.1	MCRRELYYYLLYSKC	>10000	>10000	5	FGF-R4
	32.24	MCDRLILYYRIVHKC	>10000	>10000	22	FGF-R4
	28.11	MCPYEIKYWIVWQQC	>10000	>10000	14	FGF-R4
4.25	22.94	MCNKHLYYYLLFKQC	>10000	>10000	<1	FGF-R4

Trimer-18 Purified peptide BLI binding data

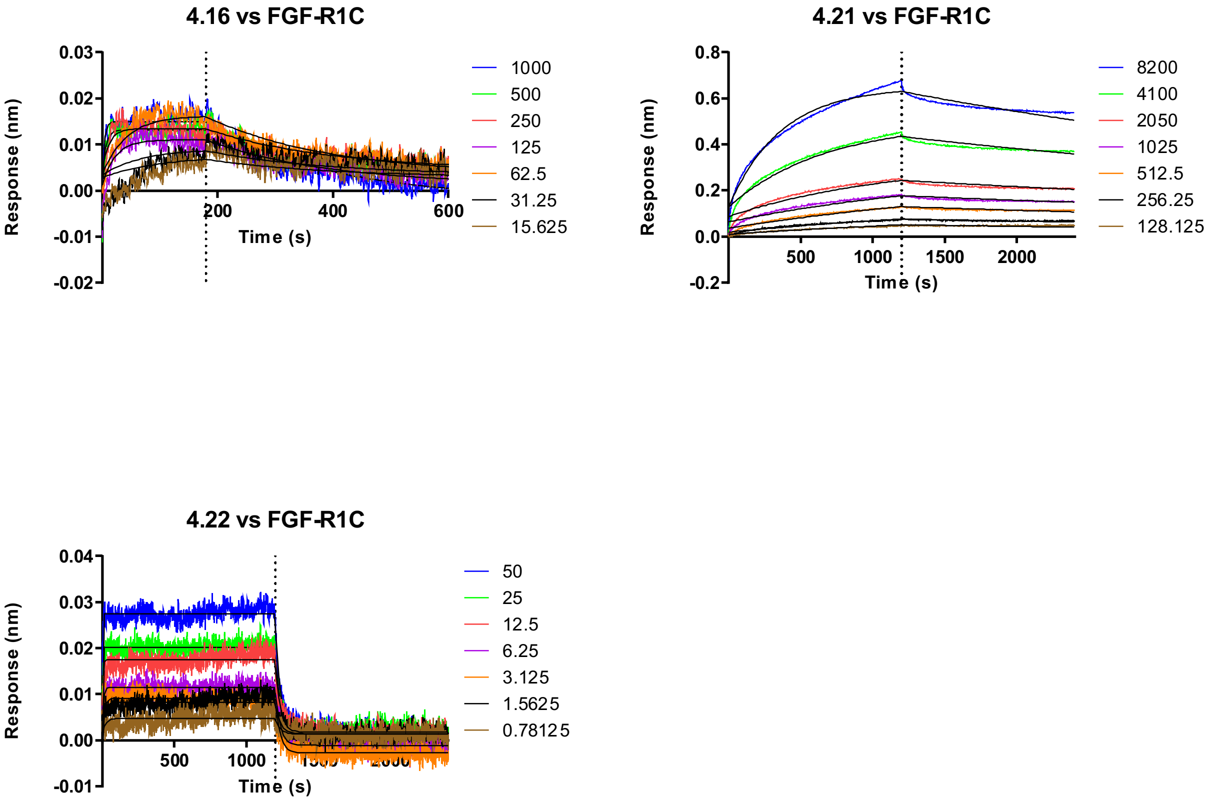

Figure S 22 Purified trimer-18 CP multi-concentration association and dissociation kinetics curves binding to FGF-R1C ECD. Data generated by Sartorius Octet RH16. Concentrations shown in nM.

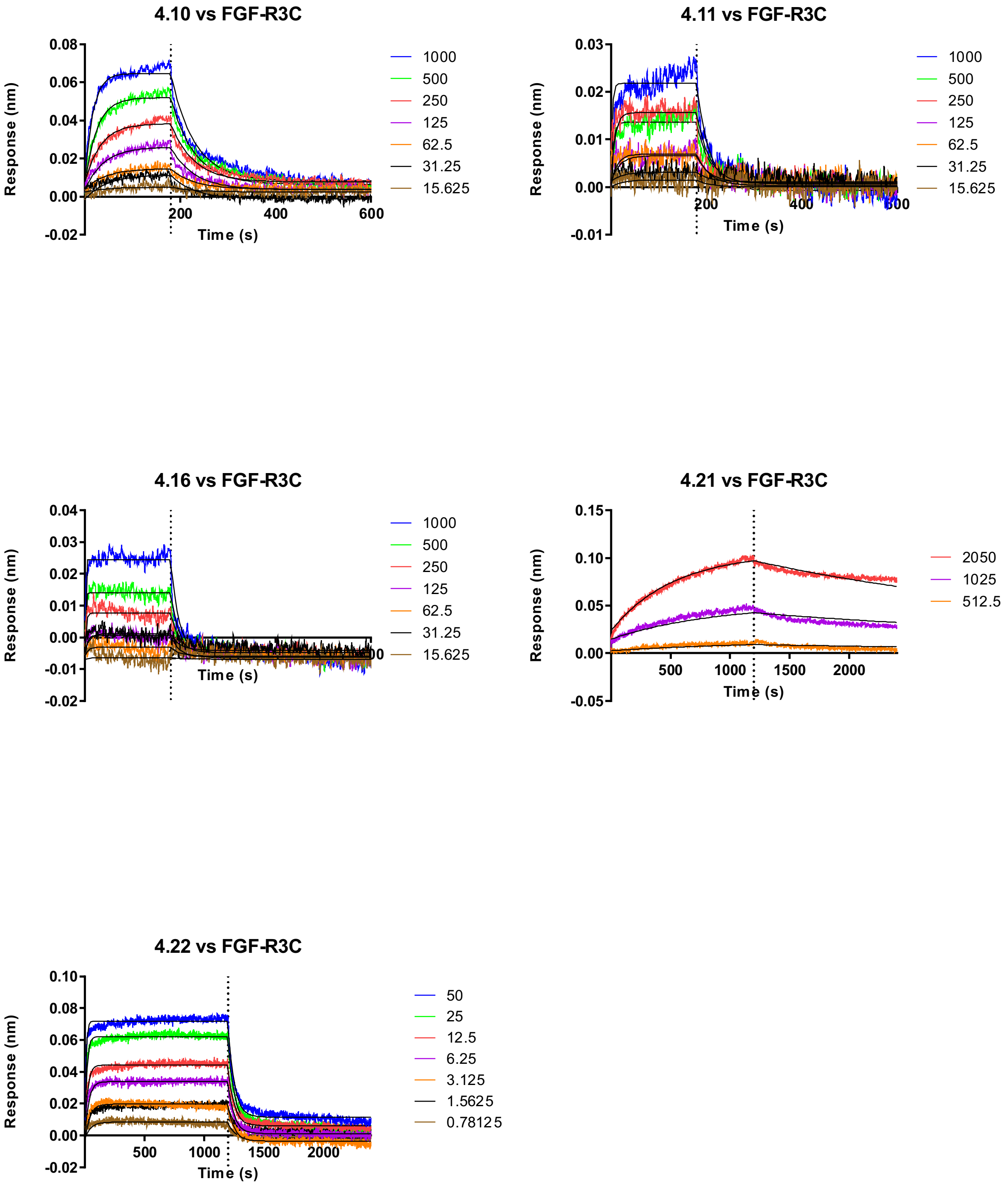

Figure S 23 Purified trimer-18 CP multi-concentration association and dissociation kinetics curves binding to FGF-R3C ECD. Data generated by Sartorius Octet RH16. Concentrations shown in nM

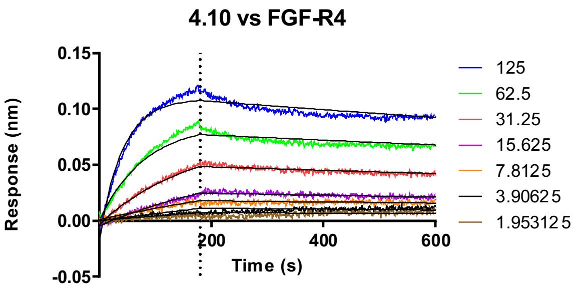
4.10 vs FGF-R4
Response (nm)
0.15
0.10
0.05
0.00
-0.05
200
400
600
Time (s)
125
62.5
31.25
15.625
7.8125
3.90625
1.953125

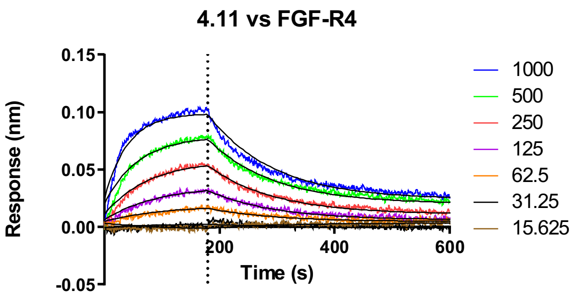
4.11 vs FGF-R4
Response (nm)
0.15
0.10
0.05
0.00
-0.05
200
400
600
Time (s)
1000
500
250
125
62.5
31.25
15.625

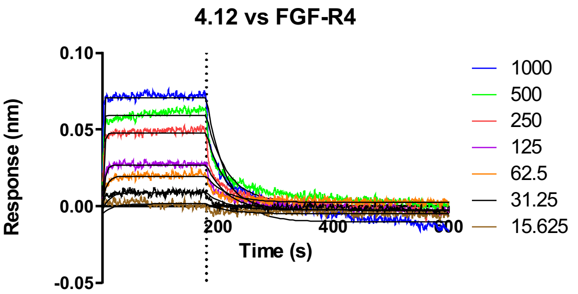
4.12 vs FGF-R4
Response (nm)
0.10
0.05
0.00
-0.05
200
400
600
Time (s)
1000
500
250
125
62.5
31.25
15.625

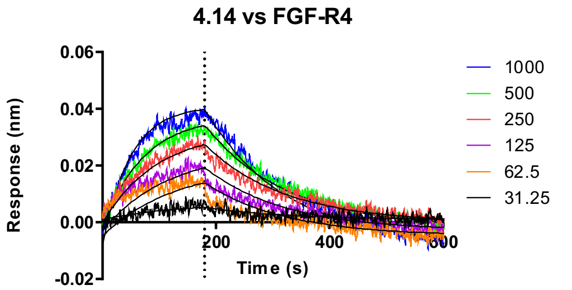
4.14 vs FGF-R4
Response (nm)
0.06
0.04
0.02
0.00
-0.02
200
400
600
Time (s)
1000
500
250
125
62.5
31.25

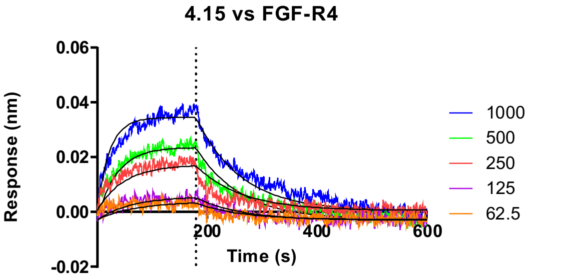
4.15 vs FGF-R4
Response (nm)
0.06
0.04
0.02
0.00
-0.02
200
400
600
Time (s)
1000
500
250
125
62.5

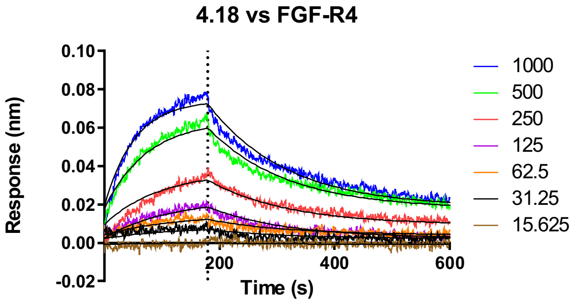
4.18 vs FGF-R4
Response (nm)
0.10
0.08
0.06
0.04
0.02
0.00
-0.02
200
400
600
Time (s)
1000
500
250
125
62.5
31.25
15.625

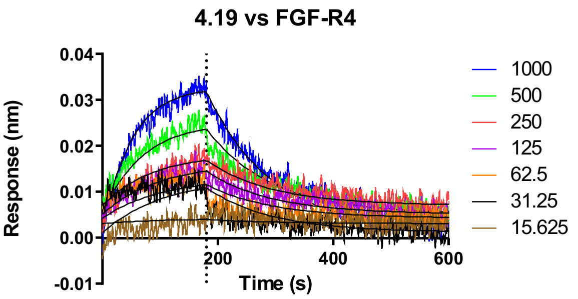
4.19 vs FGF-R4
Response (nm)
0.04
0.03
0.02
0.01
0.00
-0.01
200
400
600
Time (s)
1000
500
250
125
62.5
31.25
15.625

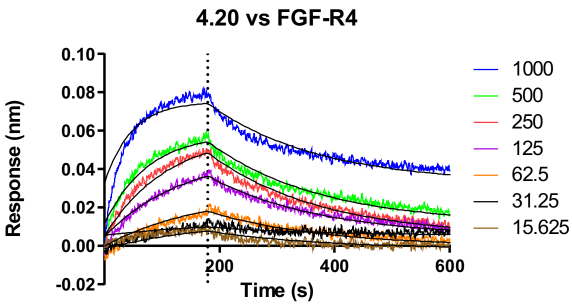
4.20 vs FGF-R4
Response (nm)
0.10
0.08
0.06
0.04
0.02
0.00
-0.02
200
400
600
Time (s)
1000
500
250
125
62.5
31.25
15.625

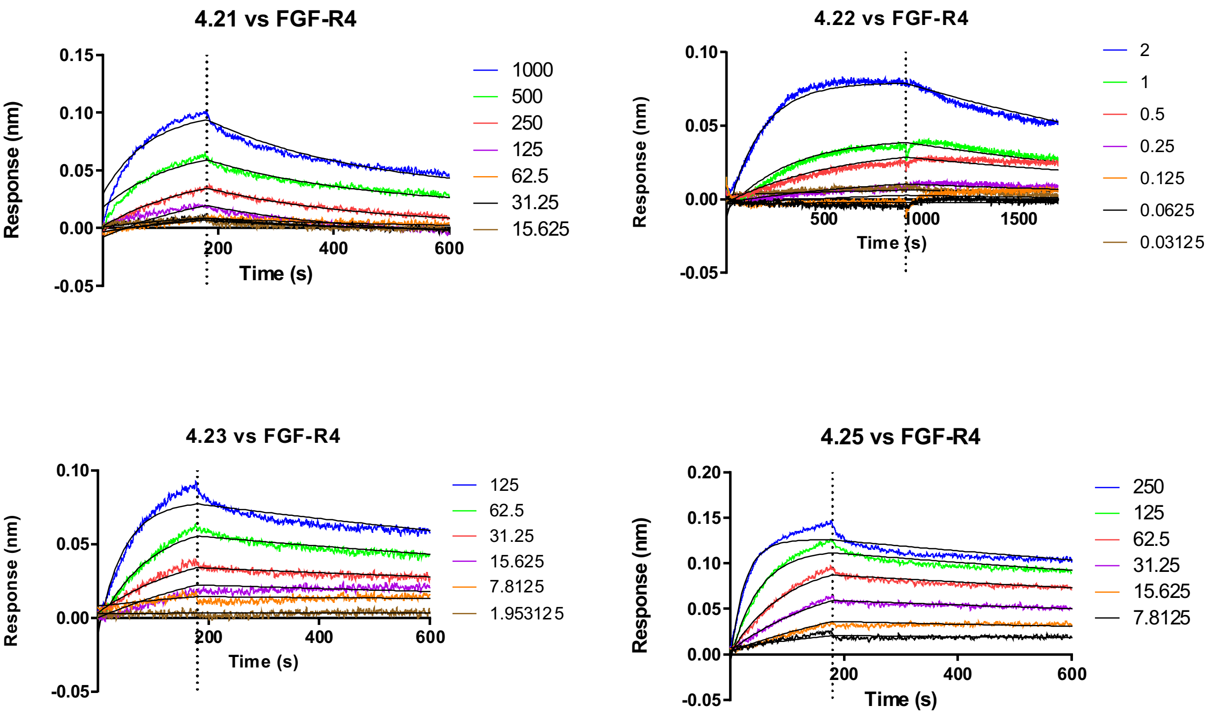

Figure S 24 Purified trimer-18 CP multi-concentration association and dissociation kinetics curves binding to FGF-R4 ECD. Data generated by Sartorius Octet RH16. Concentrations shown in nM

Alphascreen binding assay

The competitive binding assay was carried out according to the methods detailed in Hansen. et al using AlphaScreen.[3] All reagents and samples were prepared in assay buffer: 40mM Hepes, 140mM NaCl, 1mM CaCl2, 0,05% Tween20, pH=7.5, 0.1% BSA. In brief, recombinant human beta-klotho (R&D Systems) was preincubated for 60 min with the ectodomain, either Fc-fused human FGF-R1alpha(IIIc), FGF-R3alpha(IIIc) or FGF-R4 (R&D Systems), and the mixture was added to the test peptides in OptiPlate™ (PerkinElmer). Biotinylated FGF21 and heparin were further added to each well. AlphaScreen bead mix (1:1 mix of Protein A acceptor beads/ streptavidin-coated donor beads, PerkinElmer) were added to each well incubated overnight at RT in the dark, and measured on a plate reader (Envision). Assays were carried out in triplicates with concentrations ranging from 3, 10, 30, 100, 300 ,1000 and 3000 nM of peptide. No peptide control (media only) was used as a negative control, and unlabeled FGF21 and FGF1 as positive control. Data analysis was performed in Prism (GraphPad Software, Ver. 7.05, Figure S 25).

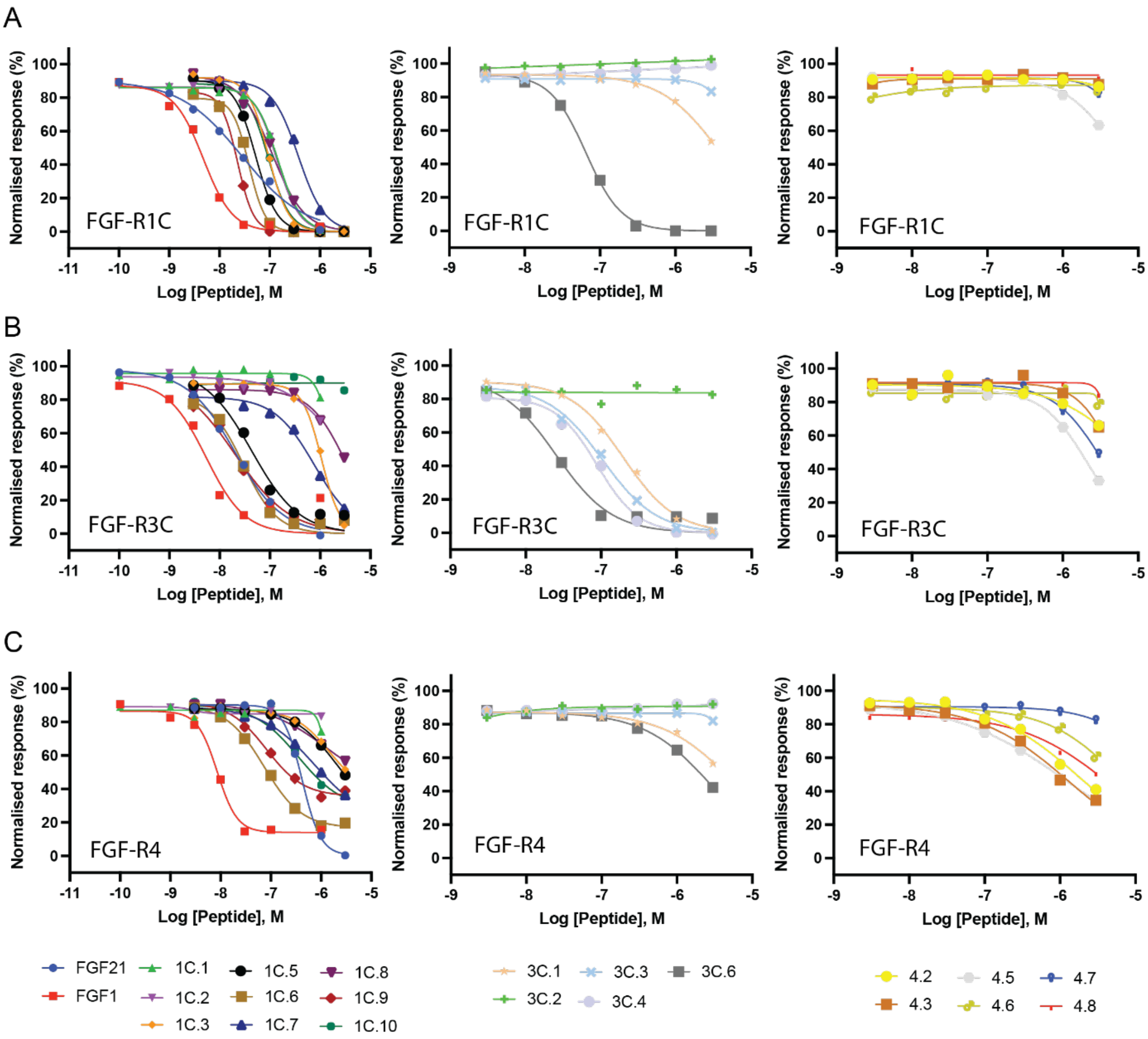

Figure S 25 Alpha screen displacement curves with purified peptides against recombinant human FGF-R Fc chimera proteins competing with biotinylated natural FGF21 ligand. A) Data against FGF-R alpha (IIIc) Fc chimera FGF-R1C, B) data against FGF-R3 (IIIc) Fc chimera FGF-R3C, C) data against FGF-R4 FGF-R4 Fc chimera proteins. IC_{50} values for controls: unlabelled FGF21 (IC50 = 23nM (FGF-R1C), 20nM (FGF-R3), 674 nM (FGFR-4), FGF1 (IC50 = 4 nM (FGF-R1C), 3 nM (FGF-R3C), 7 nM (FGF-R4).

Statistical analysis of peptide fitness for affinity prediction

Biochemical selectivity prediction: From the NGS data of mRNA display cross screens (detailed above), fitness values were calculated using an in-house python script to assess the relative enrichment of the peptides at the last round, based on the log-ratio of normalised reads counts of the cross screen round five selection as a fraction of normalised reads of the previous round selection against the original FGF-R target:

$$F_i = \log \frac{c_i^R}{N^R} \frac{N^{R-1}}{c_i^{R-1}}$$

Where F_i represents the fitness of peptides *i*, c^R_i refers to its reads counts and N^R the total number of peptides reads at round *R* (or *R-1*). The fitness values have been used as proxy for affinity prediction of the peptides.

Low fitness values (<0.1) show de-enrichment of the sequence against the new FGF-R target in the cross screen so was used to predict low affinity (>1000 nM K_D). High fitness values (>0.5) show high sequence enrichment against the new FGF-R target in the cross screen so was used to predict high affinity (<1000 nM K_D). Fitness values lying between 0.1 and 0.5 were assumed to be ambiguous and were not used to predict biochemical results. Fitness values were calculated for the purified NNK peptides for which both K_D (multi-concentration BLI) and IC_{50} (Alpha screen) data were present (24 peptides total). Fitness values were also calculated for original selections against the same target throughout all 5 rounds. These fitness values were then compared with the biochemical (K_D) data to evaluate success rate of fitness binding prediction. Success was obtained where fitness prediction matched biochemical data for each peptide (Fit <0.1 and K_D >1000 nM, or Fit >0.5 and K_D <1000 nM) and failure where fitness prediction did not match biochemical data for each peptide (Fit <0.1 and K_D <1000 nM, or Fit> 0.5 and K_D >1000 nM). Individual fitness value comparison for NNK purified peptides shown in heatmap (Figure S 26) made using Prism (GraphPad Software, Ver. 7.05). Overall results summarised as pie chart and violin plot using Prism (GraphPad Software, Ver. 7.05).

3C.3 SAR binding prediction: To predict SAR for peptide 3C.3 the relative abundance of each amino acid at each residue position was calculated using an in-house python script for all sequences present in the same cluster as 3C.3 (selection against FGF-R3C, total 3284 sequences). At each residue position, the occurrence of each amino acid was calculated relative to the fitness value (as above) of the sequence it is found in. A high relative occurrence (>200) was used to predict higher affinity binding to FGF-R3C, since this amino acid at this position was enriched more within the cluster, whereas a low relative occurrence (<200) was used to predict a lower affinity due to lower enrichment. A heatmap was then constructed of all values using Prism (GraphPad Software, Ver. 7.05).

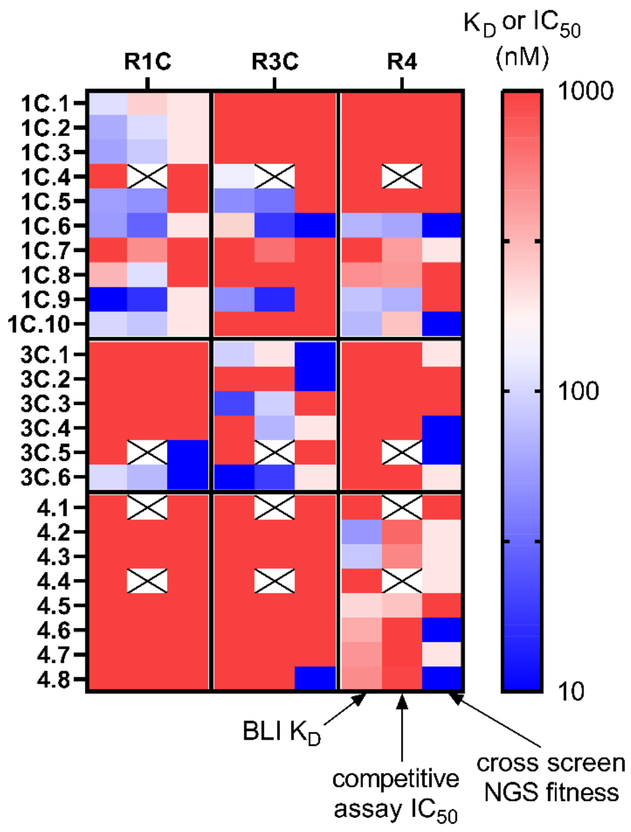

Figure S 26 heatmap showing cross screen fitness value prediction against individual peptide sequences. Columns are divided into three groups depending on whether data is for FGF-R1C, FGF-R3C, or FGF-R4. Rows depict individual sequences numbered according to the FGF-R target they were selected against. For each receptor, there are 3 sub-columns depicting multi-concentration BLI K_D (left), alpha screen IC_{50} (middle), and cross screen fitness binding prediction (right). K_D and IC_{50} affinity obtained using purified peptides and displayed on a log scale of high affinity (blue) to low affinity (red). Crosses denote where no IC_{50} data was determined. Cross screen NGS fitness values calculated as normalised read counts in cross screen round 5 as a fraction of normalised read counts in previous round. Fitness value >0.5 (Blue) was taken to predict binding to the receptor, fitness value <0.1 (red) was taken to predict poor binding to the receptor, and fitness between 0.1 and 0.5 was considered an ambiguous result and is shown in white. Where the colour of K_D/IC_{50} matches with the fitness prediction, then the prediction was considered successful.

Supporting datasets

Next generation sequencing: a complete list of peptide hits from each mRNA display campaign performed, including the number of reads for each round. NNK selections, trimer-18 selections and cross screens are included.

High throughput peptide synthesis compound identity: a complete list of the exact masses calculated and found for all peptides synthesised in 96 well plate format for high throughput BLI screening. All data obtained via LC-MS (Waters Acquity system with QDA detector, KDA3351, and TUV detector, F08UPT321M) using Waters Acquity UPLC BEH C18 column (130 Å, 1.7 µm, 2.1 mm x 50 mm). A 3 min linear gradient of 10% to 90% solvent B was run; solvent A: 0.05% TFA in water, solvent B: 0.05% TFA in MeCN.

Peptide KD: this dataset shows the K_D value of every peptide against each of the three receptors (FGF-R1C, FGF-R3C, and FGF-R4) including the purity state, origin of the peptide, target originally selected against, and type of library used during selection.

Statistical 3C.3 SAR prediction input dataset: contains all peptide sequences, along with NGS read count for each round in the NNK selection against FGF-R3C ECD belonging to the same cluster as peptide 3C.3. This dataset was used as the basis for SAR prediction heatmaps.

References

1. A. Kawamura, M. Munzel, T. Kojima, C. Yapp, B. Bhushan, Y. Goto, A. Tumber, T. Katoh, O. N. King, T. Passioura, L. J. Walport, S. B. Hatch, S. Madden, S. Muller, P. E. Brennan, R. Chowdhury, R. J. Hopkinson, H. Suga and C. J. Schofield, *Nat Commun*, 2017, **8**, 14773.
2. B. Bhushan, D. Granata, C. S. Kaas, M. A. Kasimova, Q. Ren, C. N. Cramer, M. D. White, A. M. K. Hansen, C. Fledelius, G. Invernizzi, K. Deibler, O. D. Coleman, X. Zhao, X. Qu, H. Liu, S. S. Zurmuhl, J. T. Kodra, A. Kawamura and M. Munzel, *Chem Sci*, 2022, **13**, 3256-3262.
3. A. M. K. Hansen, S. G. Vienberg, K. Lykkegaard, X. Zhao, G. Tingqing, D. Han, X. Zhang, H. Thogersen, K. Sass-Orum, T. Tagmose, K. Raun and B. Andersen, *Biochem J*, 2018, **475**, 2985-2996.
