# Supplementary material for: Identification and engineering of potent cyclic peptides with selective or promiscuous binding through biochemical profiling and bioinformatic data analysis
Source: RSC Chem Biol. 2023 Nov 14;5(1):12–8. doi: 10.1039/d3cb00168g (PMC10763615; doi:10.1039/d3cb00168g)
Supplement: CB-005-D3CB00168G-s002 [file CB-005-D3CB00168G-s002.pdf]

Electronic Supplementary Material (ESI) for RSC Chemical Biology.
This journal is © The Royal Society of Chemistry 2023

	Calculated mass					
Sequence (NNK HT screen peptides)	[M+H]/1	[M+2H]/2	[M+3H]/3	[M+4H]/4	[M+5H]/5	Found mass
Z M C W W F M P E L V L K CGSGSDYKDDDDK	3009.4	1505.2	1003.8	753.1	602.7	1004.1
Z M C F W R F F P A L L A CGSGSDYKDDDDK	2928.3	1464.7	976.8	732.8	586.5	976.7
Z M C I K M F L A H K R F CGSGSDYKDDDDK	2951.4	1476.2	984.5	738.6	591.1	984.2
Z M C D A H R L L F E L I CGSGSDYKDDDDK	2887.2	1444.1	963.1	722.5	578.2	962.9
Z M C D C H P L L C F L L CGSGSDYKDDDDK	2834.2	1417.6	945.4	709.3	567.6	945.2
Z M C S C F P P L C F L I CGSGSDYKDDDDK	2800.2	1400.6	934.1	700.8	560.8	131.4
Z M C D C L H V V C F L V CGSGSDYKDDDDK	2808.2	1404.6	936.7	702.8	562.4	200.1
Z M C H C F P T I C W L V CGSGSDYKDDDDK	2879.2	1440.1	960.4	720.6	576.6	960.4
Z M M V K C W L A Y I R T CGSGSDYKDDDDK	2941.3	1471.2	981.1	736.1	589.1	980.6
Z M C V K M W L A Y I R T CGSGSDYKDDDDK	2941.3	1471.2	981.1	736.1	589.1	122.9
Z M C V K C W L A Y I R T MGSGSDYKDDDDK	2941.3	1471.2	981.1	736.1	589.1	112.8
Z M C S H M W L F K V L L CGSGSDYKDDDDK	2934.4	1467.7	978.8	734.3	587.7	130.3
Z M M S H C W L F K V L L CGSGSDYKDDDDK	2934.4	1467.7	978.8	734.3	587.7	129.1
Z M C S H C W L F K V L L MGSGSDYKDDDDK	2934.4	1467.7	978.8	734.3	587.7	113.1
Z M C H C F P T I C W L E CGSGSDYKDDDDK	2909.2	1455.1	970.4	728.1	582.6	969.6
Z M C I Y R L S D F W V V CGSGSDYKDDDDK	2958.3	1479.6	986.8	740.3	592.5	986.9
Z M C R D F W H L W F R V CGSGSDYKDDDDK	3122.4	1561.7	1041.5	781.4	625.3	1041.2
Z M C D V L L Y K L F R Y CGSGSDYKDDDDK	2990.3	1495.7	997.4	748.3	598.9	997.3
Z M C D V L V Y K V L W A CGSGSDYKDDDDK	2866.2	1433.6	956.1	717.3	574.0	956.3
Z M C F W P I W Y K F M A CGSGSDYKDDDDK	3049.4	1525.2	1017.1	763.1	610.7	131.0
Z M C F V T L Y K L L K Y CGSGSDYKDDDDK	2948.3	1474.7	983.4	737.8	590.5	983.6
Z M C E R F V T Y Y K L F CGSGSDYKDDDDK	3026.3	1513.7	1009.4	757.3	606.1	1009.4
Z M C A P K W L I K V L S CGSGSDYKDDDDK	2815.2	1408.1	939.1	704.6	563.8	938.7
Z M C P K W L I K V L L N CGSGSDYKDDDDK	2884.3	1442.7	962.1	721.8	577.7	962.0
Z M C N I I L Y K I M K N R CGSGSDYKDDDDK	3066.5	1533.8	1022.8	767.4	614.1	1022.9
Z M C L V S I Y K I M R T CGSGSDYKDDDDK	2884.3	1442.6	962.1	721.8	577.7	962.2
Z M C S I T I Y K L L R F CGSGSDYKDDDDK	2914.3	1457.6	972.1	729.3	583.7	972.6
Z M C E P W I T I Y K V L K K CGSGSDYKDDDDK	3178.6	1589.8	1060.2	795.4	636.5	1059.8
Z M C H I T I Y K I L R S CGSGSDYKDDDDK	2904.3	1452.6	968.8	726.8	581.7	968.4
Z M C Q F T Y S K F F F CGSGSDYKDDDDK	2875.1	1438.1	959.0	719.5	575.8	958.6
Z M C P V I I Y K I L S H CGSGSDYKDDDDK	2843.2	1422.1	948.4	711.6	569.4	948.4
Z M C F A L L Y K I L S R CGSGSDYKDDDDK	2884.3	1442.6	962.1	721.8	577.7	114.1
Z M C Y K L F L L W K Q W CGSGSDYKDDDDK	3085.5	1543.2	1029.2	772.1	617.9	131.1
Z M C P K W L L K W F L A CGSGSDYKDDDDK	2962.4	1481.7	988.1	741.3	593.3	988.5
Z M C V K Y F T L W K N W CGSGSDYKDDDDK	3045.4	1523.2	1015.8	762.1	609.9	1016.4
Z M C S C P F P L C E L M CGSGSDYKDDDDK	2800.2	1400.6	934.1	700.8	560.8	875.4
Z M C S I L L Y K V L S Q CGSGSDYKDDDDK	2824.2	1412.6	942.1	706.8	565.6	941.9
Z M C I Y R L S D F W A V CGSGSDYKDDDDK	2930.2	1465.6	977.4	733.3	586.8	977.1
Z M C Y K L F L L W K Q CGSGSDYKDDDDK	2899.3	1450.1	967.1	725.6	580.7	200.1
Z M C G V L L Y K V M R V CGSGSDYKDDDDK	2838.3	1419.6	946.8	710.3	568.5	946.6
Z M C Y L Y R F A V F CGSGSDYKDDDDK	2739.0	1370.0	913.7	685.5	548.6	914.5
Z M C I A I Y K L M K M H CGSGSDYKDDDDK	2908.4	1454.7	970.1	727.8	582.5	1163.8
Z M C N I I L Y K I M K N R CGSGSDYKDDDDK	3066.5	1533.8	1022.8	767.4	614.1	1023.1
Z M C N I I L R CGSGSDYKDDDDK	2288.5	1144.8	763.5	572.9	458.5	763.3
Z M C N I L L Y K I L S V CGSGSDYKDDDDK	2836.2	1418.6	946.1	709.8	568.0	945.9
Z M C N V T I W K V L R L CGSGSDYKDDDDK	2902.3	1451.6	968.1	726.3	581.3	967.6
Z M C V W P K W L T K L I CGSGSDYKDDDDK	2944.4	1472.7	982.1	736.8	589.7	982.2
Z M C P K Y F L L W R L M T CGSGSDYKDDDDK	3128.6	1564.8	1043.5	782.9	626.5	1043.7
Z M C P R W I Y Y Y M L H CGSGSDYKDDDDK	3102.5	1551.7	1034.8	776.4	621.3	1034.5
Z M C D W P R W L V K V V CGSGSDYKDDDDK	2958.3	1479.7	986.8	740.3	592.5	986.2
Z M C P R Y F W Y H Y L L CGSGSDYKDDDDK	3118.4	1559.7	1040.1	780.4	624.5	1040.2
Z M C Q F R F S K F W L S CGSGSDYKDDDDK	3006.3	1503.7	1002.8	752.3	602.1	1003.1
Z M C I Y R L S D F W V E CGSGSDYKDDDDK	2988.2	1494.6	996.7	747.8	598.4	996.8
Z M C H Y I S D W L CGSGSDYKDDDDK	2593.8	1297.4	865.3	649.2	519.6	864.9
Z M C G H F S I T W F K W CGSGSDYKDDDDK	2969.2	1485.1	990.4	743.1	594.6	990.4
Z M C V F W T G M S I W I CGSGSDYKDDDDK	2900.2	1450.6	967.4	725.8	580.8	200.2
Z M C H I T I Y K I L R Y CGSGSDYKDDDDK	2980.3	1490.7	994.1	745.8	596.9	993.6
Z M C W V K Y W T V Y K Q CGSGSDYKDDDDK	3061.4	1531.2	1021.1	766.1	613.1	1020.9
Z M C H C F P I V C W F E CGSGSDYKDDDDK	2941.3	1471.1	981.1	736.1	589.1	980.1
Z M C D C V W P L C W M T CGSGSDYKDDDDK	2914.3	1457.6	972.1	729.3	583.7	131.3
Z M C V T W Y K V T K L R CGSGSDYKDDDDK	2954.3	1477.6	985.4	739.3	591.7	985.4
Z M C I K T F L L W R S S CGSGSDYKDDDDK	2911.3	1456.1	971.1	728.6	583.1	971.2
Z M C H V I L W R S W R Y CGSGSDYKDDDDK	3076.4	1538.7	1026.1	769.9	616.1	1026.2
Z M C P V L I Y K L W R T CGSGSDYKDDDDK	2949.3	1475.2	983.8	738.1	590.7	1179.4
Z M C I H R L S D F W V V CGSGSDYKDDDDK	2932.2	1466.6	978.1	733.8	587.2	978.1
Z M C Y K L F L L W K Q R CGSGSDYKDDDDK	3055.5	1528.2	1019.2	764.6	611.9	1019.2
Z M C S I L L Y K V L S R CGSGSDYKDDDDK	2852.2	1426.6	951.4	713.8	571.2	951.5
Z M C L I S I Y K V L K H CGSGSDYKDDDDK	2874.3	1437.6	958.8	719.3	575.7	958.6

Z M C W I T L Y K V L H A CGSGSDYKDDDDK	2904.2	1452.6	968.7	726.8	581.6	969.2
Z M C I L L Y K V L R G R CGSGSDYKDDDDK	2891.3	1446.2	964.4	723.6	579.1	964.5
Z M C F A S L W Y R V Q C CGSGSDYKDDDDK	2933.2	1467.1	978.4	734.1	587.4	159.0
Z M C H L A Y Y L A F R S CGSGSDYKDDDDK	2901.2	1451.1	967.7	726.0	581.0	967.9
Z M C V A F Y R A F M A S CGSGSDYKDDDDK	2823.1	1412.1	941.7	706.5	565.4	942.1
Z M C V S Y Y R A Y F A H CGSGSDYKDDDDK	2937.2	1469.1	979.7	735.0	588.2	979.7
Z M C K K H L L Y Y L I A CGSGSDYKDDDDK	2922.3	1461.7	974.8	731.3	585.3	975.2
Z M C L A Y Y L A F H S L CGSGSDYKDDDDK	2858.1	1429.6	953.4	715.3	572.4	1143.5
Z M C R E L L Y Y L L Q R CGSGSDYKDDDDK	3027.4	1514.2	1009.8	757.6	606.3	1211.2
Z M C V K Y Y L I M K S L CGSGSDYKDDDDK	2918.3	1459.7	973.4	730.3	584.5	973.2
Z M C S T R F V N L F W L CGSGSDYKDDDDK	2943.3	1472.1	981.8	736.6	589.5	981.7
Z M C V P C F S H T F L C CGSGSDYKDDDDK	2814.1	1407.6	938.7	704.3	563.6	938.1
Z M C W Y C F R K S F W C CGSGSDYKDDDDK	3086.4	1543.7	1029.5	772.4	618.1	1029.0
Z M C Y R F P S S H L F W A CGSGSDYKDDDDK	3071.4	1536.2	1024.5	768.6	615.1	1024.5
Z M C I R W P A R Q S L F W L CGSGSDYKDDDDK	3233.6	1617.3	1078.5	809.2	647.5	1078.8
Z M C F R W R L F H H L T P Y CGSGSDYKDDDDK	3333.7	1667.4	1111.9	834.2	667.5	1111.6
Z M C S N M W K L A N R W W A CGSGSDYKDDDDK	3223.6	1612.3	1075.2	806.6	645.5	1074.9
Z M C P E R L F Y W L L V CGSGSDYKDDDDK	2996.4	1498.7	999.5	749.8	600.1	999.0
Z M C W P I L W K V L S A CGSGSDYKDDDDK	2873.2	1437.1	958.4	719.1	575.4	958.2
Z M C I F D L F L P F I F CGSGSDYKDDDDK	2932.3	1466.7	978.1	733.8	587.3	977.7
Z M C H Y C F N K L F L C CGSGSDYKDDDDK	2948.3	1474.7	983.4	737.8	590.5	982.4
Z M C Q Q W N L Y T R H W H S CGSGSDYKDDDDK	3316.5	1658.8	1106.2	829.9	664.1	1105.9
Z M C H P T R L H L W L C C N CGSGSDYKDDDDK	3153.5	1577.3	1051.8	789.1	631.5	1051.7
Z M C F K H P W F F A W L CGSGSDYKDDDDK	3039.4	1520.2	1013.8	760.6	608.7	1013.9
MCSNMWKLANRWWACGSGSDYKDDDDK	3181.5	1591.3	1061.2	796.1	637.1	1061.1
MCIRWPARQSLFWLCGSGSDYKDDDDK	3191.6	1596.3	1064.5	798.6	639.1	1064.4
MCLFDLHLPFIFCGSGSDYKDDDDK	2880.3	1440.6	960.8	720.8	576.9	960.9
MCIFDNWLPFIYCGSGSDYKDDDDK	2946.3	1473.6	982.8	737.3	590.1	982.8
MCYFDPHFIFIPCGSGSDYKDDDDK	2914.2	1457.6	972.1	729.3	583.6	972.4
MCFFCFDRLFLCCGSGSDYKDDDDK	2929.3	1465.2	977.1	733.1	586.7	1244.7
MCVFDSFLPFIICGSGSDYKDDDDK	2816.2	1408.6	939.4	704.8	564.0	939.5
MCFYCFDSLFLCCGSGSDYKDDDDK	2876.2	1438.6	959.4	719.8	576.0	958.5
MCVFDSWLPFVYCGSGSDYKDDDDK	2891.2	1446.1	964.4	723.5	579.0	964.3
MCIFDTHMPFIPCGSGSDYKDDDDK	2836.2	1418.6	946.1	709.8	568.0	945.8
MCIFDTFLPFIFCGSGSDYKDDDDK	2878.2	1439.6	960.1	720.3	576.4	960.0
MCFVCFRHSFWCCGSGSDYKDDDDK	2950.3	1475.6	984.1	738.3	590.9	113.2
MCCYNLFSLFWACGSGSDYKDDDDK	2882.2	1441.6	961.4	721.3	577.2	240.5
MCIRWPARQSLFWICGSGSDYKDDDDK	3191.6	1596.3	1064.5	798.6	639.1	1064.4
MCRLCFSKLFWYCGSGSDYKDDDDK	2981.4	1491.2	994.5	746.1	597.1	239.2
MCLLWTRHFDRYWVCGSGSDYKDDDDK	3310.7	1655.8	1104.2	828.4	662.9	1104.3
MCFRWRLFHHLTPYCGSGSDYKDDDDK	3291.7	1646.3	1097.9	823.7	659.1	1097.9
MCIFDIHMPFIPCGSGSDYKDDDDK	2848.2	1424.6	950.1	712.8	570.4	950.0
MCYRHPHSNPLFWACGSGSDYKDDDDK	3143.4	1572.2	1048.5	786.6	629.5	1048.4
MCIGKWYTYQYWTRCGSGSDYKDDDDK	3283.6	1642.3	1095.2	821.6	657.5	1095.4
MCYKWPIRSHLYWLCGSGSDYKDDDDK	3280.7	1640.8	1094.2	820.9	656.9	1094.3
MCIYRLSDFWVVCGSGSDYKDDDDK	2916.2	1458.6	972.7	729.8	584.0	1247.6
MCHPTRLHLWLCCNCGSGSDYKDDDDK	3111.5	1556.2	1037.8	778.6	623.1	113.2
MCHYCFNKLFLCCGSGSDYKDDDDK	2906.3	1453.6	969.4	727.3	582.1	138.8
MCVRWPNLNALFWLCGSGSDYKDDDDK	3147.5	1574.3	1049.8	787.6	630.3	1163.1
MCQQWNLYTRHWHSCGSGSDYKDDDDK	3274.5	1637.7	1092.2	819.4	655.7	1092.1
MCIFDNWLPFIFCGSGSDYKDDDDK	2930.3	1465.6	977.4	733.3	586.9	977.4
MCVFDSFLPFVLCGSGSDYKDDDDK	2802.1	1401.6	934.7	701.3	561.2	934.8
MCTKFPWPLPIFWACGSGSDYKDDDDK	3121.5	1561.3	1041.2	781.1	625.1	1041.3
MCIRWPARQPLFWLCGSGSDYKDDDDK	3201.6	1601.3	1067.9	801.2	641.1	1067.9
MCQQWNLYTRHWHICGSGSDYKDDDDK	3300.6	1650.8	1100.9	825.9	660.9	1100.7
MCCFKFRDLFWVCGSGSDYKDDDDK	2979.4	1490.2	993.8	745.6	596.7	102.3
MCFVCFRHSFLCCGSGSDYKDDDDK	2877.2	1439.1	959.7	720.1	576.2	113.1
MCWYCFRKSFWCCGSGSDYKDDDDK	3044.4	1522.7	1015.5	761.8	609.7	113.1
MCFVYPRRQPLYWICGSGSDYKDDDDK	3256.7	1628.8	1086.2	814.9	652.1	1086.5
MCLFDSFVPFIFCGSGSDYKDDDDK	2850.2	1425.6	950.7	713.3	570.8	950.9
MCYVCFSHSFICCGSGSDYKDDDDK	2824.1	1412.6	942.0	706.8	565.6	157.5
MCVPCFSHTFLCCGSGSDYKDDDDK	2772.1	1386.5	924.7	693.8	555.2	924.1
MCRLCFSKLFWCCGSGSDYKDDDDK	2921.3	1461.2	974.4	731.1	585.1	990.6
MCCYRCWSLFWCCGSGSDYKDDDDK	2985.3	1493.2	995.8	747.1	597.9	1040.8
MCVFDSWLPFVFCGSGSDYKDDDDK	2875.2	1438.1	959.1	719.5	575.8	959.2
MCSQSWVLYELWRVCGSGSDYKDDDDK	3184.5	1592.7	1062.2	796.9	637.7	1063.1
MCSYCFNKLFLCCGSGSDYKDDDDK	2856.2	1428.6	952.7	714.8	572.0	954.0
MCTFDLFLPFIFCGSGSDYKDDDDK	2878.2	1439.6	960.1	720.3	576.4	960.0
MCTFDTHFPFITCGSGSDYKDDDDK	2844.1	1422.5	948.7	711.8	569.6	948.9
MCTKFPWPFPIFWACGSGSDYKDDDDK	3155.6	1578.3	1052.5	789.6	631.9	1052.4

MCFRWRLFHHLTPFCGSGSDYKDDDDK	3275.7	1638.3	1092.6	819.7	655.9	1092.7
MCCTRFVNLFWLCGSGSDYKDDDDK	2917.3	1459.1	973.1	730.1	584.3	129.4
MCVYDIFVPFIICGSGSDYKDDDDK	2844.2	1422.6	948.7	711.8	569.6	948.7
MCVYDLFLPFIYCGSGSDYKDDDDK	2908.2	1454.6	970.1	727.8	582.4	969.9
MCIWDSFVPFILCGSGSDYKDDDDK	2855.2	1428.1	952.4	714.5	571.8	952.4
MCWSLYRLFWACGSGSDYKDDDDK	2860.2	1430.6	954.1	715.8	572.8	113.2
MCLYDSFLPFIFCGSGSDYKDDDDK	2880.2	1440.6	960.7	720.8	576.8	960.9
MCTRTSFYGFWYYACGSGSDYKDDDDK	3180.4	1590.7	1060.8	795.9	636.9	239.2
MCSYGSWYGYSYLACGSGSDYKDDDDK	3035.2	1518.1	1012.4	759.5	607.8	1012.4
MCILHGLIYWAPKQCGSGSDYKDDDDK	3057.4	1529.2	1019.8	765.1	612.3	1020.1
MCIFDIHMPFIPCGSGSDYKDDDDK	2848.2	1424.6	950.1	712.8	570.4	950.7
MCRLCFSKLFWCCGSGSDYKDDDDK	2921.3	1461.2	974.4	731.1	585.1	990.5
MCFVYPRRQPLYWICGSGSDYKDDDDK	3256.7	1628.8	1086.2	814.9	652.1	1086.0
MCFRWRLFHHLTPYCGSGSDYKDDDDK	3291.7	1646.3	1097.9	823.7	659.1	1097.9
MCIFDTFLPFIFCGSGSDYKDDDDK	2878.2	1439.6	960.1	720.3	576.4	960.4
MCIYRLSDFWVVCGSGSDYKDDDDK	2916.2	1458.6	972.7	729.8	584.0	1011.0
MCTFDLFLPFIFCGSGSDYKDDDDK	2878.2	1439.6	960.1	720.3	576.4	959.9
MCYVCFSHSFICCGSGSDYKDDDDK	2824.1	1412.6	942.0	706.8	565.6	131.4
MCSNMWKLANRWWACGSGSDYKDDDDK	3181.5	1591.3	1061.2	796.1	637.1	1061.3
MCIFDNWLPFIYCGSGSDYKDDDDK	2946.3	1473.6	982.8	737.3	590.1	982.7
MCTRTSFYGFWYYACGSGSDYKDDDDK	3180.4	1590.7	1060.8	795.9	636.9	239.2
MCYRHPHSNPLFWACGSGSDYKDDDDK	3143.4	1572.2	1048.5	786.6	629.5	1048.2
MCFRWRLFHHLTPYCGSGSDYKDDDDK	3291.7	1646.3	1097.9	823.7	659.1	1097.9
MCTKFPWPFPIFWACGSGSDYKDDDDK	3155.6	1578.3	1052.5	789.6	631.9	1052.7
MCFVCFRHSFLCCGSGSDYKDDDDK	2877.2	1439.1	959.7	720.1	576.2	113.2
MCFVCFRHSFLCCGSGSDYKDDDDK	2877.2	1439.1	959.7	720.1	576.2	113.1
MCLLWTRHFDRYWVCGSGSDYKDDDDK	3310.7	1655.8	1104.2	828.4	662.9	1104.4
MCIGKWYTYQYWTRCGSGSDYKDDDDK	3283.6	1642.3	1095.2	821.6	657.5	1094.9
MCVPCFSHTFLCCGSGSDYKDDDDK	2772.1	1386.5	924.7	693.8	555.2	924.2
MCIRWPARQPLFWLCGSGSDYKDDDDK	3201.6	1601.3	1067.9	801.2	641.1	1068.0
MCHPTRLHLWLCCNCGSGSDYKDDDDK	3111.5	1556.2	1037.8	778.6	623.1	113.1
MCYKCFTGLFLCCGSGSDYKDDDDK	2813.2	1407.1	938.4	704.0	563.4	1011.0
MCHFRPNKFTAWLPCGSGSDYKDDDDK	3132.5	1566.7	1044.8	783.9	627.3	1045.0
MCWSLYRLFGSGSDYKDDDDK	2501.8	1251.4	834.6	626.2	501.2	1132.5
MCSYGSWYGYSYLACGSGSDYKDDDDK	3035.2	1518.1	1012.4	759.5	607.8	1012.2
MCCYNLFSLFWCCGSGSDYKDDDDK	2914.2	1457.6	972.1	729.3	583.6	1164.4
MCIFELNLPFIPCGSGSDYKDDDDK	2821.2	1411.1	941.1	706.0	565.0	941.3
MCRFYELFWSFPCGSGSDYKDDDDK	3010.3	1505.7	1004.1	753.3	602.9	1074.4
ACSAKTTSACGSGSDYKDDDDK	2223.3	1112.1	741.8	556.6	445.5	741.8
ACSLNHTVNCGSGSDYKDDDDK	2342.4	1171.7	781.5	586.4	469.3	781.6
LPGRTCREYPDLWWVRCYGSGSDYKDDDDK	3594.9	1797.9	1199.0	899.5	719.8	1199.2
LEERPVGSGSDYKDDDDK	2025.1	1013.0	675.7	507.0	405.8	675.8
VYMSPFGSGSDYKDDDDK	2026.1	1013.6	676.0	507.3	406.0	676.2
LSPPRYPGSGSDYKDDDDK	2112.2	1056.6	704.7	528.8	423.2	704.9
MQLPLATGSGSDYKDDDDK	2056.2	1028.6	686.1	514.8	412.0	686.2
MCYKWPIRSHLYWLCGSGSDYKDDDDK	3280.7	1640.8	1094.2	820.9	656.9	1094.0

Sequence (T18 HT Screen peptides)	Calculated mass [M+H]/1	[M+2H]/2	[M+3H]/3	[M+4H]/4	[M+5H]/5	Found mass
ZMCREIRYWQLYRAFCGSGSDYKDDDDK	3361.7	1681.3	1121.2	841.2	673.1	1121.0
ZMCEHLFYYLLVKRQCGSGSDYKDDDDK	3269.6	1635.3	1090.5	818.2	654.7	1090.5
ZMCYKNISTGIFLYCGSGSDYKDDDDK	2979.3	1490.1	993.8	745.6	596.7	993.8
ZMCTTLYYYKLWRRCGSGSEEGSGSDYKDDDDK	3770.0	1885.5	1257.3	943.3	754.8	943.4
ZMCYYNFLNKVLVYCGSGSDYKDDDDK	3096.4	1548.7	1032.8	774.8	620.1	1032.9
ZMCKEYEILWYKLQYCGSGSDYKDDDDK	3336.6	1668.8	1112.9	834.9	668.1	1112.8
ZMCKHLYYYLLWKKCGSGSEEGSGSDYKDDDDK	3762.1	1881.5	1254.7	941.3	753.2	941.1
ZMCYKHILWYKIQKLCGSGSEEGSGSDYKDDDDK	3840.2	1920.6	1280.7	960.8	768.8	961.2
ZMCKRHIRYWQLYVCGSGSEEGSGSDYKDDDDK	3769.1	1885.0	1257.0	943.0	754.6	943.2
ZMCNLSIIHGYVAYICGSGSDYKDDDDK	3023.3	1512.2	1008.4	756.6	605.5	1008.4
ZMCNVLLYKVLRHCGSGSDYKDDDDK	2915.3	1458.1	972.4	729.6	583.9	972.6
ZMCYTVLWYKYQKKCGSGSEEGSGSDYKDDDDK	3727.0	1864.0	1243.0	932.5	746.2	1243.0
ZMCHIWYFNNQFHYVCGSGSDYKDDDDK	3328.6	1664.8	1110.2	832.9	666.5	1110.1
ZMCKRHLIYYKIARCGSGSDYKDDDDK	3121.5	1561.3	1041.2	781.1	625.1	1040.9
ZMCPIHFFKIVYRHCGSGSEEGSGSDYKDDDDK	3664.0	1832.5	1222.0	916.7	733.6	1221.9
ZMCTGRHLYYWKLFHCGSGSEEGSGSDYKDDDDK	3828.1	1914.5	1276.7	957.8	766.4	958.0
ZMCHRHLYYWRLFHSCGSGSEEGSGSDYKDDDDK	3922.2	1961.6	1308.1	981.3	785.2	981.4
ZMCAKEHLFYYLLWCGSGSDYKDDDDK	3143.5	1572.2	1048.5	786.6	629.5	1048.4
ZMCDSIRAQYARWWICGSGSDYKDDDDK	3225.5	1613.3	1075.8	807.1	645.9	1076.0
ZMCEWFYYHKSWYVYCGSGSDYKDDDDK	3431.7	1716.3	1144.6	858.7	687.1	1144.5
ZMCYHRHIFQNHWYKCGSGSEEGSGSDYKDDDDK	3936.1	1968.6	1312.7	984.8	788.0	984.8
ZMCWWFKQHEFWFKVCGSGSDYKDDDDK	3428.8	1714.9	1143.6	857.9	686.6	1143.5
ZMCRWVKYYQLVHRKCGSGSDYKDDDDK	3336.7	1668.9	1112.9	834.9	668.1	1112.8
ZMCSILWYKITKPLCGSGSDYKDDDDK	3022.4	1511.7	1008.1	756.4	605.3	1008.2
ZMCYKNIDNGVLVFCGSGSDYKDDDDK	2942.2	1471.6	981.4	736.3	589.2	981.4
ZMCKYATLQWYKWFYCGSGSDYKDDDDK	3357.7	1679.3	1119.9	840.2	672.3	1119.8
ZMCDWYVTIYKVLKKCGSGSDYKDDDDK	3216.6	1608.8	1072.9	804.9	644.1	1072.9
ZMCPYAIYWYKWRKTCGSGSEEGSGSDYKDDDDK	3882.2	1941.6	1294.7	971.3	777.2	971.4
ZMCAKYFLIWRAYQQCGSGSDYKDDDDK	3247.6	1624.3	1083.2	812.6	650.3	1083.1
ZMCHIRYWQLVRKHCGSGSEEGSGSDYKDDDDK	3743.0	1872.0	1248.3	936.5	749.4	1248.4
ZMCHRYIYLDYWHCGSGSDYKDDDDK	3126.4	1563.7	1042.8	782.3	626.1	1042.7
ZMCFLDHKTKVFVYCGSGSDYKDDDDK	3057.4	1529.2	1019.8	765.1	612.3	1019.8
ZMCLWYYLQFHRRECGSGSDYKDDDDK	3271.6	1636.3	1091.2	818.6	655.1	1091.2
ZMCESDAYRWKLWWVCGSGSDYKDDDDK	3299.6	1650.3	1100.5	825.6	660.7	1100.6
ZMCTYDFVLGHHYLICGSGSDYKDDDDK	3138.4	1569.7	1046.8	785.4	628.5	1046.8
ZMCYTRFYNWEKWQLCGSGSDYKDDDDK	3394.7	1697.8	1132.2	849.4	679.7	1132.3
ZMCEQRSRWSNYWYPCGSGSDYKDDDDK	3332.5	1666.8	1111.5	833.9	667.3	1111.4
ZMCTYEFLFEELYLHCGSGSDYKDDDDK	3264.5	1632.8	1088.8	816.9	653.7	1088.8
ZMCDKFVKWYIVFRHCGSGSEEGSGSDYKDDDDK	3845.2	1923.1	1282.4	962.0	769.8	962.0
ZMCYDIVFDYITVYHCGSGSDYKDDDDK	3208.4	1604.7	1070.1	802.9	642.5	1070.1
ZMCKIWYVDHKFWWVCGSGSDYKDDDDK	3367.7	1684.4	1123.2	842.7	674.3	1123.0
ZMCKREIKYWLVFCGSGSDYKDDDDK	3042.4	1521.7	1014.8	761.4	609.3	1014.8
ZMCYHRPPNSKIWRKCGSGSDYKDDDDK	3242.6	1621.8	1081.5	811.4	649.3	1081.4
ZMCDLYTVLWYKIQKECGSGSDYKDDDDK	3359.7	1680.3	1120.6	840.7	672.7	1120.6
ZMCEDLFYYLLFHHCGSGSDYKDDDDK	3157.4	1579.2	1053.1	790.1	632.3	1053.1
ZMCEREVLYWLLVTCGSGSDYKDDDDK	3081.4	1541.2	1027.8	771.1	617.1	1027.7
ZMCVWQYSHHYNWHCGSGSDYKDDDDK	3217.4	1609.2	1073.1	805.1	644.3	1073.0
ZMCEDLFYYIINRYGCGSGSDYKDDDDK	3226.5	1613.7	1076.2	807.4	646.1	1076.1
ZMCLWHYYKIFRRQKCGSGSDYKDDDDK	3398.8	1699.9	1133.6	850.4	680.6	1133.3
ZMCDAYYVRYWQVLHCGSGSDYKDDDDK	3273.5	1637.3	1091.8	819.1	655.5	1091.8
ZMCWSKLIIKYYLSQCGSGSDYKDDDDK	3202.6	1601.8	1068.2	801.4	641.3	1068.0
ZMCVWKYRLDRNWYCGSGSDYKDDDDK	3259.5	1630.3	1087.2	815.6	652.7	1087.1
ZMCYLDDFYSYRYWACGSGSDYKDDDDK	3322.5	1661.8	1108.2	831.4	665.3	1108.3
ZMCPYDYYDWHDWRDCGSGSDYKDDDDK	3391.5	1696.2	1131.2	848.6	679.1	1131.1
ZMCPYYVQYWRVFCGSGSDYKDDDDK	3081.4	1541.2	1027.8	771.1	617.1	1027.8
ZMCVWWSHPSIWICGSGSDYKDDDDK	2971.3	1486.1	991.1	743.6	595.1	991.1
ZMCEHRHPYGHWEQCGSGSDYKDDDDK	3136.3	1568.6	1046.1	784.8	628.1	1046.1
ZMCAVYYKLWLKHSRCGSGSEEGSGSDYKDDDDK	3771.1	1886.0	1257.7	943.5	755.0	943.4
ZMCYKYSKDLWLYRLWCGSGSDYKDDDDK	3494.9	1747.9	1165.6	874.5	699.8	1165.6
ZMCYNIYWYKYVRRECGSGSDYKDDDDK	3413.7	1707.3	1138.6	854.2	683.5	1138.5
ZMCTYVYYYKLFHRCGSGSDYKDDDDK	3213.5	1607.3	1071.8	804.1	643.5	1071.8
ZMCYEIIWYKLQRVCGSGSDYKDDDDK	3171.5	1586.3	1057.8	793.6	635.1	1057.7
ZMCIKDLHLFYYLLCGSGSDYKDDDDK	3098.5	1549.7	1033.5	775.4	620.5	1033.4
ZMCIRYLFYYLISHCGSGSDYKDDDDK	3148.5	1574.7	1050.2	787.9	630.5	1050.2
ZMCHLLYYKLFHSCGSGSDYKDDDDK	2981.3	1491.1	994.4	746.1	597.1	994.4
ZMCNVLLYKDLRHCGSGSDYKDDDDK	2931.2	1466.1	977.7	733.6	587.0	977.7
ZMCYTRFWTEEWQPCGSGSDYKDDDDK	3203.4	1602.2	1068.5	801.6	641.5	1068.4
ZMCRHLFYWKLWHQRCGSGSEEGSGSDYKDDDDK	3977.3	1989.1	1326.4	995.1	796.3	994.9
ZMCLRHLYEPYWHCGSGSDYKDDDDK	3074.3	1537.7	1025.4	769.3	615.7	1025.3

ZMCAKAFLLWKGKFCGSGSEEGSGSDYKDDDDK	3515.8	1758.4	1172.6	879.7	704.0	1171.9
ZMCYEDEIWLYKVLYCGSGSDYKDDDDK	3294.6	1647.8	1098.9	824.4	659.7	1098.8
ZMCEVIWYKIQWRKCGSGSDYKDDDDK	3209.6	1605.3	1070.5	803.1	642.7	1070.4
ZMCITRKNKWEEWRPCGSGSDYKDDDDK	3303.6	1652.3	1101.9	826.6	661.5	1101.8
ZMCVKYIKYWSVFHCGSGSDYKDDDDK	3130.5	1565.7	1044.2	783.4	626.9	1044.1
ZMCTHRHPYETLWHQCGSGSDYKDDDDK	3265.5	1633.2	1089.2	817.1	653.9	1089.2
ZMCWYDQHSFYWERLCGSGSDYKDDDDK	3390.6	1695.8	1130.9	848.4	678.9	1130.8
ZMCYTRHPYEALWHQCGSGSDYKDDDDK	3261.5	1631.2	1087.8	816.1	653.1	1087.9
ZMCREIRYWQLWQCGSGSDYKDDDDK	3138.4	1569.7	1046.8	785.4	628.5	1046.8
ZMCEVKYFLIWKRWCGSGSDYKDDDDK	3228.6	1614.8	1076.9	807.9	646.5	1076.8
ZMCIFDIHMPFIPCGSGSDYKDDDDK	2890.3	1445.6	964.1	723.3	578.9	964.2
ZMCFWHKRSDFWLQFCGSGSDYKDDDDK	3357.7	1679.3	1119.9	840.2	672.3	1119.8
ZMCNKHLYYYLLFKHCGSGSDYKDDDDK	3299.6	1650.3	1100.5	825.7	660.7	1100.8
ZMCFHIKANYGFVRVCGSGSDYKDDDDK	3111.4	1556.2	1037.8	778.6	623.1	1037.9
ZMCTDLYYYLIWQRYCGSGSDYKDDDDK	3357.6	1679.3	1119.9	840.2	672.3	1119.9
ZMCYNVHWYRWIRRRCGSGSDYKDDDDK	3465.8	1733.4	1155.9	867.2	694.0	1156.0
ZMCPRYVLYYKLFHHCGSGSDYKDDDDK	3296.7	1648.8	1099.6	824.9	660.1	1099.6
ZMCNDLFYYLLWRKACGSGSDYKDDDDK	3262.6	1631.8	1088.2	816.4	653.3	1088.0
ZMCDIVIYKILRSLCGSGSDYKDDDDK	2993.4	1497.2	998.5	749.1	599.5	998.8
ZMCYEVLWYKLQRKHCGSGSDYKDDDDK	3323.7	1662.3	1108.6	831.7	665.5	1108.6
ZMCDNRRWVRYYQIFCGSGSDYKDDDDK	3376.7	1688.8	1126.2	844.9	676.1	1126.2
ZMCWWFRNHKFWQQICGSGSDYKDDDDK	3436.8	1718.9	1146.3	859.9	688.2	1146.3
ZMCYNKFITLYKVLYCGSGSDYKDDDDK	3225.6	1613.3	1075.9	807.1	645.9	1075.8
ZMCKRHITYWKLWHFCGSGSEEGSGSDYKDDDDK	3922.2	1961.6	1308.1	981.3	785.2	981.5
ZMCKHLFYYKLIRKACGSGSDYKDDDDK	3240.7	1620.8	1080.9	810.9	648.9	1080.9
ZMCHDYIVFYKVLRHCGSGSDYKDDDDK	3250.6	1625.8	1084.2	813.4	650.9	1084.1
ZMCRRELYYYLLYSKCGSGSDYKDDDDK	3327.7	1664.3	1109.9	832.7	666.3	1109.9
ZMCHNKYLYYYLLYSCGSGSDYKDDDDK	3300.6	1650.8	1100.9	825.9	660.9	1100.9
ZMCVDLFYYKLFRKLCGSGSDYKDDDDK	3265.7	1633.3	1089.2	817.2	653.9	1089.2
ZMCENKKVLFYYLLWCGSGSDYKDDDDK	3276.6	1638.8	1092.9	819.9	656.1	1092.9
ZMCYKRDLYYYLVFKCGSGSDYKDDDDK	3331.7	1666.3	1111.2	833.7	667.1	1111.1
ZMCYTELWYKYIRRHCGSGSDYKDDDDK	3388.7	1694.8	1130.2	847.9	678.5	1130.0
ZMCYDVLWYRYRYKQCGSGSDYKDDDDK	3413.7	1707.4	1138.6	854.2	683.5	1138.5
ZMCDLFYYLQHHKKVCGSGSDYKDDDDK	3251.6	1626.3	1084.5	813.6	651.1	1084.5
ZMCDKRHLYYYLLVCGSGSDYKDDDDK	3143.5	1572.2	1048.5	786.6	629.5	1048.5
ZMCHDKFITLYKVLYCGSGSDYKDDDDK	3200.6	1600.8	1067.5	800.9	640.9	1067.4
ZMCDRLILYYRIVHKCGSGSDYKDDDDK	3249.6	1625.3	1083.9	813.2	650.7	1083.8
ZMCPYEIKYWIVWQQCGSGSDYKDDDDK	3313.6	1657.3	1105.2	829.2	663.5	1105.3
ZMCYVQYYRVWRSICGSGSDYKDDDDK	3193.5	1597.2	1065.2	799.1	639.5	1065.1
ZMCDLKRELFYYLLVCGSGSDYKDDDDK	3232.6	1616.8	1078.2	808.9	647.3	1078.1
ZMCPYTKYWIVWRHSCGSGSDYKDDDDK	3296.6	1648.8	1099.5	824.9	660.1	1099.4
ZMCDKYWVKYWIAWRCGSGSDYKDDDDK	3374.7	1687.9	1125.6	844.4	675.7	1125.5
ZMCFWKKLHVFWVKDCGSGSDYKDDDDK	3293.7	1647.3	1098.6	824.2	659.5	1098.5
ZMCEHLYYYLLSHRRCGSGSDYKDDDDK	3310.6	1655.8	1104.2	828.4	662.9	1104.0
ZMCDHKRHLYYYILWCGSGSDYKDDDDK	3367.7	1684.3	1123.2	842.7	674.3	1123.2
ZMCEHRHPYSNFWILCGSGSDYKDDDDK	3259.5	1630.3	1087.2	815.6	652.7	1087.1
ZMCRELFYYRFWQKTCGSGSDYKDDDDK	3397.7	1699.4	1133.2	850.2	680.3	1133.1
ZMCTYTYVFNQTYVHCGSGSDYKDDDDK	3196.4	1598.7	1066.1	799.8	640.1	1066.1
ZMCEREFLYYYLYYKCGSGSDYKDDDDK	3410.7	1705.8	1137.6	853.4	682.9	1137.3
ZMCWYVQHSFYWERLCGSGSDYKDDDDK	3374.6	1687.8	1125.5	844.4	675.7	1125.3
ZMCKIIWLYKAARRFCGSGSDYKDDDDK	3225.7	1613.3	1075.9	807.2	645.9	1075.8
ZMCEHRHPFTQYWLRCGSGSDYKDDDDK	3330.6	1665.8	1110.9	833.4	666.9	1110.8
ZMCVYEYVFDRLTVTCGSGSDYKDDDDK	3165.4	1583.2	1055.8	792.1	633.9	1055.8
ZMCYYEVLWYQQQRHCGSGSDYKDDDDK	3373.6	1687.3	1125.2	844.1	675.5	1125.1
ZMCRDFDIIWYKLQFCGSGSDYKDDDDK	3304.6	1652.8	1102.2	826.9	661.7	1102.1
ZMCYTRHPFQKRWHYCGSGSEEGSGSDYKDDDDK	3926.1	1963.6	1309.4	982.3	786.0	982.2
ZMCKFDLDLLFYKIWCGSGSDYKDDDDK	3261.7	1631.3	1087.9	816.2	653.1	1087.8
ZMCYEVLWYQYWRKVCGSGSDYKDDDDK	3393.7	1697.4	1131.9	849.2	679.5	1131.8
ZMCYTKAYWHEPWRPCGSGSDYKDDDDK	3294.5	1647.8	1098.8	824.4	659.7	1098.8
ZMCEVIWYKIQYRLCGSGSDYKDDDDK	3171.5	1586.3	1057.8	793.6	635.1	1057.7
ZMCDLFYYLFIKRRKCGSGSEEGSGSDYKDDDDK	3869.2	1935.1	1290.4	968.1	774.6	968.0
ZMCEHRHPYEAFWHECGSGSDYKDDDDK	3298.5	1649.7	1100.2	825.4	660.5	1100.0
ZMCHFKRDYYWYLWQCGSGSDYKDDDDK	3465.7	1733.4	1155.9	867.2	693.9	1155.9
ZMCVHRHRFEPEWHVCGSGSDYKDDDDK	3289.5	1645.3	1097.2	823.1	658.7	1097.1
ZMCWWIQHPLFWSKICGSGSDYKDDDDK	3301.7	1651.3	1101.2	826.2	661.1	1101.1
ZMCYTRKPFEFFWRLCGSGSDYKDDDDK	3350.7	1675.9	1117.6	838.4	670.9	1117.4
ZMCWEEFTKFGWWTICGSGSDYKDDDDK	3290.5	1645.8	1097.5	823.4	658.9	1097.5
ZMCAYRHIYEPYWHSCGSGSDYKDDDDK	3282.5	1641.7	1094.8	821.4	657.3	1094.7
ZMCYTKATVFSQWVLCGSGSDYKDDDDK	3103.4	1552.2	1035.1	776.6	621.5	1035.1
ZMCHLWYYLVHHRRDCGSGSDYKDDDDK	3355.6	1678.3	1119.2	839.7	671.9	1119.1
ZMCKYSVYWYKWQRKCGSGSDYKDDDDK	3395.7	1698.4	1132.6	849.7	679.9	1132.5

ZMCEHKHPFERVWRSCGSGSDYKDDDDK	3268.5	1634.8	1090.2	817.9	654.5	1090.0
ZMCKYHRDLLYYFLHCGSGSDYKDDDDK	3328.7	1664.8	1110.2	832.9	666.5	1110.1
ZMCEYRHKFEERWHVCGSGSDYKDDDDK	3376.6	1688.8	1126.2	844.9	676.1	1126.1
ZMCYTRRFKWQDWHACGSGSDYKDDDDK	3354.6	1677.8	1118.9	839.4	671.7	1118.8
ZMCYTRHKFDHRWRACGSGSEEGSGSDYKDDDDK	3880.1	1940.5	1294.0	970.8	776.8	970.8
ZMCEFEIRWYQYQRKCGSGSDYKDDDDK	3406.7	1703.8	1136.2	852.4	682.1	1136.2
ZMCYTKYFYFEPWRICGSGSDYKDDDDK	3373.7	1687.3	1125.2	844.2	675.5	1125.2
ZMCYTRDRYELHWRLCGSGSDYKDDDDK	3368.6	1684.8	1123.5	842.9	674.5	1123.5
ZMCTTRKNKWEEWRPCGSGSDYKDDDDK	3291.5	1646.3	1097.8	823.6	659.1	1097.8
ZMCWEEIIWYKLQHKCGSGSDYKDDDDK	3333.7	1667.3	1111.9	834.2	667.5	1111.7
ZMCYTKSFYWPIWRSCGSGSDYKDDDDK	3294.6	1647.8	1098.9	824.4	659.7	1098.8
ZMCYTRHKFDQYWQPCGSGSDYKDDDDK	3329.6	1665.3	1110.5	833.1	666.7	1110.5
ZMCEYTYLFGYHYIKCGSGSDYKDDDDK	3257.5	1629.3	1086.5	815.1	652.3	1086.4
ZMCYTRLNKHAKWQKCGSGSDYKDDDDK	3233.5	1617.3	1078.5	809.1	647.5	1078.4
ZMCNKHLYYYLLFKQCGSGSDYKDDDDK	3290.6	1645.8	1097.5	823.4	658.9	1097.5
ZMCFNVHWYRWIRRRCGSGSDYKDDDDK	3449.8	1725.4	1150.6	863.2	690.8	1150.5
ZMCAYEYVFDRLTVTCGSGSDYKDDDDK	3137.4	1569.2	1046.5	785.1	628.3	1046.3
ZMCFRHKRSDFWLQFCGSGSDYKDDDDK	3327.7	1664.3	1109.9	832.7	666.3	1109.6
ZMCFKQHLFYYLLHCGSGSDYKDDDDK	3169.5	1585.3	1057.2	793.1	634.7	1057.1
ZMCRHLAYWKLWHRKCGSGSDYKDDDDK	3354.7	1677.9	1118.9	839.4	671.7	1118.9
ZMCVIVIYKILRSLCGSGSDYKDDDDK	2977.4	1489.2	993.1	745.1	596.3	373.2
ZMCLHIKANYGFVRVCGSGSDYKDDDDK	3077.4	1539.2	1026.5	770.1	616.3	1026.4
ZMCHWDIYWYRWVRRCGSGSDYKDDDDK	3496.8	1748.9	1166.3	875.0	700.2	1166.1
ZMCVHKKWIRYWQIFCGSGSEEGSGSDYKDDDDK	3911.3	1956.1	1304.4	978.6	783.1	978.8
ZMCEHRHPYSNFWIPCGSGSDYKDDDDK	3243.5	1622.2	1081.8	811.6	649.5	1081.9
ZMCITRHRFEQFWHLCGSGSDYKDDDDK	3330.6	1665.8	1110.9	833.4	666.9	1110.9
ZMCHVILWRSWRYCGSGSDYKDDDDK	3076.4	1538.7	1026.1	769.8	616.1	1026.0
ZMCIFDIHMPFIPCGSGSDYKDDDDK	2890.3	1445.6	964.1	723.3	578.9	964.1
