# Supplementary material for: Identification and engineering of potent cyclic peptides with selective or promiscuous binding through biochemical profiling and bioinformatic data analysis
Source: RSC Chem Biol. 2023 Nov 14;5(1):12–8. doi: 10.1039/d3cb00168g (PMC10763615; doi:10.1039/d3cb00168g)
Supplement: CB-005-D3CB00168G-s004 [file CB-005-D3CB00168G-s004.pdf]

Electronic Supplementary Material (ESI) for RSC Chemical Biology.
This journal is © The Royal Society of Chemistry 2023

sequence	kd M (FGF-R1C)	kd M(FGF-R3C)	kd M (FGF-R4)	determination method (e.g. single	peptide type (selection or	selected against	Library	comment
ZMCFRWRLFHHLTPYCGSGSDYKDDDDK	1.42E-07	>1.00E-05	1.46E-07	Crude	Selection	FGFR-1C	NNK	
ZMCIRWPARQSLFWLCGSGSDYKDDDDK	3.10E-08	4.63E-07	8.80E-08	Crude	Selection	FGFR-1C	NNK	
ZMCSNMWKLANRWWACGSGSDYKDDDDK	3.91E-07	>1.00E-05	>1.00E-05	Crude	Selection	FGFR-1C	NNK	
ZMCSTRFVNLFWLCGSGSDYKDDDDK	>1.00E-05	>1.00E-05	>1.00E-05	Crude	Selection	FGFR-1C	NNK	
ZMCVPCFSHTFLCCGSGSDYKDDDDK	5.90E-08	5.40E-08	>1.00E-05	Crude	Selection	FGFR-1C	NNK	
ZMCWYCFRKSFWCCGSGSDYKDDDDK	>1.00E-05	1.25E-06	>1.00E-05	Crude	Selection	FGFR-1C	NNK	
ZMCYRFPSSHLFWACGSGSDYKDDDDK	>1.00E-05	>1.00E-05	>1.00E-05	Crude	Selection	FGFR-1C	NNK	
ZMCVPCFSHTFLCCGSGSDYKDDDDK	>1.00E-05	1.31E-07	>1.00E-05	**Purified**	Selection	FGFR-1c	NNK	, 1st disulfide isomer
ZMCVPCFSHTFLCCGSGSDYKDDDDK	5.64E-08	4.60E-08	>1.00E-05	**Purified**	Selection	FGFR-1c	NNK	, 2nd disulfide isomer
MCCFKFRDLFWVCGSGSDYKDDDDK	7.16E-08	4.27E-05	>1.00E-05	Crude	Selection	FGF-R1C	NNK	
MCCTRFVNLFWLCGSGSDYKDDDDK	1.43E-07	>1.00E-05	>1.00E-05	Crude	Selection	FGF-R1C	NNK	
MCCVRYLARFWLCGSGSDYKDDDDK	>1.00E-05	2.72E-07	1.08E-07	Crude	Selection	FGF-R1C	NNK	
MCCYNLFSLFWACGSGSDYKDDDDK	>1.00E-05	>1.00E-05	>1.00E-05	Crude	Selection	FGF-R1C	NNK	
MCCYNLFSLFWCCGSGSDYKDDDDK	>1.00E-05	>1.00E-05	>1.00E-05	Crude	Selection	FGF-R1C	NNK	
MCCYRCWSLFWCCGSGSDYKDDDDK	>1.00E-05	>1.00E-05	>1.00E-05	Crude	Selection	FGF-R1C	NNK	
MCFRCFLASFWCCGSGSDYKDDDDK	>1.00E-05	>1.00E-05	>1.00E-05	Crude	Selection	FGF-R1C	NNK	
MCFRWRLFHHLTPFCGSGSDYKDDDDK	4.99E-08	>1.00E-05	7.90E-08	Crude	Selection	FGF-R1C	NNK	
MCFRWRLFHHLTPYCGSGSDYKDDDDK	1.28E-07	5.80E-08	>1.00E-05	Crude	Selection	FGF-R1C	NNK	
MCFRWRLFHHLTPYCGSGSDYKDDDDK	1.33E-07	2.90E-08	>1.00E-05	Crude	Selection	FGF-R1C	NNK	
MCFRWRLFHHLTPYCGSGSDYKDDDDK	1.36E-07	>1.00E-05	4.00E-07	Crude	Selection	FGF-R1C	NNK	
MCFVCFRHSFLCCGSGSDYKDDDDK	>1.00E-05	>1.00E-05	9.50E-08	Crude	Selection	FGF-R1C	NNK	
MCFVCFRHSFLCCGSGSDYKDDDDK	>1.00E-05	>1.00E-05	>1.00E-05	Crude	Selection	FGF-R1C	NNK	
MCFVCFRHSFLCCGSGSDYKDDDDK	>1.00E-05	>1.00E-05	>1.00E-05	Crude	Selection	FGF-R1C	NNK	
MCFVCFRHSFWCCGSGSDYKDDDDK	>1.00E-05	>1.00E-05	4.40E-08	Crude	Selection	FGF-R1C	NNK	
MCFVYPRRQPLYWICGSGSDYKDDDDK	1.35E-07	>1.00E-05	1.27E-07	Crude	Selection	FGF-R1C	NNK	
MCFVYPRRQPLYWICGSGSDYKDDDDK	1.47E-07	4.40E-08	>1.00E-05	Crude	Selection	FGF-R1C	NNK	
MCHFRPNKFTAWLPCGSGSDYKDDDDK	>1.00E-05	3.87E-07	>1.00E-05	Crude	Selection	FGF-R1C	NNK	
MCHPTRLHLWLCCNCGSGSDYKDDDDK	>1.00E-05	>1.00E-05	>1.00E-05	Crude	Selection	FGF-R1C	NNK	
MCIFELNLPFIPCGSGSDYKDDDDK	>1.00E-05	>1.00E-05	>1.00E-05	Crude	Selection	FGF-R1C	NNK	
MCIGKWYTYQYWTRCGSGSDYKDDDDK	4.88E-07	>1.00E-05	>1.00E-05	Crude	Selection	FGF-R1C	NNK	
MCIGKWYTYQYWTRCGSGSDYKDDDDK	5.42E-06	>1.00E-05	>1.00E-05	Crude	Selection	FGF-R1C	NNK	
MCILHGLIYWAPKQCGSGSDYKDDDDK	>1.00E-05	>1.00E-05	>1.00E-05	Crude	Selection	FGF-R1C	NNK	
MCIRWPARQPLFWLCGSGSDYKDDDDK	1.08E-08	7.90E-08	9.48E-07	Crude	Selection	FGF-R1C	NNK	
MCIRWPARQPLFWLCGSGSDYKDDDDK	1.94E-08	1.12E-07	8.20E-08	Crude	Selection	FGF-R1C	NNK	
MCIRWPARQSLFWICGSGSDYKDDDDK	4.90E-08	6.50E-08	>1.00E-05	Crude	Selection	FGF-R1C	NNK	
MCIRWPARQSLFWLCGSGSDYKDDDDK	8.49E-08	1.57E-07	>1.00E-05	Crude	Selection	FGF-R1C	NNK	
MCIYRLSDFWVVCGSGSDYKDDDDK	6.80E-08	6.30E-07	5.31E-07	Crude	Selection	FGF-R1C	NNK	
MCIYRLSDFWVVCGSGSDYKDDDDK	>1.00E-05	9.90E-08	2.29E-07	Crude	Selection	FGF-R1C	NNK	
MCLLWTRHFDRYWVCGSGSDYKDDDDK	9.49E-08	>1.00E-05	>1.00E-05	Crude	Selection	FGF-R1C	NNK	
MCLLWTRHFDRYWVCGSGSDYKDDDDK	2.70E-07	>1.00E-05	>1.00E-05	Crude	Selection	FGF-R1C	NNK	
MCRFYELFWSFPCGSGSDYKDDDDK	>1.00E-05	>1.00E-05	>1.00E-05	Crude	Selection	FGF-R1C	NNK	
MCRLCFSKLFWCCGSGSDYKDDDDK	5.00E-08	>1.00E-05	>1.00E-05	Crude	Selection	FGF-R1C	NNK	
MCRLCFSKLFWCCGSGSDYKDDDDK	>1.00E-05	1.45E-05	>1.00E-05	Crude	Selection	FGF-R1C	NNK	
MCRLCFSKLFWYCGSGSDYKDDDDK	>1.00E-05	>1.00E-05	>1.00E-05	Crude	Selection	FGF-R1C	NNK	
MCSNMWKLANRWWACGSGSDYKDDDDK	5.33E-08	>1.00E-05	1.05E-06	Crude	Selection	FGF-R1C	NNK	
MCSNMWKLANRWWACGSGSDYKDDDDK	1.12E-07	3.00E-08	>1.00E-05	Crude	Selection	FGF-R1C	NNK	
MCSQSWVLYELWRVCGSGSDYKDDDDK	>1.00E-05	>1.00E-05	>1.00E-05	Crude	Selection	FGF-R1C	NNK	
MCSYGSWYGYSYLACGSGSDYKDDDDK	>1.00E-05	>1.00E-05	>1.00E-05	Crude	Selection	FGF-R1C	NNK	
MCSYGSWYGYSYLACGSGSDYKDDDDK	>1.00E-05	>1.00E-05	>1.00E-05	Crude	Selection	FGF-R1C	NNK	
MCTKFPWPFPIFWACGSGSDYKDDDDK	1.55E-07	4.70E-08	7.00E-08	Crude	Selection	FGF-R1C	NNK	
MCTKFPWPFPIFWACGSGSDYKDDDDK	3.80E-07	>1.00E-05	2.41E-07	Crude	Selection	FGF-R1C	NNK	
MCTKFPWPLPIFWACGSGSDYKDDDDK	5.45E-07	>1.00E-05	3.46E-07	Crude	Selection	FGF-R1C	NNK	
MCTRTSFYGFWYYACGSGSDYKDDDDK	8.36E-08	2.13E-07	1.72E-07	Crude	Selection	FGF-R1C	NNK	
MCTRTSFYGFWYYACGSGSDYKDDDDK	1.67E-07	3.35E-07	>1.00E-05	Crude	Selection	FGF-R1C	NNK	
MCVPCFSHTFLCCGSGSDYKDDDDK	>1.00E-05	4.75E-07	>1.00E-05	Crude	Selection	FGF-R1C	NNK	
MCVPCFSHTFLCCGSGSDYKDDDDK	>1.00E-05	5.46E-06	>1.00E-05	Crude	Selection	FGF-R1C	NNK	
MCVRWPNLNALFWLCGSGSDYKDDDDK	3.84E-07	1.52E-06	>1.00E-05	Crude	Selection	FGF-R1C	NNK	
MCWSLYRLFGSGSDYKDDDDK	>1.00E-05	>1.00E-05	>1.00E-05	Crude	Selection	FGF-R1C	NNK	
MCWSLYRLFWACGSGSDYKDDDDK	1.77E-07	6.03E-06	>1.00E-05	Crude	Selection	FGF-R1C	NNK	
MCWYCFRKSFWCCGSGSDYKDDDDK	>1.00E-05	>1.00E-05	>1.00E-05	Crude	Selection	FGF-R1C	NNK	
MCYKCFTGLFLCCGSGSDYKDDDDK	>1.00E-05	3.89E-05	>1.00E-05	Crude	Selection	FGF-R1C	NNK	
MCYKWPIRSHLYWLCGSGSDYKDDDDK	1.44E-07	1.46E-07	>1.00E-05	Crude	Selection	FGF-R1C	NNK	
MCYKWPIRSHLYWLCGSGSDYKDDDDK	6.34E-07	9.96E-06	>1.00E-05	Crude	Selection	FGF-R1C	NNK	
MCYRHPHSNPLFWACGSGSDYKDDDDK	8.50E-08	>1.00E-05	>1.00E-05	Crude	Selection	FGF-R1C	NNK	
MCYRHPHSNPLFWACGSGSDYKDDDDK	2.37E-07	>1.00E-05	>1.00E-05	Crude	Selection	FGF-R1C	NNK	
MCYVCFSHSFICCGSGSDYKDDDDK	>1.00E-05	>1.00E-05	>1.00E-05	Crude	Selection	FGF-R1C	NNK	
MCYVCFSHSFICCGSGSDYKDDDDK	>1.00E-05	>1.00E-05	>1.00E-05	Crude	Selection	FGF-R1C	NNK	
MCIGKWYTYQYWTRCGSGSDYKDDDDK	1.11E-07	>1.00E-05	>1.00E-05	**Purified**	Selection	FGF-R1c	NNK	
MCLLWTRHFDRYWVCGSGSDYKDDDDK	6.37E-08	>1.00E-05	>1.00E-05	**Purified**	Selection	FGF-R1c	NNK	
MCSNMWKLANRWWACGSGSDYKDDDDK	5.79E-08	5.46E-06	>1.00E-05	**Purified**	Selection	FGF-R1c	NNK	
MCTKFPWPFPIFWACGSGSDYKDDDDK	3.23E-07	>1.00E-05	4.64E-07	**Purified**	Selection	FGF-R1c	NNK	
MCIRWPARQPLFWLCGSGSDYKDDDDK	1.15E-09	4.67E-08	8.20E-08	**Purified**	Selection	FGF-R1c	NNK	
MCFRWRLFHHLTPFCGSGSDYKDDDDK	1.01E-07	>1.00E-05	7.29E-08	**Purified**	Selection	FGF-R1c	NNK	
Z(Nle)CFQYFHILWPFCGSGSDYKDDDDK	>1.00E-05	>1.00E-05	>1.00E-05	Crude	Mutagenesis scan	FGF-R1c	NNK	
Z(Nle)CFTHFHMLWPFCGSGSDYKDDDDK	>1.00E-05	>1.00E-05	>1.00E-05	Crude	Mutagenesis scan	FGF-R1c	NNK	
Z(Nle)CIRWPARQPLFWLCGSGSDYKDDDDK	2.00E-09	2.86E-08	8.25E-08	Crude	Mutagenesis scan	FGF-R1c	NNK	
ZACIRWPARQPLFWLCGSGSDYKDDDDK	9.85E-09	4.45E-08	1.19E-07	Crude	Mutagenesis scan	FGF-R1c	NNK	
ZMCARWPARQPLFWLCGSGSDYKDDDDK	1.09E-08	2.14E-07	1.83E-08	Crude	Mutagenesis scan	FGF-R1c	NNK	
ZMCDRWPARQPLFWLCGSGSDYKDDDDK	1.59E-08	8.96E-07	1.19E-08	Crude	Mutagenesis scan	FGF-R1c	NNK	
ZMCERWPARQPLFWLCGSGSDYKDDDDK	4.60E-09	2.49E-07	3.88E-08	Crude	Mutagenesis scan	FGF-R1c	NNK	
ZMCFRWPARQPLFWLCGSGSDYKDDDDK	4.02E-09	6.53E-08	2.95E-08	Crude	Mutagenesis scan	FGF-R1c	NNK	
ZMCGRWPARQPLFWLCGSGSDYKDDDDK	8.75E-09	1.07E-07	6.43E-08	Crude	Mutagenesis scan	FGF-R1c	NNK	
ZMCHRWPARQPLFWLCGSGSDYKDDDDK	2.17E-09	1.54E-08	4.83E-08	Crude	Mutagenesis scan	FGF-R1c	NNK	
ZMCIAWPARQPLFWLCGSGSDYKDDDDK	2.23E-08	2.63E-07	2.57E-09	Crude	Mutagenesis scan	FGF-R1c	NNK	
ZMCIDWPARQPLFWLCGSGSDYKDDDDK	>1.00E-05	>1.00E-05	1.61E-07	Crude	Mutagenesis scan	FGF-R1c	NNK	
ZMCIEWPARQPLFWLCGSGSDYKDDDDK	>1.00E-05	7.79E-08	>1.00E-05	Crude	Mutagenesis scan	FGF-R1c	NNK	
ZMCIFWPARQPLFWLCGSGSDYKDDDDK	1.37E-07	>1.00E-05	3.36E-09	Crude	Mutagenesis scan	FGF-R1c	NNK	
ZMCIGWPARQPLFWLCGSGSDYKDDDDK	4.23E-08	4.61E-07	1.59E-08	Crude	Mutagenesis scan	FGF-R1c	NNK	
ZMCIHWPARQPLFWLCGSGSDYKDDDDK	>1.00E-05	3.44E-07	1.34E-09	Crude	Mutagenesis scan	FGF-R1c	NNK	
ZMCIIWPARQPLFWLCGSGSDYKDDDDK	1.60E-08	1.66E-07	3.95E-08	Crude	Mutagenesis scan	FGF-R1c	NNK	
ZMCIKWPARQPLFWLCGSGSDYKDDDDK	2.51E-08	1.31E-08	3.37E-09	Crude	Mutagenesis scan	FGF-R1c	NNK	
ZMCILWPARQPLFWLCGSGSDYKDDDDK	1.53E-08	3.97E-08	7.99E-09	Crude	Mutagenesis scan	FGF-R1c	NNK	
ZMCIPWPARQPLFWLCGSGSDYKDDDDK	>1.00E-05	2.42E-08	>1.00E-05	Crude	Mutagenesis scan	FGF-R1c	NNK	
ZMCIQWPARQPLFWLCGSGSDYKDDDDK	>1.00E-05	2.73E-08	2.16E-09	Crude	Mutagenesis scan	FGF-R1c	NNK	
ZMCIRAPARQPLFWLCGSGSDYKDDDDK	1.74E-07	3.19E-07	>1.00E-05	Crude	Mutagenesis scan	FGF-R1c	NNK	
ZMCIRDPARQPLFWLCGSGSDYKDDDDK	7.09E-07	4.94E-07	2.77E-08	Crude	Mutagenesis scan	FGF-R1c	NNK	
ZMCIREPARQPLFWLCGSGSDYKDDDDK	2.18E-07	>1.00E-05	2.96E-08	Crude	Mutagenesis scan	FGF-R1c	NNK	
ZMCIRFPARQPLFWLCGSGSDYKDDDDK	2.53E-08	1.58E-08	1.69E-09	Crude	Mutagenesis scan	FGF-R1c	NNK	
ZMCIRGPARQPLFWLCGSGSDYKDDDDK	>1.00E-05	2.43E-08	>1.00E-05	Crude	Mutagenesis scan	FGF-R1c	NNK	

ZMCIRHPARQPLFWLCGSGSDYKDDDDK	5.64E-08	1.05E-07	9.09E-09	Crude		Mutagenesis scan	FGF-R1c	NNK
ZMCIRIPARQPLFWLCGSGSDYKDDDDK	6.48E-08	4.07E-08	6.01E-09	Crude		Mutagenesis scan	FGF-R1c	NNK
ZMCIRKPARQPLFWLCGSGSDYKDDDDK	>1.00E-05	>1.00E-05	>1.00E-05	Crude		Mutagenesis scan	FGF-R1c	NNK
ZMCIRLPARQPLFWLCGSGSDYKDDDDK	5.42E-07	2.50E-07	3.13E-09	Crude		Mutagenesis scan	FGF-R1c	NNK
ZMCIRPPARQPLFWLCGSGSDYKDDDDK	>1.00E-05	>1.00E-05	>1.00E-05	Crude		Mutagenesis scan	FGF-R1c	NNK
ZMCIRQPARQPLFWLCGSGSDYKDDDDK	>1.00E-05	>1.00E-05	1.27E-08	Crude		Mutagenesis scan	FGF-R1c	NNK
ZMCIRRPARQPLFWLCGSGSDYKDDDDK	>1.00E-05	>1.00E-05	>1.00E-05	Crude		Mutagenesis scan	FGF-R1c	NNK
ZMCIRSPARQPLFWLCGSGSDYKDDDDK	2.83E-08	2.44E-07	4.04E-09	Crude		Mutagenesis scan	FGF-R1c	NNK
ZMCIRTPARQPLFWLCGSGSDYKDDDDK	4.07E-07	>1.00E-05	2.84E-08	Crude		Mutagenesis scan	FGF-R1c	NNK
ZMCIRVPARQPLFWLCGSGSDYKDDDDK	>1.00E-05	4.16E-07	>1.00E-05	Crude		Mutagenesis scan	FGF-R1c	NNK
ZMCIRWPADQPLFWLCGSGSDYKDDDDK	>1.00E-05	1.82E-07	>1.00E-05	Crude		Mutagenesis scan	FGF-R1c	NNK
ZMCIRWPAEQPLFWLCGSGSDYKDDDDK	>1.00E-05	6.86E-08	>1.00E-05	Crude		Mutagenesis scan	FGF-R1c	NNK
ZMCIRWPAFQPLFWLCGSGSDYKDDDDK	>1.00E-05	1.10E-07	>1.00E-05	Crude		Mutagenesis scan	FGF-R1c	NNK
ZMCIRWPAGQPLFWLCGSGSDYKDDDDK	3.65E-08	1.24E-07	>1.00E-05	Crude		Mutagenesis scan	FGF-R1c	NNK
ZMCIRWPAHQPLFWLCGSGSDYKDDDDK	2.85E-08	1.89E-07	>1.00E-05	Crude		Mutagenesis scan	FGF-R1c	NNK
ZMCIRWPAIQPLFWLCGSGSDYKDDDDK	1.03E-08	6.99E-08	>1.00E-05	Crude		Mutagenesis scan	FGF-R1c	NNK
ZMCIRWPAKQPLFWLCGSGSDYKDDDDK	2.29E-08	4.19E-08	>1.00E-05	Crude		Mutagenesis scan	FGF-R1c	NNK
ZMCIRWPALQPLFWLCGSGSDYKDDDDK	5.82E-08	7.77E-08	>1.00E-05	Crude		Mutagenesis scan	FGF-R1c	NNK
ZMCIRWPAPQPLFWLCGSGSDYKDDDDK	3.31E-08	5.58E-08	>1.00E-05	Crude		Mutagenesis scan	FGF-R1c	NNK
ZMCIRWPAQQPLFWLCGSGSDYKDDDDK	>1.00E-05	4.31E-08	>1.00E-05	Crude		Mutagenesis scan	FGF-R1c	NNK
ZMCIRWPARAPLFWLCGSGSDYKDDDDK	2.06E-08	3.26E-08	8.82E-10	Crude		Mutagenesis scan	FGF-R1c	NNK
ZMCIRWPARDPLFWLCGSGSDYKDDDDK	2.76E-08	3.21E-08	4.37E-10	Crude		Mutagenesis scan	FGF-R1c	NNK
ZMCIRWPAREPLFWLCGSGSDYKDDDDK	2.38E-08	1.46E-08	3.23E-09	Crude		Mutagenesis scan	FGF-R1c	NNK
ZMCIRWPARFPLFWLCGSGSDYKDDDDK	1.38E-08	2.37E-08	1.29E-09	Crude		Mutagenesis scan	FGF-R1c	NNK
ZMCIRWPARGPLFWLCGSGSDYKDDDDK	3.13E-08	5.04E-08	2.46E-08	Crude		Mutagenesis scan	FGF-R1c	NNK
ZMCIRWPARHPLFWLCGSGSDYKDDDDK	2.08E-08	4.60E-08	1.35E-07	Crude		Mutagenesis scan	FGF-R1c	NNK
ZMCIRWPARIPLFWLCGSGSDYKDDDDK	1.79E-08	3.52E-08	1.44E-09	Crude		Mutagenesis scan	FGF-R1c	NNK
ZMCIRWPARKPLFWLCGSGSDYKDDDDK	2.07E-08	8.12E-08	1.17E-08	Crude		Mutagenesis scan	FGF-R1c	NNK
ZMCIRWPARLPLFWLCGSGSDYKDDDDK	8.80E-09	3.81E-08	1.30E-09	Crude		Mutagenesis scan	FGF-R1c	NNK
ZMCIRWPARNPLFWLCGSGSDYKDDDDK	1.42E-08	8.92E-08	9.47E-08	Crude		Mutagenesis scan	FGF-R1c	NNK
ZMCIRWPARPPLFWLCGSGSDYKDDDDK	2.10E-08	1.20E-08	4.40E-09	Crude		Mutagenesis scan	FGF-R1c	NNK
ZMCIRWPARQALFWLCGSGSDYKDDDDK	1.95E-08	8.30E-09	2.26E-07	Crude		Mutagenesis scan	FGF-R1c	NNK
ZMCIRWPARQDLFWLCGSGSDYKDDDDK	4.26E-07	8.27E-09	1.95E-08	Crude		Mutagenesis scan	FGF-R1c	NNK
ZMCIRWPARQELFWLCGSGSDYKDDDDK	7.40E-08	1.69E-08	>1.00E-05	Crude		Mutagenesis scan	FGF-R1c	NNK
ZMCIRWPARQGLFWLCGSGSDYKDDDDK	2.79E-08	2.76E-08	2.81E-08	Crude		Mutagenesis scan	FGF-R1c	NNK
ZMCIRWPARQHLFWLCGSGSDYKDDDDK	6.36E-09	3.51E-09	7.93E-09	Crude		Mutagenesis scan	FGF-R1c	NNK
ZMCIRWPARQILFWLCGSGSDYKDDDDK	>1.00E-05	>1.00E-05	>1.00E-05	Crude		Mutagenesis scan	FGF-R1c	NNK
ZMCIRWPARQKLFWLCGSGSDYKDDDDK	8.52E-08	9.91E-08	>1.00E-05	Crude		Mutagenesis scan	FGF-R1c	NNK
ZMCIRWPARQLLFWLCGSGSDYKDDDDK	>1.00E-05	>1.00E-05	>1.00E-05	Crude		Mutagenesis scan	FGF-R1c	NNK
ZMCIRWPARQPAFWLCGSGSDYKDDDDK	1.23E-08	7.16E-08	1.32E-08	Crude		Mutagenesis scan	FGF-R1c	NNK
ZMCIRWPARQPDFWLCGSGSDYKDDDDK	1.10E-07	1.90E-07	>1.00E-05	Crude		Mutagenesis scan	FGF-R1c	NNK
ZMCIRWPARQPEFWLCGSGSDYKDDDDK	2.58E-08	1.70E-07	>1.00E-05	Crude		Mutagenesis scan	FGF-R1c	NNK
ZMCIRWPARQPFFWLCGSGSDYKDDDDK	1.19E-07	4.02E-08	5.73E-08	Crude		Mutagenesis scan	FGF-R1c	NNK
ZMCIRWPARQPGFWLCGSGSDYKDDDDK	3.47E-08	5.73E-08	1.76E-08	Crude		Mutagenesis scan	FGF-R1c	NNK
ZMCIRWPARQPHFWLCGSGSDYKDDDDK	1.84E-08	8.86E-08	2.34E-08	Crude		Mutagenesis scan	FGF-R1c	NNK
ZMCIRWPARQPIFWLCGSGSDYKDDDDK	3.51E-09	4.15E-08	1.59E-08	Crude		Mutagenesis scan	FGF-R1c	NNK
ZMCIRWPARQPLAWLCGSGSDYKDDDDK	9.60E-09	2.61E-08	>1.00E-05	Crude		Mutagenesis scan	FGF-R1c	NNK
ZMCIRWPARQPLDWLCGSGSDYKDDDDK	>1.00E-05	>1.00E-05	>1.00E-05	Crude		Mutagenesis scan	FGF-R1c	NNK
ZMCIRWPARQPLEWLCGSGSDYKDDDDK	>1.00E-05	>1.00E-05	>1.00E-05	Crude		Mutagenesis scan	FGF-R1c	NNK
ZMCIRWPARQPLFALCGSGSDYKDDDDK	>1.00E-05	>1.00E-05	>1.00E-05	Crude		Mutagenesis scan	FGF-R1c	NNK
ZMCIRWPARQPLFDLCGSGSDYKDDDDK	>1.00E-05	>1.00E-05	>1.00E-05	Crude		Mutagenesis scan	FGF-R1c	NNK
ZMCIRWPARQPLFELCGSGSDYKDDDDK	>1.00E-05	>1.00E-05	>1.00E-05	Crude		Mutagenesis scan	FGF-R1c	NNK
ZMCIRWPARQPLFFLCGSGSDYKDDDDK	2.89E-08	5.23E-08	>1.00E-05	Crude		Mutagenesis scan	FGF-R1c	NNK
ZMCIRWPARQPLFGLCGSGSDYKDDDDK	>1.00E-05	>1.00E-05	>1.00E-05	Crude		Mutagenesis scan	FGF-R1c	NNK
ZMCIRWPARQPLFHLCGSGSDYKDDDDK	1.56E-08	3.34E-08	7.68E-07	Crude		Mutagenesis scan	FGF-R1c	NNK
ZMCIRWPARQPLFILCGSGSDYKDDDDK	>1.00E-05	>1.00E-05	>1.00E-05	Crude		Mutagenesis scan	FGF-R1c	NNK
ZMCIRWPARQPLFKLCGSGSDYKDDDDK	>1.00E-05	>1.00E-05	>1.00E-05	Crude		Mutagenesis scan	FGF-R1c	NNK
ZMCIRWPARQPLFLLCGSGSDYKDDDDK	3.87E-09	6.76E-08	>1.00E-05	Crude		Mutagenesis scan	FGF-R1c	NNK
ZMCIRWPARQPLFPLCGSGSDYKDDDDK	>1.00E-05	>1.00E-05	>1.00E-05	Crude		Mutagenesis scan	FGF-R1c	NNK
ZMCIRWPARQPLFQLCGSGSDYKDDDDK	2.10E-07	9.44E-08	>1.00E-05	Crude		Mutagenesis scan	FGF-R1c	NNK
ZMCIRWPARQPLFRLCGSGSDYKDDDDK	6.20E-08	>1.00E-05	>1.00E-05	Crude		Mutagenesis scan	FGF-R1c	NNK
ZMCIRWPARQPLFSLCGSGSDYKDDDDK	3.77E-07	>1.00E-05	>1.00E-05	Crude		Mutagenesis scan	FGF-R1c	NNK
ZMCIRWPARQPLFTLCGSGSDYKDDDDK	2.69E-07	1.07E-07	1.04E-07	Crude		Mutagenesis scan	FGF-R1c	NNK
ZMCIRWPARQPLFVLCGSGSDYKDDDDK	7.10E-07	>1.00E-05	>1.00E-05	Crude		Mutagenesis scan	FGF-R1c	NNK
ZMCIRWPARQPLFWACGSGSDYKDDDDK	9.31E-09	3.07E-08	4.01E-08	Crude		Mutagenesis scan	FGF-R1c	NNK
ZMCIRWPARQPLFWDCGSGSDYKDDDDK	>1.00E-05	2.96E-07	>1.00E-05	Crude		Mutagenesis scan	FGF-R1c	NNK
ZMCIRWPARQPLFWECGSGSDYKDDDDK	1.10E-07	>1.00E-05	>1.00E-05	Crude		Mutagenesis scan	FGF-R1c	NNK
ZMCIRWPARQPLFWFCGSGSDYKDDDDK	3.29E-08	2.57E-08	2.77E-08	Crude		Mutagenesis scan	FGF-R1c	NNK
ZMCIRWPARQPLFWHCGSGSDYKDDDDK	1.32E-08	3.07E-08	2.35E-08	Crude		Mutagenesis scan	FGF-R1c	NNK
ZMCIRWPARQPLFWICGSGSDYKDDDDK	4.87E-09	2.85E-08	1.06E-08	Crude		Mutagenesis scan	FGF-R1c	NNK
ZMCIRWPARQPLFWKCGSGSDYKDDDDK	1.22E-07	4.80E-08	1.05E-07	Crude		Mutagenesis scan	FGF-R1c	NNK
ZMCIRWPARQPLFWLCGSGSDYKDDDDK	1.33E-08	3.26E-08	2.58E-08	**Crude**		Mutagenesis scan	FGF-R1c	NNK
ZMCIRWPARQPLFWPCGSGSDYKDDDDK	8.26E-08	1.10E-07	3.52E-07	Crude		Mutagenesis scan	FGF-R1c	NNK
ZMCIRWPARQPLFWQCGSGSDYKDDDDK	2.60E-08	1.32E-07	5.61E-08	Crude		Mutagenesis scan	FGF-R1c	NNK
ZMCIRWPARQPLFWRCGSGSDYKDDDDK	2.15E-08	2.80E-08	5.29E-08	Crude		Mutagenesis scan	FGF-R1c	NNK
ZMCIRWPARQPLFWSCGSGSDYKDDDDK	2.56E-08	3.45E-08	6.25E-08	Crude		Mutagenesis scan	FGF-R1c	NNK
ZMCIRWPARQPLFWTCGSGSDYKDDDDK	4.29E-08	4.16E-08	1.19E-07	Crude		Mutagenesis scan	FGF-R1c	NNK
ZMCIRWPARQPLFWVCGSGSDYKDDDDK	6.85E-09	3.42E-08	2.17E-08	Crude		Mutagenesis scan	FGF-R1c	NNK
ZMCIRWPARQPLFWWCGSGSDYKDDDDK	3.51E-08	2.49E-08	2.35E-08	Crude		Mutagenesis scan	FGF-R1c	NNK
ZMCIRWPARQPLFWYCGSGSDYKDDDDK	3.03E-08	2.03E-08	2.22E-08	Crude		Mutagenesis scan	FGF-R1c	NNK
ZMCIRWPARQPLFYLCGSGSDYKDDDDK	8.73E-09	4.09E-08	1.61E-08	Crude		Mutagenesis scan	FGF-R1c	NNK
ZMCIRWPARQPLGWLCGSGSDYKDDDDK	>1.00E-05	>1.00E-05	>1.00E-05	Crude		Mutagenesis scan	FGF-R1c	NNK
ZMCIRWPARQPLHWLCGSGSDYKDDDDK	>1.00E-05	>1.00E-05	>1.00E-05	Crude		Mutagenesis scan	FGF-R1c	NNK
ZMCIRWPARQPLIWLCGSGSDYKDDDDK	>1.00E-05	2.61E-08	>1.00E-05	Crude		Mutagenesis scan	FGF-R1c	NNK
ZMCIRWPARQPLKWLCGSGSDYKDDDDK	8.10E-09	5.59E-08	>1.00E-05	Crude		Mutagenesis scan	FGF-R1c	NNK
ZMCIRWPARQPLLWLCGSGSDYKDDDDK	>1.00E-05	1.23E-08	2.85E-08	Crude		Mutagenesis scan	FGF-R1c	NNK
ZMCIRWPARQPLPWLCGSGSDYKDDDDK	3.39E-08	6.67E-07	>1.00E-05	Crude		Mutagenesis scan	FGF-R1c	NNK
ZMCIRWPARQPLQWLCGSGSDYKDDDDK	>1.00E-05	>1.00E-05	6.75E-08	Crude		Mutagenesis scan	FGF-R1c	NNK
ZMCIRWPARQPLRWLCGSGSDYKDDDDK	>1.00E-05	>1.00E-05	>1.00E-05	Crude		Mutagenesis scan	FGF-R1c	NNK
ZMCIRWPARQPLSWLCGSGSDYKDDDDK	>1.00E-05	>1.00E-05	>1.00E-05	Crude		Mutagenesis scan	FGF-R1c	NNK
ZMCIRWPARQPLTWLCGSGSDYKDDDDK	>1.00E-05	3.89E-08	1.03E-07	Crude		Mutagenesis scan	FGF-R1c	NNK
ZMCIRWPARQPLVWLCGSGSDYKDDDDK	1.55E-07	5.41E-08	1.76E-07	Crude		Mutagenesis scan	FGF-R1c	NNK
ZMCIRWPARQPLWWLCGSGSDYKDDDDK	8.25E-09	1.15E-08	2.45E-08	Crude		Mutagenesis scan	FGF-R1c	NNK
ZMCIRWPARQPLYWLCGSGSDYKDDDDK	5.51E-09	1.21E-08	4.68E-08	Crude		Mutagenesis scan	FGF-R1c	NNK
ZMCIRWPARQPPFWLCGSGSDYKDDDDK	6.34E-07	4.12E-08	1.19E-07	Crude		Mutagenesis scan	FGF-R1c	NNK
ZMCIRWPARQPQFWLCGSGSDYKDDDDK	5.41E-09	6.89E-08	1.37E-08	Crude		Mutagenesis scan	FGF-R1c	NNK
ZMCIRWPARQPSFWLCGSGSDYKDDDDK	2.51E-08	1.52E-08	>1.00E-05	Crude		Mutagenesis scan	FGF-R1c	NNK
ZMCIRWPARQPVFWLCGSGSDYKDDDDK	1.44E-08	2.28E-08	1.91E-07	Crude		Mutagenesis scan	FGF-R1c	NNK
ZMCIRWPARQPWFWLCGSGSDYKDDDDK	3.20E-08	9.57E-09	5.75E-08	Crude		Mutagenesis scan	FGF-R1c	NNK
ZMCIRWPARQPYFWLCGSGSDYKDDDDK	5.59E-08	1.06E-08	3.61E-08	Crude		Mutagenesis scan	FGF-R1c	NNK
ZMCIRWPARQQLFWLCGSGSDYKDDDDK	2.13E-08	1.28E-08	1.18E-08	Crude		Mutagenesis scan	FGF-R1c	NNK

ZMCIRWPARQSLFWLCGSGSDYKDDDDK	4.51E-08	8.75E-08	2.25E-08	Crude	Mutagenesis scan	FGF-R1c	NNK
ZMCIRWPARQTLFWLCGSGSDYKDDDDK	1.63E-07	1.44E-07	2.87E-08	Crude	Mutagenesis scan	FGF-R1c	NNK
ZMCIRWPARQVLFWLCGSGSDYKDDDDK	>1.00E-05	>1.00E-05	>1.00E-05	Crude	Mutagenesis scan	FGF-R1c	NNK
ZMCIRWPARQWLFWLCGSGSDYKDDDDK	9.72E-08	>1.00E-05	2.96E-08	Crude	Mutagenesis scan	FGF-R1c	NNK
ZMCIRWPARRPLFWLCGSGSDYKDDDDK	2.56E-09	3.31E-08	4.13E-09	Crude	Mutagenesis scan	FGF-R1c	NNK
ZMCIRWPARSPLFWLCGSGSDYKDDDDK	7.82E-09	1.70E-08	1.59E-07	Crude	Mutagenesis scan	FGF-R1c	NNK
ZMCIRWPARTPLFWLCGSGSDYKDDDDK	1.02E-08	1.85E-08	8.73E-08	Crude	Mutagenesis scan	FGF-R1c	NNK
ZMCIRWPARVPLFWLCGSGSDYKDDDDK	1.11E-08	8.70E-09	7.62E-08	Crude	Mutagenesis scan	FGF-R1c	NNK
ZMCIRWPARWPLFWLCGSGSDYKDDDDK	1.49E-08	3.63E-08	2.90E-08	Crude	Mutagenesis scan	FGF-R1c	NNK
ZMCIRWPARYPLFWLCGSGSDYKDDDDK	8.87E-09	1.50E-08	2.45E-08	Crude	Mutagenesis scan	FGF-R1c	NNK
ZMCIRWPASQPLFWLCGSGSDYKDDDDK	2.26E-08	4.51E-08	>1.00E-05	Crude	Mutagenesis scan	FGF-R1c	NNK
ZMCIRWPATQPLFWLCGSGSDYKDDDDK	5.72E-08	4.60E-08	>1.00E-05	Crude	Mutagenesis scan	FGF-R1c	NNK
ZMCIRWPAVQPLFWLCGSGSDYKDDDDK	2.92E-08	3.26E-08	>1.00E-05	Crude	Mutagenesis scan	FGF-R1c	NNK
ZMCIRWPAWQPLFWLCGSGSDYKDDDDK	1.60E-08	5.76E-08	5.62E-09	Crude	Mutagenesis scan	FGF-R1c	NNK
ZMCIRWPAYQPLFWLCGSGSDYKDDDDK	2.91E-08	6.77E-08	>1.00E-05	Crude	Mutagenesis scan	FGF-R1c	NNK
ZMCIRWPDRQPLFWLCGSGSDYKDDDDK	>1.00E-05	9.91E-08	>1.00E-05	Crude	Mutagenesis scan	FGF-R1c	NNK
ZMCIRWPERQPLFWLCGSGSDYKDDDDK	>1.00E-05	3.44E-08	>1.00E-05	Crude	Mutagenesis scan	FGF-R1c	NNK
ZMCIRWPFRQPLFWLCGSGSDYKDDDDK	>1.00E-05	1.69E-07	6.58E-09	Crude	Mutagenesis scan	FGF-R1c	NNK
ZMCIRWPGRQPLFWLCGSGSDYKDDDDK	>1.00E-05	1.18E-07	6.51E-09	Crude	Mutagenesis scan	FGF-R1c	NNK
ZMCIRWPHRQPLFWLCGSGSDYKDDDDK	1.40E-08	3.11E-08	2.87E-08	Crude	Mutagenesis scan	FGF-R1c	NNK
ZMCIRWPIRQPLFWLCGSGSDYKDDDDK	1.34E-08	1.36E-08	9.88E-09	Crude	Mutagenesis scan	FGF-R1c	NNK
ZMCIRWPKRQPLFWLCGSGSDYKDDDDK	>1.00E-05	7.23E-09	1.46E-08	Crude	Mutagenesis scan	FGF-R1c	NNK
ZMCIRWPLRQPLFWLCGSGSDYKDDDDK	3.95E-08	3.39E-08	1.39E-08	Crude	Mutagenesis scan	FGF-R1c	NNK
ZMCIRWPNRQPLFWLCGSGSDYKDDDDK	4.25E-09	1.25E-08	3.59E-08	Crude	Mutagenesis scan	FGF-R1c	NNK
ZMCIRWPPRQPLFWLCGSGSDYKDDDDK	1.82E-08	1.32E-08	1.13E-09	Crude	Mutagenesis scan	FGF-R1c	NNK
ZMCIRWPQRQPLFWLCGSGSDYKDDDDK	6.27E-09	1.80E-08	3.21E-09	Crude	Mutagenesis scan	FGF-R1c	NNK
ZMCIRWPRRQPLFWLCGSGSDYKDDDDK	>1.00E-05	6.82E-09	2.19E-08	Crude	Mutagenesis scan	FGF-R1c	NNK
ZMCIRWPSRQPLFWLCGSGSDYKDDDDK	6.25E-09	2.09E-08	1.92E-09	Crude	Mutagenesis scan	FGF-R1c	NNK
ZMCIRWPTRQPLFWLCGSGSDYKDDDDK	1.61E-08	2.04E-08	2.36E-09	Crude	Mutagenesis scan	FGF-R1c	NNK
ZMCIRWPVRQPLFWLCGSGSDYKDDDDK	8.90E-09	1.68E-08	1.55E-08	Crude	Mutagenesis scan	FGF-R1c	NNK
ZMCIRWPWRQPLFWLCGSGSDYKDDDDK	2.14E-08	2.27E-07	6.97E-09	Crude	Mutagenesis scan	FGF-R1c	NNK
ZMCIRWPYRQPLFWLCGSGSDYKDDDDK	3.94E-09	2.37E-08	8.49E-09	Crude	Mutagenesis scan	FGF-R1c	NNK
ZMCIRWPAAQPLFWLCGSGSDYKDDDDK	1.32E-07	6.36E-08	>1.00E-05	Crude	Mutagenesis scan	FGF-R1c	NNK
ZMCIRWAARQPLFWLCGSGSDYKDDDDK	5.06E-08	1.42E-08	2.24E-08	Crude	Mutagenesis scan	FGF-R1c	NNK
ZMCIRYPARQPLFWLCGSGSDYKDDDDK	6.19E-09	2.59E-08	1.86E-08	Crude	Mutagenesis scan	FGF-R1c	NNK
ZMCISWPARQPLFWLCGSGSDYKDDDDK	7.28E-08	5.59E-08	3.36E-09	Crude	Mutagenesis scan	FGF-R1c	NNK
ZMCITWPARQPLFWLCGSGSDYKDDDDK	4.03E-08	1.48E-07	3.33E-09	Crude	Mutagenesis scan	FGF-R1c	NNK
ZMCIVWPARQPLFWLCGSGSDYKDDDDK	2.85E-08	8.11E-08	8.76E-09	Crude	Mutagenesis scan	FGF-R1c	NNK
ZMCIWWPARQPLFWLCGSGSDYKDDDDK	1.37E-07	2.16E-07	1.95E-09	Crude	Mutagenesis scan	FGF-R1c	NNK
ZMCIYWPARQPLFWLCGSGSDYKDDDDK	>1.00E-05	2.98E-07	1.02E-08	Crude	Mutagenesis scan	FGF-R1c	NNK
ZMCKRWPARQPLFWLCGSGSDYKDDDDK	1.91E-09	5.47E-08	9.84E-08	Crude	Mutagenesis scan	FGF-R1c	NNK
ZMCLRWPARQPLFWLCGSGSDYKDDDDK	8.73E-09	1.90E-07	3.87E-08	Crude	Mutagenesis scan	FGF-R1c	NNK
ZMCPRWPARQPLFWLCGSGSDYKDDDDK	1.53E-08	>1.00E-05	3.19E-08	Crude	Mutagenesis scan	FGF-R1c	NNK
ZMCQRWPARQPLFWLCGSGSDYKDDDDK	3.15E-09	2.69E-08	2.58E-08	Crude	Mutagenesis scan	FGF-R1c	NNK
ZMCRRWPARQPLFWLCGSGSDYKDDDDK	5.65E-09	1.20E-07	3.16E-07	Crude	Mutagenesis scan	FGF-R1c	NNK
ZMCSRWPARQPLFWLCGSGSDYKDDDDK	2.66E-09	2.47E-08	1.85E-08	Crude	Mutagenesis scan	FGF-R1c	NNK
ZMCTRWPARQPLFWLCGSGSDYKDDDDK	2.51E-09	2.92E-08	8.39E-09	Crude	Mutagenesis scan	FGF-R1c	NNK
ZMCVRWPARQPLFWLCGSGSDYKDDDDK	1.22E-08	1.61E-08	1.11E-08	Crude	Mutagenesis scan	FGF-R1c	NNK
ZMCWRWPARQPLFWLCGSGSDYKDDDDK	3.47E-09	3.28E-08	8.96E-09	Crude	Mutagenesis scan	FGF-R1c	NNK
ZMCYRWPARQPLFWLCGSGSDYKDDDDK	3.05E-09	4.93E-08	1.93E-08	Crude	Mutagenesis scan	FGF-R1c	NNK
ZMCAPFWFIRPPNKMCGSGSDYKDDDDK	>1.00E-05	>1.00E-05	>1.00E-05	Crude	Selection	FGF-R3C	NNK
ZMCDTWNLMYKLWVPCGSGSDYKDDDDK	>1.00E-05	>1.00E-05	>1.00E-05	Crude	Selection	FGF-R3C	NNK
ZMCDTWTLYQRFQTPCGSGSDYKDDDDK	>1.00E-05	>1.00E-05	>1.00E-05	Crude	Selection	FGF-R3C	NNK
ZMCDYWNMWSRNWIKCGSGSDYKDDDDK	>1.00E-05	>1.00E-05	>1.00E-05	Crude	Selection	FGF-R3C	NNK
ZMCDYWNMWSRNWIMCGSGSDYKDDDDK	>1.00E-05	>1.00E-05	5.47E-07	Crude	Selection	FGF-R3C	NNK
ZMCEMWNWYDRVWVYCGSGSDYKDDDDK	>1.00E-05	>1.00E-05	>1.00E-05	Crude	Selection	FGF-R3C	NNK
ZMCEMWSIYNREWRDCGSGSDYKDDDDK	>1.00E-05	>1.00E-05	>1.00E-05	Crude	Selection	FGF-R3C	NNK
ZMCEMWSIYNREWRDCGSGSDYKDDDDK	>1.00E-05	>1.00E-05	>1.00E-05	Crude	Selection	FGF-R3C	NNK
ZMCEVYGKYMPHWMWCGSGSDYKDDDDK	>1.00E-05	>1.00E-05	>1.00E-05	Crude	Selection	FGF-R3C	NNK
MCFFCFDRLFLCCGSGSDYKDDDDK	>1.00E-05	>1.00E-05	>1.00E-05	Crude	Selection	FGF-R3C	NNK
MCFYCFDSLFLCCGSGSDYKDDDDK	>1.00E-05	4.70E-07	>1.00E-05	Crude	Selection	FGF-R3C	NNK
ZMCHPTRLHLWLCCNCGSGSDYKDDDDK	9.40E-08	>1.00E-05	>1.00E-05	Crude	Selection	FGF-R3C	NNK
ZMCHPTRLHLWLCCNCGSGSDYKDDDDK	>1.00E-05	2.71E-07	>1.00E-05	Crude	Selection	FGF-R3C	NNK
MCHPTRLHLWLCCNCGSGSDYKDDDDK	>1.00E-05	>1.00E-05	>1.00E-05	Crude	Selection	FGF-R3C	NNK
ZMCHYCFNKLFICCGSGSDYKDDDDK	>1.00E-05	1.90E-08	>1.00E-05	Crude	Selection	FGF-R3C	NNK
MCHYCFNKLFLCCGSGSDYKDDDDK	3.75E-07	3.22E-07	>1.00E-05	Crude	Selection	FGF-R3C	NNK
ZMCHYCFNKLFLCCGSGSDYKDDDDK	5.22E-07	3.70E-08	>1.00E-05	Crude	Selection	FGF-R3C	NNK
MCHYCFNKLFLCCGSGSDYKDDDDK	>1.00E-05	4.21E-07	>1.00E-05	Crude	Selection	FGF-R3C	NNK
ZMCHYCFNKLFLCCGSGSDYKDDDDK	>1.00E-05	>1.00E-05	>1.00E-05	Crude	Selection	FGF-R3C	NNK
ZMCHYCFNRLFLCCGSGSDYKDDDDK	>1.00E-05	>1.00E-05	>1.00E-05	Crude	Selection	FGF-R3C	NNK
ZMCIFDIFMPFIICGSGSDYKDDDDK	>1.00E-05	8.10E-08	>1.00E-05	Crude	Selection	FGF-R3C	NNK
ZMCIFDIHMPFIPCGSGSDYKDDDDK	>1.00E-05	6.50E-08	>1.00E-05	Crude	Selection	FGF-R3C	NNK
MCIFDIHMPFIPCGSGSDYKDDDDK	>1.00E-05	>1.00E-05	>1.00E-05	Crude	Selection	FGF-R3C	NNK
MCIFDIHMPFIPCGSGSDYKDDDDK	>1.00E-05	>1.00E-05	>1.00E-05	Crude	Selection	FGF-R3C	NNK
ZMCIFDLFLPFIFCGSGSDYKDDDDK	>1.00E-05	6.40E-08	>1.00E-05	Crude	Selection	FGF-R3C	NNK
MCIFDNWLPFIFCGSGSDYKDDDDK	>1.00E-05	7.60E-08	>1.00E-05	Crude	Selection	FGF-R3C	NNK
ZMCIFDNWLPFIICGSGSDYKDDDDK	>1.00E-05	1.60E-08	>1.00E-05	Crude	Selection	FGF-R3C	NNK
MCIFDNWLPFIYCGSGSDYKDDDDK	>1.00E-05	9.10E-08	>1.00E-05	Crude	Selection	FGF-R3C	NNK
MCIFDNWLPFIYCGSGSDYKDDDDK	>1.00E-05	3.68E-05	>1.00E-05	Crude	Selection	FGF-R3C	NNK
MCIFDTFLPFIFCGSGSDYKDDDDK	>1.00E-05	3.70E-08	>1.00E-05	Crude	Selection	FGF-R3C	NNK
ZMCIFDTFLPFIFCGSGSDYKDDDDK	>1.00E-05	3.70E-08	>1.00E-05	Crude	Selection	FGF-R3C	NNK
MCIFDTFLPFIFCGSGSDYKDDDDK	>1.00E-05	1.00E-07	>1.00E-05	Crude	Selection	FGF-R3C	NNK
ZMCIFDTFLPFIICGSGSDYKDDDDK	>1.00E-05	5.00E-09	>1.00E-05	Crude	Selection	FGF-R3C	NNK
MCIFDTHMPFIPCGSGSDYKDDDDK	>1.00E-05	>1.00E-05	>1.00E-05	Crude	Selection	FGF-R3C	NNK
MCIWDSFVPFILCGSGSDYKDDDDK	>1.00E-05	8.50E-08	>1.00E-05	Crude	Selection	FGF-R3C	NNK
ZMCKNWTFIYTLKTLCGSGSDYKDDDDK	>1.00E-05	>1.00E-05	>1.00E-05	Crude	Selection	FGF-R3C	NNK
MCLFDLHLPFIFCGSGSDYKDDDDK	>1.00E-05	1.72E-07	>1.00E-05	Crude	Selection	FGF-R3C	NNK
MCLFDSFVPFIFCGSGSDYKDDDDK	>1.00E-05	2.90E-08	>1.00E-05	Crude	Selection	FGF-R3C	NNK
ZMCLTLKQAIKRFIECGSGSDYKDDDDK	>1.00E-05	5.40E-09	2.00E-07	Crude	Selection	FGF-R3C	NNK
MCLYDSFLPFIFCGSGSDYKDDDDK	>1.00E-05	8.40E-08	>1.00E-05	Crude	Selection	FGF-R3C	NNK
MCQQWNLYTRHWHICGSGSDYKDDDDK	>1.00E-05	>1.00E-05	>1.00E-05	Crude	Selection	FGF-R3C	NNK
ZMCQQWNLYTRHWHICGSGSDYKDDDDK	>1.00E-05	>1.00E-05	>1.00E-05	Crude	Selection	FGF-R3C	NNK
MCQQWNLYTRHWHSCGSGSDYKDDDDK	>1.00E-05	>1.00E-05	>1.00E-05	Crude	Selection	FGF-R3C	NNK
ZMCQQWNLYTRHWHSCGSGSDYKDDDDK	>1.00E-05	>1.00E-05	>1.00E-05	Crude	Selection	FGF-R3C	NNK
ZMCRYCFNKLFLCCGSGSDYKDDDDK	>1.00E-05	3.70E-08	>1.00E-05	Crude	Selection	FGF-R3C	NNK
MCSYCFNKLFLCCGSGSDYKDDDDK	>1.00E-05	2.37E-07	>1.00E-05	Crude	Selection	FGF-R3C	NNK
ZMCTETYNGYYEAYWCGSGSDYKDDDDK	>1.00E-05	>1.00E-05	>1.00E-05	Crude	Selection	FGF-R3C	NNK
MCTFDLFLPFIFCGSGSDYKDDDDK	>1.00E-05	1.77E-07	>1.00E-05	Crude	Selection	FGF-R3C	NNK
MCTFDLFLPFIFCGSGSDYKDDDDK	>1.00E-05	4.38E-07	>1.00E-05	Crude	Selection	FGF-R3C	NNK
MCTFDTHFPFITCGSGSDYKDDDDK	>1.00E-05	2.87E-07	>1.00E-05	Crude	Selection	FGF-R3C	NNK
ZMCTLGQAIQALMFIHGSGSDYKDDDDK	>1.00E-05	>1.00E-05	>1.00E-05	Crude	Selection	FGF-R3C	NNK

ZMCTLGQAIQALMFMCGSGSDYKDDDDK	>1.00E-05	5.40E-09	>1.00E-05	Crude	Selection	FGF-R3C	NNK
ZMCTLGQAIQALMFMWGSGSDYKDDDDK	>1.00E-05	>1.00E-05	>1.00E-05	Crude	Selection	FGF-R3C	NNK
ZMCTLGQAIQALMFMYGSGSDYKDDDDK	>1.00E-05	>1.00E-05	>1.00E-05	Crude	Selection	FGF-R3C	NNK
ZMCTWKQAVNAFMRKCGSGSDYKDDDDK	>1.00E-05	>1.00E-05	>1.00E-05	Crude	Selection	FGF-R3C	NNK
ZMCTWREAIAKFMEICGSGSDYKDDDDK	>1.00E-05	>1.00E-05	>1.00E-05	Crude	Selection	FGF-R3C	NNK
ZMCVFDSFLPFIFCGSGSDYKDDDDK	>1.00E-05	8.80E-09	>1.00E-05	Crude	Selection	FGF-R3C	NNK
ZMCVFDSFLPFIICGSGSDYKDDDDK	>1.00E-05	9.00E-09	>1.00E-05	Crude	Selection	FGF-R3C	NNK
MCVFDSFLPFIICGSGSDYKDDDDK	>1.00E-05	3.80E-08	>1.00E-05	Crude	Selection	FGF-R3C	NNK
MCVFDSFLPFVLCGSGSDYKDDDDK	>1.00E-05	6.00E-08	>1.00E-05	Crude	Selection	FGF-R3C	NNK
MCVFDSWLPFVFCGSGSDYKDDDDK	>1.00E-05	1.06E-06	>1.00E-05	Crude	Selection	FGF-R3C	NNK
MCVFDSWLPFVYCGSGSDYKDDDDK	>1.00E-05	6.18E-07	>1.00E-05	Crude	Selection	FGF-R3C	NNK
ZMCVWQPWWLFSNVVCGSGSDYKDDDDK	>1.00E-05	>1.00E-05	>1.00E-05	Crude	Selection	FGF-R3C	NNK
MCVYDIFVPFIICGSGSDYKDDDDK	>1.00E-05	2.90E-08	>1.00E-05	Crude	Selection	FGF-R3C	NNK
MCVYDLFLPFIYCGSGSDYKDDDDK	>1.00E-05	1.26E-07	>1.00E-05	Crude	Selection	FGF-R3C	NNK
ZMCWWVFEQYPKAMYCGSGSDYKDDDDK	>1.00E-05	>1.00E-05	>1.00E-05	Crude	Selection	FGF-R3C	NNK
MCYFDPHFIFIPCGSGSDYKDDDDK	>1.00E-05	>1.00E-05	>1.00E-05	Crude	Selection	FGF-R3C	NNK
ZMDACIWTAFNWWCLQGSGSDYKDDDDK	>1.00E-05	1.34E-08	>1.00E-05	Crude	Selection	FGF-R3C	NNK
ZMDACIWTTFNWWCLQGSGSDYKDDDDK	>1.00E-05	>1.00E-05	>1.00E-05	Crude	Selection	FGF-R3C	NNK
ZMDTWFPCVFCWWPDKGSGSDYKDDDDK	>1.00E-05	1.04E-07	>1.00E-05	Crude	Selection	FGF-R3C	NNK
ZMDWWYACETMCHWFPGSGSDYKDDDDK	>1.00E-05	1.00E-08	>1.00E-05	Crude	Selection	FGF-R3C	NNK
ZMECHVLMYHMWYPCHGSGSDYKDDDDK	>1.00E-05	>1.00E-05	>1.00E-05	Crude	Selection	FGF-R3C	NNK
ZMECHWWTGLMWHPCFGSGSDYKDDDDK	>1.00E-05	>1.00E-05	>1.00E-05	Crude	Selection	FGF-R3C	NNK
ZMETWFPCVFCWWPDKGSGSDYKDDDDK	>1.00E-05	6.20E-08	>1.00E-05	Crude	Selection	FGF-R3C	NNK
ZMETWFPCVFCWWPDKGSGSDYKDDDDK	>1.00E-05	>1.00E-05	>1.00E-05	Crude	Selection	FGF-R3C	NNK
ZMETWFPCVFCWWPDNGSGSDYKDDDDK	>1.00E-05	5.60E-09	>1.00E-05	Crude	Selection	FGF-R3C	NNK
ZMFDVCWWGVYTCVFDGSGSDYKDDDDK	>1.00E-05	>1.00E-05	8.40E-08	Crude	Selection	FGF-R3C	NNK
ZMFHMVYCGFCDAWWRGSGSDYKDDDDK	>1.00E-05	3.20E-08	>1.00E-05	Crude	Selection	FGF-R3C	NNK
ZMIDHWHCMLCHHMWKGSGSDYKDDDDK	>1.00E-05	>1.00E-05	>1.00E-05	Crude	Selection	FGF-R3C	NNK
ZMIDTWHCMLCKWIMDGSGSDYKDDDDK	>1.00E-05	>1.00E-05	>1.00E-05	Crude	Selection	FGF-R3C	NNK
ZMIFESCGPYICMTWQGSGSDYKDDDDK	>1.00E-05	1.58E-07	>1.00E-05	Crude	Selection	FGF-R3C	NNK
ZMIFESCGPYICVTWQGSGSDYKDDDDK	>1.00E-05	3.50E-09	>1.00E-05	Crude	Selection	FGF-R3C	NNK
ZMIGDMWCLWCQTWHQGSGSDYKDDDDK	>1.00E-05	>1.00E-05	>1.00E-05	Crude	Selection	FGF-R3C	NNK
ZMIVCTLMQWIQKCGCGSGSDYKDDDDK	>1.00E-05	>1.00E-05	>1.00E-05	Crude	Selection	FGF-R3C	NNK
ZMKCTLKQVVRFFWCGGSGSDYKDDDDK	>1.00E-05	>1.00E-05	>1.00E-05	Crude	Selection	FGF-R3C	NNK
ZMLMEGCGPYICVYRAGSGSDYKDDDDK	>1.00E-05	4.70E-08	>1.00E-05	Crude	Selection	FGF-R3C	NNK
ZMMMTLCGPYICIDHKGSGSDYKDDDDK	>1.00E-05	1.80E-08	1.10E-06	Crude	Selection	FGF-R3C	NNK
ZMMMTLCGPYICIDHKGSGSDYKDDDDK	>1.00E-05	5.70E-08	>1.00E-05	Crude	Selection	FGF-R3C	NNK
ZMMMTLCGPYICIDQEGSGSDYKDDDDK	>1.00E-05	3.10E-08	>1.00E-05	Crude	Selection	FGF-R3C	NNK
ZMMMTLCGPYICIDQKGSGSDYKDDDDK	>1.00E-05	7.10E-08	>1.00E-05	Crude	Selection	FGF-R3C	NNK
ZMMVCTLMQLIQKCGWGSGSDYKDDDDK	>1.00E-05	1.70E-08	>1.00E-05	Crude	Selection	FGF-R3C	NNK
ZMMVCTLMQWIQKCGWGSGSDYKDDDDK	>1.00E-05	>1.00E-05	>1.00E-05	Crude	Selection	FGF-R3C	NNK
ZMMVCTLMQWIQKCGWGSGSDYKDDDDK	>1.00E-05	>1.00E-05	>1.00E-05	Crude	Selection	FGF-R3C	NNK
ZMMVCTLMQWIQKCGYGSGSDYKDDDDK	>1.00E-05	>1.00E-05	>1.00E-05	Crude	Selection	FGF-R3C	NNK
ZMMVCTLMQWIRKCGWGSGSDYKDDDDK	>1.00E-05	>1.00E-05	>1.00E-05	Crude	Selection	FGF-R3C	NNK
ZMMWWWCPPHTMCYSVGSGSDYKDDDDK	>1.00E-05	>1.00E-05	>1.00E-05	Crude	Selection	FGF-R3C	NNK
ZMMWWWCQPHTMCYSVGSGSDYKDDDDK	>1.00E-05	1.47E-08	>1.00E-05	Crude	Selection	FGF-R3C	NNK
ZMMWWWCQPHTMCYSVGSGSDYKDDDDK	>1.00E-05	>1.00E-05	>1.00E-05	Crude	Selection	FGF-R3C	NNK
ZMPCHIGAVYTLAYCMGSGSDYKDDDDK	>1.00E-05	1.70E-08	>1.00E-05	Crude	Selection	FGF-R3C	NNK
ZMQCHWRYFYMWTPCYGSGSDYKDDDDK	>1.00E-05	>1.00E-05	>1.00E-05	Crude	Selection	FGF-R3C	NNK
ZMQCIRHKLHPFWYCTGSGSDYKDDDDK	9.00E-08	>1.00E-05	>1.00E-05	Crude	Selection	FGF-R3C	NNK
ZMRFHNCWQDYCWGYLGSGSDYKDDDDK	>1.00E-05	3.80E-08	6.90E-07	Crude	Selection	FGF-R3C	NNK
ZMTLGCVMKQMMCIIMGSGSDYKDDDDK	>1.00E-05	>1.00E-05	>1.00E-05	Crude	Selection	FGF-R3C	NNK
ZMTLGCVMKQMMCIIRGSGSDYKDDDDK	6.50E-08	>1.00E-05	>1.00E-05	Crude	Selection	FGF-R3C	NNK
ZMTLGCVMKQMMCIIRGSGSDYKDDDDK	>1.00E-05	3.53E-08	>1.00E-05	Crude	Selection	FGF-R3C	NNK
ZMTLKECIRMLWCAIHGSGSDYKDDDDK	>1.00E-05	5.90E-08	1.10E-07	Crude	Selection	FGF-R3C	NNK
ZMTLKECMWMLCRKIKGSGSDYKDDDDK	>1.00E-05	>1.00E-05	>1.00E-05	Crude	Selection	FGF-R3C	NNK
ZMTLKKCIYKMMCYILGSGSDYKDDDDK	>1.00E-05	1.10E-08	>1.00E-05	Crude	Selection	FGF-R3C	NNK
ZMTLKKCIYQMMCYILGSGSDYKDDDDK	>1.00E-05	>1.00E-05	>1.00E-05	Crude	Selection	FGF-R3C	NNK
ZMTLKQCMQKTWCHIHGSGSDYKDDDDK	>1.00E-05	2.24E-07	5.27E-07	Crude	Selection	FGF-R3C	NNK
ZMTLKQCMQKTWCLIHGSGSDYKDDDDK	>1.00E-05	>1.00E-05	>1.00E-05	Crude	Selection	FGF-R3C	NNK
ZMTLKQCMQKTWCRIHGSGSDYKDDDDK	>1.00E-05	6.50E-08	2.40E-06	Crude	Selection	FGF-R3C	NNK
ZMTLLMCVKSMYCKIHGSGSDYKDDDDK	>1.00E-05	1.78E-07	>1.00E-05	Crude	Selection	FGF-R3C	NNK
ZMTLMECIRMLWCAIHGSGSDYKDDDDK	>1.00E-05	1.02E-07	>1.00E-05	Crude	Selection	FGF-R3C	NNK
ZMTLMECIRMLWCAIHGSGSDYKDDDDK	>1.00E-05	>1.00E-05	>1.00E-05	Crude	Selection	FGF-R3C	NNK
ZMTLMECIRMLWCTIHGSGSDYKDDDDK	>1.00E-05	1.70E-08	>1.00E-05	Crude	Selection	FGF-R3C	NNK
ZMTLREHCRQCYYEWYGSGSDYKDDDDK	>1.00E-05	>1.00E-05	>1.00E-05	Crude	Selection	FGF-R3C	NNK
ZMTLREYCRQCYYEWYGSGSDYKDDDDK	>1.00E-05	>1.00E-05	>1.00E-05	Crude	Selection	FGF-R3C	NNK
ZMTMMDCKRQIYCKIWGSGSDYKDDDDK	>1.00E-05	3.50E-08	>1.00E-05	Crude	Selection	FGF-R3C	NNK
ZMVCTLMQWIQKCGWGSGSDYKDDDDK	>1.00E-05	>1.00E-05	>1.00E-05	Crude	Selection	FGF-R3C	NNK
ZMVDCLWYHYWSMCPMGSGSDYKDDDDK	>1.00E-05	>1.00E-05	>1.00E-05	Crude	Selection	FGF-R3C	NNK
ZMVVCTLMQWIQKCGWGSGSDYKDDDDK	>1.00E-05	>1.00E-05	>1.00E-05	Crude	Selection	FGF-R3C	NNK
ZMWHRNCWQHDCWGWIGSGSDYKDDDDK	>1.00E-05	1.80E-08	>1.00E-05	Crude	Selection	FGF-R3C	NNK
ZMWIKCFDDKFWCEVMGSGSDYKDDDDK	>1.00E-05	5.20E-08	>1.00E-05	Crude	Selection	FGF-R3C	NNK
ZMWIKCFDDKFWCEVMGSGSDYKDDDDK	>1.00E-05	>1.00E-05	>1.00E-05	Crude	Selection	FGF-R3C	NNK
ZMWKKCFANMYWCLYKGSGSDYKDDDDK	>1.00E-05	5.50E-09	7.70E-08	Crude	Selection	FGF-R3C	NNK
ZMWKKCFANMYWCLYKGSGSDYKDDDDK	>1.00E-05	>1.00E-05	>1.00E-05	Crude	Selection	FGF-R3C	NNK
ZMWSICDPQMGMCYDGSGSDYKDDDDK	>1.00E-05	>1.00E-05	>1.00E-05	Crude	Selection	FGF-R3C	NNK
ZMWSICDPQMGMCYILGSGSDYKDDDDK	>1.00E-05	>1.00E-05	>1.00E-05	Crude	Selection	FGF-R3C	NNK
ZMWSICDPQMGMCYILGSGSDYKDDDDK	>1.00E-05	>1.00E-05	>1.00E-05	Crude	Selection	FGF-R3C	NNK
ZMCHYCFNKLFLCCGSGSDYKDDDDK	1.05E-07	2.71E-09	>1.00E-05	Purified	Selection	FGF-R3c	NNK
ZMCIFDLFLPFIFCGSGSDYKDDDDK	>1.00E-05	9.52E-08	>1.00E-05	Purified	Selection	FGF-R3c	NNK
MCIFDIHMPFIPCGSGSDYKDDDDK	>1.00E-05	>1.00E-05	>1.00E-05	Purified	Selection	FGF-R3c	NNK
MCLFDSFVPFIFCGSGSDYKDDDDK	>1.00E-05	2.12E-08	>1.00E-05	Purified	Selection	FGF-R3c	NNK
ZMCQQWNLYTRHWHSCGSGSDYKDDDDK	>1.00E-05	>1.00E-05	>1.00E-05	Purified	Selection	FGF-R3c	NNK
ZMCHPTRLHLWLCCNCGSGSDYKDDDDK	>1.00E-05	>1.00E-05	>1.00E-05	Purified	Selection	FGF-R3c	NNK
ZMLMEGCGPYICVYRAGSGSGSDYKDDDDKK	>1.00E-05	7.59E-08	>1.00E-05	Purified	Selection	FGF-R3c	NNK
ZMTLKQCMQKTWCRIHGSGSGSDYKDDDDKK	>1.00E-05	>1.00E-05	>1.00E-05	Purified	Selection	FGF-R3c	NNK
ZMETWFPCVFCWWPDNGSGSGSDYKDDDDKK	>1.00E-05	3.23E-07	>1.00E-05	Purified	Selection	FGF-R3c	NNK
ZMRFHNCWQDYCWGYLGSGSGSDYKDDDDKK	>1.00E-05	2.77E-08	2.44E-07	Purified	Selection	FGF-R3c	NNK
ZMTLMECIRMLWCTIHGSGSGSDYKDDDDKK	>1.00E-05	>1.00E-05	3.22E-08	Purified	Selection	FGF-R3c	NNK
ZMSSPPCTLGCCLRRYGSGSGSDYKDDDDK	>1.00E-05	2.12E-09	2.78E-08	Purified	Selection	FGF-R3c	NNK
ZMCTLNFCKFCTAYKYGSGSGSDYKDDDDK	>1.00E-05	1.20E-08	1.86E-08	Purified	Selection	FGF-R3c	NNK
ZMFNCLLPFYSHTCSCGSGSGSDYKDDDDK	>1.00E-05	3.75E-08	>1.00E-05	Purified	Selection	FGF-R3c	NNK
ZMVYRCPYCLYLCLLTGSGSGSDYKDDDDK	2.18E-07	3.56E-09	4.92E-10	Purified	Selection	FGF-R3c	NNK
ZMACPIGCTNTLRYCYGSGSGSDYKDDDDK	>1.00E-05	2.22E-08	>1.00E-05	Purified	Selection	FGF-R3c	NNK
ZMTMCELIRINYGCNHGSGSGSDYKDDDDK	>1.00E-05	>1.00E-05	>1.00E-05	Purified	Selection	FGF-R3c	NNK
ZMPGEICTLKECILRLGSGSGSDYKDDDDK	>1.00E-05	3.25E-09	>1.00E-05	Purified	Selection	FGF-R3c	NNK
ZDCIFDSFVPFIFCGSGSDYKDDDDK	>1.00E-05	3.73E-08	>1.00E-05	Crude	Mutagenesis scan	FGF-R3c	NNK
ZECIFDSFVPFIFCGSGSDYKDDDDK	>1.00E-05	2.01E-08	>1.00E-05	Crude	Mutagenesis scan	FGF-R3c	NNK
ZFCIFDSFVPFIFCGSGSDYKDDDDK	>1.00E-05	1.79E-08	>1.00E-05	Crude	Mutagenesis scan	FGF-R3c	NNK
ZGCIFDSFVPFIFCGSGSDYKDDDDK	>1.00E-05	1.98E-08	>1.00E-05	Crude	Mutagenesis scan	FGF-R3c	NNK

ZHCIFDSFVPFIFCGSGSDYKDDDDK	>1.00E-05	1.47E-08	>1.00E-05	Crude	Mutagenesis scan	FGF-R3c	NNK
ZMCDFDSFVPFIFCGSGSDYKDDDDK	>1.00E-05	>1.00E-05	>1.00E-05	Crude	Mutagenesis scan	FGF-R3c	NNK
ZMCEFDSFVPFIFCGSGSDYKDDDDK	>1.00E-05	1.26E-07	>1.00E-05	Crude	Mutagenesis scan	FGF-R3c	NNK
ZMCFFDSFVPFIFCGSGSDYKDDDDK	>1.00E-05	>1.00E-05	>1.00E-05	Crude	Mutagenesis scan	FGF-R3c	NNK
ZMCFIFPVFSDFICGSGSDYKDDDDK	>1.00E-05	>1.00E-05	>1.00E-05	Crude	Mutagenesis scan	FGF-R3c	NNK
ZMCFIFPVFSDFICGSGSDYKDDDDK	>1.00E-05	>1.00E-05	>1.00E-05	Crude	Mutagenesis scan	FGF-R3c	NNK
ZMCFISDVFFPFICGSGSDYKDDDDK	>1.00E-05	>1.00E-05	>1.00E-05	Crude	Mutagenesis scan	FGF-R3c	NNK
ZMCFISDVFFPFICGSGSDYKDDDDK	>1.00E-05	>1.00E-05	>1.00E-05	Crude	Mutagenesis scan	FGF-R3c	NNK
ZMCGFDSFVPFIFCGSGSDYKDDDDK	>1.00E-05	1.20E-07	>1.00E-05	Crude	Mutagenesis scan	FGF-R3c	NNK
ZMCIADSFVPFIFCGSGSDYKDDDDK	>1.00E-05	1.37E-07	>1.00E-05	Crude	Mutagenesis scan	FGF-R3c	NNK
ZMCIDDSFVPFIFCGSGSDYKDDDDK	>1.00E-05	>1.00E-05	>1.00E-05	Crude	Mutagenesis scan	FGF-R3c	NNK
ZMCIEDSFVPFIFCGSGSDYKDDDDK	>1.00E-05	>1.00E-05	>1.00E-05	Crude	Mutagenesis scan	FGF-R3c	NNK
ZMCIFASFVPFIFCGSGSDYKDDDDK	>1.00E-05	>1.00E-05	>1.00E-05	Crude	Mutagenesis scan	FGF-R3c	NNK
ZMCIFDAFVPFIFCGSGSDYKDDDDK	>1.00E-05	4.75E-08	>1.00E-05	Crude	Mutagenesis scan	FGF-R3c	NNK
ZMCIFDFFVPFIFCGSGSDYKDDDDK	>1.00E-05	>1.00E-05	>1.00E-05	Crude	Mutagenesis scan	FGF-R3c	NNK
ZMCIFDGFVPFIFCGSGSDYKDDDDK	>1.00E-05	2.15E-07	>1.00E-05	Crude	Mutagenesis scan	FGF-R3c	NNK
ZMCIFDHFVPFIFCGSGSDYKDDDDK	>1.00E-05	6.92E-08	>1.00E-05	Crude	Mutagenesis scan	FGF-R3c	NNK
ZMCIFDIFVPFIFCGSGSDYKDDDDK	>1.00E-05	9.26E-09	>1.00E-05	Crude	Mutagenesis scan	FGF-R3c	NNK
ZMCIFDKFVPFIFCGSGSDYKDDDDK	>1.00E-05	>1.00E-05	>1.00E-05	Crude	Mutagenesis scan	FGF-R3c	NNK
ZMCIFDQFVPFIFCGSGSDYKDDDDK	>1.00E-05	3.90E-08	>1.00E-05	Crude	Mutagenesis scan	FGF-R3c	NNK
ZMCIFDRFVPFIFCGSGSDYKDDDDK	>1.00E-05	3.23E-07	>1.00E-05	Crude	Mutagenesis scan	FGF-R3c	NNK
ZMCIFDSAVPFIFCGSGSDYKDDDDK	>1.00E-05	2.09E-08	>1.00E-05	Crude	Mutagenesis scan	FGF-R3c	NNK
ZMCIFDSFAPFIFCGSGSDYKDDDDK	>1.00E-05	3.49088E-08	>1.00E-05	Crude	Mutagenesis scan	FGF-R3c	NNK
ZMCIFDSFDPFIFCGSGSDYKDDDDK	>1.00E-05	>1.00E-05	>1.00E-05	Crude	Mutagenesis scan	FGF-R3c	NNK
ZMCIFDSFEPFIFCGSGSDYKDDDDK	>1.00E-05	2.38176E-08	>1.00E-05	Crude	Mutagenesis scan	FGF-R3c	NNK
ZMCIFDSFFPFIFCGSGSDYKDDDDK	>1.00E-05	3.48972E-08	>1.00E-05	Crude	Mutagenesis scan	FGF-R3c	NNK
ZMCIFDSFGPFIFCGSGSDYKDDDDK	>1.00E-05	>1.00E-05	>1.00E-05	Crude	Mutagenesis scan	FGF-R3c	NNK
ZMCIFDSFHPFIFCGSGSDYKDDDDK	>1.00E-05	5.85614E-08	>1.00E-05	Crude	Mutagenesis scan	FGF-R3c	NNK
ZMCIFDSFIPFIFCGSGSDYKDDDDK	>1.00E-05	2.62658E-08	>1.00E-05	Crude	Mutagenesis scan	FGF-R3c	NNK
ZMCIFDSFKPFIFCGSGSDYKDDDDK	>1.00E-05	>1.00E-05	>1.00E-05	Crude	Mutagenesis scan	FGF-R3c	NNK
ZMCIFDSFLPFIFCGSGSDYKDDDDK	>1.00E-05	2.50786E-08	>1.00E-05	Crude	Mutagenesis scan	FGF-R3c	NNK
ZMCIFDSFPPFIFCGSGSDYKDDDDK	>1.00E-05	>1.00E-05	>1.00E-05	Crude	Mutagenesis scan	FGF-R3c	NNK
ZMCIFDSFQPFIFCGSGSDYKDDDDK	>1.00E-05	8.97419E-08	>1.00E-05	Crude	Mutagenesis scan	FGF-R3c	NNK
ZMCIFDSFRPFIFCGSGSDYKDDDDK	>1.00E-05	>1.00E-05	>1.00E-05	Crude	Mutagenesis scan	FGF-R3c	NNK
ZMCIFDSFSPFIFCGSGSDYKDDDDK	>1.00E-05	1.51861E-08	>1.00E-05	Crude	Mutagenesis scan	FGF-R3c	NNK
ZMCIFDSFTPFIFCGSGSDYKDDDDK	>1.00E-05	1.13404E-07	>1.00E-05	Crude	Mutagenesis scan	FGF-R3c	NNK
ZMCIFDSFVAFIFCGSGSDYKDDDDK	>1.00E-05	>1.00E-05	>1.00E-05	Crude	Mutagenesis scan	FGF-R3c	NNK
ZMCIFDSFVDFIFCGSGSDYKDDDDK	>1.00E-05	7.6323E-08	>1.00E-05	Crude	Mutagenesis scan	FGF-R3c	NNK
ZMCIFDSFVEFIFCGSGSDYKDDDDK	>1.00E-05	>1.00E-05	>1.00E-05	Crude	Mutagenesis scan	FGF-R3c	NNK
ZMCIFDSFVFFIFCGSGSDYKDDDDK	>1.00E-05	>1.00E-05	>1.00E-05	Crude	Mutagenesis scan	FGF-R3c	NNK
ZMCIFDSFVGFIFCGSGSDYKDDDDK	>1.00E-05	1.09573E-07	>1.00E-05	Crude	Mutagenesis scan	FGF-R3c	NNK
ZMCIFDSFVHFIFCGSGSDYKDDDDK	>1.00E-05	>1.00E-05	>1.00E-05	Crude	Mutagenesis scan	FGF-R3c	NNK
ZMCIFDSFVIFIFCGSGSDYKDDDDK	>1.00E-05	>1.00E-05	>1.00E-05	Crude	Mutagenesis scan	FGF-R3c	NNK
ZMCIFDSFVKFIFCGSGSDYKDDDDK	>1.00E-05	>1.00E-05	>1.00E-05	Crude	Mutagenesis scan	FGF-R3c	NNK
ZMCIFDSFVLFIFCGSGSDYKDDDDK	>1.00E-05	>1.00E-05	>1.00E-05	Crude	Mutagenesis scan	FGF-R3c	NNK
ZMCIFDSFVPAIFCGSGSDYKDDDDK	>1.00E-05	8.90901E-08	>1.00E-05	Crude	Mutagenesis scan	FGF-R3c	NNK
ZMCIFDSFVPDIFCGSGSDYKDDDDK	>1.00E-05	>1.00E-05	>1.00E-05	Crude	Mutagenesis scan	FGF-R3c	NNK
ZMCIFDSFVPEIFCGSGSDYKDDDDK	>1.00E-05	>1.00E-05	>1.00E-05	Crude	Mutagenesis scan	FGF-R3c	NNK
ZMCIFDSFVPFAFCGSGSDYKDDDDK	>1.00E-05	2.9764E-07	>1.00E-05	Crude	Mutagenesis scan	FGF-R3c	NNK
ZMCIFDSFVPFDFCGSGSDYKDDDDK	>1.00E-05	>1.00E-05	>1.00E-05	Crude	Mutagenesis scan	FGF-R3c	NNK
ZMCIFDSFVPFEFCGSGSDYKDDDDK	>1.00E-05	>1.00E-05	>1.00E-05	Crude	Mutagenesis scan	FGF-R3c	NNK
ZMCIFDSFVPFFFCGSGSDYKDDDDK	>1.00E-05	>1.00E-05	>1.00E-05	Crude	Mutagenesis scan	FGF-R3c	NNK
ZMCIFDSFVPFGFCGSGSDYKDDDDK	>1.00E-05	>1.00E-05	>1.00E-05	Crude	Mutagenesis scan	FGF-R3c	NNK
ZMCIFDSFVPFHFCGSGSDYKDDDDK	>1.00E-05	>1.00E-05	>1.00E-05	Crude	Mutagenesis scan	FGF-R3c	NNK
ZMCIFDSFVPFIACGSGSDYKDDDDK	>1.00E-05	1.02254E-07	>1.00E-05	Crude	Mutagenesis scan	FGF-R3c	NNK
ZMCIFDSFVPFIDCGSGSDYKDDDDK	>1.00E-05	>1.00E-05	>1.00E-05	Crude	Mutagenesis scan	FGF-R3c	NNK
ZMCIFDSFVPFIECGSGSDYKDDDDK	>1.00E-05	3.32074E-07	>1.00E-05	Crude	Mutagenesis scan	FGF-R3c	NNK
ZMCIFDSFVPFIFCGSGSDYKDDDDK	>1.00E-05	1.86E-08	>1.00E-05	Crude	Mutagenesis scan	FGF-R3c	NNK
ZMCIFDSFVPFIGCGSGSDYKDDDDK	>1.00E-05	>1.00E-05	>1.00E-05	Crude	Mutagenesis scan	FGF-R3c	NNK
ZMCIFDSFVPFIHCGSGSDYKDDDDK	>1.00E-05	>1.00E-05	>1.00E-05	Crude	Mutagenesis scan	FGF-R3c	NNK
ZMCIFDSFVPFIICGSGSDYKDDDDK	>1.00E-05	2.41918E-08	>1.00E-05	Crude	Mutagenesis scan	FGF-R3c	NNK
ZMCIFDSFVPFIKCGSGSDYKDDDDK	>1.00E-05	4.27651E-08	>1.00E-05	Crude	Mutagenesis scan	FGF-R3c	NNK
ZMCIFDSFVPFILCGSGSDYKDDDDK	>1.00E-05	2.94643E-08	>1.00E-05	Crude	Mutagenesis scan	FGF-R3c	NNK
ZMCIFDSFVPFIPCGSGSDYKDDDDK	>1.00E-05	>1.00E-05	>1.00E-05	Crude	Mutagenesis scan	FGF-R3c	NNK
ZMCIFDSFVPFIQCGSGSDYKDDDDK	>1.00E-05	5.20336E-08	>1.00E-05	Crude	Mutagenesis scan	FGF-R3c	NNK
ZMCIFDSFVPFIRCGSGSDYKDDDDK	>1.00E-05	4.03114E-08	>1.00E-05	Crude	Mutagenesis scan	FGF-R3c	NNK
ZMCIFDSFVPFISCGSGSDYKDDDDK	>1.00E-05	1.11E-07	>1.00E-05	Crude	Mutagenesis scan	FGF-R3c	NNK
ZMCIFDSFVPFITCGSGSDYKDDDDK	>1.00E-05	>1.00E-05	>1.00E-05	Crude	Mutagenesis scan	FGF-R3c	NNK
ZMCIFDSFVPFIVCGSGSDYKDDDDK	>1.00E-05	2.69415E-08	>1.00E-05	Crude	Mutagenesis scan	FGF-R3c	NNK
ZMCIFDSFVPFIWCGSGSDYKDDDDK	>1.00E-05	1.15363E-07	>1.00E-05	Crude	Mutagenesis scan	FGF-R3c	NNK
ZMCIFDSFVPFIYCGSGSDYKDDDDK	>1.00E-05	6.79689E-08	>1.00E-05	Crude	Mutagenesis scan	FGF-R3c	NNK
ZMCIFDSFVPFKFCGSGSDYKDDDDK	>1.00E-05	>1.00E-05	>1.00E-05	Crude	Mutagenesis scan	FGF-R3c	NNK
ZMCIFDSFVPFLFCGSGSDYKDDDDK	>1.00E-05	1.3E-07	>1.00E-05	Crude	Mutagenesis scan	FGF-R3c	NNK
ZMCIFDSFVPFPFCGSGSDYKDDDDK	>1.00E-05	>1.00E-05	>1.00E-05	Crude	Mutagenesis scan	FGF-R3c	NNK
ZMCIFDSFVPFQFCGSGSDYKDDDDK	>1.00E-05	>1.00E-05	>1.00E-05	Crude	Mutagenesis scan	FGF-R3c	NNK
ZMCIFDSFVPFRFCGSGSDYKDDDDK	>1.00E-05	>1.00E-05	>1.00E-05	Crude	Mutagenesis scan	FGF-R3c	NNK
ZMCIFDSFVPFSFCGSGSDYKDDDDK	>1.00E-05	>1.00E-05	>1.00E-05	Crude	Mutagenesis scan	FGF-R3c	NNK
ZMCIFDSFVPFTFCGSGSDYKDDDDK	>1.00E-05	9.53735E-08	>1.00E-05	Crude	Mutagenesis scan	FGF-R3c	NNK
ZMCIFDSFVPFVFCGSGSDYKDDDDK	>1.00E-05	3.93538E-08	>1.00E-05	Crude	Mutagenesis scan	FGF-R3c	NNK
ZMCIFDSFVPFWFCGSGSDYKDDDDK	>1.00E-05	>1.00E-05	>1.00E-05	Crude	Mutagenesis scan	FGF-R3c	NNK
ZMCIFDSFVPFYFCGSGSDYKDDDDK	>1.00E-05	>1.00E-05	>1.00E-05	Crude	Mutagenesis scan	FGF-R3c	NNK
ZMCIFDSFVPGIFCGSGSDYKDDDDK	>1.00E-05	>1.00E-05	>1.00E-05	Crude	Mutagenesis scan	FGF-R3c	NNK
ZMCIFDSFVPHIFCGSGSDYKDDDDK	>1.00E-05	>1.00E-05	>1.00E-05	Crude	Mutagenesis scan	FGF-R3c	NNK
ZMCIFDSFVPIIFCGSGSDYKDDDDK	>1.00E-05	>1.00E-05	>1.00E-05	Crude	Mutagenesis scan	FGF-R3c	NNK
ZMCIFDSFVPKIFCGSGSDYKDDDDK	>1.00E-05	6.95518E-08	>1.00E-05	Crude	Mutagenesis scan	FGF-R3c	NNK
ZMCIFDSFVPLIFCGSGSDYKDDDDK	>1.00E-05	>1.00E-05	>1.00E-05	Crude	Mutagenesis scan	FGF-R3c	NNK
ZMCIFDSFVPPIFCGSGSDYKDDDDK	>1.00E-05	2.84383E-07	>1.00E-05	Crude	Mutagenesis scan	FGF-R3c	NNK
ZMCIFDSFVPQIFCGSGSDYKDDDDK	>1.00E-05	>1.00E-05	>1.00E-05	Crude	Mutagenesis scan	FGF-R3c	NNK
ZMCIFDSFVPRIFCGSGSDYKDDDDK	>1.00E-05	>1.00E-05	>1.00E-05	Crude	Mutagenesis scan	FGF-R3c	NNK
ZMCIFDSFVPSIFCGSGSDYKDDDDK	>1.00E-05	>1.00E-05	>1.00E-05	Crude	Mutagenesis scan	FGF-R3c	NNK
ZMCIFDSFVPTIFCGSGSDYKDDDDK	>1.00E-05	2.62521E-07	>1.00E-05	Crude	Mutagenesis scan	FGF-R3c	NNK
ZMCIFDSFVPVIFCGSGSDYKDDDDK	>1.00E-05	>1.00E-05	>1.00E-05	Crude	Mutagenesis scan	FGF-R3c	NNK
ZMCIFDSFVPWIFCGSGSDYKDDDDK	>1.00E-05	1.19746E-07	>1.00E-05	Crude	Mutagenesis scan	FGF-R3c	NNK
ZMCIFDSFVPYIFCGSGSDYKDDDDK	>1.00E-05	8.53341E-08	>1.00E-05	Crude	Mutagenesis scan	FGF-R3c	NNK
ZMCIFDSFVQFIFCGSGSDYKDDDDK	>1.00E-05	>1.00E-05	>1.00E-05	Crude	Mutagenesis scan	FGF-R3c	NNK
ZMCIFDSFVRFIFCGSGSDYKDDDDK	>1.00E-05	>1.00E-05	>1.00E-05	Crude	Mutagenesis scan	FGF-R3c	NNK
ZMCIFDSFVSFIFCGSGSDYKDDDDK	>1.00E-05	>1.00E-05	>1.00E-05	Crude	Mutagenesis scan	FGF-R3c	NNK
ZMCIFDSFVTFIFCGSGSDYKDDDDK	>1.00E-05	>1.00E-05	>1.00E-05	Crude	Mutagenesis scan	FGF-R3c	NNK
ZMCIFDSFVVFIFCGSGSDYKDDDDK	>1.00E-05	>1.00E-05	>1.00E-05	Crude	Mutagenesis scan	FGF-R3c	NNK
ZMCIFDSFVWFIFCGSGSDYKDDDDK	>1.00E-05	>1.00E-05	>1.00E-05	Crude	Mutagenesis scan	FGF-R3c	NNK
ZMCIFDSFVYFIFCGSGSDYKDDDDK	>1.00E-05	>1.00E-05	>1.00E-05	Crude	Mutagenesis scan	FGF-R3c	NNK

ZMCIFDSFWPFIFCGSGSDYKDDDDK	>1.00E-05	6.4293E-08	>1.00E-05	Crude	Mutagenesis scan	FGF-R3c	NNK
ZMCIFDSFYPFIFCGSGSDYKDDDDK	>1.00E-05	2.70148E-08	>1.00E-05	Crude	Mutagenesis scan	FGF-R3c	NNK
ZMCIFDSGVPFIFCGSGSDYKDDDDK	>1.00E-05	1.69E-07	>1.00E-05	Crude	Mutagenesis scan	FGF-R3c	NNK
ZMCIFDSHVPFIFCGSGSDYKDDDDK	>1.00E-05	4.86E-08	>1.00E-05	Crude	Mutagenesis scan	FGF-R3c	NNK
ZMCIFDSIVPFIFCGSGSDYKDDDDK	>1.00E-05	1.04026E-07	>1.00E-05	Crude	Mutagenesis scan	FGF-R3c	NNK
ZMCIFDSKVPFIFCGSGSDYKDDDDK	>1.00E-05	8.36063E-08	>1.00E-05	Crude	Mutagenesis scan	FGF-R3c	NNK
ZMCIFDSLVPFIFCGSGSDYKDDDDK	>1.00E-05	4.12172E-08	>1.00E-05	Crude	Mutagenesis scan	FGF-R3c	NNK
ZMCIFDSPVPFIFCGSGSDYKDDDDK	>1.00E-05	>1.00E-05	>1.00E-05	Crude	Mutagenesis scan	FGF-R3c	NNK
ZMCIFDSQVPFIFCGSGSDYKDDDDK	>1.00E-05	>1.00E-05	>1.00E-05	Crude	Mutagenesis scan	FGF-R3c	NNK
ZMCIFDSRVPFIFCGSGSDYKDDDDK	>1.00E-05	>1.00E-05	>1.00E-05	Crude	Mutagenesis scan	FGF-R3c	NNK
ZMCIFDSSVPFIFCGSGSDYKDDDDK	>1.00E-05	>1.00E-05	>1.00E-05	Crude	Mutagenesis scan	FGF-R3c	NNK
ZMCIFDSTVPFIFCGSGSDYKDDDDK	>1.00E-05	7.83722E-08	>1.00E-05	Crude	Mutagenesis scan	FGF-R3c	NNK
ZMCIFDSVFPFIFCGSGSDYKDDDDK	>1.00E-05	>1.00E-05	>1.00E-05	Crude	Mutagenesis scan	FGF-R3c	NNK
ZMCIFDSVFPFIFCGSGSDYKDDDDK	>1.00E-05	>1.00E-05	>1.00E-05	Crude	Mutagenesis scan	FGF-R3c	NNK
ZMCIFDSVVPFIFCGSGSDYKDDDDK	>1.00E-05	1.30612E-07	>1.00E-05	Crude	Mutagenesis scan	FGF-R3c	NNK
ZMCIFDSWVPFIFCGSGSDYKDDDDK	>1.00E-05	1.49541E-08	>1.00E-05	Crude	Mutagenesis scan	FGF-R3c	NNK
ZMCIFDSYVPFIFCGSGSDYKDDDDK	>1.00E-05	2.95844E-08	>1.00E-05	Crude	Mutagenesis scan	FGF-R3c	NNK
ZMCIFDVFVPFIFCGSGSDYKDDDDK	>1.00E-05	3.36E-08	>1.00E-05	Crude	Mutagenesis scan	FGF-R3c	NNK
ZMCIFDWFVPFIFCGSGSDYKDDDDK	>1.00E-05	4.34E-08	>1.00E-05	Crude	Mutagenesis scan	FGF-R3c	NNK
ZMCIFDYFVPFIFCGSGSDYKDDDDK	>1.00E-05	6.13E-08	>1.00E-05	Crude	Mutagenesis scan	FGF-R3c	NNK
ZMCIFFSFVPFIFCGSGSDYKDDDDK	>1.00E-05	1.71E-08	>1.00E-05	Crude	Mutagenesis scan	FGF-R3c	NNK
ZMCIFHSFVPFIFCGSGSDYKDDDDK	>1.00E-05	1.02E-07	>1.00E-05	Crude	Mutagenesis scan	FGF-R3c	NNK
ZMCIFISFVPFIFCGSGSDYKDDDDK	>1.00E-05	>1.00E-05	>1.00E-05	Crude	Mutagenesis scan	FGF-R3c	NNK
ZMCIFKSFVPFIFCGSGSDYKDDDDK	>1.00E-05	>1.00E-05	>1.00E-05	Crude	Mutagenesis scan	FGF-R3c	NNK
ZMCIFLSFVPFIFCGSGSDYKDDDDK	>1.00E-05	>1.00E-05	>1.00E-05	Crude	Mutagenesis scan	FGF-R3c	NNK
ZMCIFPSFVPFIFCGSGSDYKDDDDK	>1.00E-05	>1.00E-05	>1.00E-05	Crude	Mutagenesis scan	FGF-R3c	NNK
ZMCIFQSFVPFIFCGSGSDYKDDDDK	>1.00E-05	>1.00E-05	>1.00E-05	Crude	Mutagenesis scan	FGF-R3c	NNK
ZMCIFRSFVPFIFCGSGSDYKDDDDK	>1.00E-05	>1.00E-05	>1.00E-05	Crude	Mutagenesis scan	FGF-R3c	NNK
ZMCIFSSFVPFIFCGSGSDYKDDDDK	>1.00E-05	>1.00E-05	>1.00E-05	Crude	Mutagenesis scan	FGF-R3c	NNK
ZMCIFVFIFSPDFCGSGSDYKDDDDK	>1.00E-05	>1.00E-05	>1.00E-05	Crude	Mutagenesis scan	FGF-R3c	NNK
ZMCIFVSFVPFIFCGSGSDYKDDDDK	>1.00E-05	>1.00E-05	>1.00E-05	Crude	Mutagenesis scan	FGF-R3c	NNK
ZMCIFWSFVPFIFCGSGSDYKDDDDK	>1.00E-05	>1.00E-05	>1.00E-05	Crude	Mutagenesis scan	FGF-R3c	NNK
ZMCIFYSFVPFIFCGSGSDYKDDDDK	>1.00E-05	>1.00E-05	>1.00E-05	Crude	Mutagenesis scan	FGF-R3c	NNK
ZMCIIDSFVPFIFCGSGSDYKDDDDK	>1.00E-05	>1.00E-05	>1.00E-05	Crude	Mutagenesis scan	FGF-R3c	NNK
ZMCIKDSFVPFIFCGSGSDYKDDDDK	>1.00E-05	9.08E-08	>1.00E-05	Crude	Mutagenesis scan	FGF-R3c	NNK
ZMCILDSFVPFIFCGSGSDYKDDDDK	>1.00E-05	>1.00E-05	>1.00E-05	Crude	Mutagenesis scan	FGF-R3c	NNK
ZMCIPDSFVPFIFCGSGSDYKDDDDK	>1.00E-05	8.68E-08	>1.00E-05	Crude	Mutagenesis scan	FGF-R3c	NNK
ZMCIQDSFVPFIFCGSGSDYKDDDDK	>1.00E-05	>1.00E-05	>1.00E-05	Crude	Mutagenesis scan	FGF-R3c	NNK
ZMCIRDSFVPFIFCGSGSDYKDDDDK	>1.00E-05	>1.00E-05	>1.00E-05	Crude	Mutagenesis scan	FGF-R3c	NNK
ZMCISDSFVPFIFCGSGSDYKDDDDK	>1.00E-05	>1.00E-05	>1.00E-05	Crude	Mutagenesis scan	FGF-R3c	NNK
ZMCIVDSFVPFIFCGSGSDYKDDDDK	>1.00E-05	>1.00E-05	>1.00E-05	Crude	Mutagenesis scan	FGF-R3c	NNK
ZMCIYDSFVPFIFCGSGSDYKDDDDK	>1.00E-05	2.43E-08	>1.00E-05	Crude	Mutagenesis scan	FGF-R3c	NNK
ZMCKFDSFVPFIFCGSGSDYKDDDDK	>1.00E-05	>1.00E-05	>1.00E-05	Crude	Mutagenesis scan	FGF-R3c	NNK
ZMCLFDSFVPFIFCGSGSDYKDDDDK	>1.00E-05	1.23E-08	>1.00E-05	Crude	Mutagenesis scan	FGF-R3c	NNK
ZMCPFDSFVPFIFCGSGSDYKDDDDK	>1.00E-05	>1.00E-05	>1.00E-05	Crude	Mutagenesis scan	FGF-R3c	NNK
ZMCQFDSFVPFIFCGSGSDYKDDDDK	>1.00E-05	1.37E-07	>1.00E-05	Crude	Mutagenesis scan	FGF-R3c	NNK
ZMCRFDSFVPFIFCGSGSDYKDDDDK	>1.00E-05	4.44E-07	>1.00E-05	Crude	Mutagenesis scan	FGF-R3c	NNK
ZMCSFDSFVPFIFCGSGSDYKDDDDK	>1.00E-05	1.90E-08	>1.00E-05	Crude	Mutagenesis scan	FGF-R3c	NNK
ZMCTFDSFVPFIFCGSGSDYKDDDDK	>1.00E-05	3.87E-08	>1.00E-05	Crude	Mutagenesis scan	FGF-R3c	NNK
ZMCVFDSFVPFIFCGSGSDYKDDDDK	>1.00E-05	2.55E-08	>1.00E-05	Crude	Mutagenesis scan	FGF-R3c	NNK
ZPCIFDSFVPFIFCGSGSDYKDDDDK	>1.00E-05	1.11E-08	>1.00E-05	Crude	Mutagenesis scan	FGF-R3c	NNK
ZQCIFDSFVPFIFCGSGSDYKDDDDK	>1.00E-05	1.86E-08	>1.00E-05	Crude	Mutagenesis scan	FGF-R3c	NNK
ZSCIFDSFVPFIFCGSGSDYKDDDDK	>1.00E-05	1.28E-08	>1.00E-05	Crude	Mutagenesis scan	FGF-R3c	NNK
ZTCIFDSFVPFIFCGSGSDYKDDDDK	>1.00E-05	8.98E-09	>1.00E-05	Crude	Mutagenesis scan	FGF-R3c	NNK
ZWCIFDSFVPFIFCGSGSDYKDDDDK	>1.00E-05	1.18E-08	>1.00E-05	Crude	Mutagenesis scan	FGF-R3c	NNK
ZMCAPKWLIKVLSCGSGSDYKDDDDK	>1.00E-05	>1.00E-05	>1.00E-05	Crude	Selection	FGF-R4	NNK
ZMCDAHRLLFELICGSGSDYKDDDDK	>1.00E-05	>1.00E-05	>1.00E-05	Crude	Selection	FGF-R4	NNK
ZMCDCHPLLCFLLCGSGSDYKDDDDK	>1.00E-05	>1.00E-05	>1.00E-05	Crude	Selection	FGF-R4	NNK
ZMCDCLHVVCFLVCGSGSDYKDDDDK	>1.00E-05	>1.00E-05	>1.00E-05	Crude	Selection	FGF-R4	NNK
ZMCDCVWPLCWMTCGSGSDYKDDDDK	>1.00E-05	>1.00E-05	>1.00E-05	Crude	Selection	FGF-R4	NNK
ZMCDVLLYKLFRYCGSGSDYKDDDDK	1.11E-07	1.93E-06	3.20E-08	Crude	Selection	FGF-R4	NNK
ZMCDVLVYKVLWACGSGSDYKDDDDK	>1.00E-05	>1.00E-05	7.90E-08	Crude	Selection	FGF-R4	NNK
ZMCDWPRWLVKVVCGSGSDYKDDDDK	>1.00E-05	>1.00E-05	2.61E-07	Crude	Selection	FGF-R4	NNK
ZMCEPWITIYKVLKKCGSGSDYKDDDDK	>1.00E-05	1.25E-06	2.87E-07	Crude	Selection	FGF-R4	NNK
ZMCERFVTYYKLFCGSGSDYKDDDDK	>1.00E-05	>1.00E-05	>1.00E-05	Crude	Selection	FGF-R4	NNK
ZMCFALLYKILSRCGSGSDYKDDDDK	>1.00E-05	>1.00E-05	>1.00E-05	Crude	Selection	FGF-R4	NNK
ZMCFASLWYRVQCCGSGSDYKDDDDK	>1.00E-05	>1.00E-05	3.59E-07	Crude	Selection	FGF-R4	NNK
ZMCFKHPWFFAWLCGSGSDYKDDDDK	>1.00E-05	>1.00E-05	3.50E-08	Crude	Selection	FGF-R4	NNK
ZMCFVTLYKLLKYCGSGSDYKDDDDK	>1.00E-05	1.63E-06	>1.00E-05	Crude	Selection	FGF-R4	NNK
ZMCFWPIWYKFMACGSGSDYKDDDDK	>1.00E-05	>1.00E-05	>1.00E-05	Crude	Selection	FGF-R4	NNK
ZMCFWRFFPALLACGSGSDYKDDDDK	>1.00E-05	>1.00E-05	>1.00E-05	Crude	Selection	FGF-R4	NNK
ZMCGHFSITWFKWCGSGSDYKDDDDK	>1.00E-05	>1.00E-05	>1.00E-05	Crude	Selection	FGF-R4	NNK
ZMCGVLLYKVMRVCGSGSDYKDDDDK	>1.00E-05	>1.00E-05	1.24E-07	Crude	Selection	FGF-R4	NNK
ZMCHCFPIVCWFECGSGSDYKDDDDK	>1.00E-05	>1.00E-05	>1.00E-05	Crude	Selection	FGF-R4	NNK
ZMCHCFPTICWLECGSGSDYKDDDDK	>1.00E-05	>1.00E-05	>1.00E-05	Crude	Selection	FGF-R4	NNK
ZMCHCFPTICWLVCGSGSDYKDDDDK	>1.00E-05	>1.00E-05	>1.00E-05	Crude	Selection	FGF-R4	NNK
ZMCHITIYKILRSCGSGSDYKDDDDK	>1.00E-05	>1.00E-05	8.33E-07	Crude	Selection	FGF-R4	NNK
ZMCHITIYKILRYCGSGSDYKDDDDK	>1.00E-05	>1.00E-05	1.24E-07	Crude	Selection	FGF-R4	NNK
ZMCHLAYYLAFRSCGSGSDYKDDDDK	>1.00E-05	>1.00E-05	5.80E-08	Crude	Selection	FGF-R4	NNK
ZMCHVILWRSWRYCGSGSDYKDDDDK	>1.00E-05	>1.00E-05	9.30E-08	Crude	Selection	FGF-R4	NNK
ZMCHYISDWLCGSGSDYKDDDDK	>1.00E-05	2.74E-05	>1.00E-05	Crude	Selection	FGF-R4	NNK
ZMCIAIYKLMKMHCGSGSDYKDDDDK	>1.00E-05	>1.00E-05	>1.00E-05	Crude	Selection	FGF-R4	NNK
ZMCIHRLSDFWVVCGSGSDYKDDDDK	1.90E-08	4.90E-08	4.10E-08	Crude	Selection	FGF-R4	NNK
ZMCIKMFLAHKRFCGSGSDYKDDDDK	>1.00E-05	>1.00E-05	9.70E-08	Crude	Selection	FGF-R4	NNK
ZMCIKTFLLWRSSCGSGSDYKDDDDK	>1.00E-05	>1.00E-05	>1.00E-05	Crude	Selection	FGF-R4	NNK
ZMCILLYKVLRGRCGSGSDYKDDDDK	>1.00E-05	>1.00E-05	1.28E-07	Crude	Selection	FGF-R4	NNK
ZMCIYRLSDFWAVCGSGSDYKDDDDK	2.10E-08	4.90E-08	1.33E-07	Crude	Selection	FGF-R4	NNK
ZMCIYRLSDFWVECGSGSDYKDDDDK	4.70E-08	2.81E-07	4.50E-08	Crude	Selection	FGF-R4	NNK
ZMCIYRLSDFWVVCGSGSDYKDDDDK	1.90E-08	1.20E-08	4.70E-08	Crude	Selection	FGF-R4	NNK
ZMCKKHLLYYLIACGSGSDYKDDDDK	>1.00E-05	>1.00E-05	1.15E-07	Crude	Selection	FGF-R4	NNK
ZMCLAYYLAFHSLCGSGSDYKDDDDK	>1.00E-05	2.56E-06	1.26E-07	Crude	Selection	FGF-R4	NNK
ZMCLISIYKVLKHCGSGSDYKDDDDK	>1.00E-05	>1.00E-05	>1.00E-05	Crude	Selection	FGF-R4	NNK
ZMCLVSIYKIMRTCGSGSDYKDDDDK	>1.00E-05	6.12E-06	1.93E-07	Crude	Selection	FGF-R4	NNK
ZMCNIILRCGSGSDYKDDDDK	>1.00E-05	>1.00E-05	>1.00E-05	Crude	Selection	FGF-R4	NNK
ZMCNIILYKIMKNRCGSGSDYKDDDDK	>1.00E-05	>1.00E-05	2.10E-07	Crude	Selection	FGF-R4	NNK
ZMCNIILYKIMKNRCGSGSDYKDDDDK	>1.00E-05	>1.00E-05	>1.00E-05	Crude	Selection	FGF-R4	NNK
ZMCNILLYKILSVCGSGSDYKDDDDK	>1.00E-05	>1.00E-05	1.05E-07	Crude	Selection	FGF-R4	NNK
ZMCNVTIWKVLRLCGSGSDYKDDDDK	>1.00E-05	>1.00E-05	3.50E-07	Crude	Selection	FGF-R4	NNK
ZMCPERLFYWLLVCGSGSDYKDDDDK	>1.00E-05	>1.00E-05	6.80E-08	Crude	Selection	FGF-R4	NNK
ZMCPKWFILWRQLCGSGSDYKDDDDK	>1.00E-05	>1.00E-05	>1.00E-05	Crude	Selection	FGF-R4	NNK
ZMCPKWLIKVLLNCGSGSDYKDDDDK	>1.00E-05	>1.00E-05	4.19E-06	Crude	Selection	FGF-R4	NNK

ZMCPKWLLKWFLACGSGSDYKDDDDK	>1.00E-05	>1.00E-05	>1.00E-05	Crude	Selection	FGF-R4	NNK
ZMCPKYFLLWRLMTCGSGSDYKDDDDK	>1.00E-05	>1.00E-05	1.84E-06	Crude	Selection	FGF-R4	NNK
ZMCPRWIYYYMLHCGSGSDYKDDDDK	>1.00E-05	1.32E-05	1.39E-07	Crude	Selection	FGF-R4	NNK
ZMCPRYFWYHYLLCGSGSDYKDDDDK	>1.00E-05	>1.00E-05	>1.00E-05	Crude	Selection	FGF-R4	NNK
ZMCPVIIYKILSHCGSGSDYKDDDDK	>1.00E-05	>1.00E-05	1.98E-07	Crude	Selection	FGF-R4	NNK
ZMCPVLIYKLWRTCGSGSDYKDDDDK	>1.00E-05	4.33E-06	9.40E-08	Crude	Selection	FGF-R4	NNK
ZMCQFRFSKFWLSCGSGSDYKDDDDK	>1.00E-05	>1.00E-05	>1.00E-05	Crude	Selection	FGF-R4	NNK
ZMCQFTYSKFFFCGSGSDYKDDDDK	>1.00E-05	>1.00E-05	>1.00E-05	Crude	Selection	FGF-R4	NNK
ZMCRDFWHLWFRVCGSGSDYKDDDDK	2.61E-07	>1.00E-05	>1.00E-05	Crude	Selection	FGF-R4	NNK
ZMCRELLYYLLQRCGSGSDYKDDDDK	>1.00E-05	>1.00E-05	5.90E-08	Crude	Selection	FGF-R4	NNK
ZMCRFIIKAFLNLCGSGSDYKDDDDK	>1.00E-05	>1.00E-05	>1.00E-05	Crude	Selection	FGF-R4	NNK
ZMCSCFPPLCFLICGSGSDYKDDDDK	>1.00E-05	>1.00E-05	>1.00E-05	Crude	Selection	FGF-R4	NNK
ZMCSCPFPLCELMCGSGSDYKDDDDK	>1.00E-05	>1.00E-05	>1.00E-05	Crude	Selection	FGF-R4	NNK
ZMCSHCWLFKVLLMGSGSDYKDDDDK	>1.00E-05	>1.00E-05	1.80E-07	Crude	Selection	FGF-R4	NNK
ZMCSHMWLFKVLLCGSGSDYKDDDDK	>1.00E-05	>1.00E-05	>1.00E-05	Crude	Selection	FGF-R4	NNK
ZMCSILLYKVLSQCGSGSDYKDDDDK	>1.00E-05	>1.00E-05	8.40E-08	Crude	Selection	FGF-R4	NNK
ZMCSILLYKVLSRCGSGSDYKDDDDK	>1.00E-05	>1.00E-05	>1.00E-05	Crude	Selection	FGF-R4	NNK
ZMCSITIYKLLRFCGSGSDYKDDDDK	>1.00E-05	5.91E-07	1.92E-07	Crude	Selection	FGF-R4	NNK
ZMCVAFYRAFMASCGSGSDYKDDDDK	>1.00E-05	>1.00E-05	2.94E-07	Crude	Selection	FGF-R4	NNK
ZMCVFWTGMSIWICGSGSDYKDDDDK	>1.00E-05	>1.00E-05	>1.00E-05	Crude	Selection	FGF-R4	NNK
ZMCVKCWLAYIRTMGSGSDYKDDDDK	>1.00E-05	>1.00E-05	1.40E-08	Crude	Selection	FGF-R4	NNK
ZMCVKMWLAYIRTCGSGSDYKDDDDK	>1.00E-05	>1.00E-05	>1.00E-05	Crude	Selection	FGF-R4	NNK
ZMCVKYFTLWKNWCGSGSDYKDDDDK	>1.00E-05	>1.00E-05	5.34E-07	Crude	Selection	FGF-R4	NNK
ZMCVKYYLIMKSLCGSGSDYKDDDDK	>1.00E-05	>1.00E-05	>1.00E-05	Crude	Selection	FGF-R4	NNK
ZMCVSYYRAYFAHCGSGSDYKDDDDK	>1.00E-05	3.00E-06	1.02E-07	Crude	Selection	FGF-R4	NNK
ZMCVTWYKVTKLRCGSGSDYKDDDDK	>1.00E-05	>1.00E-05	5.70E-08	Crude	Selection	FGF-R4	NNK
ZMCVVCYKAFVMCCGSGSDYKDDDDK	>1.00E-05	>1.00E-05	3.33E-06	Crude	Selection	FGF-R4	NNK
ZMCVWPKWLTKLICGSGSDYKDDDDK	>1.00E-05	>1.00E-05	1.74E-07	Crude	Selection	FGF-R4	NNK
ZMCWITLYKVLHACGSGSDYKDDDDK	>1.00E-05	2.31E-05	>1.00E-05	Crude	Selection	FGF-R4	NNK
ZMCWPILWKVLSACGSGSDYKDDDDK	>1.00E-05	>1.00E-05	2.49E-07	Crude	Selection	FGF-R4	NNK
ZMCWVKYWTVYKQCGSGSDYKDDDDK	>1.00E-05	>1.00E-05	2.07E-07	Crude	Selection	FGF-R4	NNK
ZMCWWFMPELVLKCGSGSDYKDDDDK	>1.00E-05	>1.00E-05	>1.00E-05	Crude	Selection	FGF-R4	NNK
ZMCYKLFLLWKQCGSGSDYKDDDDK	>1.00E-05	>1.00E-05	>1.00E-05	Crude	Selection	FGF-R4	NNK
ZMCYKLFLLWKQRCGSGSDYKDDDDK	>1.00E-05	>1.00E-05	>1.00E-05	Crude	Selection	FGF-R4	NNK
ZMCYKLFLLWKQWCGSGSDYKDDDDK	>1.00E-05	>1.00E-05	>1.00E-05	Crude	Selection	FGF-R4	NNK
ZMCYLYRFAVFCGSGSDYKDDDDK	>1.00E-05	>1.00E-05	>1.00E-05	Crude	Selection	FGF-R4	NNK
ZMMSHCWLFKVLLCGSGSDYKDDDDK	>1.00E-05	>1.00E-05	2.39E-07	Crude	Selection	FGF-R4	NNK
ZMMVKCWLAYIRTCGSGSDYKDDDDK	>1.00E-05	>1.00E-05	>1.00E-05	Crude	Selection	FGF-R4	NNK
ZMCNVLLYKVLRHCGSGSDYKDDDDK	>1.00E-05	>1.00E-05	1.11E-08	Crude	Selection	FGF-R4	T18_1
ZMCNVLLYKDLRHCGSGSDYKDDDDK	>1.00E-05	>1.00E-05	>1.00E-05	Crude	Selection	FGF-R4	T18_1
ZMCHLLYYKLFHSCGSGSDYKDDDDK	6.35E-08	>1.00E-05	1.14E-07	Crude	Selection	FGF-R4	T18_1
ZMCTTLYYYKLWRRCGSGSEEGSGSDYKDDDDK	6.99E-07	2.25E-06	7.18E-10	Crude	Selection	FGF-R4	T18_1
ZMCTYVYYYKLFHRCGSGSDYKDDDDK	>1.00E-05	>1.00E-05	2.88E-09	Crude	Selection	FGF-R4	T18_1
ZMCREIRYWQLYRAFCGSGSDYKDDDDK	>1.00E-05	>1.00E-05	>1.00E-05	Crude	Selection	FGF-R4	T18_1
ZMCRHLFYWKLWHQRCGSGSEEGSGSDYKDDDDK	>1.00E-05	>1.00E-05	1.79E-09	Crude	Selection	FGF-R4	T18_1
ZMCRWVKYYQLVHRKCGSGSDYKDDDDK	>1.00E-05	>1.00E-05	7.46E-10	Crude	Selection	FGF-R4	T18_1
ZMCREIRYWQLWQCGSGSDYKDDDDK	>1.00E-05	>1.00E-05	2.72E-08	Crude	Selection	FGF-R4	T18_1
ZMCYNIYWYKYVRRECGSGSDYKDDDDK	3.87E-07	>1.00E-05	5.71E-10	Crude	Selection	FGF-R4	T18_1
ZMCHIRYWQLVRKHCGSGSEEGSGSDYKDDDDK	>1.00E-05	>1.00E-05	1.01E-08	Crude	Selection	FGF-R4	T18_1
ZMCEVKYFLIWKRWCGSGSDYKDDDDK	6.50E-07	>1.00E-05	1.51E-08	Crude	Selection	FGF-R4	T18_1
ZMCYTVLWYKYQKKCGSGSEEGSGSDYKDDDDK	>1.00E-05	>1.00E-05	1.12E-09	Crude	Selection	FGF-R4	T18_1
ZMCPYAIYWYKWRKTCGSGSEEGSGSDYKDDDDK	>1.00E-05	>1.00E-05	1.08E-08	Crude	Selection	FGF-R4	T18_1
ZMCEHLFYYLLVKRQCGSGSDYKDDDDK	>1.00E-05	>1.00E-05	5.05E-10	Crude	Selection	FGF-R4	T18_1
ZMCEDLFYYIINRYGCGSGSDYKDDDDK	>1.00E-05	>1.00E-05	1.80E-08	Crude	Selection	FGF-R4	T18_1
ZMCSILWYKITKPLCGSGSDYKDDDDK	>1.00E-05	>1.00E-05	1.32E-08	Crude	Selection	FGF-R4	T18_1
ZMCEVIWYKIQWRKCGSGSDYKDDDDK	>1.00E-05	>1.00E-05	1.22E-08	Crude	Selection	FGF-R4	T18_1
ZMCKHLYYYLLWKKCGSGSEEGSGSDYKDDDDK	1.03E-06	9.28E-07	1.28E-09	Crude	Selection	FGF-R4	T18_1
ZMCEDLFYYLLFHHCGSGSDYKDDDDK	4.54E-06	>1.00E-05	8.10E-08	Crude	Selection	FGF-R4	T18_1
ZMCYKHILWYKIQKLCGSGSEEGSGSDYKDDDDK	3.31E-08	>1.00E-05	1.29E-09	Crude	Selection	FGF-R4	T18_1
ZMCYEIIWYKLQRVCGSGSDYKDDDDK	>1.00E-05	>1.00E-05	>1.00E-05	Crude	Selection	FGF-R4	T18_1
ZMCLWYYLQFHRRECGSGSDYKDDDDK	2.06E-08	>1.00E-05	4.63E-09	Crude	Selection	FGF-R4	T18_1
ZMCAVYYKLWLKHSRCGSGSEEGSGSDYKDDDDK	>1.00E-05	>1.00E-05	7.32E-09	Crude	Selection	FGF-R4	T18_1
ZMCKEYEILWYKLQYCGSGSDYKDDDDK	>1.00E-05	>1.00E-05	1.66E-08	Crude	Selection	FGF-R4	T18_1
ZMCDLYTVLWYKIQKECGSGSDYKDDDDK	2.10E-08	>1.00E-05	1.86E-08	Crude	Selection	FGF-R4	T18_1
ZMCAKYFLIWRAYQQCGSGSDYKDDDDK	>1.00E-05	>1.00E-05	9.53E-09	Crude	Selection	FGF-R4	T18_1
ZMCAKAFLLWKGKFCGSGSEEGSGSDYKDDDDK	>1.00E-05	>1.00E-05	>1.00E-05	Crude	Selection	FGF-R4	T18_1
ZMCPIHFFKIVYRHCGSGSEEGSGSDYKDDDDK	1.01E-08	>1.00E-05	1.05E-08	Crude	Selection	FGF-R4	T18_1
ZMCLWHYYKIFRRQKCGSGSDYKDDDDK	9.58E-08	>1.00E-05	7.70E-09	Crude	Selection	FGF-R4	T18_1
ZMCEWFYYHKSWYVYCGSGSDYKDDDDK	1.91E-07	>1.00E-05	>1.00E-05	Crude	Selection	FGF-R4	T18_1
ZMCHRYIYLDYWHCGSGSDYKDDDDK	>1.00E-05	>1.00E-05	4.44E-08	Crude	Selection	FGF-R4	T18_1
ZMCLRHLYEPYWHCGSGSDYKDDDDK	>1.00E-05	>1.00E-05	>1.00E-05	Crude	Selection	FGF-R4	T18_1
ZMCYKNISTGIFLYCGSGSDYKDDDDK	>1.00E-05	>1.00E-05	>1.00E-05	Crude	Selection	FGF-R4	T18_1
ZMCYKNIDNGVLVFCGSGSDYKDDDDK	>1.00E-05	>1.00E-05	1.36E-08	Crude	Selection	FGF-R4	T18_1
ZMCTYDFVLGHHYLICGSGSDYKDDDDK	>1.00E-05	>1.00E-05	>1.00E-05	Crude	Selection	FGF-R4	T18_1
ZMCTYEFLFEELYLHCGSGSDYKDDDDK	>1.00E-05	>1.00E-05	>1.00E-05	Crude	Selection	FGF-R4	T18_1
ZMCYYNFLNKVLVYCGSGSDYKDDDDK	>1.00E-05	>1.00E-05	8.49E-09	Crude	Selection	FGF-R4	T18_1
ZMCFLDHKTKVFVYCGSGSDYKDDDDK	>1.00E-05	>1.00E-05	1.30E-07	Crude	Selection	FGF-R4	T18_1
ZMCDSIRAQYARWWICGSGSDYKDDDDK	>1.00E-05	>1.00E-05	1.83E-07	Crude	Selection	FGF-R4	T18_1
ZMCESDAYRWKLWWVCGSGSDYKDDDDK	1.57E-06	>1.00E-05	1.04E-08	Crude	Selection	FGF-R4	T18_1
ZMCYDIVFDYITVYHCGSGSDYKDDDDK	>1.00E-05	>1.00E-05	2.12E-08	Crude	Selection	FGF-R4	T18_1
ZMCVWQYSHHYNWHCGSGSDYKDDDDK	>1.00E-05	>1.00E-05	>1.00E-05	Crude	Selection	FGF-R4	T18_1
ZMCVWKYRLDRNWYCGSGSDYKDDDDK	7.80E-08	>1.00E-05	2.04E-06	Crude	Selection	FGF-R4	T18_1
ZMCWWFKQHEFWFKVCGSGSDYKDDDDK	>1.00E-05	>1.00E-05	2.30E-09	Crude	Selection	FGF-R4	T18_1
ZMCWYDQHSFYWERLCGSGSDYKDDDDK	>1.00E-05	>1.00E-05	>1.00E-05	Crude	Selection	FGF-R4	T18_1
ZMCKYATLQWYKWFYCGSGSDYKDDDDK	1.14E-07	7.34E-06	6.32E-08	Crude	Selection	FGF-R4	T18_1
ZMCPYDYYDWHDWRDCGSGSDYKDDDDK	>1.00E-05	>1.00E-05	1.58E-06	Crude	Selection	FGF-R4	T18_1
ZMCWSKLIIKYYLSQCGSGSDYKDDDDK	>1.00E-05	>1.00E-05	1.63E-08	Crude	Selection	FGF-R4	T18_1
ZMCNLSIIHGYVAYICGSGSDYKDDDDK	>1.00E-05	>1.00E-05	6.69E-10	Crude	Selection	FGF-R4	T18_1
ZMCYLDDFYSYRYWACGSGSDYKDDDDK	5.43E-08	>1.00E-05	>1.00E-05	Crude	Selection	FGF-R4	T18_1
ZMCHIWYFNNQFHYVCGSGSDYKDDDDK	>1.00E-05	>1.00E-05	5.93E-09	Crude	Selection	FGF-R4	T18_1
ZMCKIWYVDHKFWWVCGSGSDYKDDDDK	>1.00E-05	>1.00E-05	1.79E-09	Crude	Selection	FGF-R4	T18_1
ZMCVWWSHPSIWICGSGSDYKDDDDK	6.45E-08	>1.00E-05	>1.00E-05	Crude	Selection	FGF-R4	T18_1
ZMCHRHLYYWRLFHSCGSGSEEGSGSDYKDDDDK	>1.00E-05	>1.00E-05	4.83E-09	Crude	Selection	FGF-R4	T18_1
ZMCEREVLYWLLVTCGSGSDYKDDDDK	4.82E-08	>1.00E-05	1.28E-07	Crude	Selection	FGF-R4	T18_1
ZMCKRHIRYWQLYVCGSGSEEGSGSDYKDDDDK	2.65E-07	8.38E-07	>1.00E-05	Crude	Selection	FGF-R4	T18_1
ZMCKREIKYWLVFCGSGSDYKDDDDK	>1.00E-05	>1.00E-05	8.09E-09	Crude	Selection	FGF-R4	T18_1
ZMCAKEHLFYYLLWCGSGSDYKDDDDK	>1.00E-05	>1.00E-05	4.13E-08	Crude	Selection	FGF-R4	T18_1
ZMCKRHLIYYKIARCGSGSDYKDDDDK	>1.00E-05	1.54E-06	1.35E-09	Crude	Selection	FGF-R4	T18_1
ZMCIRYLFYYLISHCGSGSDYKDDDDK	>1.00E-05	>1.00E-05	2.17E-09	Crude	Selection	FGF-R4	T18_1
ZMCPYYVQYWRVFCGSGSDYKDDDDK	>1.00E-05	>1.00E-05	9.09E-09	Crude	Selection	FGF-R4	T18_1
ZMCTGRHLYYWKLFHCGSGSEEGSGSDYKDDDDK	>1.00E-05	>1.00E-05	9.90E-09	Crude	Selection	FGF-R4	T18_1

ZMCDAYYVRYWQVLHCGSGSDYKDDDDK	>1.00E-05	>1.00E-05	3.59E-08	Crude	Selection	FGF-R4	T18_1
ZMCKYSKDLWLYRLWCGSGSDYKDDDDK	>1.00E-05	>1.00E-05	2.34E-09	Crude	Selection	FGF-R4	T18_1
ZMCYKYSKDLWLYRLWCGSGSDYKDDDDK	5.02E-07	>1.00E-05	2.16E-08	Crude	Selection	FGF-R4	T18_1
ZMCIKDLHLFYYLLCGSGSDYKDDDDK	3.00E-08	>1.00E-05	1.44E-08	Crude	Selection	FGF-R4	T18_1
ZMCDKFVKWYIVFRHCGSGSEEGSGSDYKDDDDK	>1.00E-05	>1.00E-05	9.11E-10	Crude	Selection	FGF-R4	T18_1
ZMCVKYIKYWSVFHCGSGSDYKDDDDK	>1.00E-05	>1.00E-05	1.66E-08	Crude	Selection	FGF-R4	T18_1
ZMCDWYVTIYKVLKKCGSGSDYKDDDDK	>1.00E-05	>1.00E-05	7.70E-09	Crude	Selection	FGF-R4	T18_1
ZMCYEDEIWLYKVLYCGSGSDYKDDDDK	>1.00E-05	>1.00E-05	9.41E-09	Crude	Selection	FGF-R4	T18_1
ZMCYHRHIFQNHWYKCGSGSEEGSGSDYKDDDDK	>1.00E-05	>1.00E-05	>1.00E-05	Crude	Selection	FGF-R4	T18_1
ZMCYHRPPNSKIWRKCGSGSDYKDDDDK	>1.00E-05	>1.00E-05	2.14E-07	Crude	Selection	FGF-R4	T18_1
ZMCEQRSRWSNYWYPCGSGSDYKDDDDK	>1.00E-05	>1.00E-05	>1.00E-05	Crude	Selection	FGF-R4	T18_1
ZMCYTRFWTEEWQPCGSGSDYKDDDDK	>1.00E-05	>1.00E-05	>1.00E-05	Crude	Selection	FGF-R4	T18_1
ZMCYTRHPYEALWHQCGSGSDYKDDDDK	>1.00E-05	>1.00E-05	>1.00E-05	Crude	Selection	FGF-R4	T18_1
ZMCTHRHPYETLWHQCGSGSDYKDDDDK	>1.00E-05	>1.00E-05	>1.00E-05	Crude	Selection	FGF-R4	T18_1
ZMCYTRFYNWEKWQLCGSGSDYKDDDDK	1.65E-08	>1.00E-05	>1.00E-05	Crude	Selection	FGF-R4	T18_1
ZMCITRKNKWEEWRPCGSGSDYKDDDDK	>1.00E-05	>1.00E-05	>1.00E-05	Crude	Selection	FGF-R4	T18_1
ZMCEHRHPYGHWEQCGSGSDYKDDDDK	>1.00E-05	>1.00E-05	>1.00E-05	Crude	Selection	FGF-R4	T18_1
ZMCFHIKANYGFVRVCGSGSDYKDDDDK	>1.00E-05	>1.00E-05	>1.00E-05	Crude	Selection	FGF-R4	T18_2
ZMCLHIKANYGFVRVCGSGSDYKDDDDK	>1.00E-05	>1.00E-05	>1.00E-05	Crude	Selection	FGF-R4	T18_2
ZMCTYTYVFNQTYVHCGSGSDYKDDDDK	>1.00E-05	>1.00E-05	>1.00E-05	Crude	Selection	FGF-R4	T18_2
ZMCVYEYVFDRLTVTCGSGSDYKDDDDK	>1.00E-05	>1.00E-05	>1.00E-05	Crude	Selection	FGF-R4	T18_2
ZMCEYTYLFGYHYIKCGSGSDYKDDDDK	>1.00E-05	>1.00E-05	>1.00E-05	Crude	Selection	FGF-R4	T18_2
ZMCAYEYVFDRLTVTCGSGSDYKDDDDK	>1.00E-05	>1.00E-05	2.82E-08	Crude	Selection	FGF-R4	T18_2
ZMCHNKYLYYYLLYSCGSGSDYKDDDDK	>1.00E-05	>1.00E-05	3.56E-08	Crude	Selection	FGF-R4	T18_2
ZMCYKRDLYYYLVFKCGSGSDYKDDDDK	>1.00E-05	>1.00E-05	8.41E-09	Crude	Selection	FGF-R4	T18_2
ZMCDKRHLYYYLLVCGSGSDYKDDDDK	>1.00E-05	>1.00E-05	3.18E-08	Crude	Selection	FGF-R4	T18_2
ZMCDKYWVKYWIAWRCGSGSDYKDDDDK	>1.00E-05	>1.00E-05	1.09E-09	Crude	Selection	FGF-R4	T18_2
ZMCEREFLYYYLYYKCGSGSDYKDDDDK	>1.00E-05	>1.00E-05	>1.00E-05	Crude	Selection	FGF-R4	T18_2
ZMCWEEFTKFGWWTICGSGSDYKDDDDK	>1.00E-05	>1.00E-05	1.37E-09	Crude	Selection	FGF-R4	T18_2
ZMCFKQHLFYYLLHCGSGSDYKDDDDK	>1.00E-05	>1.00E-05	2.16E-08	Crude	Selection	FGF-R4	T18_2
ZMCYNKFITLYKVLYCGSGSDYKDDDDK	5.05E-07	7.89E-08	1.28E-08	Crude	Selection	FGF-R4	T18_2
ZMCHDKFITLYKVLYCGSGSDYKDDDDK	>1.00E-05	>1.00E-05	4.82E-08	Crude	Selection	FGF-R4	T18_2
ZMCYYEVLWYQQQRHCGSGSDYKDDDDK	>1.00E-05	>1.00E-05	1.18E-08	Crude	Selection	FGF-R4	T18_2
ZMCRDFDIIWYKLQFCGSGSDYKDDDDK	5.36E-06	3.40E-07	2.02E-08	Crude	Selection	FGF-R4	T18_2
ZMCKYSVYWYKWQRKCGSGSDYKDDDDK	>1.00E-05	>1.00E-05	>1.00E-05	Crude	Selection	FGF-R4	T18_2
ZMCEFEIRWYQYQRKCGSGSDYKDDDDK	>1.00E-05	>1.00E-05	1.62E-08	Crude	Selection	FGF-R4	T18_2
ZMCWEEIIWYKLQHKCGSGSDYKDDDDK	>1.00E-05	>1.00E-05	6.64E-09	Crude	Selection	FGF-R4	T18_2
ZMCDNRRWVRYYQIFCGSGSDYKDDDDK	>1.00E-05	>1.00E-05	1.09E-08	Crude	Selection	FGF-R4	T18_2
ZMCENKKVLFYYLLWCGSGSDYKDDDDK	>1.00E-05	>1.00E-05	>1.00E-05	Crude	Selection	FGF-R4	T18_2
ZMCDLKRELFYYLLVCGSGSDYKDDDDK	>1.00E-05	>1.00E-05	2.71E-08	Crude	Selection	FGF-R4	T18_2
ZMCDHKRHLYYYILWCGSGSDYKDDDDK	>1.00E-05	>1.00E-05	5.29E-09	Crude	Selection	FGF-R4	T18_2
ZMCKFDLDLLFYKIWCGSGSDYKDDDDK	>1.00E-05	>1.00E-05	8.60E-09	Crude	Selection	FGF-R4	T18_2
ZMCHFKRDYYWYLWQCGSGSDYKDDDDK	>1.00E-05	>1.00E-05	7.33E-08	Crude	Selection	FGF-R4	T18_2
ZMCKYHRDLLYYFLHCGSGSDYKDDDDK	>1.00E-05	>1.00E-05	2.29E-08	Crude	Selection	FGF-R4	T18_2
ZMCVHKKWIRYWQIFCGSGSEEGSGSDYKDDDDK	>1.00E-05	>1.00E-05	5.80E-09	Crude	Selection	FGF-R4	T18_2
ZMCFWHKRSDFWLQFCGSGSDYKDDDDK	>1.00E-05	>1.00E-05	3.67E-07	Crude	Selection	FGF-R4	T18_2
ZMCWWFRNHKFWQQICGSGSDYKDDDDK	>1.00E-05	>1.00E-05	8.83E-09	Crude	Selection	FGF-R4	T18_2
ZMCFWKKLHVFWVKDCGSGSDYKDDDDK	2.27E-08	>1.00E-05	>1.00E-05	Crude	Selection	FGF-R4	T18_2
ZMCWYVQHSFYWERLCGSGSDYKDDDDK	>1.00E-05	>1.00E-05	3.29E-08	Crude	Selection	FGF-R4	T18_2
ZMCWWIQHPLFWSKICGSGSDYKDDDDK	>1.00E-05	>1.00E-05	2.15E-08	Crude	Selection	FGF-R4	T18_2
ZMCFRHKRSDFWLQFCGSGSDYKDDDDK	>1.00E-05	>1.00E-05	>1.00E-05	Crude	Selection	FGF-R4	T18_2
ZMCEHRHPYSNFWILCGSGSDYKDDDDK	>1.00E-05	>1.00E-05	>1.00E-05	Crude	Selection	FGF-R4	T18_2
ZMCEHRHPFTQYWLRCGSGSDYKDDDDK	>1.00E-05	>1.00E-05	>1.00E-05	Crude	Selection	FGF-R4	T18_2
ZMCEHRHPYEAFWHECGSGSDYKDDDDK	>1.00E-05	>1.00E-05	>1.00E-05	Crude	Selection	FGF-R4	T18_2
ZMCVHRHRFEPEWHVCGSGSDYKDDDDK	>1.00E-05	>1.00E-05	>1.00E-05	Crude	Selection	FGF-R4	T18_2
ZMCAYRHIYEPYWHSCGSGSDYKDDDDK	>1.00E-05	>1.00E-05	>1.00E-05	Crude	Selection	FGF-R4	T18_2
ZMCEHKHPFERVWRSCGSGSDYKDDDDK	>1.00E-05	>1.00E-05	>1.00E-05	Crude	Selection	FGF-R4	T18_2
ZMCEYRHKFEERWHVCGSGSDYKDDDDK	>1.00E-05	>1.00E-05	>1.00E-05	Crude	Selection	FGF-R4	T18_2
ZMCEHRHPYSNFWIPCGSGSDYKDDDDK	>1.00E-05	>1.00E-05	>1.00E-05	Crude	Selection	FGF-R4	T18_2
ZMCYTRHPFQKRWHYCGSGSEEGSGSDYKDDDDK	>1.00E-05	>1.00E-05	>1.00E-05	Crude	Selection	FGF-R4	T18_2
ZMCYTKAYWHEPWRPCGSGSDYKDDDDK	>1.00E-05	3.15E-06	>1.00E-05	Crude	Selection	FGF-R4	T18_2
ZMCYTRKPFEFFWRLCGSGSDYKDDDDK	>1.00E-05	>1.00E-05	1.68E-07	Crude	Selection	FGF-R4	T18_2
ZMCYTRHKFDHRWRACGSGSEEGSGSDYKDDDDK	>1.00E-05	>1.00E-05	>1.00E-05	Crude	Selection	FGF-R4	T18_2
ZMCYTRDRYELHWRLCGSGSDYKDDDDK	>1.00E-05	>1.00E-05	>1.00E-05	Crude	Selection	FGF-R4	T18_2
ZMCYTRHKFDQYWQPCGSGSDYKDDDDK	>1.00E-05	>1.00E-05	>1.00E-05	Crude	Selection	FGF-R4	T18_2
ZMCITRHRFEQFWHLCGSGSDYKDDDDK	>1.00E-05	>1.00E-05	>1.00E-05	Crude	Selection	FGF-R4	T18_2
ZMCYTKATVFSQWVLCGSGSDYKDDDDK	>1.00E-05	>1.00E-05	>1.00E-05	Crude	Selection	FGF-R4	T18_2
ZMCYTRRFKWQDWHACGSGSDYKDDDDK	>1.00E-05	>1.00E-05	>1.00E-05	Crude	Selection	FGF-R4	T18_2
ZMCYTKYFYFEPWRICGSGSDYKDDDDK	>1.00E-05	>1.00E-05	>1.00E-05	Crude	Selection	FGF-R4	T18_2
ZMCTTRKNKWEEWRPCGSGSDYKDDDDK	>1.00E-05	>1.00E-05	>1.00E-05	Crude	Selection	FGF-R4	T18_2
ZMCYTKSFYWPIWRSCGSGSDYKDDDDK	>1.00E-05	>1.00E-05	>1.00E-05	Crude	Selection	FGF-R4	T18_2
ZMCYTRLNKHAKWQKCGSGSDYKDDDDK	>1.00E-05	>1.00E-05	1.49E-07	Crude	Selection	FGF-R4	T18_2
ZMCTDLYYYLIWQRYCGSGSDYKDDDDK	>1.00E-05	>1.00E-05	9.77E-09	Crude	Selection	FGF-R4	T18_2
ZMCNDLFYYLLWRKACGSGSDYKDDDDK	>1.00E-05	>1.00E-05	1.45E-09	Crude	Selection	FGF-R4	T18_2
ZMCKHLFYYKLIRKACGSGSDYKDDDDK	>1.00E-05	>1.00E-05	6.54E-09	Crude	Selection	FGF-R4	T18_2
ZMCVDLFYYKLFRKLCGSGSDYKDDDDK	>1.00E-05	>1.00E-05	9.23E-09	Crude	Selection	FGF-R4	T18_2
ZMCPYTKYWIVWRHSCGSGSDYKDDDDK	>1.00E-05	>1.00E-05	3.24E-09	Crude	Selection	FGF-R4	T18_2
ZMCEHLYYYLLSHRRCGSGSDYKDDDDK	1.72E-07	>1.00E-05	7.09E-09	Crude	Selection	FGF-R4	T18_2
ZMCRELFYYRFWQKTCGSGSDYKDDDDK	>1.00E-05	>1.00E-05	1.44E-08	Crude	Selection	FGF-R4	T18_2
ZMCRHLAYWKLWHRKCGSGSDYKDDDDK	>1.00E-05	>1.00E-05	7.81E-09	Crude	Selection	FGF-R4	T18_2
ZMCDIVIYKILRSLCGSGSDYKDDDDK	>1.00E-05	2.54E-09	4.13E-09	Crude	Selection	FGF-R4	T18_2
ZMCDLFYYLQHHKKVCGSGSDYKDDDDK	>1.00E-05	>1.00E-05	7.57E-09	Crude	Selection	FGF-R4	T18_2
ZMCYVQYYRVWRSICGSGSDYKDDDDK	>1.00E-05	>1.00E-05	3.89E-09	Crude	Selection	FGF-R4	T18_2
ZMCEVIWYKIQYRLCGSGSDYKDDDDK	>1.00E-05	>1.00E-05	2.14E-08	Crude	Selection	FGF-R4	T18_2
ZMCDLFYYLFIKRRKCGSGSEEGSGSDYKDDDDK	>1.00E-05	>1.00E-05	9.60E-09	Crude	Selection	FGF-R4	T18_2
ZMCHLWYYLVHHRRDCGSGSDYKDDDDK	>1.00E-05	>1.00E-05	6.08E-09	Crude	Selection	FGF-R4	T18_2
ZMCVIVIYKILRSLCGSGSDYKDDDDK	>1.00E-05	>1.00E-05	>1.00E-05	Crude	Selection	FGF-R4	T18_2
ZMCYNVHWYRWIRRRCGSGSDYKDDDDK	>1.00E-05	>1.00E-05	>1.00E-05	Crude	Selection	FGF-R4	T18_2
ZMCYEVLWYKLQRKHCGSGSDYKDDDDK	>1.00E-05	>1.00E-05	3.38E-09	Crude	Selection	FGF-R4	T18_2
ZMCYTELWYKYIRRHCGSGSDYKDDDDK	1.12E-06	>1.00E-05	6.00E-09	Crude	Selection	FGF-R4	T18_2
ZMCYDVLWYRYRYKQCGSGSDYKDDDDK	>1.00E-05	>1.00E-05	2.16E-08	Crude	Selection	FGF-R4	T18_2
ZMCKIIWLYKAARRFCGSGSDYKDDDDK	>1.00E-05	>1.00E-05	7.32E-09	Crude	Selection	FGF-R4	T18_2
ZMCYEVLWYQYWRKVCGSGSDYKDDDDK	>1.00E-05	>1.00E-05	2.17E-08	Crude	Selection	FGF-R4	T18_2
ZMCFNVHWYRWIRRRCGSGSDYKDDDDK	>1.00E-05	>1.00E-05	3.12E-09	Crude	Selection	FGF-R4	T18_2
ZMCNKHLYYYLLFKHCGSGSDYKDDDDK	>1.00E-05	>1.00E-05	8.86E-09	Crude	Selection	FGF-R4	T18_2
ZMCPRYVLYYKLFHHCGSGSDYKDDDDK	>1.00E-05	>1.00E-05	8.28E-09	Crude	Selection	FGF-R4	T18_2
ZMCKRHITYWKLWHFCGSGSEEGSGSDYKDDDDK	>1.00E-05	>1.00E-05	9.69E-09	Crude	Selection	FGF-R4	T18_2
ZMCHDYIVFYKVLRHCGSGSDYKDDDDK	>1.00E-05	>1.00E-05	1.73E-08	Crude	Selection	FGF-R4	T18_2
ZMCRRELYYYLLYSKCGSGSDYKDDDDK	>1.00E-05	>1.00E-05	5.23E-09	Crude	Selection	FGF-R4	T18_2
ZMCDRLILYYRIVHKCGSGSDYKDDDDK	>1.00E-05	>1.00E-05	2.18E-08	Crude	Selection	FGF-R4	T18_2
ZMCPYEIKYWIVWQQCGSGSDYKDDDDK	>1.00E-05	>1.00E-05	1.42E-08	Crude	Selection	FGF-R4	T18_2

ZMCNKHLYYYLLFKQCGSGSDYKDDDDK	>1.00E-05	>1.00E-05	1.00E-09	Crude	Selection	FGF-R4	T18_2
ZMCIYRLSDFWVVCGSGSDYKDDDDK	5.34E-08	>1.00E-05	7.01E-08	Purified	Selection	FGF-R4	NNK
ZMCIYRLSDFWVECGSGSDYKDDDDK	>1.00E-05	>1.00E-05	>1.00E-05	Purified	Selection	FGF-R4	NNK
ZMCLVSIYKIMRTCGSGSDYKDDDDK	>1.00E-05	>1.00E-05	>1.00E-05	Purified	Selection	FGF-R4	NNK
ZMCHVILWRSWRYCGSGSDYKDDDDK	>1.00E-05	>1.00E-05	5.13E-08	Purified	Selection	FGF-R4	NNK
ZMCHLAYYLAFRSCGSGSDYKDDDDK	>1.00E-05	>1.00E-05	8.61E-08	Purified	Selection	FGF-R4	NNK
ZMCVTWYKVTKLRCGSGSDYKDDDDK	>1.00E-05	>1.00E-05	>1.00E-05	Purified	Selection	FGF-R4	NNK
ZMCFKHPWFFAWLCGSGSDYKDDDDK	>1.00E-05	>1.00E-05	2.33E-07	Purified	Selection	FGF-R4	NNK
ZMCIKMFLAHKRFCGSGSDYKDDDDK	>1.00E-05	>1.00E-05	3.58E-07	Purified	Selection	FGF-R4	NNK
ZMCPERLFYWLLVCGSGSDYKDDDDK	>1.00E-05	>1.00E-05	4.43E-07	Purified	Selection	FGF-R4	NNK
ZMCEPWITIYKVLKKCGSGSDYKDDDDK	>1.00E-05	>1.00E-05	4.78E-07	Purified	Selection	FGF-R4	NNK
ZMCTTLYYYKLWRRCGSGSEEGSGSDYKDDDDK	>1.00E-05	>1.00E-05	>1.00E-05	Purified	Selection	FGF-R4	T18_1
ZMCKHLYYYLLWKKCGSGSEEGSGSDYKDDDDK	>1.00E-05	5.19E-07	1.60E-09	Purified	Selection	FGF-R4	T18_1
ZMCNVLLYKVLRHCGSGSDYKDDDDK	>1.00E-05	1.15E-07	5.43E-07	Purified	Selection	FGF-R4	T18_1
ZMCESDAYRWKLWWVCGSGSDYKDDDDK	>1.00E-05	>1.00E-05	1.54E-08	Purified	Selection	FGF-R4	T18_1
ZMCYTRFYNWEKWQLCGSGSDYKDDDDK	>1.00E-05	>1.00E-05	>1.00E-05	Purified	Selection	FGF-R4	T18_1
ZMCYHRPPNSKIWRKCGSGSDYKDDDDK	>1.00E-05	>1.00E-05	5.24E-07	Purified	Selection	FGF-R4	T18_1
ZMCEREVLYWLLVTCGSGSDYKDDDDK	>1.00E-05	>1.00E-05	3.78E-07	Purified	Selection	FGF-R4	T18_1
ZMCYLDDFYSYRYWACGSGSDYKDDDDK	1.50E-08	3.70E-08	>1.00E-05	Purified	Selection	FGF-R4	T18_1
ZMCNVLLYKDLRHCGSGSDYKDDDDK	>1.00E-05	>1.00E-05	>1.00E-05	Purified	Selection	FGF-R4	T18_1
ZMCNDLFYYLLWRKACGSGSDYKDDDDK	>1.00E-05	>1.00E-05	6.02E-07	Purified	Selection	FGF-R4	T18_2
ZMCYNKFITLYKVLYCGSGSDYKDDDDK	>1.00E-05	>1.00E-05	7.90E-07	Purified	Selection	FGF-R4	T18_2
ZMCENKKVLFYYLLWCGSGSDYKDDDDK	>1.00E-05	>1.00E-05	3.21E-07	Purified	Selection	FGF-R4	T18_2
ZMCDKYWVKYWIAWRCGSGSDYKDDDDK	0.00E+00	9.10E-08	4.11E-07	Purified	Selection	FGF-R4	T18_2
ZMCFWKKLHVFWVKDCGSGSDYKDDDDK	1.70E-08	3.70E-09	1.00E-10	Purified	Selection	FGF-R4	T18_2
ZMCEHLYYYLLSHRRCGSGSDYKDDDDK	>1.00E-05	>1.00E-05	3.20E-09	Purified	Selection	FGF-R4	T18_2
ZMCYTKAYWHEPWRPCGSGSDYKDDDDK	>1.00E-05	>1.00E-05	>1.00E-05	Purified	Selection	FGF-R4	T18_2
ZMCNKHLYYYLLFKQCGSGSDYKDDDDK	2.20E-06	2.71E-06	2.60E-09	Purified	Selection	FGF-R4	T18_2
