# Supplementary material for: Identification and engineering of potent cyclic peptides with selective or promiscuous binding through biochemical profiling and bioinformatic data analysis
Source: RSC Chem Biol. 2023 Nov 14;5(1):12–8. doi: 10.1039/d3cb00168g (PMC10763615; doi:10.1039/d3cb00168g)
Supplement: CB-005-D3CB00168G-s005 [file CB-005-D3CB00168G-s005.pdf]

Electronic Supplementary Material (ESI) for RSC Chemical Biology.
This journal is © The Royal Society of Chemistry 2023

sequence	R0	R1	R2	R3	R4	R5
CIFDIHMPFIPC	306	265	570	7272	68087	189541
CVFDSFLPFIIC	110	83	218	2662	29885	52987
CIFDTFLPFIFC	55	45	105	1572	16912	29817
CTFDLFLPFIFC	63	58	283	5696	21253	17922
CVYDIFVPFIIC	40	39	147	2480	17473	17161
CLYDSFLPFIFC	48	30	149	3189	17133	14658
CIFDNWLPFIYC	46	29	203	3063	20159	12072
CVFDLFLPFIFC	27	31	100	1747	10834	11879
CVYDLFLPFIYC	35	22	136	2344	14828	9697
CTFDTFLPFIFC	26	23	126	2076	12579	9578
CVFDSFLPFILC	23	24	83	1145	7916	9373
CLFDIFLPFIFC	28	22	127	3167	7519	8178
CLFDSFVPFIFC	32	24	160	3877	11592	8114
CYFDPHFIFIPC	18	12	154	2198	8903	7874
CVFDSFLPFIFC	15	16	13	81	2736	7822
CIFDLFVPFIFC	13	14	82	1480	7464	7267
CIFDIFMPFILC	23	10	92	1415	7618	6570
CCFDSFLPFIFC	18	26	135	2185	10361	6245
CLFDTFLPFIFC	19	9	74	2484	6956	5986
CLFDIFLPFIIC	8	16	81	1157	6078	5648
CIFDLFCPFIVC	27	16	85	1887	8101	5208
CLFDLFCPFIFC	21	14	148	3966	6748	4372
CLFDTFVPFIFC	36	19	131	2886	8584	4227
CIFDIFMPFIPC	7	6	14	126	1720	3852
CIFDNWLPFIFC	10	5	6	110	2560	3301
CIFDTFLPFIIC	10	4	2	17	602	3110
CIFDTFLPFILC	5	5	18	110	1297	2960
CVFDSFLPFIPC	11	1	9	36	1353	2397
CLFDSFLPFILC	12	9	135	1916	5081	2360
CVFDIHMPFIPC	6	2	7	128	988	2271
CTFDIFLPFILC	9	5	72	1285	4659	2211
CVYDIFVPFIFC	2	9	4	49	1488	2196
CLFDSFLPFIFC	7	6	7	125	1297	2039
CVFDVFCPFIFC	7	13	84	1620	6316	2023
CIFDTFLPFIYC	3	2	3	33	796	2010
CVYDLFLPFIFC	7	0	6	83	1866	1954
CIFDIFMPFIFC	4	1	2	36	875	1953
CIFDIHMPFILC	8	1	7	41	1640	1927
CLFDIFCPFILC	4	6	106	1755	4931	1922
CIWDSFVPFILC	9	11	75	1235	5678	1919
CFYDLFLPFIFC	17	10	60	1200	5301	1859
CCFDIFLPFIFC	5	6	54	717	2962	1832
CTFDLFLPFIYC	3	9	7	129	1192	1827
CIFDSFFPFIPC	15	5	186	1819	4114	1702
CVFDSFLPFVLC	10	17	129	1623	5245	1677
CTFDIFLPFIFC	4	2	7	58	858	1459
CTFDVFLPFIFC	10	3	86	1634	4236	1439
CIWDSFVPFIFC	5	2	12	179	2011	1429
CIFDTHMPFIPC	2	1	1	64	474	1413
CLFDLFVPFVFC	15	9	76	1495	3911	1386
CIFDIHMPFTPC	4	2	8	160	724	1336
CIFDLFSPFIFC	4	2	32	401	1806	1229
CLFDLHLPFIFC	12	3	77	932	3547	1228
CIFDTFLPFIPC	1	4	4	9	507	1210
CLFDSFVPFVFC	14	9	109	1619	5010	1192
CVFDLFLPFIYC	3	2	2	33	615	1180
CVFDIFIPFVLC	12	7	84	1078	3657	1140
CTFDLFLPFIIC	3	5	6	30	678	1097
CIFDSFFPFIFC	6	7	65	785	2734	996
CIFDNWLPFIIC	0	1	1	3	209	945
CIFDLFAPFIYC	2	3	57	481	2483	902
CTFDTFLPFIIC	2	2	0	21	457	878
CIFDNWLPFIPC	3	1	2	16	540	842
CTFDTHFPFITC	7	7	77	1194	2962	832
CVFDLFLPFTFC	4	5	58	745	2684	817
CLFDIFCPFIFC	0	1	2	82	654	816
CLFDSFLPFIIC	2	2	3	71	554	816
CLYDSFLPFIIC	0	1	0	10	531	810
CVFDLFLPFIIC	1	3	2	17	346	804
CIFDLFCPFIFC	1	1	4	14	380	712
CVFDLFLPFILC	1	1	9	68	609	681
CIFDIFMPFIIC	0	0	2	9	265	676
CTFDLFLPFIPC	2	0	3	14	571	657
CCFDSFLPFIIC	2	2	3	16	409	656
CVFDSLLPFIFC	6	6	50	782	2044	615
CTFDIHMPFIPC	1	2	4	43	350	607
CTFDLFLPFILC	1	1	12	185	1011	605
CIFDNWLPFILC	2	0	2	3	273	599
CLFDIFLPFIPC	1	1	2	9	384	599
CVFDLFLPFIPC	2	0	2	12	283	599
CTFDTFLPFIYC	1	1	2	32	552	590
CLFDVFCDFIFC	14	6	113	2680	2477	587
CLFDTFLPFIIC	3	2	6	44	336	571
CIFDIHMPLIPC	2	0	5	59	237	566
CIFDLFVPFIYC	2	2	5	32	480	564
CLFDLFFPFIPC	4	2	265	1892	1693	564
CIFDLFVPFIIC	1	0	2	10	221	556
CIFDLFVPFILC	4	1	6	56	453	551
CLFDIFLPFIYC	2	0	5	62	393	550
CIFDIHMPFIFC	0	0	3	9	439	549
CIFDSFLPFVFC	3	2	5	54	412	537
CIFDLWLPFTAC	1	4	11	441	1403	534
CVFDIFIPFVFC	1	1	4	65	509	523
CVYDIFVPFIPC	3	0	2	14	450	519
CIFDLFCPFIIC	0	0	2	3	225	517
CIFDLFLPFIFC	1	1	1	20	253	508
CLFDPFLSFIPC	8	4	148	3132	2069	505
CVFDSFLPFIVC	3	2	1	23	341	505
CVYDIFVPFILC	2	1	2	8	349	482
CIFDTHFPFILC	2	3	1	11	174	476
CLFDSFVPFIIC	0	2	0	22	335	468
CCFDSFLPFVIC	1	9	52	826	1905	467
CVFDTFLPFIFC	0	1	0	25	241	465

CLFDIFLPFILC	3	0	8	130	511	451
CIFDSFLPFIFC	0	1	1	28	214	449
CTFDTFLPFILC	1	1	3	88	699	438
CVYDLFLPFIPC	0	0	0	7	445	434
CVFDSFLPFIMC	4	0	6	61	444	426
CIFDLFCPFILC	1	0	1	35	381	425
CTFDTHLPFIYC	4	7	70	1057	2554	415
CLFDTFVPFIIC	1	0	1	28	314	407
CLYDSFLPFILC	0	0	3	68	666	405
CLFDLFYPFIIC	0	2	49	657	1511	403
CLYDSFLPFIPC	0	1	1	8	429	390
CTFDIFLPFIIC	0	0	0	19	275	387
CVFDSWLPFVFC	1	4	2	52	438	374
CIFDIHMPFITC	0	1	2	21	215	364
CILDIHMPFIPC	0	1	0	30	182	360
CVFDSWLPFVYC	10	6	58	742	1923	354
CIFDLFAPFIFC	3	0	2	30	358	353
CIFDNWLPFICC	0	2	1	42	457	344
CIFDIHTPFIPC	0	1	2	19	165	339
CLFDLFCPFIIC	0	0	3	34	240	317
CVFDSFLPFIYC	0	1	0	4	171	311
CLFDSFLPFIPC	0	0	2	50	547	299
CTFDTFLPFIPC	0	0	1	8	339	297
CLFDIFCPFIIC	0	0	4	19	218	287
CIFDIHMPFIIC	1	0	1	1	123	285
CLYDSFLPFIYC	3	1	1	30	516	284
CIFDIFLPFIFC	0	0	1	2	125	276
CIYDIHMPFIPC	0	0	1	12	101	276
CTFDIFLPFIPC	1	0	1	29	418	275
CLFDSFLPFIVC	2	3	69	821	1456	273
CLFDTFLPFIYC	2	0	1	40	286	272
CIFDTFLPFICC	2	0	1	9	184	257
CIFDVHMPFIPC	1	1	1	110	166	257
CIFDIHMPFVPC	0	0	3	22	124	256
CTFDTHFPFIIC	0	1	1	10	163	248
CLFDSFVPFILC	2	2	7	71	434	246
CIFDSFLPFILC	3	1	0	8	111	245
CLFDIHMPFIPC	0	0	0	7	91	245
CVFDLHCPFIFC	4	1	32	459	1356	243
CLFDSLLPFIFC	7	6	45	510	1106	242
CIFDIHMPSIPC	1	1	1	15	135	241
CVFDSFLPFVIC	0	1	2	9	160	236
CCFDSFLPFILC	1	4	6	70	522	236
CLFDLFCPFIYC	1	5	2	68	306	235
CIFDLFVPFIPC	0	0	0	4	210	234
CCFDSFLPFIYC	1	1	4	34	462	229
CIFDLWLPFTPC	0	0	2	19	138	225
CLFDLFVPFIFC	0	3	1	12	218	223
CLFDTFLPFILC	1	2	4	74	343	218
CLFDSFVPFIYC	1	1	1	24	367	214
CIFDSFFPFILC	0	0	3	72	360	211
CIFDIHMPFIHC	0	0	2	12	190	210

CIFDIHMPFIPS	0	0	1	9	82	206
CLFDSFVPFIPC	1	0	0	14	259	204
CIFDTFLPFIVC	1	0	4	12	103	201
CLFDIFCPFIPC	0	0	1	42	424	201
CIFDIHKPFIPC	1	0	2	14	87	200
CLFDLFFPFIIC	4	1	32	275	685	197
CCFDSFLPFIPC	0	1	0	5	274	195
CIFDSFVPFIFC	1	0	0	8	113	194
CVFDIFMPFILC	1	0	1	26	168	194
CVFDVFCPFIIC	1	1	0	13	219	194
CVFDSLLPFIIC	0	0	2	17	202	193
CLFDLFCPFILC	2	1	5	139	351	192
CIFGIHMPFIPC	0	1	3	19	104	191
CYFDIFLPFIFC	5	1	58	620	945	191
CLFDTFLPFIPC	0	0	0	9	195	190
CLFDTFVPFILC	1	1	7	92	456	188
CVFDSWLPFIFC	0	2	0	1	78	187
CIFELFFPFIPC	2	1	70	339	653	181
CLFDLYCDFIFC	5	1	45	590	1053	180
CISDIHMPFIPC	1	0	0	16	109	175
CIFDIRMPFIPC	0	1	0	16	92	173
CIFDIHVPFIPC	0	1	0	10	72	168
CIFDLFLPFVFC	0	1	4	54	226	168
CVFDLFVPFIFC	0	0	2	27	163	164
CIFDLFFPFVPC	2	0	37	206	450	163
CIFELNLPFIPC	2	0	43	387	737	162
CVFDSFLPLIIC	0	1	1	11	112	161
CVYDLFLPFIIC	0	0	2	1	128	161
CIFDIHIPFIPC	0	0	0	10	58	157
CFFDIHMPFIPC	0	0	1	11	80	157
CVYDIFVPFIMC	1	0	1	37	293	154
CLFDTFVPFIYC	0	0	3	23	344	154
CTFDLFLPFICC	1	0	3	32	242	153
CIFDTFVPFIFC	0	0	2	16	93	149
CIFDLFCPFIPC	0	0	0	4	151	148
CVFDIFIPFVIC	1	0	1	20	152	148
CVFDVFCPFILC	0	0	5	71	354	148
CVYDIFVPFIYC	0	0	0	5	121	147
CNFDIHMPFIPC	1	0	0	4	57	143
CCFDIFLPFIYC	1	0	2	9	138	140
CIFDIHMPFIPY	0	0	2	9	68	138
CIFDIHMPFNPC	0	0	0	12	61	137
CFFDLHLPFVFC	1	1	56	704	591	136
CTFDLFLPFVFC	2	0	2	31	126	135
CVYDIFVPFIVC	1	0	1	24	197	135
CIFDIHMPYIPC	0	0	0	11	59	135
CVFDNWLPFIYC	2	0	2	30	226	132
CIFDSFFPFIIC	1	0	1	11	115	131
CTFDSFLPFIFC	0	1	2	11	120	130
CIFDSWLPFIYC	0	1	1	9	103	129
CTFDVFLPFIYC	0	1	5	33	243	127
CVFDSFLPFITC	1	0	1	15	167	124

CIFDINMPFIPC	0	0	0	5	60	123
CVYDLFLPFICC	0	1	3	25	292	123
CLFDTFYFFIPC	1	2	38	180	474	121
CCFDIFLPFIIC	1	1	0	10	96	120
FDSFLPFIIC	0	0	0	9	84	120
CIFDFHMPFIPC	0	0	1	12	50	120
CIWDSFVPFIIC	0	1	0	2	73	119
CLFDLFCPFIPC	1	0	0	10	161	114
CIFDIHMTFIPC	0	0	1	6	45	112
CIFDLFCPFIMC	1	0	1	44	176	112
CLFDTFVPFIPC	1	1	0	5	184	111
CLFDTMLPFIYC	0	0	44	683	689	110
CIFDTFLPFVFC	1	0	2	2	90	109
RIFDIHMPFIPC	0	0	0	3	52	107
CVYDTFVPFIIC	0	0	2	11	142	105
CIIDIHMPFIPC	2	0	2	5	59	103
CEFDSFLPFIIC	0	1	0	4	69	103
CFYDLFLPFIYC	0	0	3	19	229	102
CLFDLHLPFIPC	0	0	0	7	114	102
CIFDIHMPFISC	0	0	4	18	105	101
CIFDIHMHFIPC	0	0	0	3	40	99
CVFDVFCPFIYC	2	1	1	22	245	98
CIFDSFLPFIIC	0	0	0	1	17	97
CVFDSFLPFVFC	0	0	0	6	96	97
CIFDNWLPFIVC	0	1	0	3	59	97
CIFDIYMPFIPC	1	0	0	3	47	96
CVYDSFLPFIIC	0	0	1	13	89	95
CLYDSFLPFICC	0	0	0	13	206	95
CIFDLFVPFVFC	0	1	0	7	61	95
CIFDIQMPFIPC	0	0	1	3	31	94
CLFDLHLPFIYC	0	0	1	11	154	94
CIFDIHMSFIPC	0	0	0	4	51	93
CIFNIHMPFIPC	0	0	0	6	43	93
CTFDTHLPFIFC	0	1	3	25	297	93
YIFDIHMPFIPC	0	0	0	1	31	92
CFFDPFFFFIPC	0	0	8	31	109	90
CVLDSFLPFIIC	0	0	1	12	100	89
CVFDLFLPFICC	0	1	0	12	139	88
CIFDLFLPFVLC	2	3	68	580	547	88
CIFDTFLPLIFC	0	0	1	5	42	88
CTFDIFLPFIYC	0	0	0	1	34	88
CIFDNHMPFIPC	0	0	0	3	34	87
CVFDIFIPFILC	1	0	0	6	73	86
CVFDSFLPFISC	0	1	1	12	92	85
CTFDTFLPFICC	0	0	0	9	167	84
SIFDIHMPFIPC	0	0	0	5	29	84
CVFDLFLPFVFC	0	1	0	7	63	81
CVFDSWLPFVIC	0	0	0	2	33	81
CIFDLFFPFIPC	0	1	0	12	56	81
CIFDIFVPFILC	0	0	2	8	38	81
CIFDIHMPFIAC	0	0	1	2	47	81
CIFDTLLPFIFC	0	0	2	13	78	80

CVFDSSLPFIIC	0	0	0	13	73	80
CLYDTFLPFIFC	0	0	0	5	62	80
CVYDLFLPFILC	0	1	0	5	145	80
CIFDSFFPFVFC	0	0	30	150	351	80
CIFDIFMPFIYC	0	0	0	0	23	80
CIFDIHMPIIPC	0	0	1	2	27	80
CIFDIHMLFIPC	0	0	0	7	44	78
CIFDTFMPFIFC	0	0	0	6	61	78
CVFDLFCPFIVC	0	0	1	26	135	77
CVFDNFLPFIIC	0	0	2	2	58	77
CIYDIFVPFIIC	0	0	0	9	53	77
CTFDLFVPFIFC	1	0	2	15	93	77
CIFDTFLPFTFC	1	0	0	5	81	75
CIFEIHMPFIPC	1	0	0	4	44	74
CVFDIFLPFIFC	1	1	1	8	37	73
CVFDSFLPFTIC	0	0	0	10	77	73
CVYDSFLPFIFC	1	1	0	3	62	73
CYFDPHFIFILC	0	0	0	7	155	72
CIWDSFVPFIPC	0	2	1	4	148	72
CAFDSFLPFIIC	0	0	1	10	66	71
CVFDLFLPFTYC	0	0	0	34	192	71
CLFDLFLPFIFC	0	0	0	4	41	70
CVFDLFVPYIVC	0	1	53	670	816	70
CCFDIFLPFILC	0	0	5	19	131	70
CFFDPFFLFIPC	0	0	1	25	95	69
CVYDIHMPFIPC	0	1	0	1	17	69
CIFDIHMPFIYC	0	0	0	3	64	68
CIFDILMPFIPC	0	0	1	7	54	68
CIFDLWLPFIPC	0	0	0	3	22	68
CTFDVFLPFIIC	0	0	1	8	121	68
CVFGSFLPFIIC	1	0	0	7	41	67
CVSDSFLPFIIC	0	0	3	5	40	67
CIFDIHMPFIPR	0	0	0	4	31	67
CVFDSFSPFIIC	0	0	0	7	52	67
CTFDTHFPFIPC	0	0	0	8	184	67
CLFDTHFPFILC	0	0	1	33	81	66
CLFDTMLPFIFC	1	0	2	34	129	66
CVFDSFLPSIIC	0	1	1	6	44	65
CVFDIFIPFIFC	1	0	0	3	53	65
CIFDLFVPFICC	0	0	1	11	95	64
CIFDTHFPFITC	0	0	1	9	52	64
CFFDLFLPFIFC	0	0	4	36	109	63
CIFDSFLPFIPC	0	0	3	21	56	63
CIFDSWLPFIFC	0	0	0	1	12	63
CVFDTFLPFIIC	1	0	0	0	11	63
CVFDTFLPFILC	0	0	1	0	31	63
CTFDLFIPFVFC	0	0	8	88	220	62
CIFDLFCPFIYC	0	0	0	3	45	62
CVFDSFLPFICC	0	0	1	1	25	61
CLYDLFLPFIFC	0	0	3	23	94	60
FDLFCPFVIC	2	0	40	448	504	60
CVFDVFCPFIPC	0	0	0	5	120	60

CTFDLFLPFTFC	0	0	2	54	141	59
CVFDSWLPFIYC	2	0	0	3	54	59
CIFDTFMPFIPC	0	0	0	1	16	59
CLFDSFVPFICC	1	0	2	18	113	59
CIFDTFSPFIFC	0	0	1	10	42	59
CLYDSLLPFIFC	1	0	27	328	314	58
CLFDIFLPFICC	1	0	1	9	86	57
CIFVIHMPFIPC	0	0	1	5	28	57
CIFDLFLPFIYC	0	0	0	0	33	57
CVFDSFMPFIIC	0	0	0	3	48	56
CTFDLFLPLIFC	0	0	2	23	96	56
CVFDIFMPFIPC	0	0	0	2	20	56
CLFDIFLPFIMC	0	0	6	27	97	55
CLFDIFLPFVFC	0	0	0	22	53	54
CVFDSFMPFIFC	0	0	0	2	31	54
CIFYIHMPFIPC	0	0	0	2	26	53
CIFDTHFPFIPC	0	0	1	2	19	53
CFYDLFLPFIIC	1	0	0	2	155	53
CTFDVFLPFIPC	0	0	0	4	119	53
CLFDLHLPFIIC	0	0	1	8	91	52
CVFDIFMPFIFC	0	0	0	1	17	52
CVFDIFCPFIFC	0	0	0	8	55	52
CIFDIHLPFIPC	0	0	0	5	32	52
CLFDLFVPFVYC	1	1	3	36	148	52
CIFDSFLPFVIC	0	0	0	0	11	52
CLFDSFIPFIFC	0	0	0	15	44	51
CVFDLFFPFTPC	0	2	74	441	403	50
CVFDLFCPFIFC	0	0	0	3	34	50
CTFDIFMPFILC	0	0	0	13	115	50
CCFDSFLPFICC	0	0	0	10	128	50
CTFDSLLPFIFC	3	2	30	327	485	49
CLFDSFLPFIYC	0	0	0	1	45	49
CVFDIFIPFVPC	0	0	4	26	118	49
CTFDNWLPFIYC	0	1	1	19	134	49
CIFDLHMPFIPC	0	0	0	1	12	49
CIFDTHMPFILC	0	0	0	2	22	48
CVYDIFVPFITC	0	0	0	16	94	48
CIFDTSLPFIFC	0	0	0	9	32	47
CLFDIFLPFIVC	0	0	1	16	68	47
CLYDSFLPLIFC	1	0	2	14	61	47
CAFDLFLPFIFC	2	0	0	20	79	47
CFYDLFLPFIPC	0	0	0	4	126	47
CVFDLFLPFIVC	0	0	0	5	37	46
CIFDIFLPFILC	0	0	0	5	48	46
CVFDPFLPFIFC	0	0	0	16	52	46
CIFDSFFSFIPC	1	1	32	229	301	46
CLFDLHEPFIYC	0	0	0	1	23	46
CVFDGFLPFIIC	0	0	0	3	31	46
CIFDVFLPFIFC	0	0	0	7	42	46
CFFDPHFIFIPC	0	0	0	2	23	46
CVFDSFLPFIIS	0	0	0	4	26	45
CVYDIFVPLIIC	0	1	0	13	66	45

CVFDSFVPFIFC	0	0	0	3	28	45
CVFDLFSPFIFC	1	0	2	5	66	45
CLFDSFMPFIFC	0	0	2	15	71	45
CIFDLFSPFIIC	0	0	0	0	58	45
CIFDNWLPLIYC	0	0	0	14	78	44
CVFDSFLPYIIC	0	0	2	4	37	44
CTFDLFLPFIVC	0	1	2	12	63	44
CIFDIFLPFIIC	0	1	0	2	19	44
CIFDIHMPFIRC	0	0	0	8	52	44
CCFDIFLPFIPC	0	0	0	1	81	43
FIFDIHMPFIPC	0	0	0	6	16	43
CFFDPHLPFIFC	0	0	19	167	247	43
CIWDSFVPFIYC	2	0	0	4	113	42
CIFDTFLPFISC	0	0	1	10	34	42
CFFDLFYPFIPC	1	2	45	237	250	42
CIFDTFLPFVIC	0	0	0	0	9	41
CLFDLFVPFVIC	0	0	0	7	63	41
CILDTFLPFIFC	0	0	1	10	46	40
CIFDTFMPFILC	0	0	0	3	57	40
CTFDLFIPFVLC	3	0	74	662	635	40
CIYDSFLPFIFC	0	0	0	0	53	40
CVFDRFLPFIIC	0	0	0	2	17	40
CDYDIFVPFIIC	0	0	0	4	43	39
CVFDIFLPFIIC	0	1	0	0	16	39
CTFDSWLPYILC	0	0	36	358	388	38
CLFDTFLPFICC	1	0	0	11	76	38
CAYDIFVPFIIC	0	0	0	12	42	37
CIFDLFLPFIPC	0	0	0	3	14	37
CIFDVFCPFIFC	0	0	1	10	48	37
CTFDPFLPFIFC	0	0	2	45	82	37
CVFDNWLPFIFC	0	0	0	2	28	37
CIYDTFLPFIFC	0	0	0	7	20	37
CLFDPSLPFIPC	0	0	0	8	45	36
CFFDLFCPFIYC	0	2	31	231	287	36
CLFDIFCDFIFC	0	0	0	9	27	36
CTWDSFVPFIFC	0	0	1	4	25	36
CTFDLFMPFIFC	1	0	0	3	34	36
CTFDLLLPFIFC	0	0	3	21	58	36
FDTFLPFIFC	0	1	0	7	42	35
CIFDTFLPSIFC	0	0	0	4	43	35
CIWDSFVPFIVC	0	0	0	7	92	35
CTFDVFLPFILC	0	0	4	42	158	35
CVFDLFVPYIIC	0	0	0	32	70	35
CVFDSFLPFINC	0	0	0	3	53	35
CLFDTFIPFIFC	0	0	1	18	39	35
CTFDSWLPYIIC	0	0	2	14	93	34
CIFDTHFPFIIC	0	0	0	1	6	34
CCFDTFLPFIFC	0	0	2	13	73	34
CIFDLFSPFIYC	0	0	1	5	73	34
CVFDSFFPFIFC	0	0	1	14	64	34
CLFDLHLPFILC	0	1	5	20	117	34
CVFDSFLTFIIC	1	0	0	2	18	34

CVFDSFLPFIIY	0	0	1	0	31	34
CVFDIFIPFIPC	0	0	0	0	69	34
CIFDIHMPFFPC	0	0	0	9	15	33
CFFDLFFPFYPC	3	4	45	735	382	33
CIFDSFLPFVLC	0	1	0	3	31	33
CLFDIFFPFILC	0	0	31	305	206	33
CVFDIFVPFIIC	0	0	0	1	13	33
CVFDSYLPFIIC	0	0	0	1	31	33
CLFDTFVPFICC	0	0	1	15	101	32
CIFDIHMPFIVC	0	0	0	0	15	32
CIFDLFAPFIPC	0	0	1	2	64	32
CIFDLFLPFVPC	0	1	5	103	126	32
CLFDSFVPFVYC	0	0	5	20	171	32
CTFDLFLPSIFC	0	0	0	20	49	32
CIFDSFCPFIVC	0	0	1	23	93	32
CTFDTFLPLIFC	0	0	0	11	48	32
CVYDIFLPFIFC	0	0	0	2	29	32
CLFDIFCPFIYC	0	0	0	1	15	31
CIFDSFMPFIFC	0	0	0	0	14	31
CFFDSFMPFIFC	2	0	28	146	241	31
CIFDAFLPFIFC	1	0	0	4	38	31
CIFDLFLPFIIC	0	0	0	0	9	31
CIFDLFVPFIVC	0	0	0	3	40	31
CIFDNWLPFVYC	0	0	1	5	60	31
CHYDSFLPFIFC	0	0	0	7	23	31
CTLDLFLPFIFC	0	0	2	23	54	31
CTSDLFLPFIFC	0	0	0	17	40	31
CVFDLFLPLIFC	0	0	0	11	34	31
CVFNSFLPFIIC	0	0	0	4	18	31
CVYDIFVPFISC	0	0	0	7	53	31
RVFDSFLPFIIC	0	0	0	0	15	31
CLFDSFVPFVLC	1	0	7	33	171	31
CIFDSFIPFIFC	0	0	0	1	22	30
CIFDIFMPFIVC	0	0	0	5	18	30
CIFDTFLPFIFY	0	0	0	3	16	30
CVFDSWLPFVLC	2	0	0	1	42	30
CLFDSLLPFIIC	0	0	0	2	31	30
CLFDLFCPFICC	0	0	1	12	94	30
CFFDTFLPFIFC	0	0	0	4	23	29
CLFDIFVPFIFC	0	0	0	3	19	29
CLFDSFVPFVIC	0	0	0	13	91	29
CLFDSFVPLIFC	0	0	1	28	47	29
CTFDLFSPFIFC	0	0	0	14	54	29
CVFDSFLPFTFC	0	0	0	0	9	29
CVFDSLLPFIPC	0	0	0	2	58	29
CVYDIFMPFIIC	0	0	0	4	25	29
CYFDPHFILIPC	0	0	1	14	26	29
CLFDLFVPFVLC	0	0	4	33	146	28
CIFDLFLPFILC	0	1	0	3	21	28
CIFDSHMPFIPC	0	0	0	2	18	28
CLFDLFFPFILC	1	0	2	34	67	28
CIFDIFVPFIIC	1	0	1	0	5	28
CVFDTFIPFILC	0	0	1	4	20	28
CVYDSFLPFIYC	0	0	0	16	68	28
CLYDSFMPFIFC	0	0	0	4	19	28
CVFDSILPFIIC	0	0	0	4	14	28
CLFDLFLPFIIC	0	0	3	42	71	27
CIFDLFSPFIPC	0	1	0	3	33	27
CLFDLFYPFIPC	0	0	1	17	48	27
CTFDTFLPFVFC	0	0	0	7	68	27
CTFDTHLPFIIC	0	0	0	0	29	27
CVYDILVPFIIC	0	0	0	11	39	27
CYFDIHMPFIPC	0	0	0	0	8	27
CLYDSFLPFTFC	0	0	0	11	74	27
CLYDSFVPFIFC	0	0	0	7	29	26
CIFDIFVPFIFC	0	0	0	0	8	26
CIFDIHMPFIQC	0	0	0	1	10	26
CIFDIWLPFIYC	0	0	0	4	28	26
CLYDSFLPSIFC	0	0	1	7	14	26
CVYDLLLPFIYC	0	0	2	12	39	26
CTFDLSLPFIFC	0	1	0	18	56	26
CVFDSFLPFNIC	0	0	1	5	18	26
CVFDSFVPFIIC	0	0	0	1	14	26
CVYGIFVPFIIC	0	1	1	3	27	26
CYFDPHFIFTPC	0	1	1	28	64	26
CLYDSFSPFIFC	0	0	0	11	44	26
CLFDLFLPYVFC	1	0	35	436	401	25
CDFDLFLPFIFC	0	0	0	7	21	25
CIFDIFLPFIYC	1	0	0	0	5	25
CIFDNWPPFIYC	0	0	1	8	45	25
CIFGTFLPFIFC	0	0	0	3	18	25
CLFDIFLPFTFC	0	0	1	22	47	25
CLFDLFYPFIFC	0	0	0	19	149	25
CTFDTHFPFILC	0	0	0	1	25	25
CVFDSFLSFIIC	0	0	0	2	17	25
CVYDLFLPLIYC	0	1	0	15	41	25
CPYDSFLPFIFC	0	1	1	14	42	25
CIFDTYLPFIFC	0	0	0	3	19	24
CIFDIFLPFIPC	1	0	0	0	9	24
CIFDIFMPFIRC	0	0	0	9	55	24
CIFDSFFPFIYC	1	1	0	4	85	24
CLFDIFLPFISC	0	0	1	15	30	24
CLYDLFLPFIYC	0	0	0	4	27	24
CVFDLFMPFIFC	0	0	0	1	25	24
CVLDLFLPFIFC	0	1	0	5	32	24
CVYDIFVPFTIC	0	0	0	9	31	24
CVYDIFVPFVIC	0	0	0	8	44	24
CVYDLFLPFTYC	0	0	2	17	65	24
CISDTFLPFIFC	0	0	0	4	37	23
CAYDLFLPFIYC	0	0	0	6	20	23
CLLDSFVPFIFC	0	0	0	22	44	23
CIFDIFMPFTPC	0	0	0	2	20	23
CFYDSFLPFIFC	0	0	1	7	35	23
CIFDINMPFITC	0	0	0	1	7	23

CVFDLFLPFTIC	1	0	0	4	37	23
CVFDLWLPFYFC	0	2	16	189	231	23
CTFDLFCPFIVC	0	0	3	14	62	23
CVFDSFLPSILC	0	0	1	3	14	23
CVFDTFLPFIYC	0	0	0	1	10	23
CVFDSFLPLILC	0	1	0	5	30	23
CVFDIHMPFILC	0	1	0	2	31	23
CIFDVFCDFIFC	0	0	0	13	31	22
CLFDLFVPFVCC	0	0	0	10	61	22
CLFDIFSPFIFC	0	0	1	14	46	22
CLFDIFLPFITC	0	0	1	4	34	22
CIWDSFLPFIFC	0	0	0	0	15	22
CFYDLFLPFILC	0	2	0	18	164	22
CIFDTFLPFVLC	0	0	1	2	24	22
CIFDLWLPFIAC	0	0	1	4	10	22
CLFDTFLPFVFC	0	0	0	5	28	22
CLFDSFLPFVFC	1	0	2	19	24	22
CLYDSFLPFISC	0	0	2	12	50	22
CVFDLHCPFIYC	0	0	1	11	71	22
YIFDTFLPFIFC	0	0	0	1	7	22
CLYDNLLPFILC	2	0	36	159	231	22
CVWDSFVPFIFC	0	0	0	2	30	22
CVFDSFFPFIIC	0	0	0	0	12	22
SVFDSFLPFIIC	0	0	0	0	13	22
CVFDLHCPFIIC	0	0	0	5	48	22
CVFDLFLPFTCC	0	0	0	4	55	22
CTFDSFLPFIIC	0	0	0	0	9	22
CTFDIFMPFIPC	0	0	0	1	10	22
CSFDIHMPFIPC	0	0	0	3	9	21
CCFDLFFPFIVC	0	1	12	108	122	21
CIFDIFMPLILC	1	0	0	10	37	21
CIFDMHMPFIPC	0	0	0	5	15	21
CIFDTFLPFIFS	0	0	0	0	18	21
CLFDLFCPFIVC	0	0	1	18	42	21
CLFDTFLPFIVC	0	0	0	7	33	21
CVFDVFCPFICC	0	0	0	6	82	21
CVFDLSLPFIFC	0	0	0	8	17	21
CTFDTFLPFIVC	0	0	0	4	45	21
YVFDSFLPFIIC	0	0	0	0	5	21
CVFDSFMPFIPC	0	0	0	0	11	21
CVFDTFVPFIFC	0	0	0	0	21	21
CVFDSLLPFILC	1	0	2	16	82	21
CVYDIFLPFIIC	0	0	0	3	17	21
CVYDLFLPSIYC	0	0	0	5	32	21
CVLDSFLPFILC	0	1	0	8	21	21
CCFDIHMPFIPC	0	0	0	1	11	20
CIFDNRLPFIYC	0	0	0	14	59	20
CTFDIFMPFIFC	0	0	0	1	11	20
CIFDIHMPFINC	0	0	0	1	8	20
CIFDLFSPFILC	0	0	1	13	65	20
CLFDPFCPFIFC	0	0	1	68	56	20
CIFDSFFPFVIC	0	0	0	10	31	20

CIFDTFLHFIFC	0	0	0	0	13	20
CLFDLFLPFIMC	1	0	61	537	266	20
CLHDSFLPFIFC	0	0	0	5	20	20
CLYDSYLPFIFC	0	0	0	2	18	20
CFFDIFLPFIFC	0	0	0	0	25	20
CIFDPFVPFIFC	1	0	3	7	51	20
CVYDIFEPFIIC	0	0	0	2	26	20
CTFDSWLPYIFC	0	0	2	16	72	20
CVFDSFLHFIIC	0	0	0	3	17	20
CTFDTFPPFIFC	0	0	4	8	25	20
CVFDIFMPFIIC	0	0	0	0	4	20
CVFDLFLPFTLC	1	0	2	17	80	20
CVWDSFVPFILC	0	0	1	13	79	20
CTWDSFVPFILC	0	0	1	11	51	20
CTFDTHLPFIPC	0	0	0	5	87	20
CVFDLFFPFIPC	0	0	0	11	38	20
CVFDLFVPFIIC	0	0	0	0	10	20
CVFDSFSPFIFC	0	0	0	0	10	20
CVFESFLPFIIC	0	0	0	2	21	20
CTFDLFLPFISC	0	0	1	16	37	20
CVFDSFLLFIIC	0	0	0	0	21	20
CAFDTFLPFIFC	0	0	1	7	48	19
CIFDLFVPLIFC	0	0	0	5	21	19
CILDNWLPFIYC	1	0	1	19	36	19
CIFDSFFPFIVC	0	0	0	4	41	19
CIFGNWLPFIYC	0	0	1	6	29	19
CLFDIFLPLIFC	1	0	0	18	27	19
CLFDIFMPFIFC	0	0	0	7	16	19
CIFDLFYPFIVC	0	0	0	2	22	19
CLFDIFVPFIIC	0	0	0	0	9	19
CVLDSFLPFIFC	0	0	0	0	9	19
CLFDLHMPFIPC	0	0	0	1	6	19
CVFDIFIPFIIC	0	0	0	0	10	19
CVFDSWLPFIIC	0	0	0	0	5	19
CPFDTFLPFIFC	0	0	0	10	18	19
CTFDVFLPFICC	0	0	3	7	45	19
CLFDTFPPFIFC	0	0	0	9	19	19
CVFDIFCPFILC	0	0	0	1	12	19
CVFDSFFPFIPC	0	0	3	29	58	19
CVFDSFLPFIRC	0	1	0	4	12	19
CVFDSWLPFVCC	0	1	2	19	66	19
CLYDPFLPFIFC	0	0	1	15	41	19
CIFHIHMPFIPC	0	0	0	1	5	18
CIFDTFVPFIIC	0	0	0	0	10	18
CLFDPFVPFIFC	0	0	0	27	38	18
CIFDTFLTFIFC	0	0	0	0	9	18
CIFDTFFPFIPC	0	1	0	4	18	18
CCLDSFLPFIFC	0	0	0	10	45	18
CFFDLDLPFVFC	1	1	34	426	341	18
CCFDSFLPLIFC	0	0	0	9	34	18
CLFDIFLPFVIC	0	0	0	5	23	18
CIFDLFCPLIVC	0	0	0	9	36	18

CVYDISVPFIIC	0	0	0	6	24	18
CDFDSFLPFILC	0	0	0	3	13	18
CVVDSFLPFIIC	0	0	0	0	12	18
CNFDTFLPFIFC	0	0	0	0	11	18
CVYDVFVPFIIC	0	0	0	4	19	18
CVYDIFAPFIIC	0	0	0	9	41	18
CLYDSSLPFIFC	0	0	1	10	31	18
CVIDSFLPFIIC	0	0	0	2	14	18
CVFDSWLPFILC	0	0	0	0	9	18
CSFDLFLPFIFC	0	0	0	9	30	18
CIFDIFIPFILC	0	0	0	5	24	17
CFYDLFLPFICC	0	0	0	2	67	17
CLFDLFCPFVFC	0	0	0	12	24	17
CIFDTFLPFITC	0	0	0	0	6	17
CIFDIFIPFVLC	0	0	0	0	42	17
CIFDINMPFIHC	0	0	0	1	1	17
CIFDLFVPFTFC	0	0	0	5	36	17
CIFDNWLPSIYC	0	0	0	10	37	17
CLFDLFVPFIIC	0	0	0	1	17	17
CIYDIHKPFIPC	0	0	0	1	6	17
CLFDSFLPLIFC	0	0	0	1	3	17
CCFDPFLPFIFC	0	0	3	11	27	17
CLYDNLLPFIIC	0	0	2	10	29	17
CVFVSFLPFIIC	0	0	0	5	19	17
FDIHMPFIPC	0	0	0	1	9	17
CTFGLFLPFIFC	0	0	0	14	32	17
CTFGTFLPFIFC	0	0	0	7	23	17
CTYDLFLPFIFC	0	0	0	6	21	17
CVFDLFLPFISC	0	0	1	11	27	17
CVFDLHCPFIPC	0	0	0	2	26	17
CVFDSFLPIIIC	0	0	0	4	17	17
CVFDSFMPFILC	0	0	0	1	15	17
CVFDSLLPFIYC	0	0	1	7	75	17
CLFDTFVPLIFC	0	0	1	24	28	17
CVYDLSLPFIYC	0	0	0	9	28	17
CVFDSFLPLIFC	0	0	0	0	6	17
CIFDSFIPFILC	0	0	0	2	11	16
CLFDTFYSFIPC	0	0	0	7	29	16
CCFDSLLPFIFC	0	0	0	13	20	16
CFFDLHLPFIFC	0	0	1	4	33	16
CIFDIHMRFIPC	0	0	0	0	2	16
CAFDSFLPFIFC	0	0	0	1	5	16
CLFDIFLPLIIC	0	0	2	12	32	16
CIFNTFLPFIFC	0	0	0	0	8	16
CIWDSFVPFICC	0	0	1	0	29	16
CLFDLLCPFIFC	0	1	0	22	24	16
CLFDSFVPFTFC	0	1	0	11	45	16
CLFDSLVPFIFC	0	0	0	15	27	16
CIFDSFLPFIYC	0	0	0	0	7	16
CLYDSFLPFVFC	0	0	0	7	48	16
CIFDNWLPFIHC	0	0	1	7	38	16
CCFDSFLPFIVC	0	0	0	5	43	16
CVFDTFLPFIPC	0	0	0	0	6	16
CTFDLFLPFNFC	0	0	0	2	7	16
CTFDLYLPFIFC	0	0	0	8	22	16
CTFDTHFPFIFC	0	0	0	2	29	16
CTFDTLLPFIFC	0	0	2	14	39	16
CLYGSFLPFIFC	0	0	1	5	23	16
CTSDTFLPFIFC	0	0	0	4	13	16
CVFDVFCPFIVC	0	0	0	9	45	16
CVFYSFLPFIIC	0	0	0	1	9	16
RIFDTFLPFIFC	0	0	0	3	8	16
CYFDPHFTFIPC	0	0	0	13	41	16
CRFDSFLPFIFC	0	0	0	3	16	16
CIFDILMPFILC	0	0	0	5	15	15
CIFDIFMPFICC	0	0	0	0	5	15
CIFDIHRPFIPC	0	0	0	1	8	15
CIFDTFLPYIFC	0	0	0	1	22	15
CIFDLFLPFVYC	1	0	0	7	18	15
CIFDLLVPFIFC	0	0	0	4	29	15
CIFDTFVPFILC	0	0	0	1	11	15
CIFDSFCPFIFC	0	0	0	1	9	15
CIFDNWLPFVFC	1	0	0	0	12	15
CTFNLFLPFIFC	0	1	0	3	13	15
CLFDLFFPFIFC	0	0	1	8	58	15
CPFDTFVPFIFC	0	0	2	6	29	15
CVFDSFLPFVPC	1	0	0	15	37	15
CVFDSSLPFILC	0	0	0	2	13	15
CVFDCFLPFIIC	0	0	0	2	15	15
CVFDIHMPFTPC	1	0	0	1	13	15
CVFDLLLPFIFC	0	0	1	7	16	15
CVFDNFLPFIFC	1	0	0	0	5	15
CVFDSFLPFIFY	0	0	0	0	4	15
CVFDLFAPFIYC	0	1	0	11	51	15
CYFDPHFIFITC	0	0	0	8	24	15
CVHDIFVPFIIC	0	0	0	3	20	15
CVYDIFVPFICC	0	0	0	0	22	15
CVYDIFVPSIIC	0	0	0	4	38	15
CYFDPHLIFIPC	0	0	2	8	17	15
CYSDPHFIFIPC	1	0	0	4	13	15
CLFDILLPFIFC	0	0	0	14	18	14
CCFDIFLPFICC	0	0	0	1	36	14
CEYDLFLPFIYC	0	0	1	11	36	14
CFFDLSLPFVFC	0	1	26	209	150	14
CICDIHMPFIPC	0	0	0	1	5	14
CIFDIHMPFSPC	0	0	0	1	7	14
CIFDINMPFINC	0	0	0	1	2	14
CIFDLFCPFTVC	0	0	0	13	22	14
CLFDILLPFIIC	0	0	0	5	21	14
CIFDNWLPFISC	1	0	0	6	10	14
CIFDNWLPLIFC	0	0	0	0	10	14
CIFDSFLPFVYC	0	0	0	2	17	14
CILDLFVPFIFC	1	0	0	8	20	14
CTFDLFLTFIFC	0	0	0	4	9	14

CLFDTLLPFIFC	0	0	0	15	28	14
CTFDLFLSFIFC	0	0	0	4	16	14
CLFDVFCDFIYC	0	0	1	14	85	14
CPFDSFVPFIFC	2	0	2	17	30	14
CLLDIFLPFIFC	0	0	1	14	18	14
CPFDIFLPFIFC	0	0	0	13	22	14
CTFDLFVPFIIC	0	0	0	0	3	14
CVFDPFLPFILC	0	0	0	10	24	14
CTFDNWLPFIFC	0	0	0	1	9	14
CVSDLFLPFIFC	0	0	1	8	17	14
CLFDLFVPFILC	0	0	0	1	23	14
CVYDSFLPFILC	0	0	0	0	12	14
CTLDTFLPFIFC	0	0	1	9	41	14
CTFDTHMPFIPC	0	0	0	0	6	14
CLFDSFAPFIFC	0	0	0	24	24	13
CCFDSFLPFVFC	0	0	0	8	60	13
CIFDIFMPFITC	0	0	0	0	9	13
CIFDIFMPFVLC	0	0	0	9	27	13
CIFDIFTPFILC	0	0	0	4	30	13
CIFDIHMAFIPC	0	0	0	1	8	13
CIFDIHMPFIPG	0	0	0	1	2	13
CIFDTHFPFIFC	0	0	0	0	3	13
CIFDLFAPFICC	0	0	0	4	42	13
CIIDTFLPFIFC	0	0	2	2	17	13
CLFDLFRPFIFC	0	0	0	18	10	13
CIFDLFEPFIYC	0	0	0	1	8	13
CLFDISLPFIFC	0	0	2	7	14	13
CLFDIFFPFIPC	0	0	4	24	37	13
CIYDIHMPFITC	0	0	0	0	4	13
CLFDSLLPFILC	0	0	2	23	75	13
CIFDTFLPLILC	0	0	0	0	7	13
CIFDSFYPFIFC	0	0	0	1	21	13
CIFDNWLPFIYS	0	0	0	2	19	13
CIFDLFIPFIFC	0	0	0	2	8	13
CIFDINMPLIPC	0	0	0	0	2	13
CLFDTFLPFISC	0	0	1	12	18	13
CVYDLFMPFIYC	0	0	0	0	21	13
CLFDTFLPLIFC	0	0	0	10	31	13
CTFDIFLPFICC	0	0	0	0	10	13
CTFDAFLPFIFC	0	0	0	14	56	13
CLSDSFVPFIFC	0	0	0	11	20	13
CNIDIHMPFIPC	0	0	0	0	6	13
CNFDNWLPFIYC	0	0	0	2	18	13
CLFDVFCDFIIC	0	0	1	15	63	13
CLFDTFVPFTFC	0	0	1	8	34	13
CTFDTFQPFIFC	0	0	1	2	15	13
CTFDTFLPSIFC	0	0	0	6	24	13
CTFDTFLPFTFC	0	0	1	13	49	13
CVFDIFIPFVYC	0	0	0	2	21	13
CVYDIFMPFILC	0	0	0	0	10	13
CVFDLFLPFVLC	0	0	2	11	37	13
CYFDPHFIFIHC	0	0	0	4	27	13
CVYDIFVPFINC	0	0	0	6	45	13
CVYDIFVPFIIS	0	0	1	0	16	13
CVFGLFLPFIFC	0	0	0	4	11	13
CVFDTHMPFIPC	0	0	0	3	8	13
CVFDTFIPFVLC	1	0	0	4	37	13
CVFDSFSPFILC	1	0	0	5	12	13
CVFDLFLPSIFC	0	0	0	9	23	13
CIFDIHMPCIPC	0	0	0	1	3	12
CIFDIFMPFTLC	0	0	1	11	31	12
CIFDIFMPFILY	0	0	0	2	4	12
CLFDLFVPFIPC	0	0	0	1	14	12
CCFDSFLPSIFC	0	0	0	9	13	12
CCFDPHFIFIPC	0	0	2	9	24	12
CLFDIFSPFIIC	0	0	0	7	24	12
CIFDIPMPFIPC	0	0	0	2	2	12
CIFDIHMPFVLC	0	0	0	1	29	12
CIVDIHMPFIPC	0	0	0	0	7	12
CIFDLFAPFIIC	0	0	0	0	21	12
CIFDTFMPFIYC	0	0	0	0	2	12
CLFDIFLPYIFC	0	0	0	4	16	12
CIFDLLCPFIVC	0	0	0	18	26	12
CIYDLFLPFIFC	0	0	0	0	14	12
CIFETFLPFIFC	0	0	0	0	6	12
CISDLFVPFIFC	1	0	1	2	17	12
CIFDTFLSFIFC	0	0	0	1	13	12
CIFDTFLPIIFC	0	0	0	0	17	12
CIFDTFFPFIFC	0	0	0	1	6	12
CIFDLWLPFTVC	0	0	0	19	52	12
CLFDSFIPFVFC	0	0	0	6	24	12
CLFDTFAPFIFC	0	0	0	12	27	12
CIFDTFLLFIFC	0	0	0	1	4	12
CLFDLYLPFIPC	0	0	4	16	30	12
FVFDSFLPFIIC	0	0	0	0	12	12
CYLDPHFIFIPC	0	0	0	14	16	12
CTFDTHLPFILC	0	0	0	3	30	12
CTFDTFLPFISC	0	0	0	12	34	12
CTFDLFLPFIFY	0	0	0	4	18	12
CTFDLFLPFIFS	0	0	0	3	19	12
CLFDSFLPFICC	0	0	0	0	18	12
FDSFLPFIFC	0	0	1	0	10	12
CLYDSFLPFIVC	0	0	0	4	50	12
CLFDSFVPFVCC	0	0	0	6	88	12
CVFDIFLPFILC	0	0	0	3	11	12
CSFDTFLPFIFC	0	0	0	0	22	12
CVFDIHMPFIFC	0	0	0	0	4	12
CVFDSFLPFILS	0	0	0	2	13	12
CVFDLFLPFNFC	0	0	0	0	8	12
CYFDSFLPFIFC	0	0	0	3	31	12
CYFDPHYIFIPC	0	0	0	7	16	12
CVYDLFSPFIYC	0	0	2	7	43	12
CVFDSFLPFVMC	1	0	1	13	56	12
CVYDIFVLFIIC	0	0	0	4	19	12

CVFDSFLPFIIR	0	0	0	1	12	12
CVFDSFLPFIHC	0	0	0	0	6	12
CVFDLFMPFIPC	0	0	0	0	4	12
CIFDLFLPFTFC	0	0	0	3	19	11
CLFDLFSPFIFC	0	0	1	6	13	11
CCFDSFSPFIFC	0	0	0	6	20	11
CCFDSSLPFIFC	0	0	1	8	31	11
CIFDLFCPFIAC	0	0	1	20	42	11
CFYDIFVPFIIC	0	0	0	2	7	11
CAFDSFLPFILC	0	0	0	4	17	11
CIFDIFMPLIPC	0	0	0	1	3	11
CIFDIHKPFITC	0	0	0	0	7	11
CIFDLFVPFISC	0	0	1	4	16	11
CIFDNFLPFIFC	0	0	0	0	9	11
CIFDSWLPFIIC	0	0	0	0	2	11
CISDNWLPFIYC	0	0	2	15	35	11
CISDLFCPFIVC	0	0	0	9	13	11
CIFDNWLPFTYC	0	0	1	12	53	11
CILDIFMPFILC	0	0	2	11	28	11
CHFDPHFIFIPC	0	0	1	6	11	11
CIFDSWLPFILC	0	0	0	0	2	11
CIIDIHMPYIPC	0	0	0	0	4	11
CLFDIFMPFILC	0	0	0	1	16	11
CIFDNWHPFIYC	0	0	1	4	17	11
CLFDAFLPFIFC	0	0	0	7	6	11
CLFDHFLPFVYC	1	1	37	279	241	11
CLFDIFLPSIFC	0	0	1	9	11	11
CLFDIFMPFIIC	0	1	0	1	11	11
CLFDLFCPFTFC	0	0	1	14	27	11
CLFDLFLPFIPC	0	1	2	30	27	11
CLFDNWLPFIYC	0	0	0	5	21	11
CLFDTFVPFVFC	0	0	0	20	56	11
CVFDFFLPFIFC	1	0	1	4	29	11
CTFDLFLPFVIC	0	0	0	1	9	11
CVFDLHCPFILC	2	0	1	16	70	11
CLFDTLVPFIFC	0	1	0	20	38	11
CLYDSFLPFIFS	0	0	0	1	14	11
YDLFLPFIYC	0	0	0	6	22	11
CNFDLFLPFIFC	0	0	0	0	4	11
CTFDTSLPFIFC	0	0	0	7	24	11
CVCDIFVPFIIC	0	0	1	5	12	11
CVFDSFLPFILY	0	0	0	2	6	11
CVFDLFCPFIIC	0	0	0	0	3	11
CVFDLFCPFILC	0	0	0	2	11	11
CVFDLFLPYIFC	0	0	0	1	24	11
CVFDLFVPFIYC	0	0	0	2	14	11
FDLFCPFIIC	0	0	1	5	15	11
CVFDNWLPFIIC	0	0	0	1	2	11
CVYNIFVPFIIC	0	0	0	2	11	11
CVFDSFLPFTLC	0	0	1	6	28	11
CVFDSFLPYIFC	0	0	0	0	5	11
CVFDSFLLFILC	0	0	0	1	4	11

CVHDLFLPFIYC	0	0	0	6	11	11
CVYDIFVQFIIC	0	0	0	0	16	11
CVYDIIVPFIIC	0	0	0	3	7	11
CVYDTFLPFIFC	0	0	0	0	4	11
CVFGSFLPFILC	0	0	0	2	11	11
RLFDSFVPFIFC	0	0	0	5	10	11
RTFDLFLPFIFC	1	0	0	3	9	11
SIFDTFLPFIFC	0	0	0	0	5	11
CLFDSFVPSIFC	1	0	0	14	16	11
CYFDIFLPFIIC	0	0	0	2	26	11
CIFDIDMPFIPC	0	0	0	0	3	10
CFFDTFVPFIFC	0	0	0	7	8	10
CCFDSFMPFIFC	0	0	0	4	14	10
CEFDSFLPFIFC	0	0	0	0	9	10
CCSDSFLPFIFC	0	0	1	6	25	10
CLFDHFFPFIVC	1	0	43	192	138	10
CIFDIFMPSILC	0	0	0	8	18	10
CIFDIFMPFVFC	0	0	0	0	5	10
CLFDTMLPFIPC	0	0	0	3	19	10
CIFDIHMPFICC	0	0	0	0	11	10
CIFDTWLPFIYC	0	0	0	2	12	10
CIFDIHMPFIPF	0	0	0	1	4	10
CLFDAFVPFIFC	0	0	0	5	16	10
CLFDAFCDFIFC	0	0	0	17	41	10
CIYDNWLPFIYC	0	0	0	8	29	10
CKFDTFLPFIFC	0	0	0	1	13	10
CIFDTFLPFLCC	0	0	0	1	7	10
CIFDSFVPFIIC	0	0	0	1	3	10
CIFDPHFIFIPC	0	0	0	0	8	10
CIFDLHCPFIFC	0	0	0	2	16	10
CIFDIHMPVIPC	0	0	0	0	2	10
CLFDLFLPFVFC	0	0	0	7	9	10
CIFDDWLPFIYC	0	0	0	5	19	10
CLFDTFVPFIVC	0	0	0	8	27	10
CLFDLFVPFIYC	0	0	0	0	21	10
CVFDAFCPFIFC	0	0	1	19	48	10
CTIDLFLPFIFC	0	0	0	4	19	10
CTFDSWLPFILC	0	0	0	0	14	10
CTFDIFMPFIIC	0	0	0	0	3	10
CLYDSFLTFIFC	0	0	0	2	12	10
CLLDLFCPFIFC	0	0	0	10	19	10
CLLDIFLPFIIC	0	0	0	4	13	10
CVFDLFVPYIFC	0	0	0	7	36	10
CLFDTSLPFIFC	0	0	0	12	14	10
CVFDLFFPFIFC	0	0	0	0	10	10
CLYDIFLPFIFC	0	0	0	6	19	10
CVFDSFLPFTPC	0	0	0	2	4	10
CVYDLFLPFIYS	0	0	0	0	17	10
RVYDIFVPFIIC	0	0	0	0	10	10
CVFDTFLPFVLC	0	1	1	2	18	10
GIFDIHMPFIPC	0	0	0	0	3	10
CYFDIFLPFIYC	0	0	0	12	45	10

CYFDPHFIFIPS	0	0	0	2	11	10
CVYDIFVPYIIC	0	0	0	2	15	10
CVSDSFLPFILC	0	0	0	2	16	10
CVSDSFLPFIFC	0	0	0	0	3	10
CVFDIFVPFIFC	0	0	0	0	4	10
CLFDLHLPFICC	0	0	1	2	32	9
CCFDIFLPFVFC	0	0	1	4	10	9
CCFDSFLPFISC	0	0	0	13	32	9
CDFDIHMPFIPC	0	0	0	0	4	9
CFFDPFFIFIPC	0	0	1	4	6	9
CIFDIFVPFIPC	0	0	0	0	3	9
CLFDLYLPFIFC	0	0	2	15	37	9
CIFDSFFPFICC	0	0	0	3	37	9
CIFDIHMPFTLC	0	0	1	1	6	9
CLCDSFLPFIFC	0	0	0	3	11	9
CIFDIHMTFITC	0	0	0	0	2	9
CIFYTFLPFIFC	0	0	0	0	3	9
CIFDLFLPFICC	0	0	0	1	2	9
CIFDLFVPSIFC	0	0	0	3	13	9
CIFDNRLPFIFC	0	0	0	0	4	9
CVFDLFVPFILC	0	0	0	0	6	9
CIFDSLLPFIFC	0	0	0	4	35	9
CILDTFLPFIIC	0	0	0	0	1	9
CILDIFMPFIPC	0	0	0	0	4	9
CIYDIFVPFIFC	0	0	0	0	8	9
CIYDLFCPFIVC	0	0	0	4	12	9
CLFDIFCPLILC	0	0	0	13	20	9
CLFDIFLPFVLC	0	0	1	2	17	9
CLFDISLPFIIC	0	0	0	5	8	9
CLFDLFCPFISC	0	0	0	19	19	9
CLFDLFYPFILC	0	0	0	2	22	9
CLFDSFCPFIFC	0	0	0	21	26	9
CVFDLWLPFYYC	0	0	2	19	57	9
CLLDLFVPFVFC	0	0	0	9	8	9
CLFDSFFPFILC	0	2	30	164	101	9
CLFDSSVPFIFC	0	0	0	8	23	9
CLFDTFVPFISC	0	0	1	16	23	9
CLFDVFCDFILC	0	0	1	46	62	9
CLFDSFFPFIFC	0	0	1	7	26	9
CLFDVFLPFIFC	0	0	0	6	7	9
CLFGLFCPFIFC	0	0	0	7	7	9
CLYDNLLPFIPC	0	0	0	4	15	9
CTFDSFLPFILC	0	0	0	0	11	9
CSFDSFLPFIFC	0	0	0	6	18	9
CTFDIHMPFILC	0	0	0	0	3	9
CTFDLFIPFVIC	1	0	0	18	43	9
CLFNIFLPFIFC	0	0	1	2	4	9
CTFDLILPFIFC	0	0	0	2	6	9
CVFDLYLPFIFC	0	0	0	3	15	9
CVFDIHMPFITC	0	0	0	0	3	9
CVYDLFLPYIYC	0	0	0	2	7	9
CVYDLFLPFTFC	0	0	0	4	25	9

CVFDSFFPFILC	0	0	0	1	6	9
CVFDIHMPLIPC	0	0	0	1	3	9
CVFDSFLPFFIC	0	0	0	3	7	9
CVFDSHCPFIFC	0	0	0	1	27	9
CVFHSFLPFIIC	0	0	0	0	5	9
CVYDIFVSFIIC	0	0	0	1	15	9
CVYDLFLPFIYY	0	0	0	3	13	9
CVYDIFVPFNIC	0	0	0	2	17	9
CVYDMFLPFIYC	0	0	2	1	10	9
FIFDTFLPFIFC	0	0	1	1	4	9
RLYDSFLPFIFC	0	0	0	3	11	9
WIFDIHMPFIPC	0	0	0	0	3	9
YTFDLFLPFIFC	0	0	0	1	6	9
CLFDPFLPFILC	0	0	1	10	14	9
CVFDLFAPFIFC	0	0	0	2	10	9
CFFDPHLPFIPC	0	0	0	1	8	8
CIFDIFMPSIPC	1	0	0	0	3	8
CCFDILLPFIFC	0	0	0	1	11	8
CCFDSFLPFTFC	0	0	0	16	50	8
CCFDSFPPFIFC	0	0	0	2	16	8
CFFDSFVPFIFC	0	0	0	9	9	8
CLFDLFMPFVFC	0	0	0	2	9	8
CFYDLFIPFIFC	0	0	22	113	74	8
CFYDLFLPLIFC	0	0	1	5	18	8
CCFDIFLPFTFC	0	0	0	5	18	8
CIFDIFIPFVFC	0	0	0	0	2	8
CFYDLFLPFTFC	0	0	3	3	14	8
CVFDSFLRFIIC	0	0	0	1	3	8
CIFDTHMPFTPC	0	0	0	2	4	8
CIFDIHMPFITS	0	0	0	0	1	8
CIFDINMTFIPC	0	0	0	0	3	8
CIFDIHMPFTAC	0	0	0	0	5	8
CIFDTHFPFIVC	0	0	0	0	1	8
CVFDSFLPSIFC	0	0	0	0	4	8
CIFDINMPFIKC	0	0	0	0	3	8
CIFDLFCPFICC	0	0	0	0	12	8
CIFDNHMPIIPC	0	0	0	0	2	8
CIFDPFFPFIPC	0	0	1	27	18	8
CIFDSWLPFIPC	0	0	0	0	1	8
CIFDTFLPFNFC	0	0	0	1	5	8
CIFDTFMPFIIC	0	0	0	0	2	8
CIFDTHMPFIIC	0	0	0	0	0	8
CLFDLFLPFILC	1	0	0	13	20	8
CLFDLFLPFIYC	0	1	0	1	10	8
CIFDTILPFIFC	0	0	0	3	8	8
CIFDTLLPFIIC	0	0	0	0	6	8
CIFDVFMPFIFC	0	0	0	0	2	8
CIFGLFCPFIVC	0	0	0	5	11	8
CILDLFCPFIVC	0	0	1	4	24	8
CILDNWLPFIFC	0	0	0	0	7	8
CLFDHFIDFIFC	0	0	28	114	112	8
CLFDSFFPFIPC	0	0	3	23	23	8

CVYDFFVPFIIC	0	0	0	2	8	8
CVFDSFLTFIFC	0	0	0	0	3	8
CLYDLFCPFIFC	0	0	0	3	7	8
CLFDSFVPFISC	0	0	0	10	30	8
CLFDSFLPLILC	0	0	1	4	18	8
CLFDTFIPFILC	0	0	0	17	23	8
CLFDTFLPFTFC	0	1	1	20	35	8
CLFDTIVPFIFC	0	0	0	3	6	8
CLFDVFLPFIIC	0	0	0	2	17	8
CLFGTFLPFIFC	1	0	1	6	11	8
CLYDIFVPFIIC	0	0	0	0	14	8
CLFDSHLPFIFC	0	2	1	6	25	8
CVFDLFLPFVIC	0	0	0	0	4	8
CNYDIHMPFIPC	0	0	0	0	3	8
CTYDTFLPFIFC	0	0	0	4	14	8
CTFDLFVPFILC	0	0	0	0	10	8
CVFDIFCPFIIC	0	0	0	0	1	8
CTFDLFLPFVLC	0	0	0	8	27	8
CTFDLFLPFINC	0	0	0	1	1	8
CTFDIFLPFVLC	0	0	0	3	27	8
CTFDLFLPYIFC	0	0	1	12	30	8
CVFDLFVPFVFC	0	0	0	1	16	8
CNFDIHMPLIPC	0	0	0	0	1	8
CVYDLYLPFIYC	0	0	1	2	13	8
CVFDNWLPFICC	0	0	0	0	7	8
CVFDVHMPFIPC	0	0	0	3	3	8
CVFGIHMPFIPC	0	0	0	1	0	8
CVFVSFLPFILC	0	0	0	0	9	8
CVFDIFLPFIYC	0	0	0	0	2	8
CVYDLFLPFVYC	0	0	0	6	30	8
CVFDSFLPLVLC	0	0	2	22	27	8
CVFDVFCPFVFC	0	0	0	10	32	8
CYFDIFLPFIPC	0	0	0	1	19	8
CYFDPHFIFIFC	0	0	0	0	10	8
CYFDPHIIFIPC	0	0	0	2	9	8
CYFDPRFIFIPC	0	0	0	5	9	8
RIFDNWLPFIYC	0	0	0	4	8	8
YTFDTFLPFIFC	0	0	0	0	4	8
CLFDLYCDFIPC	0	0	0	1	16	8
CVYDSFLPFIPC	0	0	0	0	8	8
CLFDIFMPFIPC	0	0	0	0	6	7
CAFDLFLPFIYC	0	0	0	1	3	7
CAFDIFLPFILC	0	0	0	6	15	7
CIFATFLPFIFC	0	0	0	0	0	7
CIFDLFCPSIVC	0	0	0	6	10	7
CFFDLHLPFVYC	0	0	3	14	23	7
CIFDIHKPFIPS	0	0	0	0	3	7
CIFDAFVSFIVC	1	0	28	90	77	7
CIFDIFCPFILC	0	0	0	0	5	7
CIFDIFMPFTFC	0	0	0	2	11	7
CIFDTFLPFIMC	0	1	0	0	2	7
CIFDIHKPFNPC	0	0	0	1	3	7
CIFDIHKPYIPC	0	0	0	0	2	7
CIFDIHMNFIPC	0	0	0	0	1	7
CIFDISMPFILC	0	0	1	6	10	7
CIFDISMPFIPC	0	0	0	0	3	7
CIFDLFCPFVFC	0	0	0	0	2	7
CIFDLFDPFIFC	0	0	0	5	14	7
CLFDILCPFILC	0	0	1	10	12	7
CIFDKWLPFIYC	0	0	0	3	20	7
CIFDNCLPFIYC	0	0	0	2	8	7
CLFDIFCPFIRC	0	0	0	9	22	7
CIFDLFSPFIVC	0	0	1	3	19	7
CIFDSFSPFIPC	0	0	0	3	8	7
CIFDSFAPFIYC	0	0	0	3	6	7
CIFDNWLPYIYC	0	0	0	2	10	7
CIFDNWLPSIFC	0	0	0	0	3	7
CIFDNWLPFTFC	0	0	0	1	11	7
CIFDLFVTFIFC	0	0	0	1	2	7
CIFDNWLLFIYC	0	0	0	4	7	7
CLFDLFFPFIVC	0	0	1	7	25	7
CIFDLFSPLIFC	0	0	1	1	8	7
CIFDLFVPFVYC	0	0	0	1	9	7
CIFDSWLPFVFC	0	0	0	0	5	7
CIFDNWLPFITC	0	0	0	0	23	7
CIFDTFLPFIHC	0	0	0	0	3	7
CIYDIFMPFILC	0	0	0	4	8	7
CIFDTFLPLIIC	0	0	0	0	1	7
CIFDTFSPFIPC	0	0	0	0	2	7
CIFDTFVPFIYC	0	0	0	0	7	7
CIFDVFMPFILC	0	0	0	2	20	7
CIFGLFVPFIFC	0	0	0	4	12	7
CIIDNWLPFIYC	0	0	0	2	20	7
CILDIFMPFIFC	0	0	0	0	2	7
CISDIFMPFILC	0	0	0	4	4	7
CIWDSFVPFIMC	0	0	0	8	36	7
CIWDTFVPFILC	0	1	0	0	14	7
CIYDLFVPFIFC	0	0	0	2	4	7
CTFDLFLPFVYC	0	0	0	0	6	7
CIFDLFFPFIFC	0	0	0	1	1	7
CTSDIFLPFILC	0	0	0	7	8	7
CLFGIFLPFIFC	1	0	0	10	10	7
CLFDLHLPFVFC	0	0	2	23	30	7
CLFDPFLSFILC	1	0	1	15	59	7
CLFDSFFPFIIC	0	0	1	3	7	7
CLFDSFPPFIFC	0	0	0	0	1	7
CLFDTFDPFIFC	0	0	0	6	17	7
CLFDTFLHFIFC	0	0	0	0	4	7
CLFDVFCDFIPC	0	0	0	3	26	7
CTFDLFMPFIPC	0	0	0	0	2	7
CLIDIFLPFIFC	0	0	0	3	6	7
CLLDIFCPFILC	0	0	0	7	13	7
CLLDTFVPFIFC	0	0	1	6	25	7
CLSDIFLPFIIC	0	0	0	0	8	7

CLYDSFLPFIFY	0	0	0	5	12	7
CLFDSFDPFIFC	0	0	0	6	25	7
CTFDLFLPFIFF	0	0	0	0	4	7
CTFDLFCPFIFC	0	0	0	1	16	7
CTFDHFLPFIFC	0	0	0	5	17	7
CTFDLFAPFIYC	0	0	0	4	19	7
CNFDLFCPFIVC	0	0	0	0	10	7
FDLFCPFIFC	0	0	0	1	3	7
CLYDYFLPFIFC	0	0	0	1	9	7
CVCDLFLPFIYC	0	0	0	3	15	7
CLFDLHEPFIFC	0	0	0	0	3	7
CPFDLFCPFIFC	0	0	1	13	16	7
CYFDPHFISIPC	0	0	0	8	10	7
CVYDTFVPFIFC	0	0	0	0	16	7
CVFDIFLPFIPC	0	0	0	0	3	7
CTFDSFLPFIYC	0	0	0	0	4	7
CVFDSFLPIIFC	0	0	0	0	1	7
CVFDVFCPLIFC	0	1	0	8	15	7
CVLDVFCPFIFC	0	0	1	6	22	7
CVNDIFVPFIIC	0	0	0	1	7	7
CVYDIFLPFIYC	0	0	0	0	1	7
CVYDIFVPIIIC	0	0	0	1	3	7
CVYDLFLPFIHC	0	0	0	9	25	7
CVYDLFLTFIYC	0	0	0	1	8	7
CVYDLFMPFIPC	0	0	0	0	3	7
CVFDSLLPFICC	0	0	0	4	23	7
CVYGLFLPFIYC	0	0	0	8	26	7
SLYDSFLPFIFC	0	0	0	2	2	7
CTFDTHLPFICC	0	0	2	14	56	7
YLFDSFVPFIFC	0	0	0	0	2	7
FVYDIFVPFIIC	0	0	0	1	6	7
FLYDSFLPFIFC	0	0	0	1	4	7
CYFDPHFVFIPC	0	0	0	5	8	7
CLFDLFCPLIFC	0	0	2	14	33	7
CYFDPHFIFVPC	0	0	0	4	16	7
CIFDIQMPLIPC	0	0	0	0	1	6
CLFDSFLPFIRC	0	0	0	6	31	6
CAFDSFLPFIPC	0	0	0	0	2	6
CCFDIFLPLIFC	0	0	0	7	21	6
CCFDSFLPFIFS	0	0	0	1	12	6
CCFDSYLPFIFC	0	0	1	3	3	6
CCFDTFLPFIYC	0	0	0	0	7	6
CCFGSFLPFIFC	0	0	0	5	10	6
CCFNSFLPFIFC	0	0	0	3	4	6
CFFDLDLPFIFC	0	0	0	9	19	6
CDFDLFLPFILC	0	0	0	0	1	6
CEYDLFLPFIFC	0	0	0	0	6	6
CFFDLFCPFIFC	0	0	3	13	44	6
CFFDLFVPFIFC	0	0	0	4	10	6
CFFDPHLPFVFC	0	0	8	71	67	6
CGFDSFLPFIIC	0	0	0	0	4	6
CIFDIHMPFMPC	0	0	0	1	6	6
CIFDIHMHFIHC	0	0	0	0	2	6
CIFDIHTPFILC	0	0	0	0	5	6
CPFDIFLPFIIC	0	0	0	5	15	6
CLFDSFLPFTLC	0	0	1	6	11	6
CGFDLFLPFIFC	0	0	0	0	5	6
CIFDIFCPFIFC	0	0	0	0	3	6
CVNDLFLPFIYC	0	0	0	3	9	6
CIFDIFIPFIFC	0	0	0	0	2	6
CIFDIFKPFILC	1	0	0	1	10	6
CIFDIFMPFIHC	0	0	1	2	16	6
CIFDIFMPFIPS	0	0	0	0	2	6
CIFDIFMPFIPY	0	0	0	0	2	6
CIFDIFMPFISC	0	0	0	0	4	6
CIFDIFMPFNLC	0	0	0	1	4	6
CIFDIFMPSIFC	0	0	0	0	0	6
CIFDLFLPFVIC	0	0	0	16	25	6
CIFDTFSPFILC	0	0	0	0	6	6
CIFDLFCQFIVC	0	0	0	3	9	6
CIFDLFSPFICC	0	0	0	2	14	6
CIFDLFSPFTFC	0	0	0	4	13	6
CIFDLFVPFIFS	0	0	0	0	4	6
CIFDLFVPFNFC	0	0	0	0	1	6
CIFDLFYPFIPC	0	0	0	8	15	6
CIFDLSVPFIFC	1	0	0	0	23	6
CIFDNWLPFINC	0	0	0	7	17	6
CIFDNWLPFVLC	0	0	0	0	1	6
CIFDLIVPFIFC	0	0	0	0	5	6
CIFDSFVPFVFC	0	0	0	0	7	6
CIFDSFVPFIYC	0	0	0	0	1	6
CIFDTFLPFIFR	0	0	0	1	5	6
CIFDSFFHFIPC	0	0	1	2	3	6
CIFDTFCPFIFC	0	0	0	0	1	6
CIFDSFYPFIPC	0	0	1	4	9	6
CIFDTFLPSILC	0	1	0	1	1	6
CIFDSFIPFVFC	0	0	0	0	10	6
CIFDSFFPLIPC	0	0	3	15	10	6
CIFDSFFPFVLC	0	0	1	18	32	6
CIFDNWLPIIYC	0	0	0	1	9	6
CLFDIFCPFTLC	0	0	0	10	18	6
CIFDIYMPFILC	0	0	0	0	11	6
CIFELFFPFILC	0	0	1	3	22	6
CIFDTFVPFIPC	0	0	0	0	4	6
CIFVTFLPFIFC	0	0	0	0	10	6
CIIDIFMPFILC	0	0	0	1	4	6
CIIDLFVPFIFC	0	0	0	1	4	6
CIRDSFVPFILC	0	0	0	6	15	6
CIWDSFVPLILC	0	0	0	6	28	6
CIYDIFMPFIFC	0	0	0	0	2	6
CLFDIFCPFIVC	0	0	0	1	9	6
CLFDIFCPFVLC	0	0	0	4	17	6
CLFDIFLHFIIC	0	0	0	3	9	6
CLFDIFLPFIFS	0	0	0	2	8	6

CLFDIFLPFIFY	0	0	0	5	8	6
CLFDIFLPSIIC	0	0	0	4	13	6
CLFDIFLPYIIC	0	0	0	1	9	6
CLFDLFCPFIFY	0	0	0	2	5	6
CLFDLHLPLIFC	0	0	2	6	13	6
CLFDLYCDFIIC	0	0	1	6	29	6
CLFDLYCPFIFC	0	0	0	3	8	6
CIFDLCCPFIVC	0	0	0	0	4	6
CLFDPFVPFVFC	0	1	2	24	20	6
CLFDSFVPYIFC	0	0	0	7	9	6
CVFDSFLPFIFS	0	0	0	0	5	6
CVFYSFLPFILC	0	0	0	0	4	6
CSFDIFLPFILC	0	0	0	2	4	6
CVFDLFLSFIFC	0	0	0	2	2	6
CLFDTFIPFIIC	1	0	0	0	4	6
CLFDTFLPSIFC	0	0	1	9	15	6
CLFDTFVLFIFC	0	0	0	6	13	6
CLFDTMLPFIIC	0	0	1	0	10	6
CLNDSFLPFIFC	0	0	0	5	5	6
CLYDLFLPFIPC	0	0	0	1	3	6
CLYDSFLPFITC	0	0	0	1	8	6
CLYDSFLPIIFC	0	0	0	1	9	6
CLYDSFLPYIFC	0	0	0	4	10	6
CLYDSFLSFIFC	1	0	0	2	7	6
CLYYSFLPFIFC	0	0	0	0	9	6
FDLFIPFVLC	2	0	30	166	123	6
FDSFLPFIPC	0	0	0	0	6	6
YDLFLPFIFC	1	0	0	1	11	6
CLYDSFLHFIFC	0	0	0	2	17	6
CVFDLFIPFVLC	0	0	0	7	10	6
CSFDVFLPFIFC	0	0	0	1	6	6
CTFDIFLPFTFC	0	0	0	0	5	6
CTFDIFLPFVFC	0	0	0	0	6	6
CTFDIFLPLILC	0	0	0	9	20	6
CTFDIFVPFIIC	0	0	0	0	1	6
CTFDILLPFIFC	0	0	0	0	5	6
CTFDLFIPFIFC	0	0	0	2	15	6
CTFDLFVPFVFC	0	0	1	0	9	6
CTFDLFLLFIFC	0	0	0	4	8	6
CTFDLFMPFIYC	0	0	0	0	3	6
CTFDLFPPFIFC	0	0	0	13	10	6
CTFDLFVPFIYC	0	0	0	0	8	6
CTFDSWLPFIFC	0	0	0	0	4	6
CTFDTFLSFIFC	0	0	0	3	7	6
CTFDVFLPFVFC	0	0	0	8	15	6
CVFDIFIPFVCC	0	0	0	0	9	6
CLFDSFLPFVLC	0	0	1	5	32	6
CVFDIFVPFILC	0	0	0	0	0	6
CVFDLFLHFIFC	0	0	0	1	5	6
YIFDNWLPFIYC	0	0	0	3	8	6
CVYDIFLPFILC	0	0	0	0	11	6
CVYDIFAPFIFC	0	0	0	0	3	6

CVFDSFWPFIIC	0	0	0	0	5	6
CVFDLHLPFIFC	0	0	0	2	8	6
CVFDLILPFIFC	0	0	0	1	3	6
CVFDLWLPFIFC	0	0	0	1	2	6
CVFDNWLPFILC	0	0	0	0	4	6
CVFDNWLPFIPC	0	0	0	1	7	6
CVFDPFLPFIIC	0	0	0	0	0	6
CVFDSFLPFALC	0	0	1	13	9	6
CVFDSFLTFILC	0	0	0	1	5	6
CVFDSSLPFIFC	0	0	0	0	8	6
CVFDTFIPFIIC	0	0	0	0	1	6
CVFDTFIPFVIC	0	0	0	1	10	6
CVFDVFCDFIFC	0	0	0	4	9	6
CVFDVLCPFIFC	0	0	0	10	30	6
CVFGSFLPFIFC	0	0	0	0	6	6
CVFNSFLPFILC	0	0	0	0	3	6
CVYDIFVPFFIC	0	0	0	1	5	6
CVYDIFMPFIFC	0	0	0	0	1	6
CVFDSFLPFNFC	0	0	0	0	2	6
YIFDIFMPFILC	0	0	0	0	3	6
CVYYIFVPFIIC	1	0	1	0	10	6
CVFDLFVPFIPC	0	0	0	0	11	6
CVFDLFSPFIYC	0	0	0	0	2	6
CVYDIFMPFIPC	0	0	0	0	5	6
CVYDIFVPFIIF	0	0	0	0	5	6
CVYDIFVPFVFC	0	0	0	0	13	6
CVYDIFVPLIFC	0	0	0	0	7	6
CVYDNFVPFIIC	0	0	0	1	10	6
CVYDIFVRFIIC	0	0	0	0	1	6
CYFDLFLPFIFC	0	0	0	0	4	6
CYFDPHFIFIPY	0	0	0	0	9	6
CYFDTFLPFIFC	0	0	0	3	13	6
CYFNPHFIFIPC	0	0	0	2	4	6
FVFDSFLPFILC	0	0	0	1	1	6
RVFDSFLPFIFC	0	0	0	0	1	6
CYFDIFLPFILC	0	0	1	7	28	6
SIFDIFMPFILC	0	0	0	0	9	6
STFDLFLPFIFC	0	0	0	4	8	6
CCFDLFFPFIIC	0	0	0	2	10	5
CFFDLLLPFVYC	0	0	21	83	35	5
CFFDLHLPFVLC	0	0	1	9	19	5
CFFDLFFPFIPC	0	0	0	8	12	5
CCFDSFLTFIFC	0	0	0	0	8	5
CDFDVFCPFIFC	0	0	0	2	14	5
CCFDSFLPFITC	0	0	0	0	2	5
CIFDNHMPFNPC	0	0	0	0	0	5
CFYDPFLPFIFC	0	0	0	6	26	5
CIIDIHMPFITC	0	0	0	0	5	5
CFFDSFLPFILC	0	0	0	1	6	5
CIFDNWFPFIYC	0	0	0	2	13	5
CLFDSFVTFIFC	0	0	0	3	6	5
CAYDLFLPFIFC	0	0	0	1	3	5

CHFDTFVPFIFC	0	0	0	2	5	5
CILDSFVPFIFC	0	0	0	1	3	5
CTFDLFVPFIPC	0	0	0	0	2	5
CLFDSYVPFIFC	0	0	0	2	16	5
CIFDIFIPFIPC	0	0	0	0	5	5
CIFDIFMPLIIC	0	0	0	0	3	5
CIFDNHMPYIPC	0	0	0	0	1	5
CIFDIFMPYILC	0	0	0	1	7	5
CIFDIHMPFIPW	0	0	0	0	1	5
CIFDIHMPLITC	0	0	0	0	0	5
CIFDIHMTFIHC	0	0	0	0	3	5
CIFDIHTPFTPC	0	0	0	0	1	5
CIFDIWLPFIFC	0	0	0	0	4	5
CIFDLFCPFIEC	0	0	0	2	11	5
CIFDLFCPFNVC	0	0	0	1	9	5
CIFDLFVPFIFY	0	0	0	3	7	5
CIFDLFVPFVLC	0	0	0	0	12	5
CIFDLWLPFIFC	0	0	0	0	2	5
CIFDLWLPFILC	0	0	0	0	1	5
CILDLFCPFIIC	0	0	0	0	1	5
CIFNIFMPFILC	0	0	0	1	3	5
CILDTFLPFILC	0	0	0	0	4	5
CIFDNWLPFLCC	0	0	0	0	17	5
CIFYIHMPFITC	0	0	0	0	1	5
CIFDNWLPFNFC	0	0	0	0	0	5
CIFDNWLPLIIC	0	0	0	0	0	5
CIFDNWLQFIYC	0	0	0	1	9	5
CIFDPFLSFIPC	0	0	0	6	15	5
CIFDSFCPFIIC	0	0	0	0	3	5
CIFDSFFPFITC	0	0	1	8	21	5
CIFDSFIPFIPC	0	0	0	2	6	5
CIFDSFLPFIVC	0	0	0	2	2	5
CIFDSFVPFILC	0	0	0	0	6	5
CIFDSFVPFIPC	0	0	0	0	5	5
CIFDNWPPFIFC	0	0	0	0	7	5
CIFDSWLPYILC	0	0	0	3	9	5
CIFDTHLPFIYC	0	0	0	0	3	5
CIFGIFMPFIPC	0	0	0	1	2	5
CIFDTFIPFIFC	0	0	0	0	1	5
CIFELSFPFIPC	0	0	1	8	15	5
CIFDTHMPFIFC	0	0	0	0	5	5
CIFDTYMPFIFC	0	0	0	0	2	5
CIFDTHIPFIPC	0	0	0	0	2	5
CIFDTFMPFICC	0	0	0	0	0	5
CIFDTFLRFIFC	0	0	0	0	1	5
CIFDTFLPFILY	0	0	0	0	2	5
CIFGIFMPFILC	0	0	1	4	8	5
CIFDNWLPFIYY	0	0	0	4	20	5
CIFDSLFPFIFC	0	0	1	3	4	5
CINDIHKPFIPC	0	0	0	0	2	5
CIIDIHMPFIPS	0	0	0	0	0	5
CINDIHMPFIPC	0	0	0	0	2	5

CIWDSFLPFILC	0	0	0	0	6	5
CIYDIHMPFIPS	0	0	0	0	3	5
CIYDIHMPLIPC	0	0	0	0	2	5
CIYDIHMPYIPC	0	0	0	1	0	5
CIYDTFLPFILC	0	0	0	0	2	5
CLFDIFCPFIHC	0	0	1	3	10	5
CLFDIFLPFNFC	0	0	0	2	2	5
CLFDIFLPIIFC	0	0	0	1	8	5
CLFDIFLPIIIC	0	0	0	2	2	5
CLFDIFLTFIFC	0	0	0	2	3	5
CLFDIFWPFIPC	0	0	0	0	0	5
CLFDLFYPFIVC	0	0	0	6	10	5
CLFDLHMPFIFC	0	0	0	1	7	5
CLFDLYLPFILC	0	1	24	57	53	5
CLFDLYVPFVFC	0	0	0	1	6	5
CLFDPFLPFIPC	0	0	1	28	23	5
CLFDSFIPFIIC	0	0	0	0	3	5
CLFDSFLPFITC	0	0	0	0	11	5
CLFDSFLPFNLC	0	0	0	2	8	5
CLFDSFPPFILC	0	0	1	9	11	5
CIFDNWLPFIMC	0	0	0	0	2	5
CLFDSFVPFIVC	0	0	0	5	19	5
CIIDTFLPFIIC	0	0	0	0	0	5
CLFDLSLPFIPC	0	2	25	145	55	5
CLFDTFLPFNFC	0	0	0	0	6	5
CCYDSFLPFIFC	0	0	0	1	10	5
CVLDSFLPFIPC	0	0	0	0	4	5
CTFDIFLPFIRC	0	0	1	7	23	5
CVFDLFLPFIFY	0	0	0	2	17	5
CLFDTFVPSIFC	0	0	0	9	14	5
CLFDTSVPFIFC	0	0	0	14	23	5
CLFDVFCPFILC	0	0	1	7	12	5
CLFEIFLPFIFC	0	0	0	2	6	5
CLFNTFLPFIFC	0	0	0	3	7	5
CLLDSFLPFILC	0	0	0	11	22	5
CLLDTFLPFIFC	0	0	0	13	24	5
CLSDIFLPFIFC	0	0	1	8	12	5
CLSDSFLPFIFC	0	0	0	0	4	5
CLSDTFLPFIFC	0	0	0	8	12	5
CLWDSFVPFIFC	0	0	0	0	0	5
CLYDSFLPFIFR	0	0	0	1	8	5
CLYDSLLPFIIC	0	0	0	1	8	5
CLYESFLPFIFC	0	0	1	2	6	5
CLYNSFLPFIFC	0	1	0	7	11	5
FDLFLPFIFC	0	0	2	23	21	5
FDSFLPFILC	0	0	0	3	6	5
FDSFLPFVLC	2	0	1	4	7	5
CNFDIFMPFILC	0	0	0	0	6	5
CNFDIHKPFIPC	0	0	0	0	1	5
CNFDIHMPFITC	0	0	0	0	1	5
CNFDIHMPFNPC	0	0	0	0	5	5
CNFDTFLPFILC	0	0	0	0	1	5

CLFGSFLPFIFC	0	0	0	0	2	5
CVFDLFLPFIFS	0	0	0	2	12	5
CVFDIFVPFVLC	1	0	0	3	14	5
CVLDLFLPFTFC	0	0	0	3	4	5
CTFDLFLRFIFC	0	0	0	0	4	5
CTFDTFLPFIIS	0	0	0	0	2	5
CTFDSFFPFIPC	0	0	1	4	11	5
CTFDSFFPFIFC	0	0	0	2	4	5
CTFDNWLPFIIC	0	0	0	0	3	5
CTFDIFLPFIVC	0	1	0	0	5	5
CTFDLFLHFIFC	0	0	0	2	17	5
CTFDLFLPLIIC	0	0	0	0	0	5
CTFDLFLPIIFC	0	0	0	8	5	5
CTFDLFLPFIMC	0	0	0	0	8	5
CTFDIFVPFILC	0	0	0	0	5	5
CTFDTHCPFIFC	0	0	0	1	9	5
CTFDTFLPFNFC	0	0	0	0	7	5
CVFDIFIPFVRC	0	0	0	7	21	5
CTFDTYLPFIFC	0	0	0	1	9	5
CTFDVFPPFIFC	0	0	0	4	11	5
CTFNTFLPFIFC	0	0	0	1	12	5
CTFYTFLPFIFC	0	0	0	0	4	5
CTIDTFLPFIFC	0	0	0	2	12	5
CVCDSFLPFIIC	0	0	0	0	0	5
CVFDGFLPFIFC	0	0	0	1	6	5
CVFDIFIPFIYC	0	0	0	0	2	5
CVFDLFLLFIFC	0	0	0	0	4	5
CVFDIFIPLVLC	0	0	1	5	11	5
CPFDLFLPFIFC	0	0	0	0	4	5
CTFDVFLPSIFC	0	0	0	4	4	5
RIFDLFCPFIVC	0	0	0	0	5	5
CVYDIFVTFIIC	0	0	0	3	18	5
CVYDIFVPFIIY	0	0	0	1	17	5
CVFDLFVPYILC	0	0	0	12	17	5
CVFDSCLPFIIC	0	0	0	0	6	5
CVFDSFLLFIFC	0	0	0	0	1	5
CVFDSFLPFIIF	0	0	0	1	2	5
CVFDSFLPFNLC	0	0	0	1	12	5
CVFDSFLPFSIC	0	0	0	0	0	5
CVFDSFLPIILC	0	0	0	1	4	5
CVFDSFLPVIIC	0	0	0	0	3	5
CVFDSFLQFILC	0	0	0	0	8	5
CVFDSFLTLIIC	0	0	0	0	2	5
CVFDSFVPFIPC	0	0	0	0	0	5
CVFDSFVPFVFC	0	0	0	1	9	5
CVFDSILPFILC	0	0	0	2	3	5
CVFDSLLPLIIC	0	0	0	0	5	5
CVFDSSLPFVLC	0	0	0	4	11	5
CVFDSYLPFILC	0	0	0	1	4	5
CVFDSYLPFVLC	0	0	0	2	4	5
CVFDTFIPFVFC	0	0	0	2	7	5
CVFDTHFPFILC	0	0	0	3	4	5

CVFDVFCPFTFC	0	0	1	8	24	5
CVFELFLPFIFC	0	0	0	0	3	5
CVFELYTPFIFC	0	0	0	2	13	5
CVFDLFLPLILC	0	0	0	0	1	5
CVFNLFLPFIFC	0	0	0	1	5	5
CVFDLFMPFILC	0	0	0	1	9	5
CVFDSWLPFIPC	0	0	0	0	3	5
CVYDLFLPFINC	0	0	0	1	16	5
CYFGPHFIFIPC	0	0	0	3	12	5
CVYDLFLPFIVC	0	0	0	1	11	5
CVYDIIVPFIFC	0	0	0	1	0	5
YVFDSFLPFILC	0	0	0	2	3	5
CVYDLFLPFVLC	0	0	0	1	13	5
CVYDLFSPFIFC	0	0	0	0	9	5
CVYDLILPFIYC	0	0	0	2	8	5
CYFAPHFIFIPC	0	0	0	1	4	5
CYFDHHFIFIPC	0	0	0	1	5	5
CYFDPHFIIIPC	0	0	0	1	8	5
CVYEIFVPFIIC	0	0	0	2	10	5
CYFVPHFIFIPC	0	0	1	5	3	5
SVYDIFVPFIIC	0	0	0	0	6	5
SYFDPHFIFIPC	0	0	0	2	5	5
RYFDPHFIFIPC	0	0	0	0	6	5
RVFDLFLPFIFC	0	0	0	0	6	5
RVYDLFLPFIYC	0	0	0	1	7	5
CLFDTFCPFIFC	0	0	0	1	5	5
GVFDSFLPFIIC	0	0	0	0	5	5
FTFDLFLPFIFC	0	0	0	3	10	5
SLFDIFLPFIFC	0	0	0	1	6	5
CLFDISLPFIYC	0	0	0	0	0	4
CCFDSFFPFIFC	0	0	0	2	3	4
CAFDIFIPFVLC	0	0	1	2	11	4
CEFDSFLPFVLC	0	0	0	1	10	4
CAFDTHFPFITC	0	0	0	4	10	4
CCFDLFLPFIFC	0	0	0	0	10	4
CCFDSFLPLVIC	0	0	0	3	5	4
CCFDSFLPFIFY	0	0	0	4	8	4
CCFDSFLPFLCC	0	0	0	0	7	4
CCFDSFLPFNFC	0	0	0	1	7	4
CCFDSFLSFIFC	0	0	0	4	8	4
CCFDSFQPFIFC	0	0	0	4	6	4
CCFYSFLPFIFC	0	0	0	0	3	4
CCIDSFLPFIFC	0	0	0	2	5	4
CCLDIFLPFIFC	0	0	0	2	5	4
CILDIHMPFILC	0	0	0	0	7	4
CEFDSFLPFIPC	0	0	0	0	1	4
CILDIHMPFTPC	0	0	0	0	2	4
CILDLFCPFIFC	0	0	0	0	2	4
CIFETFLPFILC	0	0	0	0	0	4
CIFDIHMTIIPC	0	0	0	0	0	4
CDYDIFVPFIFC	0	0	0	0	0	4
CIFDIHMPLILC	0	0	0	0	4	4

CFFDIFMPFILC	0	0	0	3	6	4
CFFDIHMPFITC	0	0	0	0	0	4
CFFDLFCPFIVC	0	0	0	2	10	4
CFFDLFLPFIPC	0	0	0	0	6	4
CFFDLFSPFIFC	0	0	0	0	1	4
CFFDSFFPFIPC	0	0	22	66	40	4
CFFDSLWPFAVC	0	0	26	124	84	4
CVFDLFLTFIFC	0	0	0	1	10	4
CHFDIFLPFIFC	0	0	0	4	11	4
CFYDLLLPFIFC	0	0	0	12	17	4
CIFDIHMPFVIC	0	0	0	0	3	4
CHFDLFCPFIFC	0	0	0	3	2	4
CHFDSFVPFIFC	0	0	0	8	11	4
CIFDIHMPSILC	0	0	0	1	3	4
CLFDIYLPFIFC	0	0	0	2	4	4
CLFDLFCHFIFC	0	0	1	4	7	4
CVFDLFLPFVYC	1	0	0	0	3	4
CVFDLFLPIIFC	0	0	0	0	7	4
CIFYIHMTFIPC	0	0	0	1	2	4
CIFDIHMPFNPS	0	0	0	0	2	4
CIFDIHMPFNLC	0	0	0	0	0	4
CIFAIHMPFIPC	1	0	0	1	1	4
CIFDAFLPFIYC	0	0	0	0	1	4
CIFDIFMPFILS	0	0	0	0	8	4
CIFDIFMPFIMC	0	0	0	1	3	4
CIFDIFMPFVPC	0	0	0	0	4	4
CIFDIFMPIILC	0	0	0	0	1	4
CIFDIFMPLIFC	0	0	0	0	3	4
CIFDIFMTFILC	0	0	0	2	4	4
CIFDIFTPFIPC	0	0	0	1	6	4
CIFDIHKPLIPC	0	0	0	0	1	4
CIFDIHLPFIFC	0	0	0	1	4	4
CIFDIHLPFIYC	0	0	0	0	2	4
CIFDIHMHFITC	0	0	0	0	5	4
CIFDIHMHFTPC	0	0	0	0	0	4
CIFDIHMPFNHC	0	0	0	0	1	4
CIIDTFLPFIYC	0	0	0	1	0	4
CIFDINMHFIPC	0	0	0	0	2	4
CIFNLFVPFIFC	0	0	0	1	3	4
CIFDSFMPFILC	0	0	0	0	4	4
CIFDLFCPFVIC	0	0	0	1	8	4
CIFDNFMPFILC	0	0	0	0	2	4
CIFDILMPFIFC	0	0	0	1	2	4
CIFDTFLPLIPC	0	0	0	0	2	4
CIFDINKPFITC	0	0	0	0	0	4
CIFDINMPFNPC	0	0	0	0	0	4
CIFDLFCLFIVC	0	0	0	2	17	4
CIFDLFCPFIVY	0	0	0	1	10	4
CIFDLFCPFVLC	0	0	0	0	3	4
CIFDLFCPFVVC	0	0	0	8	19	4
CIFDLFCPIIVC	0	0	0	2	4	4
CIFDLFCPLIFC	0	0	0	0	1	4
CIFDLFFPFTPC	0	0	0	1	10	4
CIFDLFLPFVCC	0	0	0	0	4	4
CIFDLFRPFIFC	0	0	0	0	1	4
CIFDLFVPFILS	0	0	0	0	0	4
CIFDLFVPLIIC	0	0	0	0	1	4
CIFDNWIPFIYC	0	0	0	3	7	4
CIFDTFLPFIYS	0	0	0	0	4	4
CIFDLSCPFIVC	0	0	1	4	13	4
CIFDTFLPFSFC	0	0	0	0	2	4
CIFDNWLPFVIC	0	0	0	0	0	4
CIFDSFIPFVIC	0	0	0	0	0	4
CIFDNWLSFIYC	0	0	1	1	8	4
CIFDNWPPFIPC	0	0	0	1	2	4
CIFDPFSPFIFC	0	0	0	7	6	4
CIFDPFVPFIIC	0	0	0	0	1	4
CIFDNWLLFIFC	0	0	0	0	2	4
CIFDSFFPFVVC	0	0	0	0	10	4
CIFDSFFPLIFC	0	0	2	4	13	4
CIFDTFLPCIFC	0	0	0	0	1	4
CIFDSFMPFIPC	0	0	0	0	1	4
CIFDSLFPFIPC	0	0	1	7	10	4
CIFDSSFPFIFC	0	0	0	3	5	4
CIFDSSFPFIPC	0	0	2	7	10	4
CIFDTFLPFIIS	0	0	0	0	1	4
CIFDTFLPFINC	0	0	0	0	1	4
CIFDTFLPFIRC	0	0	0	0	3	4
CIFDLFVPYIFC	0	0	0	3	10	4
CIFDMFVPFIIC	0	0	0	0	0	4
CIFHTFLPFIFC	0	0	0	0	1	4
CLFDIFVPFIPC	0	0	0	0	1	4
CIFDIHMTLIPC	0	0	0	0	2	4
CISDNWLPFIFC	0	0	0	0	5	4
CILDTFLPFIYC	0	0	0	0	1	4
CIFDTHMPLIPC	0	0	0	0	0	4
CIFDTHTPFIPC	0	0	0	1	1	4
CIFDTILPFIIC	0	0	0	0	2	4
CIFDTLLPFILC	0	0	0	0	11	4
CIFDTLLPFIYC	0	0	0	1	5	4
CIFDTSLPFIIC	0	0	0	0	4	4
CIFDTSLPFIPC	0	0	0	0	1	4
CIFDTYLPFIIC	0	0	0	0	2	4
CIFDVFLPFIYC	0	0	0	0	3	4
CIFDTFSPFIYC	0	0	0	0	3	4
CIFELFFPFTPC	0	0	1	7	10	4
CIFGNWLPFIFC	0	0	0	0	3	4
CIFGTFLPFIIC	0	0	0	0	0	4
CLFDILCPFIFC	0	0	0	0	3	4
CLFDIFLSFIIC	0	0	0	1	1	4
CIFDNLLPFIYC	0	0	0	1	9	4
CLFDIFLPFIIY	0	0	0	1	6	4
CIFDTFLPLIYC	0	0	0	0	1	4
CLFDFFCPFIFC	0	0	0	4	3	4

CIFDTHMPFIPY	0	0	0	0	2	4
CISDSFFPFIFC	0	0	0	2	2	4
CIWDPFVPFIFC	0	0	0	0	4	4
CIYDIHMPFNPC	0	0	0	0	1	4
CIYDIHMTFIPC	0	0	0	0	0	4
CIYDIQMPFIPC	0	0	0	0	0	4
CIYDNWLPFILC	0	0	0	0	1	4
CIYDSFLPFILC	0	0	0	1	0	4
CIYDTYLPFIFC	0	0	0	0	1	4
CLFDIFCPFICC	0	0	0	0	6	4
CLFDIFFPFIIC	0	0	0	4	11	4
CLFDIFFPFVLC	0	1	20	60	44	4
CLFDIFLHFIFC	0	0	0	4	8	4
CLFDIFLPFIHC	0	0	0	2	2	4
CLFDIFLPFIIS	0	0	0	0	9	4
CVFDLFLPLIYC	0	0	0	1	2	4
CYFDPNFIFIPC	0	0	0	2	3	4
CVFDLWLPFTFC	0	0	0	0	5	4
CLFDLFEPFVFC	0	0	0	5	15	4
CLFDLFVPFAFC	0	0	2	9	15	4
CLFDLSCPFIFC	1	0	1	19	18	4
CLFDPFLSFITC	0	0	1	11	6	4
CLFDSFHPFILC	0	0	0	7	7	4
CLSDLFVPFVFC	0	0	0	7	6	4
CLFDSFIPFILC	0	0	0	0	4	4
CLFDSFLPFTFC	0	0	0	2	7	4
CLFDSFMPFIIC	0	0	0	0	3	4
CLFDSFVPFIFS	0	0	0	3	10	4
CLFDSFVPFITC	0	0	0	1	5	4
CLFDSFVPFNFC	0	0	0	0	5	4
CLFDSFVPFVSC	0	0	1	12	18	4
CLFDSFVSFIFC	0	0	0	4	2	4
CLFDSILPFILC	0	0	0	0	2	4
CLFDSLLPFIPC	0	0	0	2	20	4
CLFDSFIPFIPC	0	0	0	0	0	4
CLFDLFFPFITC	0	0	3	8	11	4
CLFDLFCTFIFC	0	0	0	1	1	4
CLFDLFCPFIFS	0	0	0	1	8	4
CLFDTFLPLIIC	0	0	0	1	1	4
CLFDTFLPYIFC	0	0	0	0	4	4
CLFDTFVPFLCC	0	0	0	0	5	4
CLFDTFVQFIFC	0	0	0	1	9	4
CLFDVFCDLIFC	0	0	0	15	6	4
CLFDVFCPFIFC	0	0	0	0	10	4
CLFDYFVPFIFC	0	0	0	2	6	4
CLFGTFVPFIFC	0	0	0	7	13	4
CLFNSFVPFIFC	0	0	0	4	4	4
CLIDIFLPFIIC	0	0	0	0	4	4
CLLDSFLPFIFC	0	0	0	0	1	4
CLFDTFCPFILC	0	0	0	6	24	4
CLFDLFAPFVFC	0	0	0	9	19	4
CLFDSWLPFIFC	0	0	0	0	2	4

CLFDSSLPFIFC	0	0	0	3	6	4
CTFDSLLPFIIC	0	0	0	1	11	4
CLFDSFVPLVFC	0	0	5	10	11	4
CSFDSFLPFILC	0	0	0	6	12	4
CLFDTFLPFIFY	0	0	0	4	2	4
CTFDIFLPFTLC	0	0	1	5	10	4
CLYDNLLPFIFC	0	0	2	5	36	4
CLYDSFFPFIFC	0	0	0	3	6	4
CLYDSFLAFIFC	0	0	0	0	1	4
CLYDFFLPFIFC	0	1	24	52	35	4
CLYDSILPFIFC	0	0	0	3	14	4
CLYDSVLPFIFC	0	0	0	0	2	4
CLYDTFVPFIFC	0	0	0	2	9	4
CNFDIHMPFIPS	0	0	0	0	0	4
CNFDINMPFITC	0	0	0	0	0	4
CLYDSFLPFNFC	0	0	1	3	8	4
CNFDLFVPFIFC	0	0	0	3	7	4
CVFDLWLPFVFC	0	0	0	0	8	4
CPFDIFLPFILC	0	0	0	0	6	4
CTFDISLPFILC	0	0	0	4	9	4
CTFDLYLPFIYC	0	0	0	1	1	4
CTFALFLPFIFC	0	0	0	0	2	4
CTFDFFLPFIFC	1	0	0	5	15	4
CSFDLFLPFIYC	0	0	0	0	3	4
CTFDIFLHFIFC	0	0	0	0	2	4
CTFDLFCPFIIC	0	0	0	0	1	4
CTFDLFIPFIIC	0	0	0	0	4	4
CTFDLFIPFILC	0	0	0	2	22	4
CTFDLFLPFIFR	0	0	0	0	9	4
CTFDLFLPFIHC	0	0	0	1	6	4
CTFDLFMPFILC	0	0	0	0	7	4
CTFDLFQPFIFC	0	0	1	3	8	4
CLFDTFLPIIFC	0	0	0	1	5	4
CLFDTFLPFIFS	0	0	0	1	4	4
CTFDTFMPFIFC	0	0	0	1	6	4
CTFETFLPFIFC	0	0	0	0	4	4
CTFVLFLPFIFC	0	0	0	0	5	4
CTLDLFLPFIPC	0	0	0	0	1	4
CTSDLFLPFIIC	0	0	0	0	2	4
CTSDVFLPFIFC	0	0	1	0	9	4
CTVDTFLPFIFC	0	0	0	0	2	4
CVFDAFCPFIIC	0	0	0	0	1	4
CVFDIFVPFVFC	0	0	0	0	0	4
CVFDIHMPFVPC	0	0	0	0	3	4
CVFDIHVPFIPC	0	0	0	0	1	4
CVFDLFCPFVIC	0	0	0	3	15	4
CVFDLFFPFVPC	0	0	0	2	6	4
CVFDLFLPFIFR	0	0	0	1	5	4
CVFDLFLPFITC	0	0	0	1	7	4
CVFDTFLPFICC	0	0	0	0	5	4
CVFDVFCPFISC	0	0	1	4	17	4
CTFDVSLPFIFC	0	0	1	7	12	4

CTFDTFLTFIIC	0	0	0	0	0	4
CYFDPHFIFNPC	0	0	0	2	8	4
CTFDTFLPLIIC	0	0	0	0	4	4
CLFDTFIPC	0	0	0	0	3	4
CVFDLYLPFIPC	0	0	1	0	4	4
CVFDRFLPFIFC	0	0	0	0	2	4
CVFDSFCPFILC	0	0	0	0	2	4
CVFDSFLNFIIC	0	0	0	0	3	4
CVFDSFLPFIQC	0	0	0	2	9	4
CVFDSFLPLIPC	0	0	0	0	4	4
CVFDSFLPSVLC	0	0	0	10	12	4
CVFDSFLPYILC	0	1	0	2	17	4
CVFDSFSPFVLC	0	0	0	2	12	4
CVFDSFVPFILC	0	0	0	0	3	4
CVFDSILPFVLC	0	0	0	1	4	4
CVFDSLLPFVLC	0	0	0	10	18	4
CVFDLFLPFTPC	0	0	0	10	11	4
CVFDTFIPFIPC	0	0	0	0	3	4
CVFDLFLPFLCC	0	0	0	0	9	4
CVFDLFLPFIRC	0	0	0	0	1	4
CVFDVFLPFIFC	0	0	0	0	5	4
CVFDSFSPFIPC	0	0	0	0	8	4
CYFDPQFIFIPC	0	0	0	4	4	4
CVIDLFLPFIFC	0	0	0	1	2	4
CVLDIHMPFIPC	0	0	0	2	2	4
CVLDSFLPFVLC	0	0	0	11	16	4
CTFDTFLPFIFY	0	0	0	2	11	4
CVYDIFIPFVLC	0	0	0	2	5	4
CVYDIFVPFTFC	0	0	0	0	8	4
CVYDIFVPLILC	0	0	0	0	3	4
CVYDILVPFIFC	0	0	0	0	3	4
CVYDIYVPFIIC	0	0	0	3	11	4
CVYDLFLHFIYC	0	0	0	0	8	4
CVYDLFSPFIPC	0	0	0	0	2	4
CVYDTFVPFILC	0	0	0	0	4	4
CVWDSFVPFIIC	0	0	0	0	3	4
CTFDTFLHFIFC	0	1	0	1	5	4
CVYDIFVPFIFF	0	0	0	0	1	4
CTFDSFVPFIFC	0	0	0	1	4	4
CYIDPHFIFIPC	0	0	1	3	2	4
CLFDTFHPFIFC	0	0	0	1	9	4
CVIDSFLPFILC	0	0	0	0	7	4
CVYNLFLPFIYC	0	0	0	0	12	4
CVFYSFLPFIFC	0	0	0	0	1	4
YCFDSFLPFIFC	0	0	0	1	4	4
CVFVLFLPFIFC	0	0	0	1	2	4
FIFDNWLPFIYC	0	0	0	2	11	4
FLFDIFLPFIIC	0	0	0	0	2	4
SCFDSFLPFIFC	0	0	0	0	4	4
SIFDNWLPFIYC	0	0	0	1	4	4
SVFDLFLPFIFC	0	0	0	0	6	4
RLFDTFVPFIFC	0	0	2	0	5	4
CVFESFLPFILC	0	0	0	1	2	4
YVYDLFLPFIYC	0	0	0	1	6	4
CYFDPHFIFISC	0	0	0	1	12	4
CVYYLFLPFIYC	0	0	0	0	8	4
CVFDYFLPFILC	0	0	0	0	3	4
YLYDSFLPFIFC	0	0	0	1	2	4
CCFDISLPFIFC	0	0	1	2	3	3
CAFDTFLPFILC	0	0	0	1	2	3
CAFDTFLPFIYC	0	0	0	0	3	3
CCFDIFLPFIVC	0	0	0	0	5	3
CCFDSFLPFAIC	0	0	0	4	2	3
CCFDIFLPIIFC	0	0	0	1	1	3
CFFDSLLPFIFC	0	0	0	0	2	3
CCFDNWLPFIYC	0	0	0	0	1	3
CCFDPFLPFIIC	0	0	0	0	2	3
CCFDSFLAFIFC	0	0	0	0	2	3
CCFDSFLHFIFC	0	0	0	0	5	3
CAFDLFLPFIPC	0	0	0	0	0	3
CAFDSWLPFVYC	0	0	0	4	5	3
CDFDLFLPFNFC	0	0	0	0	0	3
CAFDLFLPFIIC	0	0	0	0	0	3
CLFDPFFPFIPC	0	0	1	24	15	3
CAYDIFVPFIFC	0	0	0	0	3	3
CCFDSFLPFIFF	0	0	0	3	2	3
CCFDSFLPFVLC	0	0	0	5	18	3
CCFDSFLPFVSC	0	0	0	9	5	3
CDFDSFLPFIFC	0	0	0	0	0	3
CCFVSFLPFIFC	0	0	0	3	7	3
CCFDYFLPFIFC	0	0	0	2	8	3
CCFDTFLPFILC	0	0	0	1	1	3
CCFDSFVPFIFC	0	0	0	0	4	3
CCFDSFSPFIIC	0	0	0	0	0	3
CCFDSFLPLILC	0	0	0	0	4	3
CCFDSFLPFVTC	0	0	0	3	7	3
CIYDIFMPFIPC	0	0	0	0	4	3
CIWDSSVPFILC	0	0	0	5	9	3
CIWDSFVPLIFC	0	0	0	0	5	3
CIWDSFVPFTLC	0	0	2	5	4	3
CIWDSFMPFIFC	0	0	0	1	4	3
CIWDSFLPFIPC	0	0	0	0	1	3
CIIDIHKPFIPC	0	0	0	0	6	3
CIFDLFFPFVLC	0	0	0	3	9	3
CIFDLFFPFILC	0	0	0	0	3	3
CFFDLHLPFVCC	0	0	0	4	18	3
CFFDLFVPFIIC	0	0	0	0	0	3
CFFDLFLPFILC	0	0	6	4	8	3
CFFDLSLPFIFC	0	0	1	2	10	3
CEFDSFLPLIIC	0	0	0	0	1	3
CEFDSFLPFINC	0	0	0	0	1	3
CEFDSFLPFILC	0	0	0	0	3	3
CDYDIFVPFIPC	0	0	0	0	0	3
CFFDNWLPFIYC	0	0	0	7	16	3

CFFDSFCPFIFC	0	1	18	115	64	3
CFFDPSFFFIPC	0	0	0	1	0	3
CFYDIFVPFIFC	0	0	0	0	2	3
CIFDFFCPFIVC	0	0	0	0	1	3
CIFDLFFPFIVC	0	0	0	4	3	3
CFYDSLLPFIFC	0	0	8	42	29	3
CHYDSFLPFIIC	0	0	0	0	1	3
CFYDLFLPFIFY	0	0	0	1	7	3
CLFDPHEPFIYC	0	0	0	0	0	3
CVYDLFLPFMYC	0	0	0	0	0	3
CLFDSFDPFIIC	0	0	0	0	1	3
CVFDTFIPFIFC	0	0	0	0	1	3
CVFDSYLPYIIC	0	0	0	0	0	3
CVFDSWLPFIVC	0	0	0	0	0	3
CVFDSRLPFVYC	0	0	1	4	7	3
CIYDNWLPFICC	0	0	0	2	2	3
CIFDIHMPFTHC	0	0	0	0	1	3
CIFDIHMPIIHC	0	0	0	0	0	3
CIFDIHMPLIHC	0	0	0	0	0	3
CIFDIHMPLTPC	0	0	0	0	3	3
CIFDIHLPFILC	0	0	0	1	0	3
CIFDIHLPFIIC	0	0	0	0	3	3
CIFDIHKPFILC	0	0	0	0	3	3
CIFDIHKNFIPC	0	0	0	0	0	3
CIFDIHITFIPC	0	0	0	1	0	3
CIFDIFMTFIPC	0	0	0	1	2	3
CIFDIHMHFILC	0	0	0	0	0	3
CIFDIFMPFVIC	0	0	0	0	1	3
CIFDIFMPFIFY	0	0	0	0	0	3
CIFDIFMHFILC	0	0	0	3	4	3
CIFDIFLPFVFC	0	0	0	0	0	3
CIFDIFKPFIPC	0	0	0	1	1	3
CIFDIFIPFIIC	0	0	0	0	0	3
CIFDFFLPFVFC	0	0	16	39	31	3
CIFDIFMPFNFC	0	0	0	0	0	3
CIFDIHMHIIPC	0	0	0	0	0	3
CIFDIHMPFTFC	0	0	0	1	8	3
CIFDLFCPFTFC	0	0	1	7	4	3
CIFDLFCPFISC	0	0	0	0	1	3
CIFDLFCPFIGC	0	0	0	2	2	3
CIFDLFAPFIYY	0	0	0	1	4	3
CIFDIQIPLIPC	0	0	0	0	1	3
CIFDINMTLIPC	0	0	0	0	1	3
CIFDINMPFIIC	0	0	0	0	0	3
CIFDIIMPFILC	0	0	0	0	6	3
CIFDIHVPFILC	0	0	0	0	2	3
CIFDIHMTFNPC	0	0	0	0	0	3
CIFDIHMTFIPS	0	0	0	0	0	3
CIYDLFLPFIYC	0	0	0	0	2	3
CIFDMFVPFIFC	0	0	0	0	5	3
CIYDSFLPFIIC	0	0	0	0	2	3
CIFDLFLPFVVC	0	0	1	2	2	3

CIFDSYFPFIPC	0	0	0	1	3	3
CIFDLYVPFIFC	0	0	0	0	7	3
CIFDLWLPFTLC	0	0	0	0	2	3
CIFDLWLPFIIC	0	0	0	0	0	3
CIFDLLCPFIFC	0	0	0	0	1	3
CIFDLICPFIVC	0	0	0	3	2	3
CIFDLHLPFVFC	0	0	0	3	4	3
CIFDLHLPFIFC	0	0	0	0	4	3
CIFDLFYPFILC	0	0	0	0	2	3
CIFDLFVPIIFC	0	0	0	1	7	3
CIFDLFVPFITC	0	0	0	0	2	3
CIFDLFRPFIVC	0	0	0	7	14	3
CIFYNWLPFIYC	0	1	0	2	4	3
CIFDLFLPLIFC	0	0	0	0	2	3
CIFDLFIPFIYC	0	0	0	0	0	3
CIFELFVPFIFC	0	0	0	2	5	3
CVFDSLLPFVFC	0	0	0	4	6	3
CIFDTCLPFIFC	1	0	0	0	5	3
CIFDNHMTFIPC	0	0	0	0	1	3
CIFDSWLPYIIC	0	0	0	0	3	3
CIFDSWLPFICC	0	0	0	2	2	3
CIFDSFLPSIFC	0	0	0	0	0	3
CIFDSFLPFICC	0	0	0	0	5	3
CIFDSFFPSIPC	0	0	1	5	7	3
CIFDSFFPFVPC	0	0	0	5	12	3
CIFDSFFPFTPC	1	0	1	20	19	3
CIFDSFFPFNPC	0	0	0	1	2	3
CIFDSFFPFISC	0	0	0	6	15	3
CIFDRFFPFIFC	0	0	0	0	3	3
CIFDPFFSFIPC	0	0	0	3	6	3
CIFYLFVPFIFC	0	0	0	0	5	3
CIYDTFLPFIYC	0	0	0	0	0	3
CIFDTFLPFVYC	0	0	0	0	3	3
CIFDTHLPFILC	0	0	0	0	0	3
CIFDTHKPFIPC	0	0	0	0	0	3
CIFDTHFPFTLC	0	0	0	0	2	3
CIFDTHFPFIMC	0	0	0	0	0	3
CIFDTFWPFIFC	0	0	0	0	0	3
CIFDTFLTFIYC	0	0	0	0	1	3
CIFYINMTFITC	0	0	0	0	0	3
CIFDTFLPYILC	0	0	0	0	4	3
CIFDTHMPFIYC	0	0	0	0	0	3
CIFDTFLPFTYC	0	0	0	0	5	3
CIFDTFLPFTIC	0	0	0	0	1	3
CIFDTFLPFIFW	0	0	0	0	0	3
CIFDTFLNFIIC	0	0	0	0	0	3
CIFDTFLSFIPC	0	0	0	0	0	3
CIFDTVLPFIFC	0	0	0	1	2	3
CIFDTSLPFIYC	0	0	0	0	1	3
CIFDTILPFILC	0	0	0	0	0	3
CIFDTFLAFIFC	0	0	0	0	4	3
CIFYINMPFIPC	0	0	0	0	2	3

CIFYINMHFITC	0	0	0	0	0	3
CIFVLFCPFILC	0	0	0	0	0	3
CIFENWLPFIFC	0	0	0	0	1	3
CIFELYFPFIPC	0	0	0	4	5	3
CIFDTHMPSIPC	0	0	0	0	0	3
CIFELNLPFILC	0	0	0	6	19	3
CIFYLFCPFIVC	0	0	0	0	1	3
CIFEIHMPFITC	0	0	0	0	0	3
CIFDVFLPFILC	0	0	0	0	1	3
CIFDTYMPFIPC	0	0	0	0	0	3
CIFDTYLPFIPC	0	0	0	0	0	3
CIFDNWLPYIFC	0	0	0	0	1	3
CIFDNWLPSIPC	0	0	0	0	0	3
CIFDNWLPFIYF	0	0	0	0	2	3
CISDIFMPFIFC	0	0	0	0	0	3
CLFDLFVPFIVC	0	0	0	0	0	3
CLFDLFMPFIFC	0	0	0	0	1	3
CLFDLFLPYIFC	0	0	0	2	16	3
CLFDLLFPFIPC	0	0	1	14	11	3
CLFDIFFPFIFC	0	0	0	3	20	3
CLFDIFCPFITC	0	0	0	0	4	3
CISDTFLPFIYC	0	0	0	1	1	3
CISDSFLPFVFC	0	0	0	1	1	3
CISDLFSPFIFC	0	0	0	0	3	3
CISDLFAPFIYC	0	0	0	0	7	3
CISDIHMPFILC	0	0	0	0	2	3
CISDIFMPFIPC	0	0	0	2	1	3
CIWDPFVPFILC	0	0	0	4	14	3
CIFDNWLPFIFY	0	0	0	0	0	3
CINDIHMPYIPC	0	0	0	0	0	3
CILEIHMPFIPC	0	0	0	0	0	3
CILDSFFPFIPC	0	0	1	9	14	3
CILDLFVPFILC	0	0	0	0	1	3
CIWDSFAPFILC	0	0	0	4	14	3
CIWDSFEPFIFC	0	0	0	2	5	3
CIWDSFEPFILC	0	0	0	2	9	3
CLFDIFCDFIIC	0	0	0	0	0	3
CLFDHFLPFVFC	0	0	0	4	15	3
CLFDHFCPFIFC	0	0	0	2	5	3
CIYHIHMPFIPC	0	0	0	0	1	3
CLFDLFVPLVFC	0	0	0	6	17	3
CLFDLFVPSVFC	0	0	0	5	6	3
CLFDLHSPFIFC	0	0	1	3	7	3
CLFDLYCDFILC	0	0	1	6	36	3
CIFDNWLPC	0	0	0	0	6	3
CIIDIFMPFIPC	0	0	0	0	2	3
CLFDIFCPFIMC	0	0	0	1	1	3
CIIHIHMPYIPC	0	0	0	0	0	3
CLFDLFCPFIMC	0	0	0	0	2	3
CLFDILLPFIYC	0	0	0	2	2	3
CLFDIHMPFTPC	0	0	0	0	1	3
CLFDIFMPFIYC	0	0	0	0	1	3

CLFDIFLTFILC	0	0	0	1	1	3
CLFDLFCPYIFC	0	0	0	3	2	3
CLFDIFLTFIIC	0	0	0	0	4	3
CLFDIFLPLILC	0	0	0	2	6	3
CLFDIFLPFTYC	0	0	0	2	1	3
CLFDIFLPFNPC	0	0	0	0	0	3
CLFDIFLPFNIC	0	0	0	1	3	3
CLFDIFLPFIYS	0	0	0	0	0	3
CLFDIFLPFINC	0	0	0	2	12	3
CLFDIFLPSIPC	0	0	0	0	0	3
CLFDIFLLFIFC	0	0	0	0	2	3
CLFDLFFPLIPC	1	0	1	16	8	3
CLFDLFLPFTFC	0	0	0	1	1	3
CLFDPALPFIFC	0	0	18	71	36	3
CLFDPFCPFIIC	0	0	0	1	2	3
CIFDLFGPFIFC	0	0	0	4	2	3
CLFDNWLPFIFC	0	0	0	1	7	3
CLFDLYSDFIFC	0	0	0	2	7	3
CLFDLYLPFIIC	0	0	0	1	0	3
CLFDLFIPFIIC	0	0	0	3	10	3
CVFDSLLPSIFC	0	0	0	1	3	3
CIFDNWLPFIFS	0	0	0	0	1	3
CVFDTFLPSIFC	0	0	0	0	1	3
CLFDSFLPSIFC	0	0	0	2	1	3
CLFDSFVPLILC	0	0	0	2	3	3
CLFDSFVPIIFC	0	0	0	1	6	3
CLFDSFVHFIFC	0	0	0	2	11	3
CLFDSFMPFIYC	0	0	0	0	2	3
CLFDSFLTFILC	0	0	0	2	2	3
CLFDSFLTFIIC	0	0	0	0	0	3
CLFDSFVPYIYC	0	0	0	0	2	3
CLFDSFLPSILC	0	0	0	5	5	3
CLFDSFLPLIIC	0	0	0	0	1	3
CLFDSFLPFVIC	0	0	0	1	10	3
CLFDSFLPFISC	0	0	0	1	2	3
CLFDSFLHFIIC	0	0	0	0	0	3
CLFDSFHPFIFC	0	0	0	0	2	3
CTFDVLLPFIFC	0	0	6	13	13	3
CLFDSILPFIFC	0	0	0	0	0	3
CLFNLFCPFIFC	0	0	1	2	1	3
CLFDSLVPFVFC	0	0	0	10	20	3
CLFDSYLPFIIC	0	0	0	1	0	3
CLFESFVPFIFC	0	0	0	1	6	3
CLFEIFLPFIIC	0	0	0	2	2	3
CLFDVFSDFIFC	0	0	1	8	7	3
CLFDVFCDFVFC	0	0	0	6	10	3
CLFDVFCDFICC	0	0	1	8	28	3
CLFDTYVPFIFC	0	0	1	0	3	3
CLFDTILPFIFC	0	0	2	14	6	3
CLFDTFYFFILC	0	0	0	1	4	3
CLFDTFVPFITC	0	0	0	0	4	3
CLFDTFLPFSFC	0	0	0	0	1	3

CLFDTFLPFINC	0	0	0	0	0	3	CTFDIFSPFILC	0	0	0	5	14	3
CLFDTFLPFIFR	0	0	0	0	1	3	CQFDIFLPFIIC	0	0	0	1	7	3
CLFGSFVPFVFC	0	0	0	4	8	3	CNFDIHMTFIPC	0	0	0	0	1	3
CLYDSFVPFICC	0	0	0	0	0	3	CSFDTHFPFITC	0	0	0	0	8	3
CTFDVFLPLIFC	0	0	1	8	10	3	CTFDIFLPFISC	0	0	0	0	1	3
CLFVLHLPFIFC	0	0	0	1	3	3	CSFDSFVPFIFC	0	0	0	0	0	3
CLSDTFVPFIFC	0	0	0	8	8	3	CTFDIFLPFIFY	0	0	0	0	0	3
CLSDLHLPFIFC	0	0	0	2	5	3	CTFDIFLPFIFS	0	0	0	0	1	3
CLSDLFFPFIPC	0	0	1	4	2	3	CTFDIFFPFILC	0	0	0	1	3	3
CLSDLFCPFIFC	0	0	0	17	9	3	CTFDIFLHFILC	0	0	0	0	3	3
CLLDSFVPFVFC	0	0	1	6	15	3	CTFDIFLSFILC	0	0	0	1	1	3
CLYDIFLPFIIC	0	0	0	2	11	3	CVFDSILPIIIC	0	0	0	0	0	3
CLLDSFVPFIIC	0	0	0	0	1	3	CTFDTFLPLFFC	0	0	0	0	1	3
CLLDSFLPFIIC	0	0	0	0	1	3	CTFDTFLPLIYC	0	0	0	0	2	3
CLIDSFVPFIFC	0	0	0	1	8	3	CTFDTFLTFIFC	0	1	0	3	2	3
CLIDIFCPFILC	0	0	0	0	6	3	CTFDTFMPFIPC	0	0	0	0	2	3
CLIDIFCPFIFC	0	0	0	1	0	3	CNFDINMPFIPC	0	0	0	1	0	3
CLFYIFLPFIFC	0	0	0	4	2	3	CLFDTYLPFIFC	0	0	0	3	11	3
CLFVSFLPFILC	0	0	0	2	0	3	CTFDTFLPFIFS	0	0	0	1	9	3
CLLDSFLPFIVC	0	0	1	2	5	3	CTFDSWLPYIPC	0	0	1	4	24	3
CLYDLFIPFIFC	0	0	0	7	10	3	CTFDSLLPFIPC	0	0	0	1	7	3
CLYDSFLPFFFC	0	0	0	1	2	3	CTFDSLLPFILC	0	1	1	5	19	3
CLYDSFLNFIFC	0	0	0	0	0	3	CTFDLYLPFIIC	0	0	0	0	1	3
CNFDIHMPFIHC	0	0	0	0	1	3	CTFDLLLPFIPC	0	0	0	1	2	3
FDLFCPFILC	0	0	0	1	3	3	CTFDTFLPFIFW	0	0	0	0	0	3
CLYHSFLPFIFC	0	0	0	0	3	3	CTFDLFLTFIIC	0	0	0	0	0	3
CLYDTFLPFILC	0	0	0	0	4	3	CTFDLFLPLIPC	0	0	0	0	0	3
CLYDSLLPFILC	0	0	0	8	8	3	CTFDLFLPFTYC	0	0	0	1	10	3
CLYDLFLPFIIC	0	0	0	0	5	3	CTFDLFLPFTLC	0	0	0	1	2	3
CLYDSILPFIIC	0	0	0	0	2	3	CTFDLFLPFLCC	0	0	0	0	9	3
CLYDSFMPFIIC	0	0	0	0	1	3	CTFDLFIPFVPC	0	0	2	16	16	3
CLYDSFLTFIIC	0	0	0	0	2	3	CTFDILLPFILC	0	0	3	4	20	3
CLYDSFLRFIFC	0	0	1	0	5	3	CTFDLFLPLIYC	0	0	0	1	5	3
CLYDSFLPFIYS	0	0	0	1	3	3	CTFDTFLPFITC	0	0	0	0	8	3
CLYDSFLPFIMC	0	0	0	1	1	3	CTFDTFLPFLCC	0	0	0	0	8	3
CLFDTFLPFIFF	0	0	0	1	0	3	CTFDTFLPFSFC	0	0	0	1	1	3
CLFDTFLLFIFC	0	0	0	1	4	3	CTFDTILPFIFC	0	0	0	2	4	3
CLFDTFIPFVFC	0	0	0	0	1	3	CNFDNWLPFIFC	0	0	0	0	2	3
CSFDIFLPFIIC	0	0	0	0	1	3	CTFDTHLPFITC	0	0	0	9	17	3
CTFDTFLPFVIC	0	0	0	0	2	3	CTFDTHFPLIIC	0	0	0	0	0	3
CTFDTFLPFTPC	0	0	0	0	1	3	CTFDTHFPFIYC	0	0	0	2	7	3
CTFDIHTPFIPC	0	0	0	0	2	3	CTFDTHFPFIVC	0	0	0	0	3	3
CTFDIHMPFTPC	0	0	0	2	8	3	CTFDTHFPFIAC	0	0	1	3	5	3
CTFDTFLPFTLC	0	0	0	0	1	3	CTFDTFVPFIFC	0	0	0	0	8	3
CTFDIFSPFIIC	0	0	0	0	2	3	CTFDTFQPFIYC	0	0	0	0	0	3
CRFDIFLPFIFC	0	0	0	1	7	3	CLYDCFLPFIFC	0	0	0	0	1	3
CNIDIHKPFIPC	0	0	0	0	0	3	CNFDTFLPFIIC	0	0	0	0	1	3
CQFDIFCPFILC	0	0	0	1	6	3	CVYDLHLPFIFC	0	0	0	0	1	3
CPYDSFLPFIYC	0	0	0	0	2	3	CVFDLFCPFIYC	0	0	0	0	1	3
CPFDTFLPFIIC	0	0	0	0	3	3	CVFDCFLPFIFC	0	0	0	0	4	3
CPFDLFCPFIIC	0	0	0	0	0	3	CTSDTFLPFILC	0	0	0	0	0	3
CNYYINKHFITC	0	0	0	0	0	3	CTSDLFLPFIYC	0	0	0	0	1	3

CTLDVFLPFIFC	0	0	0	6	14	3	CVFDVSCPFIFC	0	0	0	5	10	3
CTLDTFLPFIIC	0	0	0	0	0	3	CVFDVFCPSIFC	0	0	0	5	12	3
CTLDLFLPFIYC	0	0	1	1	4	3	CVFVSFLPFIFC	0	0	0	0	2	3
CTYDVFLPFIFC	0	0	0	3	5	3	CVFDSFLPFSLC	0	0	0	0	1	3
CVFDLFLPFIHC	0	0	0	0	3	3	CVFDSFLPFLCC	0	0	0	0	0	3
CVFDLFLPFIMC	0	0	0	2	2	3	CVFDSFLPFIPS	0	0	0	0	1	3
CVFDLFLPFINC	0	0	0	0	0	3	CVFDIHMPFIPY	0	0	0	0	2	3
CVFDSFLPFILR	0	0	0	1	1	3	RIFDLFVPFIFC	0	0	0	0	3	3
CVFDSFLPFIIG	0	0	0	0	1	3	CVFDFFLPFILC	0	0	8	20	13	3
CVFDSFLPFIAC	0	0	0	2	3	3	RIFDIFMPFIPC	0	0	0	0	1	3
CVFDGFCPFIFC	0	0	0	4	6	3	CVYDLCLPFIYC	0	0	0	0	5	3
CVFDSFLHFIFC	0	0	0	0	3	3	CVYDIFVRFIFC	0	0	0	0	1	3
CVFDSFKPFIIC	0	0	0	0	0	3	CVYDIFEPFIFC	0	0	0	0	2	3
CVFDSFFPFVLC	0	0	0	0	1	3	CVYDIFVPFIIR	0	0	0	1	7	3
CVFDSFFPFVFC	0	0	0	3	10	3	CVYDIFVPSIFC	0	0	0	0	2	3
CVFDPWLPFVYC	0	0	0	6	11	3	CVYDIFVQFIFC	0	0	0	0	1	3
CVFDLYLPFIIC	0	0	0	2	11	3	CVYDLFLSFIYC	0	0	0	3	1	3
CVFDLWLPFYCC	0	0	0	0	6	3	CYFEPHFIFIPC	0	0	0	0	5	3
CVFDLSLPFTFC	0	0	0	4	5	3	CVYDLFFPFIYC	0	0	0	2	5	3
CVFDLSLPFIYC	0	0	0	0	1	3	CVYDLFLPFISC	0	0	0	3	9	3
CVFDLFLPFTFY	0	0	0	0	2	3	CVYDLFLPFIYG	0	0	0	0	0	3
CVLDSFLPFIIS	0	0	0	0	0	3	CVYDLFLPFNYC	0	0	0	2	12	3
CTLDIFLPFIFC	0	0	0	0	3	3	CVYDLFLPIIYC	0	1	1	1	4	3
CVSDLFLPFIYC	0	0	1	0	1	3	CVYDLFLPLIFC	0	0	0	2	4	3
CVSDSFLPFVLC	0	0	1	8	6	3	CVYDLFVPFIFC	0	0	0	0	2	3
CVFDFFCPFIFC	0	0	0	0	2	3	CLFDPSLSFIPC	0	0	1	24	7	3
CVFDIFMPFIYC	0	0	0	0	0	3	CVYDLLLPFIFC	0	0	0	2	5	3
RIFDIHMPFILC	0	0	0	0	1	3	CYFDPHSIFIPC	0	0	0	4	11	3
CVIDSFLPFIFC	0	0	0	0	1	3	CYFDPHFIFIYC	0	0	0	1	2	3
CVFDSFMPFIIS	0	0	0	0	0	3	CYFDIFLPFICC	0	0	0	2	9	3
CVFDSFMPFIYC	0	0	0	0	1	3	CVYVIFVPFIIC	0	0	0	3	12	3
CTFGTFLPFIYC	0	0	0	0	0	3	CVYNLFLPFIFC	0	0	0	0	1	3
CVFDSFLTFNIC	0	0	0	0	0	3	CVYGIFVPFIPC	0	0	0	0	3	3
CVFDSFLSFIPC	0	0	0	0	0	3	CVYELFLPFIYC	0	0	0	2	4	3
CVFDSFLPYIIS	0	0	0	0	2	3	RIFDIFMPFILC	0	0	0	0	6	3
CVFDSFLPFVVC	0	0	0	2	1	3	CVYDVFVPFIFC	0	0	0	0	3	3
CVFDSFSPFIVC	0	0	0	0	0	3	CVYDSYLPFIIC	0	0	0	0	1	3
CVFDSFLPFVRC	0	0	0	3	8	3	CVSDSFLPFIPC	0	0	0	0	2	3
CVFDTFVPFIIC	0	0	0	0	0	3	CYFDSHFIFIPC	0	0	0	2	1	3
CVFDTWLPFVYC	0	0	0	0	5	3	RIFDTFLPFILC	0	0	0	0	2	3
CVLDLFLPFIYC	0	0	0	0	1	3	FYFDPHFIFIPC	0	0	0	0	3	3
CVLDLFLPFIIC	0	0	0	0	1	3	FVFDSFLPFIFC	0	0	0	0	1	3
CVLDIFIPFVLC	0	0	1	2	7	3	FIFDLFVPFIFC	0	0	0	0	1	3
CVHDLFLPFIFC	0	0	0	0	0	3	CYYDPHFIFIPC	0	0	0	2	9	3
CVFDIHMPFIHC	0	0	0	1	2	3	CYYDLFLPFIFC	0	0	0	2	12	3
CVHDIFVPFIFC	0	0	0	0	0	3	FVYDLFLPFIYC	0	0	0	0	3	3
CVFDTLLPFIFC	0	0	0	5	8	3	FLFDSFVPFIFC	0	0	0	2	1	3
CVFYLFLPFIFC	0	0	0	0	0	3	RLFDIFLPFIIC	0	0	0	0	7	3
CVFNSFLPFIFC	0	0	0	0	2	3	YIFDLFCPFIVC	0	0	0	1	0	3
CVFHSFLPFILC	0	0	0	0	0	3	RLFDSFVPFVFC	0	0	0	0	2	3
CVFESFLPFVLC	0	0	0	0	2	3	RTFDIFLPFIFC	0	0	0	0	0	3
CVFELYTPFILC	0	0	19	91	57	3	SVYDLFLPFIFC	0	0	0	0	0	3

SVFDSFLPFVLC	0	0	0	2	1	3
STFDTFLPFIFC	0	0	0	0	4	3
SLFDSFVPFIFC	0	0	1	1	3	3
RLFDLFVPFVFC	0	0	0	0	0	3
SLFDLFVPFVFC	0	0	0	0	1	3
SIFDNWLPFIFC	0	0	0	0	1	3
RVYDLFLPFIFC	0	0	0	0	1	3
RVFDSFLPFILC	0	0	0	0	8	3
RTFDTFLPFIFC	0	0	0	0	7	3
SLFDLFCPFIFC	0	0	0	0	3	3
CCFDSFLRFIFC	0	0	0	0	1	2
CCFDLFFPFIFC	0	0	0	1	7	2
CCFDSFLPIIFC	0	0	0	0	5	2
CCFDSFLPLIYC	0	0	0	0	0	2
CCFDSLLPFIIC	0	0	0	0	2	2
CCFDSFLPLIIC	0	0	0	0	0	2
CCFDSFVPFIIC	0	0	0	0	0	2
CCFDSLLPFIYC	0	0	0	0	0	2
CCFDSSLPFIIC	0	0	0	0	1	2
CCFDVFLPFIFC	0	0	0	4	6	2
CCFESFLPFIFC	0	0	1	0	3	2
CCLDIFLPFIYC	0	0	0	0	0	2
CCFDSFPPFIIC	0	0	0	0	0	2
CCFDSFLPFINC	0	0	0	0	0	2
CCLDSFLPFIIC	0	0	0	0	0	2
CCFDSFLPFVMC	0	0	0	7	13	2
CCFDSFLPFIAC	0	0	0	0	1	2
CCFDIFMPFILC	0	0	0	0	3	2
CCFDSFLLFIFC	0	0	0	0	4	2
CFWDSFVPFIFC	0	0	0	0	4	2
CFYDLFLPSIFC	1	1	0	1	12	2
CCFDIFVPFIFC	0	0	0	0	0	2
CDFDLFLPFTFC	0	0	0	3	4	2
CAFDIFLPFIFC	0	0	0	0	2	2
CAFDIFMPFILC	0	0	0	0	1	2
CDYDIFVPFILC	0	0	0	0	1	2
CDYDIHMPFIPC	0	0	0	0	0	2
CEFDSYLPFIIC	0	0	0	0	3	2
CEFDSYMPFIIC	0	0	0	0	0	2
CEIDSFLPFIIC	0	0	0	0	1	2
CEFHSFLPFIIC	0	0	0	0	0	2
CDFDIFIPFVFC	0	0	0	0	2	2
CDFDIHMPFIPS	0	0	0	0	0	2
CDFDLFLPFIYC	0	0	0	0	1	2
CDFDNWLPFIYC	0	0	0	0	0	2
CDFDSFMPFILC	0	0	0	0	0	2
CFYDLFMPFIFC	0	0	0	2	5	2
CFNDLFLPFIYC	0	0	0	0	0	2
CFYDLFSPFIFC	0	0	1	6	13	2
CFYDLFVPFIIC	0	0	0	0	0	2
CCSDIFLPFIFC	0	0	0	2	6	2
CAYDIFVPFIPC	0	0	0	0	0	2
CAFDLFLPFILC	0	0	0	1	4	2
CAFDLFLPFTFC	0	0	0	3	9	2
CAFDSFLPFISC	0	0	0	0	0	2
CAFDSFLPFVLC	0	0	0	10	10	2
CCFDIFSPFIFC	0	0	1	1	6	2
CAYDLFLPFIPC	0	0	0	0	1	2
CAYDLFLPFILC	0	0	0	0	0	2
CCFDIFLHFIFC	0	0	0	0	1	2
CCFDIFLPFIFR	0	0	0	1	1	2
CCFDIFLPYIFC	0	0	0	1	2	2
CCFDIFLSFIFC	0	0	0	1	1	2
CLFDNFLPFIIC	0	0	0	1	4	2
CIFDIVMPFILC	0	0	0	0	0	2
CFFDLFFPFVPC	0	0	2	9	8	2
CFFDLFLPFIIC	0	0	0	0	3	2
CFFDLFLPFIYC	0	0	0	1	6	2
CFFDLSLPFVCC	0	0	0	1	4	2
CFFDNWLPFIFC	0	0	0	0	2	2
CFFDPFFFFILC	0	0	0	0	2	2
CFFDPFFSFIPC	0	0	0	1	3	2
CFFDLFCPFIPC	0	0	0	4	6	2
CFFDPHLPFIIC	0	1	0	0	3	2
CFFDSFMPFILC	0	0	0	0	10	2
CFFDSFMPFIYC	0	0	0	1	12	2
CFFDSFVPFIIC	0	0	0	0	1	2
CFFDTFLPFICC	0	0	0	0	1	2
CFFDTFLPFIIC	0	0	0	0	1	2
CIFDLFAHFIYC	0	0	0	0	1	2
CILDSFLPFVFC	0	0	0	0	0	2
CIWDPFVPFIPC	0	0	0	0	0	2
CIFDIFMPYIFC	0	0	0	0	0	2
CIWDSFVPFIFY	0	0	0	0	1	2
CIWDSFMPFIIC	0	0	0	0	1	2
CILDTFLPFIPC	0	0	1	0	3	2
CILDTHMPFIPC	0	0	0	0	1	2
CILEIHMPLIPC	0	0	0	0	1	2
CINHIHMPFIPC	0	0	0	0	0	2
CIRDSFVPFIFC	0	0	1	1	11	2
CISDLFLPFIYC	0	0	0	0	0	2
CISDNWLPFICC	0	0	0	0	2	2
CISDTFLPFIPC	0	0	0	0	3	2
CISELNLPFIPC	0	0	0	2	2	2
CFFDTFLPFILC	0	0	0	1	1	2
CFFDTFLPFIPC	0	0	0	0	0	2
CFFDSFLPFIFC	0	0	1	3	7	2
CFFDVFCPFIFC	0	0	0	0	2	2
CFYDIFLPFIFC	0	0	0	0	1	2
CFYDIHMPFIPC	0	0	0	0	1	2
CFYDLFCLLFFC	0	0	0	0	1	2
CFYDLFLPFISC	0	0	0	0	11	2
CFYDLFLPFVFC	0	0	0	1	14	2
CFFDIHMPFTPC	0	0	0	0	0	2

CFFDIFMPFIIC	0	0	0	0	0	2
CEYDSYLPFIIC	0	0	0	0	0	2
CFCDLFLPFIFC	0	0	0	2	3	2
CFFDIFMPFIPC	0	0	0	0	3	2
CFFDIFLPFIIC	0	0	0	0	0	2
CFFDIFLPFIYC	0	0	0	0	0	2
CFFDIFMPFIFC	0	0	0	0	1	2
CIFDIFMPLFFC	0	0	0	0	0	2
CIFDIFMRFILC	0	0	0	0	0	2
CHFDLFCPFIIC	0	0	0	0	2	2
CHFDTFLPFIFC	0	0	0	3	9	2
CHFDTYLPFIYC	0	0	0	0	0	2
CIFDKWLPFIFC	0	0	0	0	3	2
CGYDIFVPFIIC	0	0	0	0	2	2
CFYDLILPFIFC	0	0	0	0	2	2
CFYDLMLPFIFC	0	0	0	0	4	2
CFYDLYLPFIFC	0	0	0	1	2	2
CFYHLFLPFIFC	0	0	0	0	0	2
CFYVTFVPFIFC	0	0	0	0	0	2
CLFDNWLPFILC	0	0	0	0	0	2
CIFDIFMPYIPC	1	0	0	0	0	2
CLFDPFFIFFPC	0	0	31	130	23	2
CVFDSFLPIIPC	0	0	0	0	0	2
CVFDSFLPLFFC	0	0	0	0	0	2
CVFDSFLPLIIS	0	0	0	0	0	2
CVFDSFLPSIMC	0	0	0	0	2	2
CIFDIHMTIIPS	0	0	0	0	0	2
CIFDIFMSFIFC	0	0	0	0	0	2
CIFDAFLPFIPC	0	0	0	0	1	2
CIFDAFVPFIVC	0	0	0	3	20	2
CIFDAHMPFIPC	0	0	0	1	1	2
CIFDDWLPFIFC	0	0	0	1	3	2
CIFDIFMPFFLC	0	0	0	0	3	2
CIFDFFLPFIFC	0	0	0	2	3	2
CIFDFHMPFITC	0	0	0	0	0	2
CIFDFHMPLIPC	0	0	0	0	1	2
CIFDFNMPFIPC	0	0	0	0	0	2
CIFDIDMPFISC	0	0	0	0	0	2
CIFDIFIPFVPC	0	0	0	0	1	2
CIFDIFLPFINC	0	0	0	0	0	2
CIFDIFLPLIFC	0	0	0	0	1	2
CIFDFFMPFILC	0	0	0	0	2	2
CIFDIHMNFIHC	0	0	0	0	3	2
CIFDAFLPFILC	0	0	0	1	1	2
CIFDIHMTFIQC	0	0	0	0	0	2
CIFDIFMLFIPC	0	0	0	0	4	2
CIFDIFMTFIFC	0	0	0	0	0	2
CIFDIFRPFILC	0	0	0	0	0	2
CIFDIFTPFIFC	0	0	0	0	10	2
CIFDIFMPFIFS	0	0	0	0	1	2
CIFDIFTPFIIC	0	0	0	1	1	2
CIFDIHIHFIPC	0	0	0	0	0	2

CIFDIHIPFIFC	0	0	0	0	1	2
CIFDIHIPFIHC	0	0	0	0	1	2
CIFDIHIPFVLC	0	0	0	0	3	2
CIFDIHIPIIPC	0	0	0	0	1	2
CIFDIHKHFIPC	0	0	0	0	0	2
CIFDIHKHYIPC	0	0	0	0	0	2
CIFDIHFPFITC	0	0	0	0	2	2
CIFDIHKPIIPC	0	0	0	2	2	2
CIFDIFMPFFPC	0	0	0	0	1	2
CIFDIHMNFINC	0	0	0	0	0	2
CIFDIHMPFIHS	0	0	0	0	0	2
CIFDIIMPIILC	0	0	0	0	0	2
CIFDIKIPLIPC	0	0	0	0	0	2
CIFDIHMPFIMC	0	0	0	0	1	2
CIFDIHMPFIPT	0	0	0	0	2	2
CIFDIHMPFKPC	0	0	0	1	1	2
CIFDIHMPFLCC	0	0	0	0	1	2
CIFDIHMPFTPS	0	0	0	1	0	2
CIFDIHMPFTTC	0	0	0	1	2	2
CIFDIHMPFYPC	0	0	0	0	3	2
CIFDIHMPIIPS	0	0	0	0	4	2
CIFDIHMPINPS	0	0	0	0	0	2
CIFDIHMPLIAC	0	0	0	0	0	2
CIFDIHMPLIIC	0	0	0	0	0	2
CIFDIHMPYIIC	0	0	0	0	0	2
CIFDIHMPYITC	0	0	0	0	3	2
CIFDIHMQFIPC	0	0	0	0	0	2
CIFDIHMTFIPF	0	0	0	0	0	2
CIFDIHNPFIPC	0	0	0	0	0	2
CIFDIHTPLIPC	0	0	0	0	0	2
CIFDIIMPFIFC	0	0	0	0	0	2
CIFDINMPFILC	0	0	0	0	3	2
CIFDIKMPFIPC	0	0	0	0	0	2
CIFDILMPFINC	0	0	0	0	0	2
CIFDINIPFIPC	0	0	0	0	1	2
CIFDINIPFITC	0	0	0	0	1	2
CIFDINMHFITC	0	0	0	0	1	2
CIFDIIMPFINC	0	0	0	0	0	2
CIFDINMPFTPC	0	0	0	0	2	2
CIFDINMPFIQC	0	0	0	0	1	2
CIFDINMRFIPC	0	0	0	0	0	2
CIFDIQKPFIPC	0	0	0	0	0	2
CIFDIQMPFTPC	0	0	0	0	0	2
CIFDIRMPFILC	0	0	0	0	1	2
CIFDIRMPLIPC	0	0	0	0	2	2
CIFDISMPFIFC	0	0	0	0	1	2
CIWDSFVPFTFC	0	0	2	0	4	2
CLFDIFCSFILC	0	0	0	1	2	2
CIYDIHMPFIPY	0	0	0	0	0	2
CIFDLLSPFIFC	0	0	0	2	4	2
CIFDLLVPFILC	0	0	0	0	1	2
CIFDLSCPFIFC	0	0	0	0	2	2

CIFDLSSPFIFC	0	0	0	3	4	2
CIFDLSVPFIPC	0	0	0	0	0	2
CIFDLLLPFIFC	0	0	0	1	0	2
CIFDLWLPFIVC	0	0	0	0	1	2
CIFDLFVPFIIS	0	0	0	0	0	2
CIFDLFVHFIFC	0	0	0	1	3	2
CIFDLFAPSIYC	0	0	0	0	5	2
CIFDLFCLFILC	0	0	0	0	0	2
CIFDLFCPFIHC	0	0	0	0	1	2
CIFDLFCPFIVR	0	0	0	2	3	2
CIFDLFCPFIVS	0	0	0	0	9	2
CIFDLFCPYIVC	0	0	0	2	10	2
CIFDLFEPFIFC	0	0	0	0	1	2
CIFDLLVPFIIC	0	0	0	0	1	2
CIFDLLLPFVFC	0	0	0	1	2	2
CIFDTHLPFIPC	0	0	0	0	0	2
CIFDLLAPFIYC	0	0	1	0	9	2
CIFDTHMPFITC	0	0	0	1	1	2
CIFDTHMPFNPC	0	0	0	0	2	2
CIFDTHYPFILC	0	0	0	0	0	2
CIFDSFCPFILC	0	0	0	0	9	2
CIFGNWLPFILC	0	0	0	0	1	2
CIFDQFVPFIFC	0	0	1	3	6	2
CIFDPFLPFVFC	0	0	0	1	5	2
CIFDLFVPFLCC	0	0	0	1	2	2
CIFDLFVPFSFC	0	0	0	0	1	2
CIFDLFVPFTLC	0	0	0	2	0	2
CIFDLFVPLILC	0	0	0	0	2	2
CIFDLFVPLIPC	0	0	0	0	0	2
CIFDLFVPLIYC	0	0	0	0	1	2
CIFDLFYPFIIC	0	0	0	0	2	2
CIFDLFVPFINC	0	0	0	0	0	2
CIFDLFVLFIFC	0	0	0	1	1	2
CIFDLFFPFAPC	0	0	0	1	2	2
CIFDLFLPFTLC	0	0	0	0	1	2
CIFDLFLPFTYC	0	0	0	1	1	2
CIFDNWLQFIFC	0	0	0	0	3	2
CIFDNWLQFILC	0	0	0	0	0	2
CIFDNWLTFIIC	0	0	0	0	0	2
CIFDNWLTFIYC	0	0	0	0	6	2
CIFDNWPPFIIC	0	0	0	0	2	2
CIFDNWRPFIYC	0	0	0	0	1	2
CIFDNWRQFIYC	0	0	0	0	0	2
CIFDNWLPSIIC	0	0	0	0	1	2
CIFDNWLKFIYC	0	0	0	0	0	2
CIFDNWFPFIVC	0	0	0	0	0	2
CIFDNSLPFIYC	0	0	0	0	4	2
CIFDLWLPLTAC	0	0	1	6	4	2
CIFDLWPPFTPC	0	0	0	0	1	2
CIFDLYDPFIFC	0	0	0	0	0	2
CIFDMFCPFIVC	0	0	0	2	11	2
CIFDNWLPLILC	0	0	0	0	1	2
CIFDNWLPFILS	0	0	0	0	0	2
CIFDNWLPIINC	0	0	0	0	0	2
CIFDLWLPFIYC	0	0	0	0	0	2
CIFDLFLTFIFC	0	0	0	0	0	2
CIFDLFMPFILC	0	0	0	0	0	2
CIFDLFMPFIYC	0	0	0	0	1	2
CIFDLFSPFVFC	0	0	0	4	5	2
CIFDLFLPFIVC	0	0	0	0	1	2
CIFDLWLPFTSC	0	0	1	0	1	2
CIFDNWLPIIIC	0	0	0	0	0	2
CIFDLWLPITAC	0	0	0	0	2	2
CIFDNWLPFMYC	0	0	0	0	1	2
CIFDNWLPFNYC	0	0	0	1	13	2
CIFDNWLPFSYC	0	0	0	0	3	2
CIFDNWLPFTPC	0	0	0	0	6	2
CIFDNWLPFVPC	0	0	0	0	0	2
CIFDTHMLFIPC	0	0	0	0	2	2
CIFDTHFPSILC	0	0	0	0	0	2
CIYDIHMPFIHC	0	0	0	0	0	2
CIFDTFLPFFFC	0	0	0	0	2	2
CIFDSFYSFIPC	0	0	0	2	5	2
CIFDSFMPFIIC	0	0	0	0	0	2
CIFDSFCPFIPC	0	0	0	0	1	2
CIFDSFFLLFFC	0	0	0	0	0	2
CIFDSFFPFIFS	0	0	0	0	3	2
CIFDSFFPFIFY	0	0	0	0	2	2
CIFDSFFPFIHC	0	0	2	6	17	2
CIFDSFFPFIPS	0	0	1	6	6	2
CIFDSFSPFIIC	0	0	0	0	1	2
CIFDSFFPFIPY	0	0	0	1	5	2
CIFDSFFPIIFC	0	0	0	0	2	2
CIFDSFIPFIIC	0	0	0	0	2	2
CIFDSFLPFIMC	0	0	0	0	1	2
CIFDTFLPFNLC	0	0	0	0	1	2
CIFDTFLPFILS	0	0	0	0	1	2
CIFDTHFPFVLC	0	0	0	0	1	2
CIFDTFLPFILR	0	0	0	0	0	2
CVFDSFLPYINT	0	0	0	0	0	2
CIFDSLLPFVFC	0	0	0	0	0	2
CIFDSLLPFVIC	0	0	0	1	1	2
CIFDSWLPFVLC	0	0	0	0	0	2
CIFDSWLPFVYC	0	0	1	2	20	2
CIFDSWLPLIYC	0	0	0	0	0	2
CIFDSWLPYIFC	0	0	0	0	0	2
CIFDTFKPFIFC	0	0	0	0	2	2
CIFDSIFPFIPC	0	0	0	0	2	2
CIFDTFLHFILC	0	0	0	0	2	2
CIFDTFLPFIAC	0	0	0	0	1	2
CIFDTFLPFIFF	0	0	0	1	0	2
CIFDTFLPFIFT	0	0	0	0	0	2
CIFDSFLPFTFC	0	0	0	0	0	2
CIFDSFLPFVCC	0	0	0	0	10	2

CIFDSFLSFILC	0	0	0	0	0	2
CIFDSFFPFTFC	0	0	0	3	12	2
CIFGLFVPFIYC	0	0	0	0	1	2
CIFEIHMPYIPC	0	0	0	0	0	2
CIFDTLLPLIFC	0	0	0	0	1	2
CIFDTLLPFICC	0	0	0	0	1	2
CIFDTILPFINC	0	0	0	0	0	2
CIFDTFLPSIIC	0	0	0	0	3	2
CIFDTFLPSIVC	0	0	0	0	0	2
CIFDTFLPSIYC	0	0	0	0	2	2
CIFDTFLPYNFC	0	0	0	0	0	2
CIFDTFLSFIYC	0	0	0	0	1	2
CIFDTFMPIIFC	0	0	0	0	1	2
CIFDTFMPYIFC	0	0	0	0	0	2
CIFDTFPPFIFC	0	0	0	0	1	2
CIFDTFSPFIIC	0	0	0	0	5	2
CIFDTFVPFIVC	0	0	0	0	0	2
CIFGIHMPFITC	0	0	0	0	0	2
CIFGIHMPFILC	0	0	0	0	3	2
CIFGIFMPFIFC	0	0	0	0	0	2
CIFDVFCPFIIC	0	0	0	0	0	2
CIFDTFLPFNYC	0	0	0	0	0	2
CIFDTFLPFVVC	0	0	0	0	0	2
CIFDTFLPINFC	0	0	0	0	0	2
CIFDTMLPFIIC	0	0	0	0	0	2
CIFDTWLPFIFC	0	0	0	0	0	2
CIFDTYLPFILC	0	0	0	0	1	2
CIFDVFMPFIPC	0	0	0	1	5	2
CIFETFLPFTFC	0	0	0	0	0	2
CIFDVHMPFIIC	0	0	0	0	0	2
CIFDVHMPFILC	0	0	0	0	2	2
CIFDTMLPFIFC	0	0	0	0	3	2
CIFDVHMPLIPC	0	0	0	0	0	2
CIFELNFPFIPC	0	0	0	1	3	2
CIFDNFFPFIFC	0	0	0	0	1	2
CIFDNFLPFILC	0	0	0	0	1	2
CIFDNFLTLIFC	0	0	0	0	0	2
CIYDIHTPFIPC	0	0	0	0	1	2
CIFYTFLPFIYC	0	0	0	0	0	2
CIIDIHKPYIPC	0	0	0	0	0	2
CIIDIHMPFTPC	0	0	0	0	2	2
CIIDIHMPLIPC	0	0	0	0	0	2
CIIDIHMPYITC	0	0	0	0	1	2
CIIDIQMPFIPC	0	0	0	0	0	2
CIFYNWLPFIIC	0	0	0	0	0	2
CIIDTFMPFIFC	0	0	0	0	1	2
CIIDTIMPFIFC	0	0	0	0	0	2
CILDIFLPFIFC	0	0	0	0	0	2
CILDIFMPFIIC	0	0	0	0	0	2
CILDIHMPFIHC	0	0	0	0	1	2
CILDLFAPFIYC	0	0	0	3	4	2
CILDLFVPFIIC	0	0	0	0	0	2
CILDLFVPFIYC	0	0	0	0	0	2
CIFYTFLPFILC	0	0	0	0	1	2
CIYDINMPFITC	0	0	0	0	0	2
CIFDNFMPFIIC	0	0	0	0	0	2
CIYDLFAPFIYC	0	0	0	0	2	2
CLFDIFYPFIFC	0	0	0	0	1	2
CLFDIFYPFILC	0	0	0	8	6	2
CLFDIILPFIFC	0	0	0	1	3	2
CLFDISCPFIFC	0	0	0	0	3	2
CLFDISSPFIFC	0	0	0	0	0	2
CLFDIYLPFIIC	0	0	0	0	2	2
CLFDLFCDFIFC	0	0	0	1	1	2
CLFDLFCLFIFC	0	0	0	5	1	2
CLFDLFCPFLCC	0	0	0	0	0	2
CLFDLFCPIIFC	0	0	0	3	4	2
CLFDLFCPLILC	0	0	0	1	1	2
CLFDLFCPSIFC	0	0	0	5	12	2
CLFDLFCPYIYC	0	0	0	0	1	2
CLFDLFEPFIFC	0	0	0	0	1	2
CIYDLFCPFIFC	0	0	0	0	0	2
CIIDTILPFIFC	0	0	0	0	1	2
CIFYNWLPFIFC	0	0	0	0	4	2
CIFYINMPFIHC	0	0	0	0	0	2
CIFYIFMPFILC	0	0	0	1	1	2
CILDTFLPFICC	0	0	0	0	0	2
CIWDSFVPYILC	0	0	0	0	7	2
CIWDSFVTFILC	0	0	0	0	4	2
CIWDSLVPFILC	0	0	0	8	14	2
CIWDSSVPFIFC	0	0	0	2	1	2
CIWDTFVPFIFC	0	0	0	0	0	2
CIWGSFVPFILC	0	0	0	3	11	2
CIWDSFVPSIFC	0	0	0	0	2	2
CIYDIFVPFIMC	0	0	0	0	2	2
CIYDIHKPYIPC	0	0	0	0	0	2
CIYDIHKPYITC	0	0	0	1	0	2
CIYDIHKPYNPC	0	0	0	0	0	2
CIYDIHMPFIFC	0	0	0	0	0	2
CILDNWLPFIIC	0	0	0	0	1	2
CIFDLFAPFVFC	0	0	0	0	4	2
CILDLWLPFTAC	0	0	0	4	6	2
CIFNNWLPFIYC	0	0	0	3	6	2
CIFGTFLPFIPC	0	0	0	0	0	2
CIFGTFLPFIYC	0	0	0	0	0	2
CIFGTHMPFIPC	0	0	0	0	1	2
CIFHNWLPFIYC	0	0	0	1	2	2
CIFNIHMPFILC	0	0	0	0	0	2
CIFNNWLPFICC	0	0	0	0	0	2
CIFNSFFPFIFC	0	0	0	0	4	2
CIFVTFLPFIIC	0	0	0	0	0	2
CIFNTFLPFIIC	0	0	0	0	1	2
CIFNTFLPFIYC	0	0	0	0	0	2
CIFVIFMPFIFC	0	0	0	0	1	2

CIFVIFMPFIPC	0	0	0	0	1	2
CIFVNWLPFIYC	0	0	0	4	7	2
CLFDIFVPFIYC	0	0	0	0	0	2
CLFDIFVPFILC	0	0	0	0	3	2
CLFDIFSPFIMC	0	0	0	0	1	2
CIYDNHMPFIPC	0	0	0	0	0	2
CLFDIFLPFIFR	0	0	0	1	4	2
CLFDIFCPFNFC	0	0	0	0	0	2
CLFDIFLPFIIR	0	0	1	0	1	2
CLFDIFLPFIPS	0	0	0	0	0	2
CLFDIFLPFSIC	0	0	0	0	0	2
CLFDIFLPFTIC	0	0	0	5	18	2
CLFDIFLPFVCC	0	0	0	0	0	2
CLFDIFLPFVYC	0	0	0	1	3	2
CLFDIFLPLIPC	0	0	0	0	0	2
CLFDIFLPYIYC	0	0	0	0	0	2
CLFDIFLPFIKC	0	0	0	0	0	2
CLFDIFLRFIFC	0	0	0	1	3	2
CLFDIFCLFILC	0	0	0	1	1	2
CLFDGFCDFIFC	0	0	0	6	2	2
CIYDLFSPFIFC	0	0	0	1	0	2
CLFDIFLPFIFF	0	0	0	0	0	2
CIFDNNIPFIKC	0	0	0	0	0	2
CIFDNGLPFIYC	0	0	0	1	1	2
CIFDNHKPFIPC	0	0	0	0	2	2
CIFDNHMNLIPC	0	0	0	0	0	2
CIFDNHMPFIHC	0	0	0	0	0	2
CIFDNLLPFIFC	0	0	0	0	2	2
CIFDNLLPFIIC	0	0	0	0	1	2
CIFDNNMPFINC	0	0	0	0	1	2
CLFDIFCPYILC	0	0	0	4	8	2
CIFDNRLPFIIC	0	0	0	0	1	2
CIFDPHMPFIPC	0	0	0	0	0	2
CIFGNWLPFIPC	0	0	0	0	1	2
CIFGSFFPFIPC	0	0	0	7	5	2
CIFGSFLPFIIC	0	0	0	0	0	2
CLFDIFCPLIFC	0	0	0	0	3	2
CIYDLWLPFTAC	0	0	0	1	3	2
CIYDSFFPFIPC	0	0	0	1	7	2
CLFDLFFPFNPC	0	0	0	2	1	2
CIYDSFLPFVFC	0	0	0	0	1	2
CLFDLFVPFVVC	0	0	0	1	12	2
CLFDLFVPIVFC	0	0	0	0	1	2
CLFDLFVTFVFC	0	0	0	0	7	2
CLFDLFMPFILC	0	0	0	0	3	2
CLFDLFWPFIFC	0	0	0	0	3	2
CLFDLFYPFIYC	0	0	0	0	7	2
CLFDLFYPSIIC	0	0	0	2	5	2
CLFDLHLPFITC	0	0	0	0	2	2
CLFDLHLPFIVC	0	0	0	1	13	2
CLFDLICPFIFC	0	0	0	3	4	2
CLFDLYCDFIVC	0	0	0	0	1	2

CLFDNFLPFIFC	0	0	0	2	2	2
CLFDLFYHFIIC	0	0	0	0	2	2
CLFDLFHPFIIC	0	0	0	5	3	2
CLFDLFFPFTIC	0	0	0	0	2	2
CLFDLFVPFVSC	0	0	2	11	12	2
CLFDLFVPFVFS	0	0	0	1	3	2
CLFDLFSPFIPC	0	0	0	9	6	2
CIYDTFMPFIFC	0	0	0	0	3	2
CIYGIHMPFIPC	0	0	0	0	0	2
CLFDHFIDFIIC	0	0	0	0	7	2
CIYHIHKPFIPC	0	0	0	0	0	2
CKFDTFLPFIIC	0	0	0	0	1	2
CKFDTFLPFILC	0	0	0	0	2	2
CLDDSFLPFIFC	0	0	0	0	1	2
CLFDLFRPFIIC	0	0	0	0	1	2
CLFDFFLPFIIC	0	0	0	0	2	2
CLFDFFVPFIFC	0	0	0	0	2	2
CIYYIHMPFIPC	0	0	0	0	0	2
CLFDIFLRFIIC	0	0	0	0	1	2
CLFDIFLSFIFC	0	0	0	2	2	2
CLFDIFRPFILC	0	0	0	15	8	2
CVFDSFLPSVFC	0	0	0	0	0	2
FDTFLPFIYC	0	0	0	0	1	2
CVFDSFLSFILC	0	0	0	2	8	2
CLFDSFVPFIFY	0	0	0	2	9	2
CLFDSFVPFVFY	0	0	0	1	3	2
CLFDSFVPSVFC	0	0	0	7	6	2
CLFDSFLPFINC	0	0	0	0	1	2
CTFGTFLPFILC	0	0	0	0	0	2
CLFDPFLPFIFC	0	0	0	2	6	2
CLFDPFLPFIIC	0	0	0	0	1	2
CLFDPFLSFIIC	0	0	0	0	1	2
CLFDPFLSLIPC	0	0	0	25	5	2
CLFDPFVPFIPC	0	0	0	0	2	2
CLFDPHFIFIPC	0	0	0	0	2	2
CLFDRFLPFIIC	0	0	0	0	0	2
CLFDSFAPFIIC	0	0	0	1	2	2
CLFDSFAPFVFC	1	0	0	6	15	2
CLFDSFVPFTYC	0	0	0	1	1	2
CLFDSFVPFIFR	0	1	0	0	1	2
CLYDSYLPYIFC	0	0	0	0	1	2
CLYDTFLPFIYC	0	0	0	0	2	2
CLYDPFLPFIPC	0	0	0	0	1	2
CNFDIHMPYIPC	0	0	0	0	3	2
CLFGSFLPFIVC	0	0	0	4	2	2
CLFDSFLPIIFC	0	0	0	0	1	2
CLFDSFLPYILC	0	0	0	1	9	2
CLFDSFLSFIPC	0	0	0	3	3	2
CLFDSFLTFIPC	0	0	0	1	0	2
CLFDSFMPFIPC	0	0	0	0	2	2
CLFDSFVAFIFC	0	0	0	1	4	2
CLFDSFLPFIQC	0	0	0	0	0	2

CLFDSFVPFAFC	0	0	1	9	23	2
CLFDSFDPFVFC	0	0	0	7	5	2
CLFDSFGPFIFC	0	0	0	1	2	2
CLFDSFIPFIYC	0	0	0	0	2	2
CLFDSFIPFVLC	0	0	0	0	4	2
CLFGIFCPFILC	0	0	0	6	11	2
CLFGIFLPFVFC	0	0	0	0	0	2
CLFGLFFPFIIC	0	0	0	1	0	2
CLFGLFVPFVFC	0	0	2	4	4	2
CLFGLHLPFIFC	0	0	0	0	5	2
CLFGLYCDFIFC	0	0	0	2	0	2
CLFDVFCGFIFC	0	0	0	9	8	2
CLFDTFVTFIFC	0	0	0	1	11	2
CLFDTFVRFIFC	0	0	0	0	5	2
CLFDTFVQFIIC	0	0	0	0	1	2
CLFDSLLPFIYC	0	1	0	2	34	2
CLFDSSLPFIPC	0	0	0	0	3	2
CLFDSSVPFILC	0	0	0	0	0	2
CLFDSSVPFVFC	0	0	1	4	11	2
CLFDSYCDFIFC	0	0	0	1	0	2
CLFELFCPFIFC	0	0	0	1	2	2
CLFDVFCDFTFC	0	0	0	8	14	2
CLFDTFYFFTPC	0	0	0	1	5	2
CLFDSLLPFICC	0	0	0	3	22	2
CLFDSFLHFIFC	0	0	0	0	1	2
CLFDSFLLFIFC	0	0	0	0	0	2
CLFDSFLPFIFS	0	0	0	0	0	2
CLFDSFLPFIGC	0	0	3	3	10	2
CLFDSLFPFIVC	0	0	20	17	10	2
CLFGSFVPFIFC	0	0	0	4	16	2
CLFDSLLPFIVC	0	0	0	5	4	2
CLFDVFCDFNFC	0	0	0	1	1	2
CLFDTHFPFIFC	0	0	0	0	0	2
CLFDTHFPFIPC	0	0	0	1	4	2
CLFDTHFPFIVC	0	0	0	0	0	2
CLFDTHMPFIPC	0	0	0	0	0	2
CLFDTILPFILC	0	0	5	13	4	2
CLFDVFCDFISC	1	0	0	11	7	2
CLYDTFLPFIIC	0	0	0	0	1	2
CLYDSFSPFIIC	0	0	0	0	0	2
CVFDSFLPFVSC	0	0	0	1	3	2
CLFVSFVPFIFC	0	0	0	2	4	2
CLFVTFLPFIFC	0	0	0	3	4	2
CLFVTFVPFIFC	0	0	0	1	3	2
CLFYSFLPFILC	0	0	0	1	1	2
CLFYSFVPFIFC	0	0	0	1	4	2
CLFYTFLPFILC	0	0	0	0	0	2
CLIDIFLPFIYC	0	0	0	0	0	2
CLIDLFCPFIFC	0	0	0	4	5	2
CLIDLHLPFIFC	0	0	0	1	1	2
CLIDTFLPFIYC	0	0	0	0	0	2
CLLDIFCPFIFC	0	0	0	0	2	2
CLLDSFLPFIYC	0	0	0	0	0	2
CLLDVFCDFIFC	0	0	0	12	7	2
CLYDIHMPFIPC	0	0	0	0	0	2
CLYDSFMPFILC	0	0	0	0	2	2
CVYDTFVPFIPC	0	0	0	0	5	2
CVYDTSVPFIIC	0	0	0	0	1	2
CVYDLFLPFFYC	0	1	0	0	7	2
CTFHTFLPFIFC	0	0	0	2	4	2
CVFDVFFPFILC	0	0	0	0	0	2
CLSDLFCPFILC	0	0	0	0	0	2
CLSDSFLPFIVC	0	0	0	1	0	2
CLSDSFVPFVFC	0	0	0	3	7	2
CLSDIFCPFIYC	0	0	0	0	1	2
CLNDSYLPFILC	0	0	0	0	0	2
CLNDTFDPFIFC	0	0	0	0	1	2
CLYDLFFPFIIC	0	0	0	1	1	2
CLYGSFLPFIYC	0	0	0	0	1	2
CLYDYYLPFIFC	0	0	0	0	0	2
CLYDYFLPFIYC	0	0	0	0	1	2
CLYDSFFPFIIC	0	0	0	0	0	2
CLYDSFKPFIFC	0	0	0	0	1	2
CLYDSFLLFIFC	0	0	0	3	5	2
CLYDSFLPFLCC	0	0	1	0	11	2
CLYDSFLPFTLC	0	0	0	0	0	2
CLYDSFLPFTYC	0	0	0	0	2	2
CLYDSFLPFVIC	0	0	0	2	8	2
CLYDSFLPFVLC	0	0	0	1	13	2
CLYDSFLPYIIC	0	0	0	0	0	2
FDTFLPFIIC	0	0	0	0	1	2
CNFDIHKPIIPC	0	0	0	0	0	2
CNFDIHIPFIPC	0	0	0	0	0	2
FDLFIPFVFC	0	0	1	2	11	2
CLFVIFLPFIFC	0	0	0	3	7	2
CLYDPFLPFIIC	0	0	0	0	0	2
CLYDPFLSFIPC	0	0	0	3	3	2
FDIFLPFIIC	0	0	0	0	2	2
FDLFCPFVMC	0	0	0	5	1	2
FDLFCPFVYC	0	0	0	0	0	2
FDLFVPFIFC	0	0	0	0	2	2
CNFDIFLPFIIC	0	0	0	0	1	2
FDLFVPFVFC	0	0	0	1	3	2
FDLHLPFIFC	0	0	1	0	2	2
CLYVSFLPFIFC	0	0	1	3	10	2
FDSFLPFITC	0	0	0	0	1	2
CNFDIFLPFIFC	0	0	1	1	1	2
CLFDSYLPFILC	0	0	0	4	5	2
CLFDSYLPYIFC	0	0	0	0	0	2
CLFDTFCPFIIC	1	0	0	0	2	2
CTFDTHMPFIYC	0	0	0	2	6	2
CPYDSFLPFIIC	0	0	0	0	0	2
CPYDSFLPFILC	0	0	0	0	2	2
CPFDVFCDFIFC	0	0	0	19	7	2

CQFDTFLPFIFC	0	0	0	0	1	2
CQFDLHLPFIFC	0	0	0	0	5	2
CPFDPFLSFIPC	0	0	0	16	7	2
CPFDLFLPFIPC	0	0	0	0	0	2
CPFDIFLPFIPC	0	0	1	0	1	2
CNFDPHFIFIPC	0	0	0	1	4	2
CNFDTFMPFIFC	0	0	0	0	0	2
CNFHIHKPFIPC	0	0	0	0	0	2
CNIHIHMPFIPC	0	0	0	0	0	2
CTFDIFLPFIHC	0	0	0	4	15	2
CTFDIFLPFILS	0	0	0	0	2	2
CLFDTFFPFIIC	0	0	0	1	1	2
CTFDIFLPFIMC	0	0	0	0	0	2
CTFDPHFIFIPC	0	0	0	0	3	2
CTFDRFLPFIFC	0	0	0	1	2	2
CTFDSLLPFIYC	0	0	0	3	9	2
CTFDSWLPFIPC	0	0	0	0	3	2
CTFDTFLLFIFC	0	0	0	0	2	2
CTFDTFLPFINC	0	0	0	0	1	2
CTFDTFLPFIRC	0	0	0	0	4	2
CTFDTFLPFTIC	0	0	0	0	4	2
CTFDTFLPIIFC	0	0	0	0	7	2
CTFDTFLPIIYC	0	0	0	0	1	2
CTFDTFLPLICC	0	0	0	0	1	2
CTFDTFLPLILC	0	0	0	0	2	2
CTFDTFLPVIFC	0	0	1	0	0	2
CNWDSFVPFIFC	0	0	0	0	1	2
CNYDIHKPFIPC	0	0	0	0	2	2
CSYDLFLPFIFC	0	0	0	5	17	2
CSFDTFLPFIIC	0	0	0	0	3	2
CSFDTFLPFILC	0	0	0	0	1	2
CRFDSFLPFILC	0	0	0	0	2	2
CRFDSFLPFIPC	0	0	0	0	1	2
CTFDAFLPFIIC	0	0	0	0	3	2
CSFDSFLPFVIC	0	0	0	0	3	2
CSFDLFLPFIIC	0	0	0	0	1	2
CSFDLFVPFIFC	0	0	0	1	2	2
CSFDSFLPFICC	0	0	0	0	0	2
CSFDSFLPFIIC	0	0	0	0	0	2
CSFDTFLPFIPC	0	0	0	0	0	2
CTFDIFCPFILC	0	0	0	0	1	2
CNYDIHMPFIPS	0	0	0	0	0	2
CNYDIHMPYIPC	0	0	0	0	1	2
CNYDIQMPFIPC	0	0	0	0	0	2
CPFDIFCPFIFC	0	0	0	0	3	2
CNLDIHMPFIPC	0	0	0	0	0	2
CTFDIFIPFVLC	0	0	0	1	3	2
CTFDPFLPFIPC	0	0	0	0	2	2
CTFDPFLPFIIC	0	0	0	0	4	2
CTFDTFLPYIFC	0	0	1	5	8	2
CTFDIFLPSILC	0	0	0	2	7	2
CTFDLFLPYIYC	0	0	0	0	3	2

CTFDLFLTFIYC	0	0	0	0	2	2
CTFDLFSPFIYC	0	0	0	0	0	2
CTFDLFVPFVLC	0	0	1	4	10	2
CTFDLILPFIIC	0	0	0	0	3	2
CTFDLLLPFIYC	0	0	0	1	2	2
CTFDLSLPFIIC	0	0	0	0	1	2
CTFDLSLPFIYC	0	0	0	0	2	2
CTFDLYLPYIYC	0	0	0	0	0	2
CTFDLFMPFIIC	0	0	0	0	0	2
CTFDNFLPFIFC	0	0	0	0	5	2
CTFDLFLHFIYC	0	0	0	0	0	2
CTFDIFLPFNLC	0	0	1	0	2	2
CTFDLFLPSIYC	0	0	0	1	2	2
CTFDLFLPLILC	0	0	0	1	7	2
CTFDLFLPFTCC	0	0	0	0	4	2
CLFDTFVPFNFC	2	0	0	3	6	2
CLFDTFLPCIFC	0	0	0	0	0	2
CLFDTFLPFIHC	0	0	0	0	0	2
CLFDTFVPFIFS	0	0	0	3	5	2
CLFDTFVPFIMC	0	0	0	0	1	2
CLFDTFVPFIRC	0	0	0	0	2	2
CLFDTFVPLILC	0	0	0	0	3	2
CTFDLFLPFNIC	0	0	0	0	0	2
CLFDSLLPFISC	0	1	0	3	5	2
CNFDLFDPFIFC	0	0	0	0	1	2
CLFDSFLPFIHC	0	0	2	3	14	2
CNFDNWHPFIYC	0	0	0	0	0	2
CTFDLFLPFIFW	0	0	0	1	1	2
CTFDLFLPFIRC	0	0	0	0	1	2
CTFDIFLPLIFC	0	0	0	0	6	2
CTFDIFLPYIFC	1	0	0	0	2	2
CTFDTFPPFIYC	0	0	0	0	0	2
CTFDIFMPFIYC	0	0	0	0	0	2
CTFDTHFPFTTC	0	0	0	4	2	2
CTFDTHFPFVTC	0	0	0	4	1	2
CTFDTHLPFVYC	0	0	0	2	10	2
CTFDTILPFIYC	0	0	0	0	0	2
CTFDTHFPFIMC	0	0	0	1	1	2
CTFDTLLPFIIC	0	0	0	0	0	2
CTFDVFLPFISC	0	0	0	7	11	2
CTFDVFLPFIVC	0	0	1	0	11	2
CTFDVFLPFLCC	0	0	0	0	1	2
CTFELFLPFIFC	1	0	0	7	7	2
CTFELFLPFIYC	0	0	0	0	0	2
CTFGIFLPFILC	0	0	1	6	5	2
CNFDLFSPFIFC	0	0	0	0	0	2
CTFDVFCPFIFC	0	0	0	1	1	2
CTFDTFVPFILC	0	0	0	0	1	2
CTFDTHFPFNTC	0	0	0	2	1	2
CTFDTHFPFITY	0	0	0	1	5	2
CTFDTHFPFITS	0	1	0	0	4	2
CTFDLFFPFIFC	0	0	0	2	8	2

CTFDIHIPFIPC	0	0	0	0	0	2
CTFDINMPFIPC	0	0	0	0	0	2
CTFDIYLPFIFC	0	0	0	0	0	2
CTFDLFCPFIPC	0	0	0	0	2	2
CTFDLFFPFIPC	0	0	0	1	5	2
CTFDNWLPLIYC	0	0	0	0	0	2
CTFDLFIPFIPC	0	0	0	3	11	2
CTFDLFLAFIFC	0	0	0	0	3	2
CTFDLFLHFIIC	0	0	0	0	0	2
CTFDLFAPFIFC	0	0	0	1	3	2
CTFDNFQPFIFC	0	0	0	0	0	2
CTFDNWLPFILC	0	0	0	0	0	2
CVYDTFVPFIMC	0	0	0	0	0	2
CVYDSLLPFIFC	0	0	0	0	1	2
CVYDSFLPFVLC	0	0	0	2	6	2
CTYDIHMPFIPC	0	0	0	0	1	2
CTFVTFLPFIFC	0	0	1	1	14	2
CTFYLFLPFIFC	0	0	0	1	7	2
CTIDLFLPFIYC	0	0	0	0	1	2
CTIDLYLPFIFC	0	0	0	0	0	2
CTLDIFLPFILC	0	1	0	5	7	2
CTLDLFLPFIIC	0	0	0	0	2	2
CTYDIFLPFILC	0	0	0	1	6	2
CTLDTFLPFILC	0	0	0	0	3	2
CTSDTFLPFIIC	0	0	0	0	0	2
CTSDTHFPFITC	0	0	1	2	4	2
CTWDLFFPFILC	1	0	15	49	28	2
CTWDSFVPFIIC	0	0	0	0	1	2
CTWDSFVPFIPC	0	0	0	0	0	2
CTWDSFVPFIYC	0	0	0	0	0	2
CTLDTHLPFIYC	0	0	1	5	5	2
CTWDSFVPLIFC	0	0	0	0	1	2
CVFDIHMPFIPR	0	0	0	0	1	2
CVFDLFMPFIIC	0	0	0	0	1	2
CVFDIFCPFTFC	0	0	0	0	0	2
CTYDLYLPFIFC	0	0	0	1	0	2
CVCDIFVPFIFC	0	0	0	0	0	2
CVFALFLPFIFC	0	0	0	0	2	2
CVFDCFLPFIPC	0	0	0	0	0	2
CVFDFHMPFIPC	0	0	0	0	1	2
CVFDIFCPFIPC	0	0	0	0	2	2
CTYDLFLPFILC	0	0	0	0	1	2
CVFDIFCPFIYC	0	0	0	0	3	2
CVFDIFIPC	0	0	0	0	5	2
CVFDIFMPFIVC	0	0	0	0	0	2
CVFDIFMPFVLC	0	0	0	3	1	2
CVFDIFVPFIPC	0	0	0	0	3	2
CVFDIFVPFIYC	0	0	0	0	0	2
CVFDIHMPFIIC	0	0	0	0	2	2
CVFDIHMPFIPS	0	0	0	0	1	2
CVFDIHMPSIPC	0	0	0	1	4	2
CVFDILMPFIPC	0	0	0	0	1	2

CVFDLFVPFIVC	0	0	0	0	1	2
CVFDLFPPFTFC	0	0	1	3	4	2
CVFDISIPFVLC	0	0	1	2	4	2
CVFDIYIPFVLC	0	0	1	2	3	2
CVFDLCLPFIFC	0	0	0	0	1	2
CVFDLFCPFVFC	0	0	0	0	0	2
CVFDLFDPFIFC	0	0	0	0	17	2
CVFDLFFPFILC	0	0	0	0	3	2
CVFDLFFTFIFC	0	0	0	0	0	2
CVFDLFLHFIFF	0	0	0	0	0	2
CVFDLFLPFFFC	0	0	0	0	1	2
CVFDLFLPFTSC	0	0	0	7	17	2
CVFDLFLPLIIC	0	0	0	0	0	2
CVFDLFLPVIFC	0	0	0	0	1	2
CVFDLFLPYIIC	0	0	0	0	0	2
CVFDLFLRFIFC	0	0	0	0	1	2
CVFDLFSPFIIC	0	0	0	0	1	2
CVFDLFSPFILC	0	0	0	0	0	2
CVFDLLLPFIYC	0	0	0	0	1	2
CVFDLFVPFICC	0	0	0	0	0	2
CVFDLFVPFVYC	0	0	0	0	1	2
CVFDLFWPFIFC	0	0	0	0	3	2
CVFDLHCPFICC	0	0	0	2	18	2
CVFDLHCPFTFC	0	0	2	4	4	2
CVFDLHMPFIPC	0	0	0	0	1	2
CVFDLLLPFILC	0	0	0	0	2	2
CVFDLLLPFIPC	0	0	0	0	0	2
CVFDNFLPFILC	0	0	0	0	0	2
CVFDLSLPFILC	0	0	0	0	2	2
CVFDLWLPFIPC	0	0	0	0	1	2
CVFDLWLPFTAC	0	0	0	6	6	2
CVFDLWLPFTPC	0	0	0	0	0	2
CVFDLYLPFIYC	0	0	0	0	0	2
CVFDMFLPFIFC	0	0	0	0	0	2
CTYDLFLPFIYC	0	0	0	1	3	2
CVFDNFLPFIPC	0	1	0	0	3	2
CVFDPFLPFIYC	0	0	0	0	3	2
CVFDYLLPFIFC	0	0	0	3	0	2
CVFDSFIPFIIC	0	0	0	0	0	2
CVFDSFIPFILC	0	0	0	0	0	2
CVFDSFLLFIPC	0	0	0	0	0	2
CVFDSFLPFELC	0	0	1	1	5	2
CVFDSFLPFFLC	0	0	0	0	1	2
CVFDSFLPFIFR	0	0	0	0	0	2
CVFDSFLPFIHS	0	0	0	0	0	2
CVFDSFLPFVCC	0	0	0	1	1	2
CVFDSFLHFILC	0	0	0	0	1	2
CVFDSLLPIILC	0	0	0	0	0	2
CVFDSLLPLIFC	0	0	0	5	11	2
CVFDSLPPFIFC	0	0	0	3	8	2
CVFDVICPFIFC	0	0	0	0	1	2
CVFDSFIPFIFC	0	0	0	0	1	2

CVFDSFFPFITC	0	0	0	1	3	2
CVFDSIMPFIIC	0	0	0	0	1	2
CVFDSFLTFIMC	0	0	0	0	0	2
CVFDSFMPFIVC	0	0	0	0	1	2
CVFDSFMPYIIC	0	0	0	0	1	2
CVFDSFVPLIIC	0	0	0	0	0	2
CVFDSFWPFIPC	0	0	0	0	1	2
CVFDSILPFIFC	0	0	0	0	1	2
CVFDSIMPIIIC	0	0	0	0	1	2
CVFDSCLPFIFC	0	0	0	0	0	2
CVFDSFLTFITC	0	0	0	0	0	2
CVFDSLLPFIFS	0	0	0	0	1	2
CVFDSFLPFVLR	0	0	0	0	2	2
CVFDSFLPFVIS	0	0	0	0	0	2
CVFDPLLPFIFC	0	0	1	4	5	2
CVFDRFLPFILC	0	0	0	0	0	2
CVFDVYCPFIFC	0	0	0	2	6	2
CVFEIHMPFIPC	0	0	0	0	1	2
CVFNSWLPFVFC	0	0	0	0	0	2
CVFELFLPFTFC	0	0	0	1	2	2
CVFDSWLPFAFC	0	0	0	0	2	2
CVFDSWLPFICC	0	0	0	0	0	2
CVFDSWLPFVVC	0	0	0	0	3	2
CVFDSWLPLIIC	0	0	0	0	0	2
CVFDSWLPLVFC	0	0	0	2	0	2
CVFDSWLPSVYC	0	0	0	2	4	2
CVFDSYLPFIPC	0	0	0	0	2	2
CVFDSYLPFISC	0	0	0	0	0	2
CVFDSYLPFNIC	0	0	0	0	0	2
CVFDTFLPFIVC	0	0	0	1	2	2
CVFDTFLPFVFC	0	0	0	0	2	2
CVFDTFLPVIFC	0	0	0	0	0	2
CVFDTFMPFIFC	0	0	0	0	0	2
CVFDTFMPFIIC	0	0	0	0	2	2
CVFDPFLPFVLC	0	0	1	4	11	2
CVFDSVLPFIIC	0	0	0	0	5	2
CVFDSSLPFIPC	0	0	0	0	1	2
CVFDSPLPFIFC	0	0	0	3	8	2
CVFHLFLPFIYC	0	0	0	0	0	2
CVFESFLPFIFC	0	0	0	0	0	2
CVFGLFLPFILC	0	0	0	0	0	2
CVFDVFSPFIFC	0	0	0	3	6	2
CVFGSFLPFIPC	0	0	0	0	2	2
CVFGSLLPFIFC	0	0	0	1	1	2
CVFGSWLPFVYC	0	0	0	0	2	2
CVFHSFLPFVLC	0	0	0	0	1	2
CVFDTMLPFIFC	0	0	0	0	0	2
CVFNLFLPFTFC	0	0	0	0	1	2
CVFNSFLPFIPC	0	0	0	0	1	2
CVFNSFLPFVLC	0	0	0	0	6	2
CVFGSFLPFVLC	0	0	0	4	8	2
CVFDVFCQFIFC	0	0	0	4	9	2

CVFDVFCPFIFR	0	0	0	1	2	2
CVFDLFLSFIIC	0	0	0	0	0	2
CVFDLFMPYIVC	0	0	0	0	2	2
CVYDSFLPFIVC	0	0	0	0	0	2
CVYDIYEPFINC	0	0	0	0	0	2
CVYDIFVPINIC	0	0	0	0	0	2
CVYDIFEPFIMC	0	0	0	1	0	2
CVYDIFCPFILC	0	0	0	0	0	2
CVYDFFLPFIYC	0	0	0	1	2	2
CVFVVFCPFIFC	0	0	0	0	5	2
CVHDLFLPFIPC	0	0	0	0	1	2
CVIDSFLPFIPC	0	0	0	0	1	2
CVIDSFMPFIIC	0	0	0	0	1	2
CVIDVFCPFIFC	0	0	0	1	5	2
CVLDLFLPFILC	0	0	0	0	0	2
CVLDLFLPFIPC	0	0	0	0	1	2
CVNDIFVPFIFC	0	0	0	1	0	2
CVSDIFIPFVLC	0	0	0	1	7	2
CVSDSLLPFIFC	0	0	0	1	4	2
CVYDIYVPFIFC	0	0	0	0	0	2
CVYDISVPFILC	0	0	0	0	0	2
CVFDPFLPFIPC	0	0	0	0	2	2
CVYDISVPFIFC	0	0	0	0	1	2
CYFDPHFIFIPW	0	0	0	1	0	2
CYFDPHFIFINC	0	0	0	0	1	2
CYFDPHFIFIIC	0	0	0	1	2	2
CVYDIFLPFIPC	0	0	0	0	0	2
CVYDIFVAFIIC	0	0	0	0	1	2
CVYDIFVLFIFC	0	0	0	0	2	2
CVYDIFVPFFFC	0	0	0	0	1	2
CVYDIFVPFIFS	0	0	0	0	0	2
CVYDIFVPFIIG	0	0	0	0	1	2
CVYDIFVPFTLC	0	0	0	0	0	2
CVYDIFLHFIIC	0	0	0	0	0	2
CVYDIFVPFVLC	0	0	0	0	12	2
CVYDIFVTFIFC	0	0	0	0	0	2
CVYDIIVPFINC	0	0	0	0	1	2
CVYDILVPFILC	0	0	0	0	2	2
CVVDLFVPYIVC	0	0	0	0	0	2
CVWDSFVPFIPC	0	0	0	0	4	2
CVWDSFVPFIVC	0	0	0	0	4	2
CVYYIFVQFIIC	0	0	0	0	0	2
CVYVLFLPFIFC	0	0	0	0	1	2
CVYDWFLPFIYC	0	0	0	1	2	2
CVYDLFLPFLCC	0	0	0	0	9	2
CVYDLFLPFVFC	0	0	0	0	10	2
CVYDLFLPFVIC	0	0	0	0	11	2
CVYDLFLSFIFC	0	0	0	0	0	2
CVYDLFLSFIPC	0	0	0	0	0	2
CVYDLFMPFIFC	0	0	0	0	2	2
CVYDLSLPFIFC	0	0	0	0	9	2
CVYDLSLPFIPC	0	0	0	0	3	2

CVYDLYLPFIFC	0	0	0	0	4	2
CVYDLYMPFIYC	0	0	0	1	1	2
CVYDSFLPFINC	0	0	0	0	0	2
CYFDPHFIFC	0	0	0	0	15	2
CYFDLFVPFIFC	0	0	0	0	0	2
CYFDIFPPFIFC	0	0	0	3	3	2
CVYDIYVPFIPC	0	0	0	1	0	2
CTFNLFLPFIYC	0	0	0	0	1	2
CVYDLFLLFIFC	0	0	0	1	1	2
CVYDLFLPFIC	0	0	0	0	0	2
CVYYLFLPFIFC	0	0	0	0	1	2
CYFDPHFIFIQC	0	0	0	0	3	2
SIFDLFVPFIFC	0	0	0	0	3	2
SIFDIHMPFTPC	0	0	0	0	1	2
GIFDTFLPFIFC	0	0	0	1	2	2
FIFDIFMPFIIC	0	0	0	0	0	2
RCFDIFLPFIFC	0	0	0	0	1	2
CYFDPHFIYIPC	0	0	0	4	9	2
CYFDPHFNFIPC	0	0	0	0	3	2
CYFDPYFIFIPC	0	0	0	2	5	2
CYFDSFLPFVIC	0	0	0	0	2	2
CYFDTHFIFITC	0	0	0	0	1	2
FVFDVFCPFIFC	0	0	0	0	2	2
FLFDTFVPFIFC	0	0	0	1	2	2
FLFDSFLPFIFC	0	0	0	0	0	2
CVFNVFCPFIFC	0	0	0	1	8	2
FLFDLFCPFIFC	0	0	0	2	4	2
CYYDPHFIFIPS	0	0	0	0	0	2
FIFDNWLPFIFC	0	0	0	0	0	2
FIFDTFLPFILC	0	0	0	0	1	2
SIFDIFMPFIPC	0	0	0	0	1	2
SLFDTFVPFIFC	0	0	0	1	2	2
SLFDTFLPFIFC	0	0	0	2	2	2
SIFDTFLPFIYC	0	0	0	0	1	2
RFYDLFLPFIFC	0	0	0	2	4	2
RIFDIFMPFIFC	0	0	0	0	2	2
RIFDNWLPFIFC	0	0	0	0	0	2
RISDIHMPFIPC	0	0	0	0	0	2
RIWDSFVPFILC	0	0	0	0	7	2
RLFDLFCPFIFC	0	0	1	0	0	2
RLFDSFLPFILC	0	0	0	0	3	2
RVFDIFIPFVLC	0	0	0	0	1	2
RVFDSWLPFVFC	0	0	0	0	0	2
RVFDTFLPFIFC	0	0	0	0	0	2
RVFDVFCPFIFC	0	0	0	1	9	2
SIFDIFMPFIFC	0	0	0	0	0	2
CLFDPFCPFILC	0	0	0	2	3	2
YVYDIFVPFIIC	0	0	0	0	5	2
SVYDLFLPFIYC	0	0	0	2	4	2
RCFDSFLPFIFC	0	0	0	1	4	2
SVFDSFLPFIFC	0	0	0	0	2	2
SVYDIFVPFIFC	0	0	0	0	0	2
WIFDTFLPFIFC	0	0	0	0	1	2
YVFDSFLPFIFC	0	0	0	0	0	2
WVYDIFVPFIIC	0	0	0	0	1	2
STFDIFLPFILC	0	0	0	1	4	2
YIWDSFLPFIIC	0	0	0	0	0	2
YLFDIFLPFIFC	0	0	0	1	4	2
YLFDSFLPFILC	0	0	0	0	1	2
YTFDLFLPFILC	0	0	0	0	0	2
YYFDPHFIFIPC	0	0	0	2	3	2
